# Conformational Change of H64 and Substrate Transportation: Insight Into a Full Picture of Enzymatic Hydration of CO_2_ by Carbonic Anhydrase

**DOI:** 10.3389/fchem.2021.706959

**Published:** 2021-07-09

**Authors:** Yuzhuang Fu, Fangfang Fan, Yuwei Zhang, Binju Wang, Zexing Cao

**Affiliations:** ^1^ State Key Laboratory of Physical Chemistry of Solid Surfaces and Fujian Provincial Key Laboratory of Theoretical and Computational Chemistry, College of Chemistry and Chemical Engineering, Xiamen University, Xiamen, China; ^2^ School of Biological and Chemical Engineering, Zhejiang University of Science and Technology, Hangzhou, China

**Keywords:** carbonic anhydrase, QM/MM, enzymatic cycle, CO2 conversion, MD simulations

## Abstract

The enzymatic hydration of CO_2_ into HCO_3_^−^ by carbonic anhydrase (CA) is highly efficient and environment-friendly measure for CO_2_ sequestration. Here extensive MM MD and QM/MM MD simulations were used to explore the whole enzymatic process, and a full picture of the enzymatic hydration of CO_2_ by CA was achieved. Prior to CO_2_ hydration, the proton transfer from the water molecule (WT1) to H64 is the rate-limiting step with the free energy barrier of 10.4 kcal/mol, which leads to the ready state with the Zn-bound OH^−^. The nucleophilic attack of OH^−^ on CO_2_ produces HCO_3_^−^ with the free energy barrier of 4.4 kcal/mol and the free energy release of about 8.0 kcal/mol. Q92 as the key residue manipulates both CO_2_ transportation to the active site and release of HCO_3_^−^. The unprotonated H64 in CA prefers in an inward orientation, while the outward conformation is favorable energetically for its protonated counterpart. The conformational transition of H64 between inward and outward correlates with its protonation state, which is mediated by the proton transfer and the product release. The whole enzymatic cycle has the free energy span of 10.4 kcal/mol for the initial proton transfer step and the free energy change of −6.5 kcal/mol. The mechanistic details provide a comprehensive understanding of the entire reversible conversion of CO_2_ into bicarbonate and roles of key residues in chemical and nonchemical steps for the enzymatic hydration of CO_2_.

## Introduction

**FIGURE 1:**
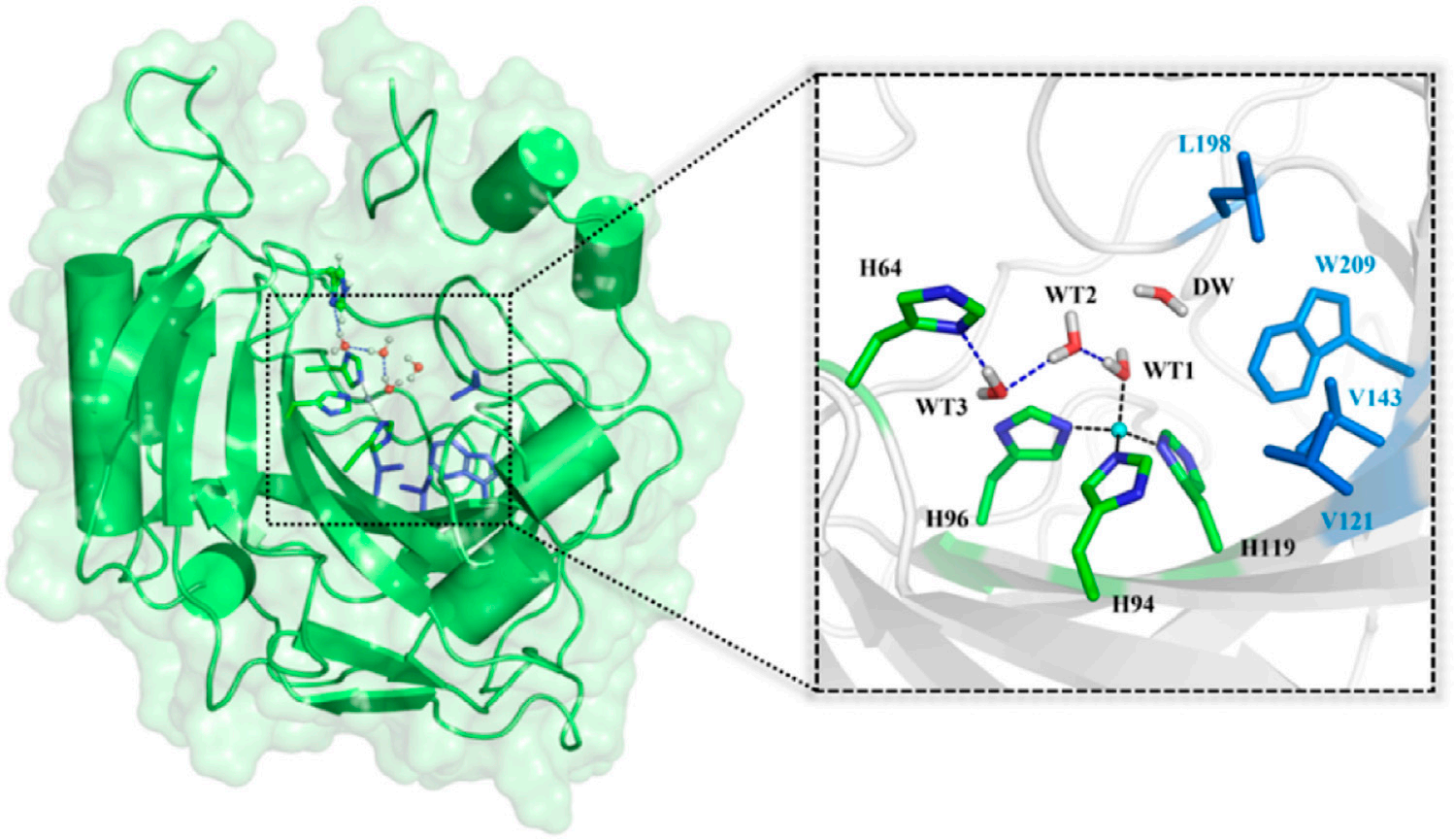
Structures of CA and the selected active domain highlighted in the dotted box, where the zinc ion is coordinated with H94, H96, H119, and WT1, and the residues colored in blue represent the hydrophobic pocket in the active site of the equilibrated ready state of CA prepared based on the X-ray crystal structure (PDB code: 2CBA).

CO_2_ reduction and carbon neutral have been constantly drawing attention in human societies. In order to alleviate and finally solve the ecological and environmental problems caused by the increased emissions of the industrial byproduct CO_2_, lots of efforts to its capture, sequestration, and conversion (Jo et al., 2014; Sanz-Pérez et al., 2016; Darunte et al., 2016; Kim et al., 2018; Kar et al., 2018) have been made in both academic and industry communities. Carbonic anhydrase (CA), as the first recognized zinc-containing enzyme, can efficiently catalyze the interconversion between CO_2_ and bicarbonate (HCO_3_^−^), which has been widely used in biological capture and sequestration of CO_2_. (Krishnamurthy et al., 2008; Vinoba et al., 2011; Vinoba et al., 2013; Alvizo et al., 2014). As shown in Figure 1, the active site of CA contains a zinc ion coordinated by three histidine residues (H94, H96, and H119) and one water molecule (WT1) (Håkansson et al., 1992) and a stable hydrogen bond network responsible for the proton transfer between WT1 and H64. (Avvaru et al., 2010; Mikulski et al., 2013; Singh et al., 2019). The deprotonation/protonation of the imidazole side chain of H64 mediated by the water chain is generally considered as the rate-determining step, and the corresponding free energy barrier is ∼10 kcal/mol in the reaction cycle. (Silverman and Vincent, 1983; Duda et al., 2001; Maupin et al., 2009; Riccardi et al., 2010; Aggarwal et al., 2014).

**FIGURE 2:**
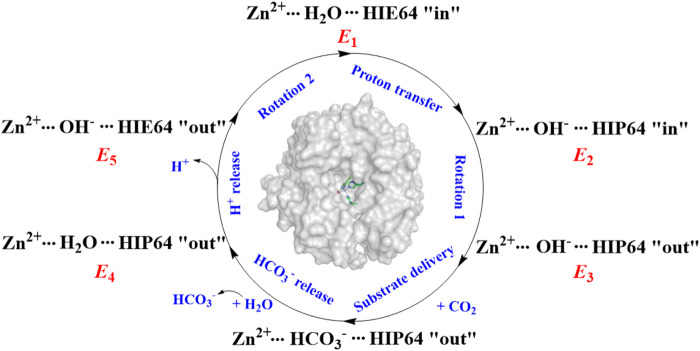
The enzymatic hydration cycle of CO_2_ by CA, where “in” and “out” stand for *inward* and *outward* conformations, respectively.

It was widely accepted that the hydration of CO_2_ is triggered by the proton transfer from WT1 to H64 through a water chain, along with the rotation of H64 side chain from *inward* to *outward* orientation. The previous studies suggest that the protonation state of H64 and WT1 in the water bridge is related to the conformational dynamics of H64 in the proton transfer and CO_2_ hydration. (Cui and Karplus, 2003; Riccardi et al., 2008; Maupin et al., 2011; Taraphder et al., 2016; Paul et al., 2018). As shown in Figure 2, the enzymatic coversion of CO_2_ into bicarbonate by CA can be described by using five states, denoted as *E*_1_, *E*_2_, *E*_3_, *E*_4_, and *E*_5_. H64, as the proton accepter, is found to have two different conformations in the crystal structure, i.e. *inward* and *outward* configurations, corresponding to HIE64 and HIP64. (Tu et al., 1989; An et al., 2002; Silverman and Mckenna, 2007). Based on some mutants of the wild-type CA, the proton transfer step and the role of the conformational change of H64 in the enzymatic hydration of CO_2_ by CA have been investigated in the past decades (Loferer et al., 2003; Roy and Taraphder, 2008; Roy and Taraphder, 2009; Piazzetta et al., 2014; Chen et al., 2017; Chen et al., 2018a; Chen et al., 2018b), and the dynamical interconversion between *inward* and *outward* conformations of H64, detected in the experiment, was not observed during the MD simulations.

Despite all these important contributions, a comprehensive understanding of the whole biological seguestration process of CO_2_ by CA and roles of key residues in CO_2_ transportation and binding to the active site, the catalytic reversible hydration of CO_2_, and the bicarbonate release, is still required. Here the entire enzymatic catalysis is divided into five chemical and nonchemical steps, where the chemical steps include the proton transfer between WT1 and H64 and the formation of HCO_3_^−^ from the nucleophilic attack of the Zn-bound OH^−^ on CO_2_, and the nonchemical steps consist of the conformational transition of H64 between *inward* to *outward*, the CO_2_ transportation from the bulk solution to the active site, and the release of HCO_3_^−^.

To build a full picture of the enzymatic hydration of CO_2_ by CA, extensive QM/MM and MM MD simulations have been carried out, and the following issues will be discussed in the present study 1) the hydrogen transfer mechanism 2) the plausible channels for CO_2_/HCO_3_^−^ transportation 3) the role of key residues and water in the conformational transition of H64, CO_2_/HCO_3_^−^ transportation, and CO_2_ hydration 4) mechanisms and corresponding dynamical and thermodynamic properties for key chemical and non-chemical steps. Here the global simulation may provide a comprehensive understanding of this enzymatic catalysis and open up an avenue to further improve the biological capture of CO_2_ by CA.

## Computational Details

### Setup of Enzyme-Substrate Complex Model

The initial enzyme model was prepared on the basis of the X-ray crystal structure of the human carbonic anhydrase II (PDB code: 2CBA) (Håkansson et al., 1992), and the initial enzyme-substrate complex was built by replacing DW (the water molecule, see Figure 1) with CO_2_. Both models with and without substrate were equilibrated by using 200 ns MM MD simulations, respectively. The protonation states of all ionizable residues were determined by using the PROPKA software (Olsson et al., 2011) at PH 7.0 and the hydrogen bond network in the X-ray crystal structure. (Fan et al., 2019a; Fan et al., 2019b). The zinc ions and its coordination sphere were parametrized by using the “MCPB.py” modeling tool. (Li and Merz, 2016; Li and Merz, 2017). The substrate and protein were described by the AMBER14SB (Gao et al., 2017) and AMBER GAFF force fields (Wang et al., 2004), respectively. The partial atomic charges of CO_2_ were determined by the restrained electrostatic potential (RESP) (Bayly et al., 1993) charge at the HF/6-31G* level with Gaussian 09. (Frisch et al., 2013). The computational model was solvated into a 95 × 93 × 101 Å cuboid TIP3P (Sun and Kollman, 1995) water box, with 10 Å buffer distances between protein atoms and the edge (Chen et al., 2018a) of the box. The whole system was neutralized by a chloride ion. The topology parameters and initial coordinates were produced by Amber *tleap* tools. The molecular dynamics simulation contains four steps, including the energy minimization to adjust poor interatomic distances, the system heating up from 0 to 300 K with a constant pressure for 1,000 ps, the equilibration under the NVT ensemble for 1,000 ps to relax the system density to about 1.0 g/cm^3^, and the final 200 ns MD simulation under the NPT ensemble with a timescale of 2 fs for the enzyme-CO_2_ complex and the enzyme system separately. The SHAKE algorithm (Miyamoto and Kollman, 1992) was used to constrain all hydrogen bonds and the cut off value was set to 10.0 Å for both van der Waals and electrostatic interactions. The root-mean-square deviation (RMSD) was applied to evaluate the stability of the enzyme backbone during MD simulations. All the above processes are accomplished by Amber18 (Case et al., 2005; Götz et al., 2012; Le Grand et al., 2013) and Gaussian 09 software. (Frisch et al., 2013).

### Umbrella Sampling

**FIGURE 3:**
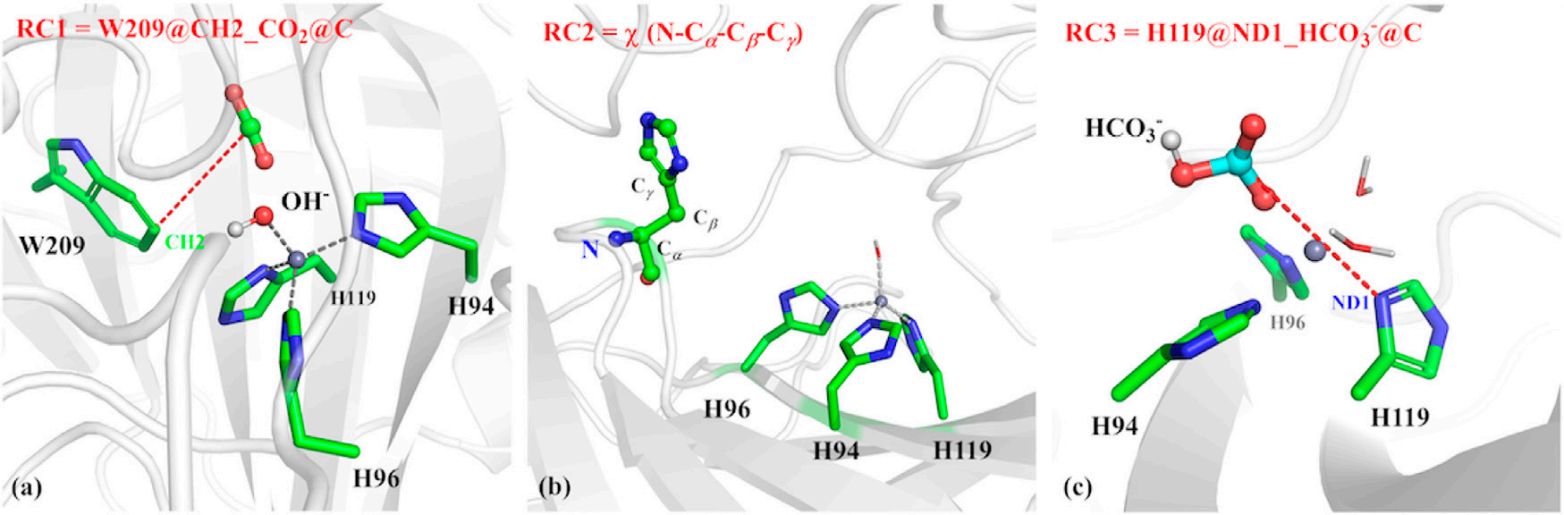
**(A)** Reaction coordinate (RC1) defined by the distance between CO_2_@C and W209@CH2 for the delivery of CO_2_, **(B)** Reaction coordinate (RC2) defined as the dihedral angle χ(N–C_*α*_–C_*β*_–C_*γ*_), **(C)** Reaction coordinate (RC3) defined by the distance between H119@ND1 and HCO_3_^-^@C.

The umbrella sampling and the weighted Histogram analysis method (WHAM) (Kumar et al., 1992; Souaille and Roux, 2001) were applied to estimate thermodynamic and dynamic properties of CO_2_ delivery in different channels and release of HCO_3_^−^, as well as the conformational transition of H64. The initial structure was selected from the stable configuration equilibrated by MD simulations. For the CO_2_ transportation, the distance between W209@CH2 and CO_2_@C was defined as the reaction coordinate (RC1, see Figure 3A), which varies from 3.4 to 18.0 Å with a 0.2 Å interval for two adjacent windows. Finally, 25 ns MD simulations with the biasing harmonic potential of 50 kcal/mol were performed for each window. According to the high-resolution crystal structure and the previous studies (Roy and Taraphder, 2009), the dihedral angle χ (N–C_*α*_–C_*β*_–C_*γ*_) was defined as the reaction coordinate (RC2, see Figure 3B), which changes from 43.87° to −38.40° with a 3° interval for two adjacent windows in the 25 ns MD simulation with a force constant of 200 kcal/mol rad (Sanz-Pérez et al., 2016), describing the conformational transition of H64 from *inward* to *outward* orientation. In order to explore the conformational change of H64, umbrella samplings under different conditions were performed: 1) the side chain of the protonated H64 rotates from *inward* to *outward* conformation without CO_2_ in the active site; 2) the side chain of deprotonated H64 rotates back from outward to inward conformation after the detachment of HCO_3_^−^. The distance between H119@ND1 and HCO_3_^−^@C was defined as the reaction coordinate to identify the release channel of HCO_3_^−^ (RC3) (see Figure 3C), which varies from 4.6 Å to 17.0 Å with a 0.2 Å interval for two adjacent windows, and 35 ns MD simulation was carried out for each window with the appropriate biasing harmonic potential. And the last 10 ns MD simulations of all above samplings were collected to generate free energy profiles by using WHAM. (Kumar et al., 1992; Souaille and Roux, 2001).

### QM/MM and QM/MM MD Simulations

QM/MM calculations were employed to explore the plausible mechanism for the proton transfer between WT1 and H64. The QM/MM calculations were performed by using the ChemShell package (Sherwood et al., 2003; Metz et al., 2014), combining Orca (Neese, 2018) and DL_POLY (Smith and Forester, 1996) for QM and MM subsystems, respectively, and the AMBER GAFF force field was employed throughout this study for the MM region. The QM/MM system consists of ∼74,000 atoms, and the QM subsystem consists of more than 120 atoms, including Y7, H64, H94, H96, E106, H119, T199, T200, zinc ion, WT1, WT2, WT3, and DW, which was described by the B3LYP functional with the Def2/TZVP and def2/J auxiliary basis set, dispersion corrections were computed with Grimme’s D3 method. (Grimme, 2006; Grimme et al., 2011). The MM subsystem was described by the AMBER force field, and the boundary was described by the charge-shift model. (Sherwood et al., 2003; Metz et al., 2014). Then a 21 psWell-Tempered Adaptively Biased Molecular Dynamics (WT-ABMD) method was carried out to get the thermodynamic characteristics and free energy profile for the hydration of CO_2_. (Barducci et al., 2008). The initial structure for WT-ABMD was obtained from 1.5 ps QM(B3LYP/6-31G**)/MM MD simulation without any restrain, the QM region for QM/MM MD and WT-ABMD consists of more than 70 atoms, including H94, H96, H119, E106, T199, hydroxyl ion, zinc ion, CO_2_ and one water molecule around the hydroxyl ion, the QM region and MM region for both of them were describe by Gaussian 09 and Amber force field, respectively. The distance between Zn-bound OH^−^@O and CO_2_@C was defined as the reaction coordinate (RC4) to describe the formation of HCO_3_^−^. The mode was set to *FLOODING*, with a timescale of 0.05 ps The resolution of reaction is set to 0.25 Å, and the reaction coordinate RC4 changes from 0.9 Å to 3.2 Å. All above calculations were carried out by the modified Orca 4.0,^52^ Gaussian 09^42^ and Amber 18 programs. (Case et al., 2005; Götz et al., 2012; Le Grand et al., 2013).

## Results and Discussions

### Equilibrium Configurations

**FIGURE 4:**
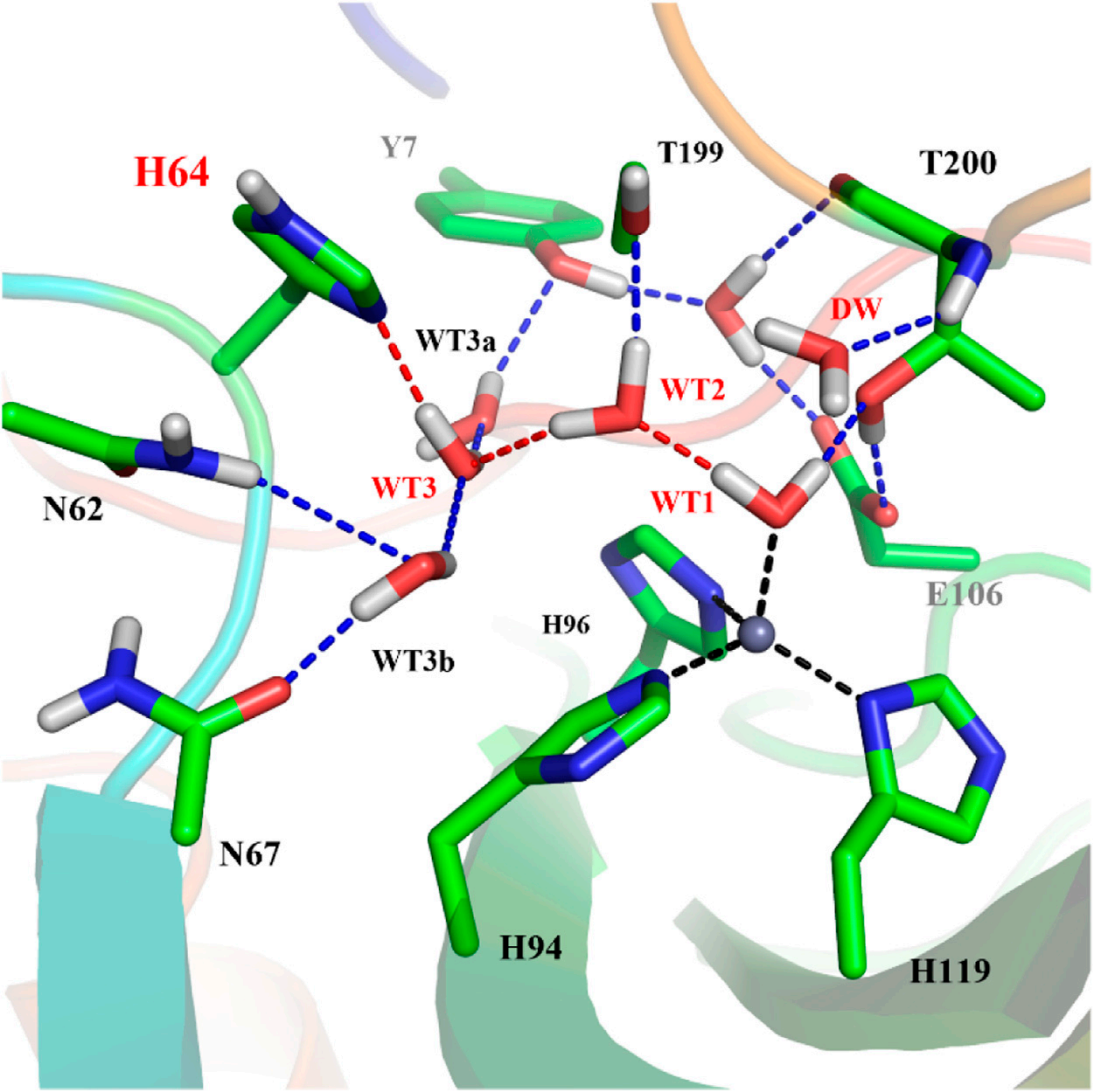
The represent structure of the active site from the MD simulation, where the blue dotted lines represent hydrogen bonds and the red dotted lines show the proton transfer pathway between WT1 and H64.

To explore the delivery channel of CO_2_ and the reaction mechanism of carbonic anhydrase, a 200 ns MM MD simulation without substrate was carried out. On the basis of the root-mean-square deviation (RMSD) (see Supplementary Figure 1), it can be noted that the CA system may become stable after 140 ns during MD simulations, and thus we choose the snapshot after 140 ns as the initial structure for the further study. The representative structure selected from the MD simulation and the QM region highlighted in the stick model are displayed in Figure 4, where the zinc ion is coordinated with three histidines (H94, H96, and H119) and one water molecule, and the residue H64 is stable at the *inward* conformation. Besides that, there is a stable hydrogen network in the active site, which may mediate the proton transfer from WT1 to H64 through a water chain containing two water molecules (WT2 and WT3). To evaluate the stability of the water chain, we counted the probability of water molecules appearing within 1.8 Å and 2.2 Å of T199@OG1, T200@H and H64@ND1, and there is a relatively high probability of 76% to form the hydrogen bond (see Supplementary Table 1). At the same time, the deeper water molecule (DW), as shown in Figure 1, surrounded by the side chain residues of V121, V143, L198, and W209 (hydrophobic pocket), will be squeezed out during the CO_2_ delivery from the bulk water to the active site. Such desolvation free energy from the displacement of the water molecule solvating the protein pocket into the bulk water may facilitate the substrate binding. In order to study the hydration of CO_2_, the water molecule (DW, refer to Figure 1) is replaced by CO_2_, and a 200 ns MM MD simulation for the enzyme-substrate system was also performed to achieve its equilibrium configuration. As shown in Supplementary Figure 2, the CA-CO_2_ complex system becomes stable after 100 ns, where CO_2_ survives in the active site with the Zn⋅⋅⋅CO_2_@C distance ranging from 3.5 to 4.5 Å, as shown in Supplementary Figure 3.

### Proton Transfer for the Ready State Formation

**FIGURE 5:**
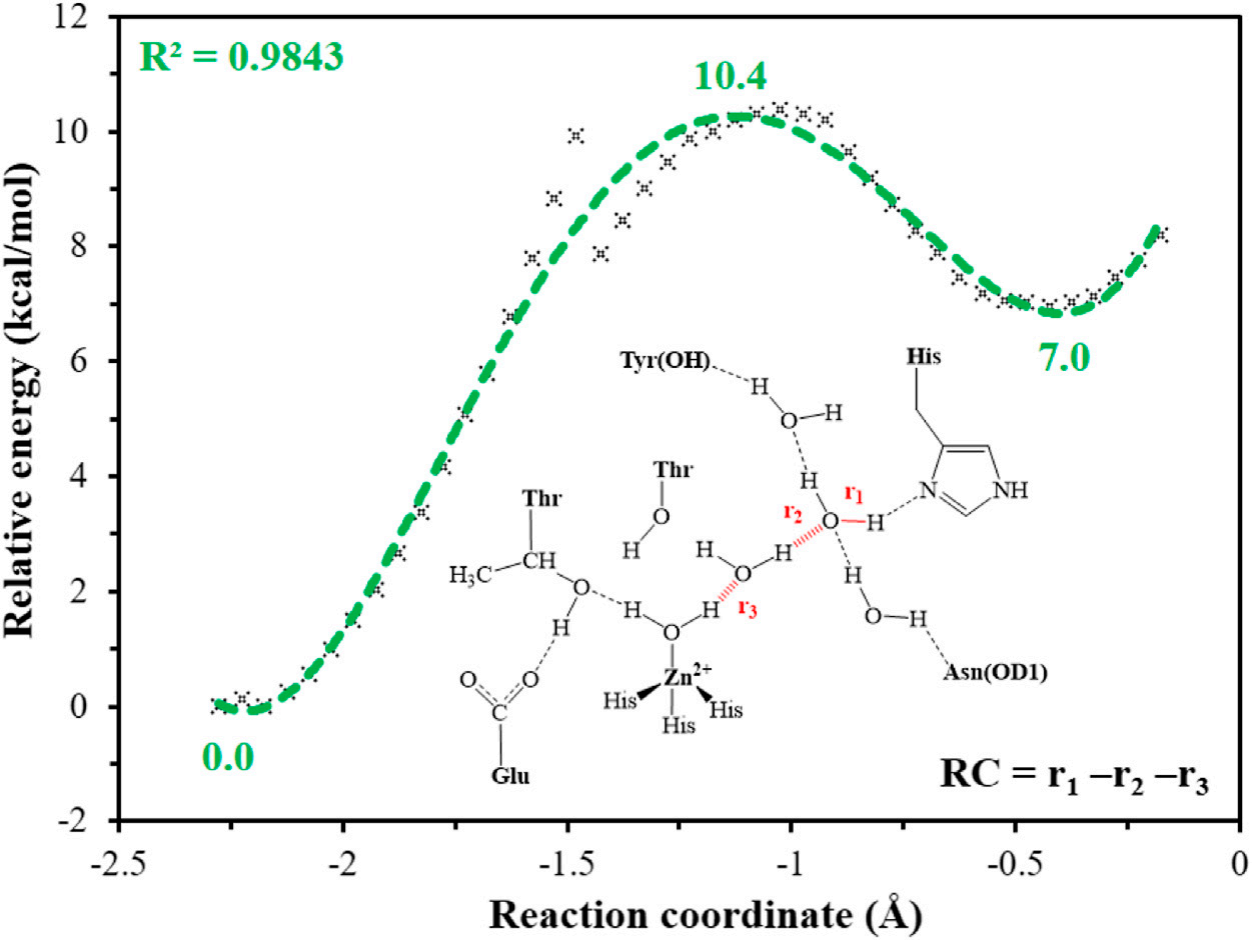
QM(B3LYP/TZVP–D3)/MM-predicted relative energy profiles for the proton transfer between H64 and WT1, where the reaction coordinate is defined as r_1_—r_2_—r_3_ (in red).

**FIGURE 6:**
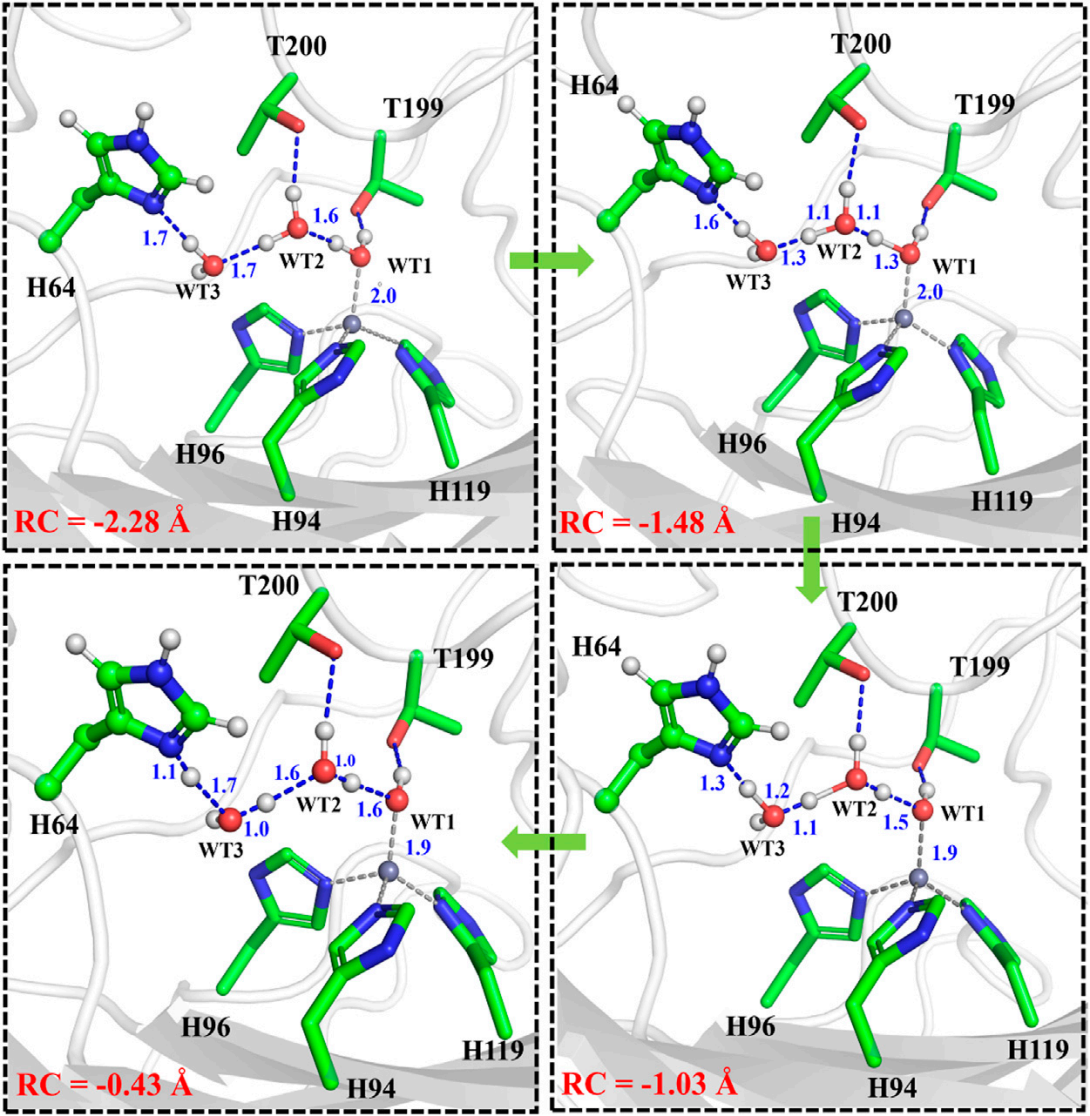
Representative structures of the active site during the proton transfer between WT1 and the residue H64.

It is generally assumed that the proton transfer from WT1 to H64 through a water chain is responsible for formation of the ready state of CA for CO_2_ hydration, and this key step has been studied by using QM(B3LYP)/MM calculations and MD simulations. As shown in Figure 5, the distance of r_1_—r_2_—r_3_ is defined as the reaction coordinate (RC) to explore the proton transfer, and the predicted free energy barrier is ∼10.4 kcal/mol, consistent with the experimental result. (Silverman and Mckenna, 2007). Selected representative conformations of the active domain, involved in the proton transfer, are shown in Figure 6. The initial proton transfer from WT1 to WT2 leads to the formation of H_3_O^+^ at RC = −1.48 Å, then WT3 accepts the proton from WT2 to reach the transition state, and finally, the proton transfers to H64 at RC = −0.43 Å. We note that Mulliken gross atomic charges of zinc maintain less changed during the proton transfer process, although it appears to fluctuate to some extent, from 0.44 to 0.42 and then to 0.48, as shown in Supplementary Figure 4. The excess negative charges from the proton transfer mainly populate the Zn-bound OH^−^. Our QM/MM results reveal that the hydrogen-bonded water chain plays a vital role in the proton transfer, as shown in previous study. (Mikulski et al., 2013). The whole proton tranfer process is in accord with the Grotthus mechanism (Agmon, 1995; Riccardi et al., 2006), and it is endothermic by 7.0 kcal/mol.

### Rotation of the Protonated H64 and Transportation of CO_2_

**FIGURE 7:**
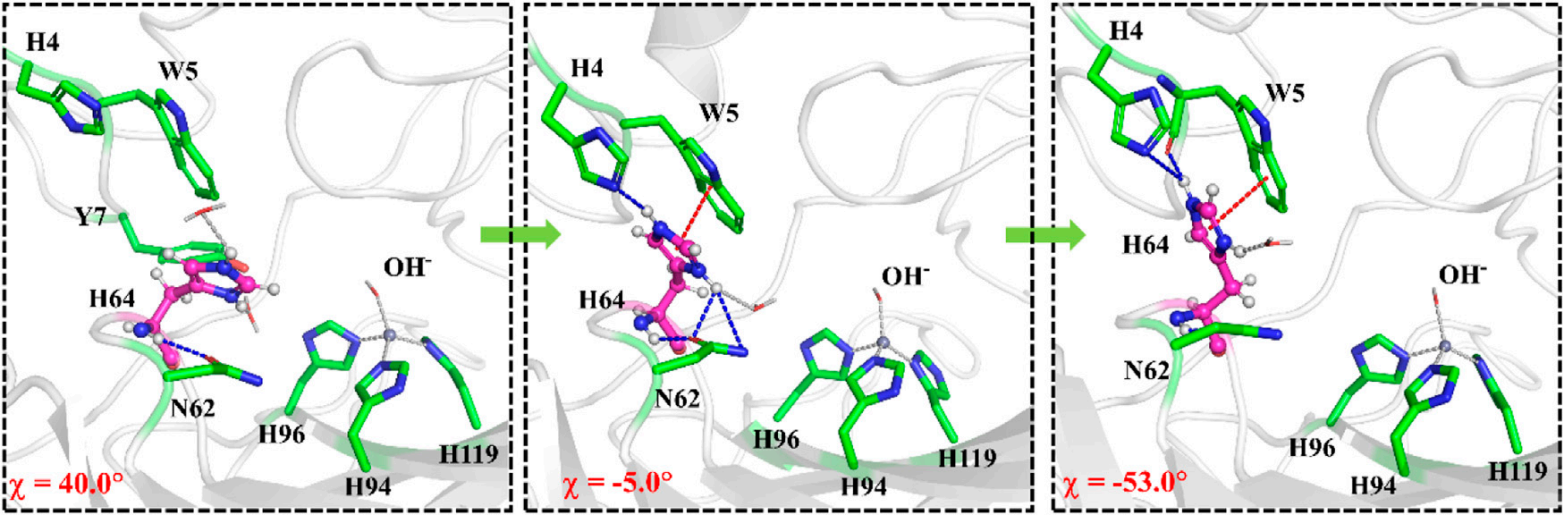
Representative structures and conformational changes for the rotation of H64 from *inward* to *outward* conformation, where the reaction coordinate is defined as the dihedral angle χ(N–C_*α*_–C_*β*_–C_*γ*_).

The umbrella sampling and the WHAM technology were used to calculate the potential of mean force (PMF) for the rotation of H64 and the transportation of CO_2_. At first, in order to describe the rotation of the protonated H64, the dihedral angle χ (N–C_*α*_–C_*β*_–C_*γ*_) was selected as the reaction coordinate (RC2), and the *inward* and *outward* conformations correspond to the dihedral angles of 40.0° and −52.0°, respectively, as shown in Figure 7. The protonated H64 rotates within a narrow channel between W5 and N62, and the driving force of the rotation may come from the hydrogen bond interaction between H64 and H4. As Figure 7 shows, the residue H4 gradually approaches to H64 and forms a hydrogen bond during the rotation, while H4 is back to the initial position and H64 evolves into its *outward* orientation when χ = −53.0°. The *outward* conformation of H64 can be stabilized by the π-π stacking interaction with the residue W5 and hydrogen bond interaction with the residue N62. As Figure 9A shows, the conformational change of the protonated H64 from *inward* to *outward* is quite facile with a free energy barrier of 0.5 kcal/mol and an energy release of 3.9 kcal/mol. The protonated H64 will maintains the *outward* conformation until the product release from the active site (*vide infra*).

**FIGURE 8:**
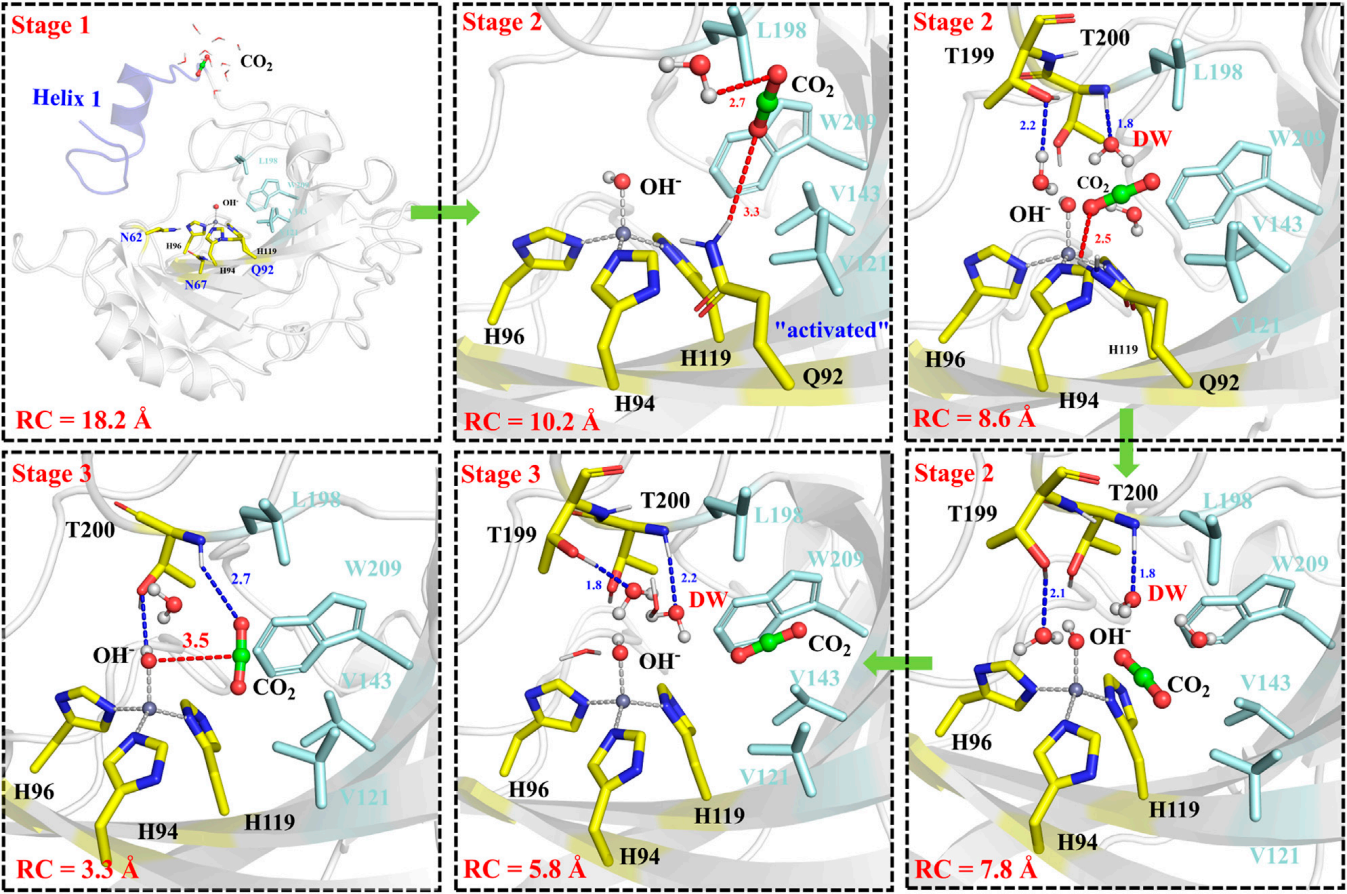
Representative structures and conformational changes for the delivery of CO_2_, where the reaction coordinate is defined as the distance between W209@CH2 and CO_2_@C.

**FIGURE 9:**
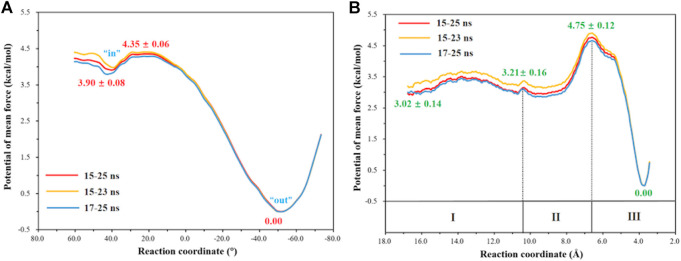
**(A)** Free energy profiles for the rotation of the protonated H64 from *inward* to *outward* conformation. **(B)** Free energy profiles for the CO_2_ delivery to the active site of CA.

The next step is CO_2_ delivery from the bulk water to the active site of CA, and here the distance between CO_2_@C and W209@CH2 was defined as the reaction coordinate (RC1) (see Figure 3A). According to the position evolution of CO_2_ and the relative free energy changes depicted in Figure 8 and Figure 9B, the whole CO_2_ transportation process can be subdivided into three stages: 1) The initial stage (RC1 ≥ 10.4 Å). CO_2_, surrounded by water molecules through the hydrogen bond interaction, is outside of the protein; 2) The second stage (6.6 Å ≤ RC1 < 10.4 Å). CO_2_ is getting into the protein gradually, while it is still out of the hydrophobic pocket, composed by V121, V143, L198 and W209. Besides that, as shown in Figure 8, during the stage I, Q92 is parallel to the *β*-sheet protein at before, however, Q92 is “activated” at this stage, and to drive CO_2_ delivery into the hydrophobic pocket through its hydrogen bond interaction with CO_2_. CO_2_ transportation during this stage needs to overcome a free energy barrier of ∼1.5 kcal/mol; 3) The third stage (10.4 Å ≤ RC1 ≤ 6.6 Å). CO_2_ is getting into the hydrophobic pocket and squeezes out the water molecule (DW), in which a hydrogen bond between CO_2_ and T200 is formed, and at the same time, Q92 is back to the initial state. There is the energy release of about −4.8 kcal/mol at this stage.

The whole process of CO_2_ delivery to the active site is exothermic by 3.0 kcal/mol with a free energy barrier of 1.7 kcal/mol, where the residue Q92 plays a crucial role in this process. Note that the number of water molecules within 3 Å of CO_2_ decreases gradually during the CO_2_ delivery, as shown in Supplementary Figure 5, although there is a short-term fluctuation during CO_2_ entry into the hydrophobic pocket. And the desolvation effect from the squeezing out of water molecules solvating the active-site protein is conducive to enhancing the binding affinity of CO_2_.

### Mechanisms for the Enzymatic Hydration of CO_2_

**FIGURE 10:**
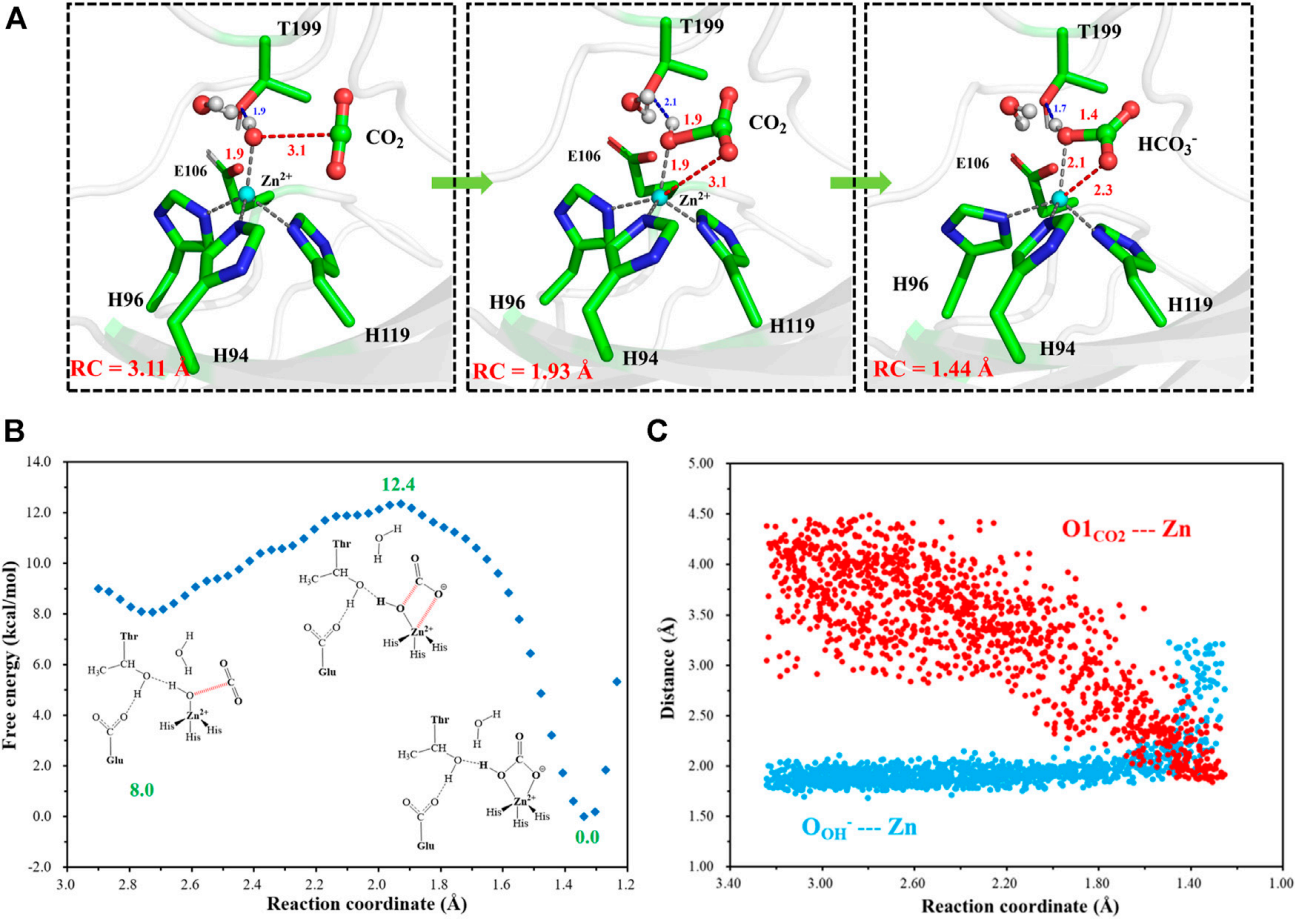
**(A)** Selected representative structures of the active site in the enzymatic hydration of CO_2_. The distance between OH^−^@O and CO_2_@C is defined as RC4. **(B)** The predicted free energy profile for the hydration of CO_2_ by metadynamics simulations at the QM(B3LYP/6-31G**)/MM level. The reaction coordinate is defined as the distance between OH^−^@O and CO_2_@C. **(C)** Evolution of selected interatomic distances along the reaction coordinate.

After another 200 ns MD simulation, the equilibrium configuration of the CA-CO_2_ complex system is obtained. The Well-Tempered Adaptively Biased Molecular Dynamics (WT-ABMD) method was used to explore the free energy profile for the hydration of CO_2_. The corresponding reaction coordinate (RC4) is defined as the distance between CO_2_@C and OH^−^@O (see Figure 10A), and the nucleophilic attack of OH^−^@O on CO_2_@C produces bicarbonate, which is coordinated to the zinc ion in a bidentate mode. As Figure 10 shows, the enzymatic CO_2_ hydration has the reaction ΔG of −12.4 kcal/mol and the free energy barrier of about 4.4 kcal/mol, suggesting that the CO_2_ hydration is quite facile, both thermodynamically and kinetically. Clearly, the notable energy release may facilitate the release of HCO_3_^−^ and the rotation of H64. The evolution of selected distances depicted in Figure 10C reveals that the distance between CO_2_@O1 and the zinc ion decreases as the RC4 decreases, while the distance between OH^−^@O and the zinc ion slightly increases to ∼ 2.1 Å, leading to a stable pentacoordinate Zn^2+^ configuration after the HCO_3_^−^ formation, where the newly-generated bicarbonate is bound to Zn^2+^ in a bidentate coordination. Besides that, as shown in Figures 10B,C, the distance between OH^−^@O and the zinc ion increases to more than 3 Å, corresponding to the configuration change from the bidentate to monodentate coordination directly, and there is a sharp rise of the free energy.

### Release of HCO_3_^−^ and Deprotonation of H64

**FIGURE 11:**
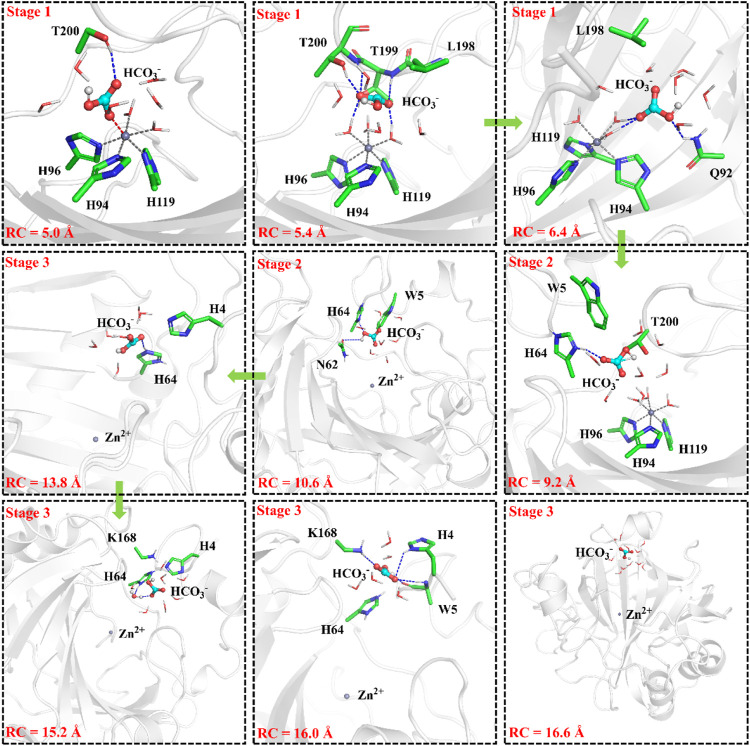
Representative structures and conformational changes of the transportation channel for the release of HCO_3_^-^, where the distance between H119@NE2 and HCO_3_^-^@C is defined as RC5.

**FIGURE 12:**
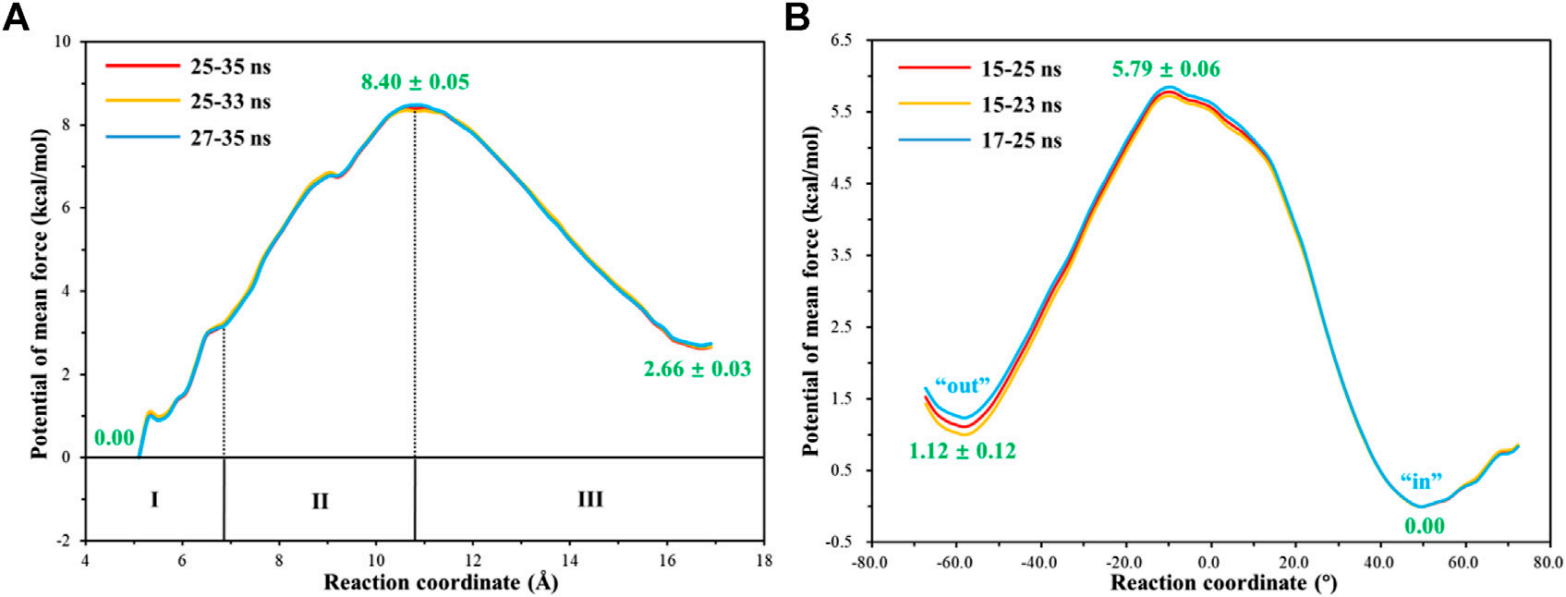
**(A)** Free energy profile for the release of HCO_3_^-^ from the active site to the bulk water. **(B)** Free energy profile for the rotation of the unprotonated H64 from *outward* to *inward* conformation.

To have an insight into the release of HCO_3_^−^, the representative snapshot from the 50 ns MM MD simulation was selected as the initial structure for subsequent investigation of the product release. The umbrella sampling and WHAM methods were applied to predict the relative free energy profile for the product release, based on the reaction coordinate (RC5) defined by the distance between H119@NE2 and HCO_3_^−^@C. According to conformational features and free energy profiles, the process of HCO_3_^−^ release could be subdivided into three stages. At the first stage, HCO_3_^−^ is initially bound to Zn^2+^ through the electrostatic interactions, and then it dissociates from the zinc coordination shell. The zinc-unbound HCO_3_^−^ has relative strong hydrogen bond interactions with the active-site residues L198, T199, T200, Q92 and surrounding water molecules, as shown in Figure 11. Importantly, Q92 as the key residue is also involved in the release of HCO_3_^−^. In the second stage, HCO_3_^−^ is pulled out of the protein with the guide of residues H64, Y5, and N62. As shown in Figure12A, there is a free energy barrier of ∼8.4 kcal/mol for the first two stages including dissociation of HCO_3_^−^ and ligand exchange around Zn^2+^. In the third stage, HCO_3_^−^ gets into the bulk water through helix 1 and builds hydrogen interactions with H4, W5, H64 and Lys168. The release process of HCO_3_^−^ requires a free energy of about 2.7 kcal/mol. Compared with the results of Markov-state model (Chen et al., 2018a; Chen et al., 2018b), as shown in Figure 9B and Figure 12A, our results also found that the diffuses of HCO_3_^-^ release is more difficult than CO_2_ delivery, besides that, we obtain free energy profile through umbrella sampling, and observed the influences of the key residues such as H64, Q92, and T200 on substrates delivery and product release.

**FIGURE 13:**
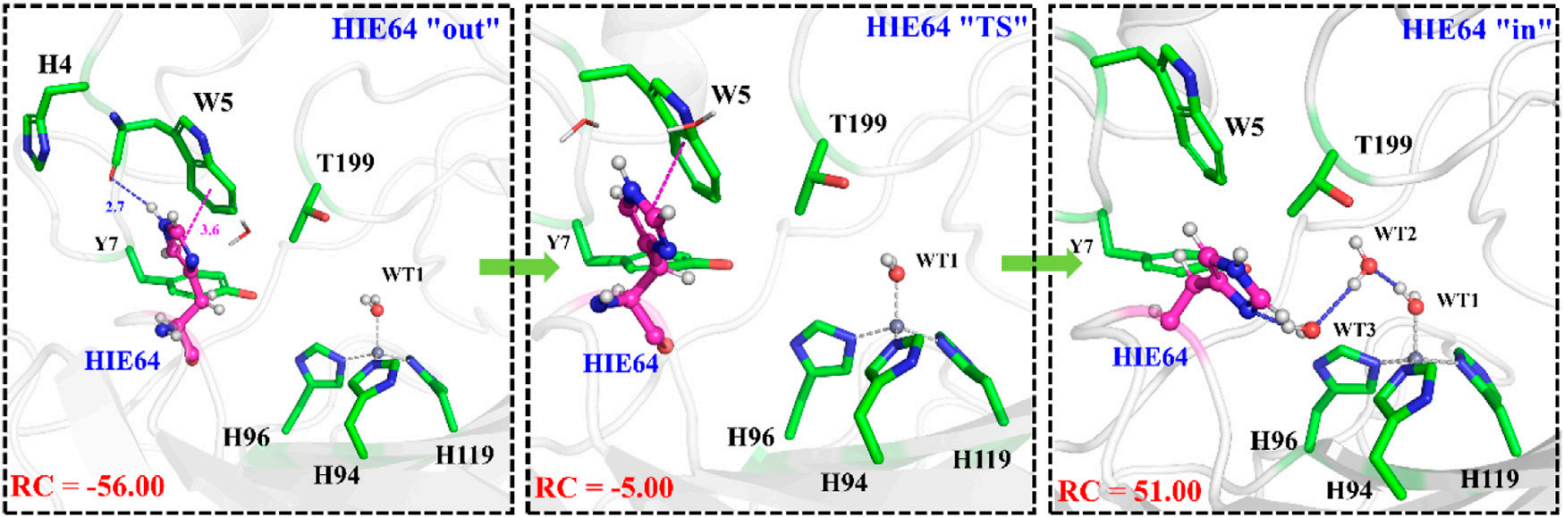
Representative structures and conformational changes of the rotation of H64 from *out*ward to *inward* conformation, where the reaction coordinate is defined as the dihedral angle χ(N–C_*α*_–C_*β*_–C_*γ*_).

**FIGURE 14:**
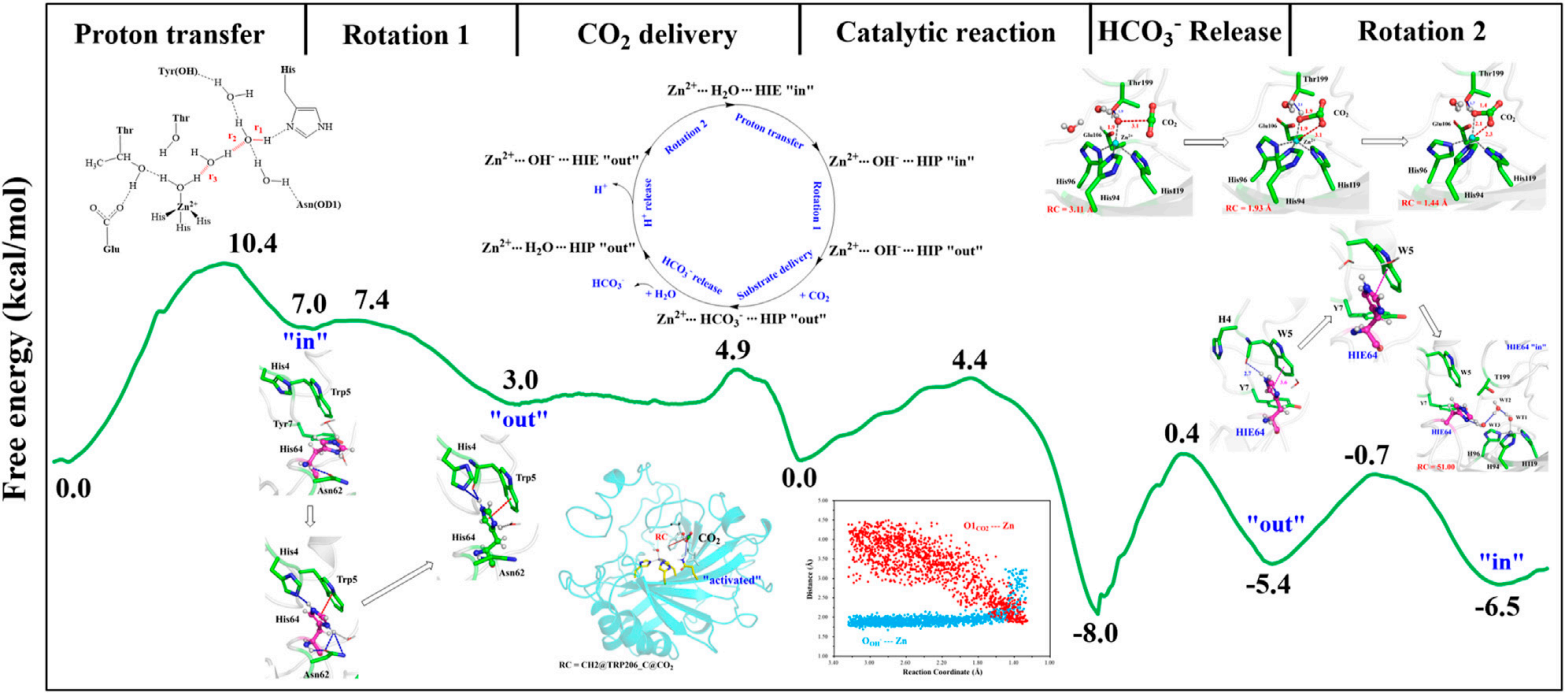
Free energy profiles for the entire enzymatic catalytic process.

With the departure of HCO_3_^−^, the protonated H64 may release one proton to the medium and rotates back to the *inward* conformation, recovering to the ready state of CA for the next cycle. Previous studies proposed that the rate-limiting step might be related to external buffers. (Tu and Silverman, 1975; Silverman et al., 1979; Lindskog et al., 1991; Lu and Voth, 1998). At high buffer concentrations, the proton transfer between the zinc-bound water and H64 is the rate-limiting step, while the proton release to the medium is the rate-limiting step at low buffer concentrations. (Tu and Silverman, 1975; Silverman et al., 1979; Lindskog et al., 1991). Here primary QM/MM and QM calculations were performed to explore the deprotonation of H64, and test calculations indicate that the proton abstractions from H64 by the nearby residue N62 and water molecule are quite difficult (see Supplemenatary Figures 6, 7). On the contrary, the proton transfer from H64 to a phosphate group with the aid of one water molecule is predicted to be barrier free by QM calculations with a cluster model, and the full geometry optimization directly converges to the proton-transfer product with the energy release of 112 kcal/mol, suggesting that the deprotonation of H64 by the phosphate group, which approximately mimics the presence of external buffers as the proton acceptor or donor, is quite facile. As Figure 13 shows, the hydrogen-bond interaction between H4 and H64 disappears after the deprotonation of H64, while W5 still maintains the hydrogen bond and π-π stacking interactions with H64 in the *outward* conformation. Afterward, H64 recovers to the *inward* conformation through the narrow channel between W5 and N62, and the water chain of two molecules connecting H64 to Zn^2+^ is again formed, as observed in the initial configuration of CA. The narrow channel for the rotation of H64 was proposed in previous transition path sampling study. (Roy and Taraphder, 2009). As Figure 12B shows, the H64 rotation back to the *inward* conformation along with its deprotonation experiences about a free energy barrier of 4.7 kcal/mol with the free energy release of about 1.1 kcal/mol, leading to that CA evolves to its initial state and completion of the enzymatic cycle for CO_2_ hydration. The free energy profiles for the whole catalytic hydration process of CO_2_ are summarized in Figure 14, and this enzymatic capture of CO_2_ is favorable, both thermodynamically and kinetically.

## Conclusion

Extensive QM(B3LYP)/MM MD and MM MD simulations with the umbrella sampling have been used to study the whole enzymatic hydration of CO_2_ by carbonic anhydrase, including the CO_2_ delivery to the active site, the catalytic mechanisms for proton transfer and CO_2_ hydration, the dissociation and release of HCO_3_^−^, the conformational change of H64 side chain, and the role of key residues. The present results reveal that Q92 plays role in CO_2_ transportation and the release of HCO_3_^−^. Besides that, H4, N62 and W5 also show nonnegligible effects, as they stabilize the protonated H64 and provide the driving force for the rotation of H64 and the release of HCO_3_^−^. The proton transfer from the water molecule (WT1) to H64, leading to formation of the Zn-bound OH^−^, is the rate-limiting step with an energy barrier of about 10.4 kcal/mol, in line with previous experiments and calculations. The conformational transition of the protonated H64 from *inward* to *outward* mediates CO_2_ entry into the active site, which squeezes out the water molecules from the hydrated active-site with an energy release of 7.0 kcal/mol. After the entrance of CO_2_ into the hydrophobic pocket, the nucleophilic attack of OH^−^@O on CO_2_@C forms HCO_3_^−^, which is bound to the zinc ion in the bidentate coordination, with a free energy span of 4.4 kcal/mol and the free energy release of 8.0 kcal/mol. The release of HCO_3_^−^ from the active site has the free-energy span of 8.4 kcal/mol. During the release of HCO_3_^−^, H64 may be deprotonated with the aid of H4 and K168 and then rotates back from *outward* to *inward* conformation with the free energy barrier of 4.7 kcal/mol. The present study provides a comprehensive understanding of enzymatic hydration CO_2_ by carbonic anhydrase, which is important for rational enzyme engineering of biological capture and sequestration of CO_2_.

## Data Availability

The original contributions presented in the study are included in the article/Supplementary Material, further inquiries can be directed to the corresponding authors.
