# Supplementary material for: Conformational Change of H64 and Substrate Transportation: Insight Into a Full Picture of Enzymatic Hydration of CO2 by Carbonic Anhydrase
Source: Front Chem. 2021 Jul 9;9:706959. doi: 10.3389/fchem.2021.706959 (PMC8299336; doi:10.3389/fchem.2021.706959)
Supplement: Supplementary file 1 [file DataSheet2.docx]

RC_PT.xyz

15368

N 55.987999 44.729000 30.986000

H1 56.198002 44.820999 31.975000

H2 56.813999 45.055000 30.496000

H3 55.789001 43.747002 30.841999

CA 54.796001 45.548000 30.648001

HA 55.113998 46.543999 30.947001

CB 54.374001 45.537998 29.146000

HB2 53.425999 46.066002 29.034000

HB3 55.108002 46.055000 28.534000

CG 54.169998 44.179001 28.521999

ND1 55.178001 43.408001 27.934000

CE1 54.625999 42.199001 27.789000

HE1 55.229000 41.358002 27.497999

NE2 53.388000 42.152000 28.292999

HE2 52.939999 41.277000 28.556999

CD2 53.055000 43.405998 28.714001

HD2 52.134998 43.657001 29.225000

C 53.585999 45.144001 31.493999

O 53.514000 43.991001 31.920000

N 52.618999 46.035000 31.723000

H 52.696999 46.965000 31.316999

CA 51.396000 45.675999 32.459000

HA 51.692001 45.084999 33.325001

CB 50.674000 46.924000 32.973999

HB2 50.362999 47.542000 32.130001

HB3 49.770000 46.605000 33.498001

CG 51.502998 47.768002 33.911999

ND1 51.321999 49.111000 34.132999

HD1 50.606998 49.694000 33.701000

CE1 52.238998 49.514000 35.023998

HE1 52.346001 50.527000 35.377998

NE2 52.997002 48.478001 35.423000

CD2 52.535000 47.362000 34.722000

HD2 52.935001 46.361000 34.806000

C 50.438999 44.796001 31.648001

O 50.258999 44.993999 30.444000

N 49.816002 43.818001 32.313999

H 49.966000 43.748001 33.313000

CA 48.943001 42.824001 31.674000

HA 49.457001 42.459000 30.782000

CB 48.757999 41.610001 32.599998

HB2 48.424999 40.775002 31.983000

HB3 49.729000 41.324001 33.007999

CG 47.775002 41.740002 33.728001

CD1 48.073002 42.039001 35.014000

HD1 49.070000 42.227001 35.395000

NE1 46.916000 42.044998 35.770000

HE1 46.888000 42.194000 36.776001

CE2 45.811001 41.742001 35.009998

CZ2 44.451000 41.610001 35.319000

HZ2 44.108002 41.768002 36.331001

CH2 43.550999 41.248001 34.303001

HH2 42.499001 41.125999 34.522999

CZ3 44.023998 41.023998 32.999001

HZ3 43.334000 40.726002 32.220001

CE3 45.394001 41.160999 32.702000

HE3 45.740002 40.953999 31.700001

CD2 46.326000 41.534000 33.695999

C 47.601002 43.423000 31.214001

O 47.102001 44.389999 31.802999

N 47.000999 42.845001 30.172001

H 47.465000 42.075001 29.698000

CA 45.717999 43.287998 29.624001

HA2 44.992001 43.410999 30.426001

HA3 45.858002 44.255001 29.143000

C 45.143002 42.298000 28.614000

O 45.342999 41.091999 28.752001

N 44.426998 42.807999 27.608999

H 44.238998 43.805000 27.639999

CA 43.817001 42.028999 26.520000

HA 44.200001 41.012001 26.572001

CB 42.289001 41.929001 26.726999

HB2 41.894001 42.952999 26.629999

HB3 41.855999 41.342999 25.903000

CG 41.865002 41.316002 28.052000

CD1 41.532001 39.953999 28.155001

HD1 41.597000 39.319000 27.267000

CE1 41.083000 39.388000 29.353001

HE1 40.819000 38.330002 29.410999

CZ 40.936001 40.187000 30.499001

OH 40.455002 39.713001 31.676001

HH 40.303001 38.714001 31.686001

CE2 41.283001 41.546001 30.419001

HE2 41.202000 42.167000 31.313999

CD2 41.734001 42.091999 29.216999

HD2 41.999001 43.152000 29.191000

C 44.217999 42.535999 25.108999

O 43.592999 42.155998 24.111000

N 45.255001 43.383999 25.014000

H 45.771999 43.589001 25.862000

CA 45.789001 43.962002 23.773001

HA2 44.969002 44.160000 23.080999

HA3 46.263000 44.917000 24.011999

C 46.821999 43.069000 23.066999

O 47.126999 41.955002 23.502001

N 47.382999 43.553001 21.954000

H 47.118000 44.486000 21.662001

CA 48.297001 42.785999 21.077000

HA 47.881001 41.786999 20.919001

CB 48.383999 43.498001 19.716999

HB2 48.841000 44.477001 19.867001

HB3 49.021999 42.903000 19.063999

CG 47.028999 43.691002 19.018000

HG2 46.570000 42.721001 18.833000

HG3 46.366001 44.278000 19.655001

CD 47.202000 44.455002 17.698000

HD2 47.582001 45.452000 17.930000

HD3 47.925999 43.957001 17.052000

CE 45.862999 44.599998 16.966999

HE2 45.085999 44.845001 17.698999

HE3 45.943001 45.433998 16.266001

NZ 45.514000 43.372002 16.212999

HZ1 44.581001 43.426998 15.821000

HZ2 46.188000 43.195000 15.474000

HZ3 45.507999 42.544998 16.808001

C 49.707001 42.577999 21.665001

O 50.445000 41.709999 21.208000

N 50.084000 43.372002 22.662001

H 49.448002 44.120998 22.908001

CA 51.372002 43.360001 23.379999

HA 52.139000 42.863998 22.787001

CB 51.800999 44.828999 23.556999

HB2 52.766998 44.868999 24.062000

HB3 51.914001 45.304001 22.580999

CG 50.821999 45.647999 24.350000

ND1 49.451000 45.764999 24.089001

CE1 48.966999 46.560001 25.049999

HE1 47.924999 46.827000 25.159000

NE2 49.952000 46.928001 25.884001

HE2 49.825001 47.477001 26.732000

CD2 51.130001 46.362999 25.462999

HD2 52.105999 46.450001 25.921000

C 51.289001 42.625999 24.736000

O 52.277000 42.030998 25.176001

N 50.113998 42.644001 25.385000

H 49.355000 43.152000 24.952999

CA 49.917999 42.273998 26.795000

HA 50.817001 41.769001 27.150000

CB 49.758999 43.567001 27.613001

HB2 49.849998 43.299000 28.660999

HB3 50.570000 44.250999 27.365000

CG 48.431000 44.301998 27.452000

OD1 47.562000 43.953999 26.665001

ND2 48.221001 45.337002 28.226000

HD21 47.356998 45.848999 28.122000

HD22 48.937000 45.626999 28.882999

C 48.766998 41.284000 27.058001

O 48.358002 41.106998 28.209000

N 48.213001 40.695000 26.000000

H 48.544998 40.966000 25.084999

CA 46.998001 39.886002 26.072001

HA2 46.287998 40.438000 26.672001

HA3 46.583000 39.790001 25.072001

C 47.160999 38.481998 26.687000

O 48.285000 38.068001 26.986000

N 46.058998 37.710999 26.826000

CD 44.681999 38.179001 26.655001

HD2 44.487999 38.417000 25.608999

HD3 44.486000 39.044998 27.285000

CG 43.768002 37.042999 27.106001

HG2 42.872002 36.972000 26.489000

HG3 43.488998 37.182999 28.149000

CB 44.657001 35.812000 26.965000

HB2 44.632000 35.476002 25.926001

HB3 44.349998 35.012001 27.634001

CA 46.058998 36.319000 27.309000

HA 46.174999 36.324001 28.392000

C 47.132000 35.381001 26.718000

O 47.641998 34.509998 27.424000

N 47.528999 35.598999 25.459999

H 47.064999 36.344002 24.957001

CA 48.632999 34.923000 24.756001

HA 48.414001 33.860001 24.665001

CB 48.657001 35.527000 23.333000

HB2 47.754002 35.195999 22.816999

HB3 48.609001 36.615002 23.414000

CG 49.859001 35.202000 22.438000

HG2 49.646000 35.570999 21.434999

HG3 50.721001 35.744999 22.820000

CD 50.221001 33.720001 22.351000

OE1 51.430000 33.407001 22.323999

OE2 49.335999 32.834999 22.268999

C 50.000999 35.042999 25.473000

O 50.866001 34.180000 25.299999

N 50.198002 36.082001 26.295000

H 49.407001 36.696999 26.454000

CA 51.506001 36.536999 26.795000

HA 52.270000 35.838001 26.448999

CB 51.835999 37.901001 26.163000

HB2 51.008999 38.590000 26.344999

HB3 52.720001 38.319000 26.642000

CG 52.113998 37.838001 24.683001

ND1 53.174000 37.145000 24.083000

CE1 53.028999 37.360001 22.761999

HE1 53.660000 36.933998 21.993999

NE2 51.973999 38.157001 22.518000

HE2 51.627998 38.419998 21.599001

CD2 51.391998 38.477001 23.721001

HD2 50.521000 39.098000 23.879000

C 51.661999 36.615002 28.326000

O 52.791000 36.828999 28.778999

N 50.603001 36.448002 29.125000

H 49.702000 36.271999 28.701000

CA 50.674999 36.540001 30.596001

HA 51.009998 37.542000 30.868000

CB 49.278999 36.319000 31.202000

HB2 48.966000 35.298000 30.983000

HB3 49.370998 36.395000 32.286999

CG 48.158001 37.236000 30.792999

CD1 48.262001 38.436001 30.171000

HD1 49.186001 38.905998 29.853001

NE1 47.001999 38.972000 29.975000

HE1 46.831001 39.828999 29.450001

CE2 46.018002 38.164001 30.496000

CZ2 44.620998 38.268002 30.552000

HZ2 44.125000 39.134998 30.139000

CH2 43.881001 37.230999 31.143999

HH2 42.803001 37.294998 31.195999

CZ3 44.547001 36.103001 31.657000

HZ3 43.974998 35.304001 32.104000

CE3 45.950001 36.007000 31.590000

HE3 46.445999 35.126999 31.968000

CD2 46.724998 37.039001 31.017000

C 51.674000 35.541000 31.216999

O 52.313000 35.844002 32.229000

N 51.881001 34.374001 30.594000

H 51.262001 34.148998 29.818001

CA 52.859001 33.363998 31.037001

HA 52.563999 33.030998 32.033001

CB 52.826000 32.138000 30.114000

HB2 53.448002 31.364000 30.566000

HB3 51.805000 31.763000 30.052999

CG 53.341999 32.388000 28.719000

ND1 54.688999 32.511002 28.350000

CE1 54.674000 32.780998 27.032000

HE1 55.551998 32.963001 26.426001

NE2 53.412998 32.846001 26.577000

HE2 53.143002 33.103001 25.629000

CD2 52.560001 32.591000 27.622999

HD2 51.478001 32.569000 27.591999

C 54.299000 33.891998 31.146000

O 55.043999 33.410999 32.004002

N 54.686001 34.912998 30.363001

H 53.969002 35.318001 29.761000

CA 55.980000 35.636002 30.554001

HA 56.814999 34.937000 30.480000

CB 56.193001 36.639000 29.434999

HB2 55.460999 37.432999 29.584000

HB3 57.176998 37.088001 29.584000

CG 56.090000 36.002998 28.025000

HG2 56.726002 35.129002 27.877001

HG3 55.085999 35.632000 27.841000

CD 56.419998 36.991001 26.900999

HD2 55.818001 37.889999 26.975000

HD3 57.456001 37.327000 26.910999

CE 56.191002 36.362000 25.542000

HE2 56.625999 35.360001 25.568001

HE3 55.129002 36.235001 25.305000

NZ 56.916000 37.183998 24.537001

HZ1 57.915001 37.181000 24.733999

HZ2 56.848000 36.756001 23.629999

HZ3 56.631001 38.154999 24.447001

C 56.154999 36.441002 31.879999

O 57.189999 37.089001 32.099998

N 55.145000 36.487999 32.742001

H 54.252998 36.115002 32.453999

CA 55.221001 37.166000 34.051998

HA 56.279999 37.185001 34.300999

CB 54.696999 38.624001 33.875999

HB2 54.209000 38.731998 32.897999

HB3 53.948002 38.800999 34.638000

CG 55.716000 39.735001 34.141998

OD1 56.723000 39.498001 34.853001

OD2 55.464001 40.877998 33.709000

C 54.513000 36.391998 35.220001

O 54.773998 36.591000 36.419998

N 53.667000 35.432999 34.832001

H 53.432999 35.391998 33.849998

CA 52.965000 34.491001 35.702000

HA 53.367001 34.566002 36.709999

CB 51.479000 34.897999 35.758999

HB2 51.041000 34.775002 34.766998

HB3 50.955002 34.223000 36.438999

CG 51.249001 36.331001 36.208000

CD1 51.138000 36.633999 37.577999

HD1 51.202000 35.845001 38.314999

CE1 50.990002 37.970001 37.994999

HE1 50.903000 38.201000 39.048000

CZ 50.977001 39.009998 37.048000

HZ 50.915001 40.040001 37.374001

CE2 51.063999 38.709000 35.675999

HE2 51.035999 39.507000 34.944000

CD2 51.203999 37.374001 35.257999

HD2 51.331001 37.158001 34.206001

C 53.188999 33.035000 35.234001

O 52.254002 32.410999 34.722000

N 54.408001 32.460999 35.382999

CD 55.653999 33.151001 35.785000

HD2 55.726002 33.266998 36.866001

HD3 55.799000 34.126999 35.320000

CG 56.789001 32.222000 35.380001

HG2 57.623001 32.287998 36.080002

HG3 57.078999 32.391998 34.341999

CB 56.125000 30.860001 35.484001

HB2 55.987999 30.580000 36.528000

HB3 56.646000 30.096001 34.905998

CA 54.734001 31.108000 34.922001

HA 54.823002 31.086000 33.834999

C 53.778000 29.995001 35.388000

O 53.708000 28.952999 34.748001

N 53.033001 30.197001 36.488998

H 53.154999 31.065001 37.005001

CA 52.028999 29.236000 36.977001

HA 52.563000 28.302000 37.124001

CB 51.492001 29.645000 38.367001

HB 52.348999 29.929001 38.980000

CG2 50.542999 30.853001 38.286999

HG21 49.595001 30.559999 37.832001

HG22 50.345001 31.240999 39.284000

HG23 50.993999 31.649000 37.695000

CG1 50.799999 28.444000 39.055000

HG12 49.783001 28.323999 38.674999

HG13 51.353001 27.528000 38.834000

CD1 50.759998 28.601999 40.577000

HD11 50.317001 27.714001 41.028000

HD12 51.771999 28.719999 40.959999

HD13 50.165001 29.473000 40.846001

C 50.912998 28.945000 35.946999

O 50.264999 27.900000 36.025002

N 50.769001 29.791000 34.914001

H 51.316002 30.645000 34.930000

CA 49.971001 29.528999 33.709000

HA 48.944000 29.349001 34.018002

CB 49.977001 30.773001 32.803001

HB1 50.037998 31.683001 33.395000

HB2 50.821999 30.750000 32.116001

HB3 49.056999 30.798000 32.216000

C 50.431000 28.289000 32.914001

O 49.709000 27.851000 32.020000

N 51.609001 27.716000 33.199001

H 52.222000 28.156000 33.886002

CA 52.112999 26.473000 32.591999

HA 51.537998 26.202999 31.702000

CB 53.525002 26.813999 32.131001

HB2 54.030998 27.339001 32.933998

HB3 54.137001 25.923000 32.033001

CG 53.512001 27.698999 30.864000

HG2 52.958000 27.259001 30.027000

HG3 53.122002 28.690001 31.118999

CD 54.992001 27.771999 30.469999

HD2 55.555000 28.222000 31.287001

HD3 55.325001 26.750000 30.294001

CE 55.327000 28.548000 29.212000

HE2 54.543999 28.414000 28.455999

HE3 55.414001 29.601999 29.429001

NZ 56.657001 28.191999 28.670000

HZ1 56.834999 28.746000 27.839001

HZ2 57.433998 28.184999 29.320000

HZ3 56.606998 27.257999 28.299000

C 51.987000 25.235001 33.504002

O 52.435001 24.153000 33.153999

N 51.301998 25.368999 34.632000

H 50.898998 26.278000 34.771000

CA 50.688999 24.252001 35.344002

HA2 51.389999 23.434000 35.466000

HA3 50.379002 24.531000 36.348999

C 49.542000 23.608000 34.599998

O 49.094002 24.051001 33.541000

N 49.160000 22.448999 35.125000

H 49.618000 22.224001 36.007000

CA 48.113998 21.611000 34.499001

HA 48.296001 21.580999 33.422001

CB 48.202999 20.139000 34.937000

HB2 47.804001 20.003000 35.936001

HB3 47.616001 19.547001 34.237000

CG 49.542999 19.458000 35.036999

HG2 49.981998 19.718000 35.993999

HG3 49.366001 18.389000 35.068001

CD 50.466000 19.687000 33.859001

OE1 51.674999 20.002001 34.028999

OE2 49.966000 19.509001 32.730000

C 46.666000 22.106001 34.758999

O 45.730999 21.620001 34.119999

N 46.438999 22.980000 35.752998

H 47.248001 23.290001 36.286999

CA 45.098999 23.226999 36.338001

HA 44.414001 22.497000 35.909000

CB 45.118999 22.957001 37.853001

HB2 45.862999 23.601000 38.325001

HB3 44.140999 23.197001 38.273998

CG 45.400002 21.472000 38.141998

HG2 44.723000 20.871000 37.532001

HG3 46.419998 21.216000 37.865002

CD 45.161999 21.072001 39.597000

HD2 44.117001 21.264000 39.851002

HD3 45.348999 20.000000 39.674999

NE 46.028000 21.775000 40.556000

HE 46.754002 22.379999 40.199001

CZ 45.879002 21.740999 41.866001

NH1 44.972000 21.018999 42.449001

HH11 44.436001 20.360001 41.889999

HH12 44.801998 21.055000 43.442001

NH2 46.645000 22.466000 42.611000

HH21 47.257999 23.125000 42.147999

HH22 46.402000 22.636000 43.582001

C 44.466000 24.586000 36.000000

O 43.634998 25.087000 36.754002

N 44.823002 25.195000 34.873001

H 45.463001 24.724001 34.247002

CA 44.299000 26.511000 34.480999

HA 44.346001 27.153999 35.355999

CB 45.213001 27.125000 33.410999

HB2 45.306000 26.424000 32.584999

HB3 44.769001 28.035999 33.018002

CG 46.611000 27.468000 33.942001

HG2 47.044998 26.604000 34.443001

HG3 47.250999 27.705000 33.095001

CD 46.589001 28.672001 34.879002

OE1 46.256001 29.780001 34.474998

NE2 46.942001 28.504999 36.132999

HE21 46.898998 29.288000 36.771000

HE22 47.285999 27.601000 36.452000

C 42.818001 26.496000 34.037998

O 42.286999 25.457001 33.629002

N 42.175999 27.664000 34.112999

H 42.688000 28.427000 34.542999

CA 40.771999 27.959000 33.766998

HA 40.332001 27.113001 33.240002

CB 39.991001 28.219000 35.056999

HB2 40.518002 28.965000 35.653000

HB3 38.995998 28.603001 34.827000

OG 39.866001 27.021999 35.793999

HG 39.011002 26.641001 35.533001

C 40.675999 29.215000 32.884998

O 41.542999 30.083000 33.012001

N 39.648998 29.396000 32.028000

CD 39.632999 30.517000 31.097000

HD2 40.566002 30.577000 30.538000

HD3 39.453999 31.443001 31.643999

CG 38.484001 30.254000 30.139000

HG2 38.820000 29.622000 29.320999

HG3 38.073002 31.179001 29.749001

CB 37.508999 29.487000 31.025999

HB2 36.778000 28.934999 30.437000

HB3 37.006001 30.177999 31.704000

CA 38.451000 28.569000 31.818001

HA 37.984001 28.311001 32.764999

C 38.737000 27.287001 31.014999

O 39.840000 27.118000 30.500000

N 37.737000 26.416000 30.837999

H 36.838001 26.618000 31.253000

CA 37.785999 25.283001 29.891001

HA 38.625000 25.431999 29.205999

CB 38.023998 23.934999 30.599001

HB 38.148998 23.184000 29.820999

CG1 39.318001 23.941000 31.421000

HG11 40.151001 24.254999 30.794001

HG12 39.229000 24.625000 32.263000

HG13 39.515999 22.937000 31.790001

CG2 36.844002 23.480000 31.474001

HG21 36.528000 24.275999 32.143002

HG22 36.000000 23.191999 30.846001

HG23 37.138000 22.614000 32.067001

C 36.507999 25.204000 29.047001

O 35.487999 25.799999 29.391001

N 36.544998 24.447001 27.955999

H 37.433998 24.032000 27.688000

CA 35.375000 24.097000 27.150999

HA 34.743000 24.982000 27.070999

CB 35.867001 23.705999 25.747999

HB2 36.637001 24.417999 25.447001

HB3 36.313000 22.709999 25.778000

CG 34.786999 23.743999 24.673000

OD1 33.651001 24.184999 24.944000

OD2 35.098000 23.427999 23.504000

C 34.556999 22.969999 27.811001

O 35.084999 21.896000 28.114000

N 33.261002 23.197001 28.042999

H 32.877998 24.094000 27.761999

CA 32.359001 22.259001 28.729000

HA 32.977001 21.646000 29.386000

CB 31.361000 22.995001 29.655001

HB 30.702999 23.608999 29.040001

CG2 30.518000 21.974001 30.450001

HG21 29.725000 22.485001 30.990999

HG22 30.039000 21.252001 29.795000

HG23 31.146000 21.437000 31.159000

CG1 32.126999 23.903999 30.653999

HG12 32.766998 23.278999 31.280001

HG13 32.770000 24.589001 30.101999

CD1 31.253000 24.785000 31.559000

HD11 31.889000 25.271000 32.298000

HD12 30.766001 25.548000 30.952000

HD13 30.499001 24.205000 32.089001

C 31.738001 21.305000 27.695000

O 30.561001 21.360001 27.337999

N 32.617001 20.450001 27.181999

H 33.541000 20.600000 27.563000

CA 32.395000 19.421000 26.146000

HA 32.022999 19.886000 25.228001

CB 33.734001 18.726000 25.830000

HB2 34.380001 19.341999 25.215000

HB3 34.257000 18.597000 26.771000

CG 33.644001 17.400999 25.087000

OD1 34.512001 16.535000 25.329000

OD2 32.780998 17.311001 24.174999

C 31.421000 18.360001 26.711000

O 31.771000 17.677999 27.684999

N 30.249001 18.191000 26.079000

H 29.997000 18.882999 25.386000

CA 29.216999 17.216999 26.503000

HA 29.051001 17.333000 27.573999

CB 27.829000 17.500999 25.846001

HB 27.808001 17.086000 24.844000

CG2 26.556999 17.004999 26.558001

HG21 26.465000 15.944000 26.374001

HG22 26.541000 17.091000 27.643999

HG23 25.677999 17.518999 26.157000

OG1 27.622000 18.874001 25.719999

HG1 27.559000 19.172001 26.639999

C 29.695000 15.758000 26.365000

O 29.032000 14.920000 26.969000

N 30.851999 15.439000 25.753000

H 31.405001 16.176001 25.320000

CA 31.424999 14.075000 25.872000

HA 30.628000 13.335000 25.868999

CB 32.291000 13.702000 24.669001

HB2 31.638000 13.869000 23.816000

HB3 33.183998 14.327000 24.639000

CG 32.766998 12.258000 24.669001

ND1 31.933001 11.150000 24.618000

CE1 32.749001 10.086000 24.506001

HE1 32.442001 9.070000 24.297001

NE2 34.035000 10.471000 24.556000

HE2 34.830002 9.837000 24.485001

CD2 34.070000 11.846000 24.653999

HD2 34.919998 12.515000 24.587999

C 32.243000 13.870000 27.153000

O 32.264999 12.807000 27.750000

N 32.945000 14.914000 27.579000

H 32.752998 15.787000 27.120001

CA 33.803001 14.985000 28.764999

HA 34.368999 14.059000 28.865999

CB 34.867001 16.069000 28.566000

HB 34.452000 17.021999 28.247000

CG2 35.672001 16.313999 29.833000

HG21 36.125999 15.388000 30.197001

HG22 36.418999 17.070000 29.603001

HG23 35.032001 16.743000 30.598000

OG1 35.737999 15.661000 27.575001

HG1 35.217999 15.839000 26.768999

C 32.952999 15.197000 30.024000

O 33.095001 14.453000 30.993999

N 32.020000 16.169001 29.983000

H 31.943001 16.725000 29.136000

CA 31.164000 16.589001 31.087999

HA 31.830000 16.570000 31.934000

CB 30.836000 18.104000 30.891001

HB1 30.055000 18.480000 31.555000

HB2 31.721001 18.723000 31.056999

HB3 30.573000 18.275000 29.851999

C 30.033001 15.581000 31.485001

O 28.858999 15.966000 31.561001

N 30.382000 14.326000 31.827000

H 31.379999 14.129000 31.829000

CA 29.396999 13.292000 32.222000

HA 28.534000 13.370000 31.556999

CB 29.943001 11.830000 32.115002

HB2 30.763000 11.648000 32.811001

HB3 29.146999 11.110000 32.331001

CG 30.426001 11.559000 30.690001

HG2 29.599001 11.901000 30.069000

HG3 31.340000 12.132000 30.551001

CD 30.659000 10.092000 30.341000

HD2 31.506001 9.646000 30.857000

HD3 29.754999 9.539000 30.598000

CE 30.962999 9.963000 28.851999

HE2 30.716000 8.938000 28.589001

HE3 30.429001 10.706000 28.252001

NZ 32.380001 10.185000 28.549000

HZ1 33.004002 9.443000 28.826000

HZ2 32.554001 10.288000 27.563000

HZ3 32.716999 11.014000 29.028000

C 28.891001 13.571000 33.624001

O 29.684999 13.874000 34.513000

N 27.589001 13.432000 33.884998

H 26.986000 13.041000 33.169998

CA 27.049000 13.636000 35.243000

HA 27.447001 14.602000 35.557999

CB 25.506001 13.742000 35.181000

HB2 25.143000 14.648000 35.667000

HB3 25.239000 13.901000 34.139999

CG 24.730000 12.546000 35.724998

CD1 24.159000 12.569000 37.013000

HD1 24.297001 13.455000 37.618000

CE1 23.631001 11.381000 37.571999

HE1 23.452000 11.344000 38.627998

CZ 23.582001 10.187000 36.834000

OH 23.122999 9.020000 37.351002

HH 22.733000 9.121000 38.247002

CE2 24.191999 10.171000 35.568001

HE2 24.287001 9.240000 35.039001

CD2 24.768999 11.333000 35.023998

HD2 25.312000 11.282000 34.094002

C 27.539000 12.558000 36.266998

O 28.014000 11.465000 35.932999

N 27.382000 12.842000 37.560001

H 27.113001 13.792000 37.745998

CA 27.740999 12.017000 38.735001

HA 27.783001 10.969000 38.432999

CB 29.174999 12.397000 39.169998

HB2 29.813999 12.379000 38.297001

HB3 29.197001 13.420000 39.553001

CG 29.864000 11.429000 40.131001

OD1 31.018999 11.048000 39.816002

OD2 29.305000 11.063000 41.195999

C 26.749001 12.142000 39.950001

O 26.287001 13.240000 40.306999

N 26.360001 11.006000 40.570999

CD 26.555000 9.647000 40.062000

HD2 27.555000 9.236000 40.219002

HD3 26.353001 9.602000 39.004002

CG 25.537001 8.736000 40.727001

HG2 25.978001 7.746000 40.840000

HG3 24.561001 8.725000 40.233002

CB 25.426001 9.445000 42.053001

HB2 26.320000 9.195000 42.622002

HB3 24.579000 9.103000 42.634998

CA 25.438000 10.945000 41.727001

HA 24.440001 11.326000 41.499001

C 25.921000 11.587000 43.014000

O 25.181999 11.815000 43.987000

N 27.233999 11.741000 43.042999

H 27.722000 11.275000 42.278999

CA 27.893000 12.406000 44.165001

HA 27.351000 12.128000 45.051998

CB 29.287001 11.913000 44.469002

HB2 29.650000 12.397000 45.381001

HB3 29.261000 10.835000 44.615002

OG 30.106001 12.222000 43.372002

HG 29.728001 11.691000 42.641998

C 27.823000 13.919000 44.153000

O 28.018999 14.515000 45.210999

N 27.457001 14.543000 43.021999

H 27.045000 13.976000 42.287998

CA 27.422001 16.011000 42.890999

HA 28.099001 16.413000 43.637001

CB 27.959000 16.570000 41.529999

HB2 27.504000 16.046000 40.680000

HB3 27.709000 17.635000 41.497002

CG 29.490000 16.500999 41.368000

HG 29.841000 15.492000 41.555000

CD1 29.780001 16.882999 39.903999

HD11 30.833000 16.832001 39.611000

HD12 29.226999 16.226000 39.236000

HD13 29.377001 17.879999 39.755001

CD2 30.330000 17.325001 42.348000

HD21 30.075001 18.388000 42.404999

HD22 30.246000 16.805000 43.299999

HD23 31.384001 17.231001 42.094002

C 25.996000 16.509001 43.243999

O 25.253000 16.815001 42.324001

N 25.558001 16.580000 44.522999

H 26.268000 16.542999 45.234001

CA 24.162001 16.874001 44.987000

HA 23.457001 16.236000 44.448002

CB 24.031000 16.586000 46.522999

HB2 24.697001 17.219999 47.098999

HB3 23.025999 16.839001 46.854000

CG 24.330999 15.168000 47.005001

HG2 25.393999 15.007000 46.801998

HG3 24.179001 15.131000 48.091999

CD 23.350000 14.205000 46.278999

HD2 22.309999 14.504000 46.458000

HD3 23.518999 14.208000 45.205002

CE 23.566000 12.759000 46.681999

HE2 22.761000 12.157000 46.241001

HE3 24.546000 12.480000 46.285000

NZ 23.590000 12.626000 48.152000

HZ1 23.533001 11.647000 48.401001

HZ2 24.443001 13.038000 48.514000

HZ3 22.791000 13.082000 48.585999

C 23.732000 18.341000 44.660999

O 24.587000 19.115000 44.228001

N 22.488001 18.823000 44.903000

CD 21.275000 18.098000 45.215000

HD2 21.093000 18.093000 46.293999

HD3 21.389000 17.080000 44.863998

CG 20.174999 18.851999 44.442001

HG2 19.155001 18.632999 44.762001

HG3 20.271000 18.660999 43.375000

CB 20.599001 20.292000 44.737999

HB2 20.315001 20.572001 45.758999

HB3 20.204000 20.989000 44.002998

CA 22.118000 20.204000 44.616001

HA 22.483000 20.464001 43.620998

C 22.777000 21.166000 45.568001

O 23.287001 20.789000 46.630001

N 22.841000 22.433001 45.160999

H 22.507999 22.642000 44.227001

CA 23.427999 23.540001 45.931999

HA 24.268000 23.139000 46.498001

CB 23.864000 24.698000 44.978001

HB2 22.955999 25.188999 44.620998

HB3 24.410000 25.415001 45.588001

CG 24.677999 24.375000 43.714001

HG 24.030001 23.761999 43.089001

CD1 25.075001 25.672001 42.990002

HD11 25.441000 25.481001 41.978001

HD12 24.254000 26.395000 42.931999

HD13 25.895000 26.059000 43.584999

CD2 25.975000 23.603001 44.057999

HD21 25.722000 22.572001 44.284000

HD22 26.656000 23.586000 43.203999

HD23 26.495001 24.016001 44.926998

C 22.538000 24.134001 47.033001

O 21.407000 24.542999 46.768002

N 23.101999 24.287001 48.224998

H 24.030001 23.910000 48.390999

CA 22.496000 25.096001 49.285000

HA 21.417999 25.108000 49.099998

CB 22.846001 24.448000 50.616001

HB2 23.936001 24.441000 50.719002

HB3 22.386999 24.981001 51.451000

OG 22.396000 23.099001 50.583000

HG 21.549000 23.030001 50.109001

C 22.931000 26.582001 49.249001

O 23.628000 27.113001 50.119999

N 22.549999 27.236000 48.160000

H 21.952999 26.716999 47.528000

CA 22.731001 28.677999 47.980000

HA 23.746000 28.951000 48.249001

CB 22.504000 29.066000 46.509998

HB 21.535999 28.709999 46.152000

CG1 22.579000 30.573999 46.368999

HG11 23.462999 30.951000 46.889000

HG12 22.589001 30.777000 45.299999

HG13 21.705000 31.039000 46.823002

CG2 23.587000 28.400000 45.639000

HG21 24.545000 28.870001 45.837002

HG22 23.615999 27.330000 45.846001

HG23 23.389000 28.572001 44.582001

C 21.856001 29.410000 48.980000

O 20.656000 29.162001 49.028999

N 22.417000 30.311001 49.786999

H 23.427000 30.423000 49.789001

CA 21.606001 31.357000 50.476002

HA 20.639999 31.377001 49.966999

CB 21.306999 31.110001 51.959999

HB2 20.861000 30.124001 52.070000

HB3 22.250999 31.073999 52.500999

OG 20.455000 32.165001 52.429001

HG 21.006001 32.928001 52.714001

C 22.306000 32.700001 50.362000

O 23.488001 32.808998 50.685001

N 21.561001 33.708000 49.910000

H 20.577999 33.488998 49.764999

CA 21.992001 35.108002 49.839001

HA 22.833000 35.230999 50.522999

CB 22.459999 35.480000 48.401001

HB2 21.549999 35.598000 47.831001

HB3 22.938000 36.460999 48.402000

CG 23.362000 34.556000 47.599998

CD1 24.580999 34.139000 48.155998

HD1 24.858999 34.394001 49.158001

CE1 25.506001 33.389999 47.415001

HE1 26.466999 33.123001 47.824001

CZ 25.145000 32.941002 46.132999

OH 25.937000 32.131001 45.375000

HH 26.841999 32.091000 45.699001

CE2 23.954000 33.368999 45.560001

HE2 23.871000 33.098000 44.523998

CD2 23.075001 34.230999 46.255001

HD2 22.180000 34.604000 45.775002

C 20.886000 36.083000 50.348999

O 20.913000 37.255001 49.997002

N 19.922001 35.626999 51.164001

H 20.040001 34.681999 51.493999

CA 18.789000 36.387001 51.722000

HA 18.155001 36.792000 50.924999

CB 17.948999 35.462002 52.618999

HB2 18.642000 34.950001 53.285000

HB3 17.296000 36.016998 53.297001

CG 17.139999 34.448002 51.842999

OD1 16.871000 34.587002 50.619999

OD2 16.851000 33.417000 52.480999

C 19.204000 37.555000 52.608002

O 18.445999 38.521000 52.618000

N 20.333000 37.488998 53.345001

H 20.882999 36.645000 53.301998

CA 20.806999 38.462002 54.366001

HA 20.011000 39.192001 54.566002

CB 21.216999 37.720001 55.696999

HB2 22.090000 37.078999 55.580002

HB3 21.542999 38.417000 56.473000

CG 20.134001 36.818001 56.285999

HG2 19.931000 35.993000 55.602001

HG3 20.466999 36.436001 57.247002

CD 18.827999 37.522999 56.569000

OE1 18.677000 38.728001 56.419998

NE2 17.794001 36.801998 56.943001

HE21 16.908001 37.285999 56.912998

HE22 17.874001 35.814999 57.169998

C 21.990999 39.291000 53.853001

O 22.687000 39.966000 54.631001

N 22.216000 39.257000 52.534000

H 21.586000 38.748001 51.921001

CA 23.292999 40.005001 51.889999

HA 24.250999 39.608002 52.237000

CB 23.298000 39.632000 50.411999

HB1 22.488001 40.032001 49.800999

HB2 24.270000 39.881001 49.993999

HB3 23.198000 38.558998 50.340000

C 23.250000 41.543999 52.125999

O 22.166000 42.145000 52.249001

N 24.455000 42.129002 52.112000

H 25.236000 41.493000 51.990002

CA 24.643999 43.577000 52.180000

HA 23.749001 44.022999 51.769001

CB 24.737000 44.186001 53.587002

HB 24.715000 45.270000 53.484001

CG2 23.639999 43.754002 54.542000

HG21 23.802000 44.340000 55.439999

HG22 22.655001 44.004002 54.143002

HG23 23.742001 42.687000 54.750999

OG1 25.941999 43.853001 54.202000

HG1 26.615000 44.027000 53.522999

C 25.805000 44.053001 51.311001

O 26.969000 44.023998 51.737000

N 25.459000 44.535999 50.109001

H 24.483999 44.493000 49.838001

CA 26.378000 45.212002 49.181000

HA 27.254000 44.577000 49.056000

CB 25.743999 45.375999 47.792000

HB2 26.516001 45.658001 47.075001

HB3 25.316000 44.422001 47.480999

OG 24.725000 46.360001 47.780998

HG 25.065001 47.167000 47.342999

C 26.858000 46.567001 49.714001

O 26.170000 47.231998 50.500999

N 28.046000 46.973000 49.263000

H 28.540001 46.342999 48.636002

CA 28.775999 48.139000 49.761002

HA 28.169001 48.655998 50.501999

CB 30.073999 47.671001 50.452999

HB2 30.715000 47.192001 49.712002

HB3 30.605000 48.549000 50.825001

CG 29.871000 46.685001 51.619999

HG 29.315001 45.814999 51.273998

CD1 31.229000 46.195000 52.116001

HD11 31.742001 45.660000 51.320000

HD12 31.839001 47.037998 52.436001

HD13 31.094999 45.515999 52.958000

CD2 29.125000 47.313000 52.799999

HD21 29.677999 48.172001 53.181000

HD22 28.128000 47.626999 52.492001

HD23 29.014999 46.577000 53.595001

C 29.059999 49.160999 48.654999

O 28.600000 50.299999 48.737000

N 29.784000 48.751999 47.605000

H 30.048000 47.775002 47.573002

CA 30.231001 49.613998 46.494999

HA 29.506001 50.415001 46.369999

CB 31.589001 50.283001 46.816002

HB2 31.872999 50.909000 45.967999

HB3 31.476000 50.937000 47.682999

CG 32.728001 49.290001 47.098000

HG2 32.550999 48.813999 48.062000

HG3 32.748001 48.528000 46.320000

CD 34.094002 49.981998 47.139999

HD2 34.209999 50.577999 46.234001

HD3 34.125999 50.646999 48.005001

NE 35.175999 48.987000 47.243999

HE 34.987000 48.160999 47.807999

CZ 36.352001 48.999001 46.643002

NH1 36.792999 50.009998 45.951000

HH11 36.277000 50.875999 45.949001

HH12 37.709000 49.951000 45.533001

NH2 37.123001 47.957001 46.705002

HH21 36.858002 47.150002 47.255001

HH22 37.983002 47.931999 46.166000

C 30.285999 48.870998 45.158001

O 30.516001 47.664001 45.136002

N 30.122999 49.598999 44.055000

H 29.929001 50.587002 44.185001

CA 30.445000 49.165001 42.683998

HA 30.618999 48.094002 42.713001

CB 29.239000 49.403000 41.745998

HB 28.388000 48.890999 42.196999

CG2 28.882999 50.893002 41.615002

HG21 27.910999 50.978001 41.141998

HG22 28.819000 51.369999 42.591000

HG23 29.625999 51.419998 41.014000

CG1 29.471001 48.789001 40.348000

HG12 30.225000 49.368000 39.825001

HG13 29.856001 47.779999 40.469002

CD1 28.230000 48.686001 39.451000

HD11 27.813999 49.669998 39.234001

HD12 28.518000 48.219002 38.507000

HD13 27.474001 48.069000 39.933998

C 31.752001 49.824001 42.210999

O 32.027000 50.966999 42.577000

N 32.591000 49.108002 41.452000

H 32.334999 48.146999 41.243000

CA 33.971001 49.523998 41.161999

HA 33.991001 50.612999 41.110001

CB 34.862000 49.090000 42.344002

HB2 35.839001 49.556999 42.212002

HB3 34.424000 49.494999 43.255001

CG 35.036999 47.562000 42.515999

HG 34.373001 47.028999 41.846001

CD1 36.474998 47.145000 42.210999

HD11 36.736000 47.448002 41.199001

HD12 37.161999 47.619999 42.912998

HD13 36.570999 46.063000 42.296001

CD2 34.693001 47.104000 43.931000

HD21 34.863998 46.032001 44.027000

HD22 35.319000 47.624001 44.647999

HD23 33.643002 47.310001 44.139999

C 34.525002 48.997002 39.827000

O 34.359001 47.820000 39.507000

N 35.254002 49.831001 39.078999

H 35.398998 50.777000 39.428001

CA 36.096001 49.386002 37.957001

HA 35.737000 48.417000 37.608002

CB 35.960999 50.381001 36.784000

HB2 34.928001 50.369999 36.445000

HB3 36.191002 51.383999 37.141998

CG 36.834999 50.124001 35.556999

OD1 37.615002 49.189999 35.474998

ND2 36.750999 50.973999 34.564999

HD21 37.345001 50.855999 33.757000

HD22 36.098000 51.747002 34.652000

C 37.546001 49.205002 38.439999

O 38.087002 50.054001 39.151001

N 38.179001 48.090000 38.057999

H 37.672001 47.453999 37.448002

CA 39.546001 47.694000 38.441002

HA 39.941002 48.412998 39.160999

CB 39.478001 46.331001 39.150002

HB2 40.478001 46.064999 39.485001

HB3 38.842999 46.415001 40.032001

CG 38.959000 45.199001 38.278000

OD1 38.826000 45.314999 37.069000

ND2 38.612000 44.085999 38.872002

HD21 38.292999 43.306999 38.300999

HD22 38.770000 43.958000 39.861000

C 40.562000 47.666000 37.273998

O 41.717999 47.283001 37.467999

N 40.155998 48.057999 36.061001

H 39.199001 48.382999 35.955002

CA 40.987000 47.979000 34.861000

HA2 40.743999 48.814999 34.208000

HA3 42.033001 48.053001 35.146000

C 40.846001 46.695999 34.046001

O 41.532001 46.567001 33.033001

N 39.971001 45.757000 34.425999

H 39.523998 45.859001 35.332001

CA 39.659000 44.554001 33.634998

HA 39.832001 44.782001 32.582001

CB 40.630001 43.417999 33.994999

HB2 40.296001 42.495998 33.492001

HB3 41.590000 43.708000 33.540001

CG 40.846001 43.138000 35.451000

ND1 40.056000 42.257000 36.159000

CE1 40.570999 42.164001 37.372002

HE1 40.162998 41.567001 38.185001

NE2 41.667000 42.957001 37.490002

HE2 42.320999 42.958000 38.278000

CD2 41.862999 43.582001 36.276001

HD2 42.700001 44.251999 36.096001

C 38.182999 44.106998 33.679001

O 37.748001 43.428001 32.743999

N 37.398998 44.528000 34.678001

H 37.835999 44.993999 35.466999

CA 35.931999 44.448002 34.708000

HA 35.549000 44.676998 33.712002

CB 35.507000 43.015999 35.070000

HB1 35.969002 42.294998 34.395000

HB2 35.799000 42.792999 36.092999

HB3 34.424999 42.912998 34.981998

C 35.320999 45.467999 35.700001

O 36.036999 46.095001 36.488998

N 33.987999 45.612000 35.692001

H 33.457001 45.035000 35.054001

CA 33.237999 46.279999 36.770000

HA 33.924000 46.925999 37.313000

CB 32.134998 47.191002 36.210999

HB2 31.650999 47.673000 37.058998

HB3 32.598000 47.973999 35.609001

CG 31.059000 46.507000 35.390999

CD1 31.198999 46.393002 33.994999

HD1 32.055000 46.823002 33.497002

CE1 30.232000 45.702000 33.243000

HE1 30.351000 45.595001 32.173000

CZ 29.114000 45.140999 33.882000

HZ 28.377001 44.598000 33.305000

CE2 28.948999 45.291000 35.269001

HE2 28.075001 44.877998 35.754002

CD2 29.920000 45.971001 36.023998

HD2 29.792000 46.073002 37.091999

C 32.686001 45.255001 37.776001

O 32.259998 44.168999 37.388000

N 32.683998 45.577000 39.068001

H 33.028999 46.492001 39.334000

CA 32.487999 44.601002 40.147999

HA 31.947001 43.750000 39.741001

CB 33.860001 44.099998 40.676998

HB2 34.189999 44.747002 41.487999

HB3 33.726002 43.105000 41.098999

CG 34.992001 44.040001 39.653999

OD1 35.319000 43.007000 39.091000

ND2 35.620998 45.153000 39.349998

HD21 36.308998 45.104000 38.613998

HD22 35.298000 46.036999 39.716999

C 31.635000 45.206001 41.278000

O 31.784000 46.395000 41.549000

N 30.782000 44.424000 41.956001

H 30.740999 43.443001 41.699001

CA 30.011999 44.876999 43.133999

HA 30.211000 45.933998 43.292000

CB 28.486000 44.736000 42.945999

HB 28.223000 43.680000 42.932999

CG1 27.730000 45.411999 44.099998

HG11 27.983000 46.471001 44.146999

HG12 26.656000 45.306000 43.950001

HG13 27.986000 44.944000 45.049999

CG2 28.004999 45.368000 41.633999

HG21 26.919001 45.317001 41.574001

HG22 28.316000 46.409000 41.584999

HG23 28.419001 44.820000 40.789001

C 30.500999 44.139999 44.381001

O 30.454000 42.912998 44.419998

N 30.989000 44.884998 45.375999

H 30.944000 45.891998 45.254002

CA 31.568001 44.384998 46.634998

HA 31.976999 43.395000 46.436001

CB 32.745998 45.287998 47.066002

HB2 33.507000 45.226002 46.286999

HB3 32.409000 46.321999 47.127998

CG 33.380001 44.888000 48.410999

HG2 32.785999 45.299999 49.228001

HG3 33.362999 43.799000 48.493000

CD 34.834999 45.355999 48.549999

OE1 35.738998 44.491001 48.487000

OE2 35.103001 46.573002 48.705002

C 30.531000 44.223999 47.764999

O 29.625000 45.048000 47.916000

N 30.705999 43.187000 48.591999

H 31.493999 42.569000 48.419998

CA 29.841999 42.824001 49.720001

HA 29.077000 43.587002 49.862000

CB 29.139999 41.488998 49.396999

HB2 29.882999 40.765999 49.055000

HB3 28.714001 41.088001 50.314999

CG 28.032000 41.594002 48.356998

CD1 28.341999 41.758999 46.990002

HD1 29.372000 41.756001 46.660999

CE1 27.316000 41.952999 46.048000

HE1 27.566999 42.125000 45.011002

CZ 25.969999 41.938999 46.458000

HZ 25.174000 42.073002 45.736000

CE2 25.653999 41.747002 47.813999

HE2 24.618000 41.757999 48.127998

CD2 26.680000 41.570000 48.758999

HD2 26.426001 41.422001 49.798000

C 30.621000 42.747002 51.043999

O 31.822001 42.480000 51.068001

N 29.920000 42.956001 52.159000

H 28.934999 43.176998 52.062000

CA 30.402000 42.578999 53.493999

HA 31.421000 42.935001 53.658001

CB 29.462000 43.283001 54.476002

HB2 29.434000 44.363998 54.319000

HB3 28.441000 42.966999 54.285999

CG 29.757000 42.988998 55.928001

OD1 28.850000 43.365002 56.702000

OD2 30.841999 42.466000 56.282001

C 30.479000 41.028999 53.688000

O 29.530001 40.266998 53.466000

N 31.601000 40.508999 54.192001

H 32.332001 41.113998 54.535000

CA 31.952000 39.082001 54.351002

HA 31.076000 38.452000 54.164001

CB 33.040001 38.735001 53.335999

HB2 33.247002 37.669998 53.424999

HB3 32.609001 38.925999 52.355000

CG 34.377998 39.501999 53.505001

OD1 34.493000 40.342999 54.438999

OD2 35.308998 39.264000 52.700001

C 32.497002 38.768002 55.743000

O 33.056000 37.701000 55.977001

N 32.289001 39.692001 56.667999

H 31.850000 40.542000 56.325001

CA 32.609001 39.521000 58.095001

HA 33.685001 39.334000 58.164001

CB 32.319000 40.847000 58.791000

HB2 32.494999 40.651001 59.834999

HB3 33.036999 41.615002 58.520000

OG 31.010000 41.341000 58.611000

HG 31.033001 41.759998 57.720001

C 31.792999 38.428001 58.868999

O 32.210999 37.931999 59.922001

N 30.576000 38.181000 58.363998

H 30.350000 38.830002 57.627998

CA 29.455000 37.324001 58.792999

HA 29.731001 36.619999 59.584999

CB 28.333000 38.198002 59.360001

HB2 28.186001 39.041000 58.682999

HB3 27.412001 37.616001 59.424999

CG 28.629999 38.669998 60.770000

HG2 28.549000 37.770000 61.383999

HG3 29.655001 39.026001 60.877998

CD 27.656000 39.813999 61.101002

OE1 28.063999 40.923000 61.403000

NE2 26.368000 39.678001 60.910000

HE21 25.764999 40.411999 61.270000

HE22 26.004000 38.764000 60.708000

C 28.930000 36.574001 57.551998

O 28.979000 37.078999 56.431000

N 28.427000 35.369999 57.784000

H 28.490000 35.132000 58.762001

CA 27.780001 34.393002 56.856998

HA 28.450001 34.235001 56.013000

CB 27.579000 33.048000 57.615002

HB2 26.958000 33.324001 58.459000

HB3 26.948000 32.303001 57.119999

CG 28.827000 32.354000 58.178001

OD1 28.648001 31.242001 58.702000

OD2 29.937000 32.931999 58.257999

C 26.448000 34.866001 56.215000

O 25.518999 34.064999 56.021000

N 26.341000 36.166000 55.879002

H 27.172001 36.733002 56.019001

CA 25.180000 36.844002 55.243999

HA 24.323999 36.741001 55.915001

CB 25.606001 38.314999 54.980999

HB2 26.537001 38.320999 54.407001

HB3 24.867001 38.812000 54.353001

CG 25.691000 39.169998 56.252998

HG2 24.669001 39.186001 56.611000

HG3 26.245001 38.770000 57.103001

CD 26.221001 40.589001 56.021000

HD2 27.315001 40.547001 56.018002

HD3 25.871000 41.046001 55.091000

CE 25.695999 41.401001 57.212002

HE2 24.614000 41.264999 57.341000

HE3 26.122000 41.007999 58.133999

NZ 25.948999 42.835999 57.020000

HZ1 26.912001 43.159000 57.049999

HZ2 25.605000 43.083000 56.105000

HZ3 25.448999 43.355000 57.737999

C 24.740999 36.278999 53.895000

O 23.549999 36.198002 53.589001

N 25.719000 35.862999 53.104000

H 26.634001 35.803001 53.529999

CA 25.591000 35.435001 51.734001

HA 24.605000 34.990002 51.605999

CB 25.774000 36.672001 50.823002

HB1 25.708000 36.410999 49.769001

HB2 25.020000 37.445999 50.990002

HB3 26.773001 37.047001 51.044998

C 26.667000 34.352001 51.577000

O 27.872000 34.591000 51.601002

N 26.240000 33.105999 51.448002

H 25.309000 32.966000 51.077000

CA 27.135000 31.954000 51.551998

HA 28.124001 32.301998 51.266998

CB 27.254000 31.384001 52.963001

HB 27.877001 30.504000 52.780998

CG1 27.966000 32.286999 53.965000

HG11 28.889000 32.638000 53.521999

HG12 27.386000 33.171001 54.196999

HG13 28.090000 31.820000 54.943001

CG2 25.913000 31.056000 53.676998

HG21 25.636000 31.761000 54.466000

HG22 25.054001 31.070000 53.009998

HG23 26.002001 30.091999 54.165001

C 26.792000 30.827000 50.570000

O 25.632000 30.452999 50.377998

N 27.829000 30.271999 49.952000

H 28.752001 30.631001 50.169998

CA 27.771000 29.094999 49.092999

HA 26.743000 28.941000 48.769001

CB 28.601999 29.410000 47.837002

HB2 28.183001 30.298000 47.367001

HB3 29.615999 29.660000 48.141998

CG 28.670000 28.312000 46.769001

HG 29.086000 27.413000 47.206001

CD1 27.309000 27.982000 46.155998

HD11 26.881001 28.884001 45.714001

HD12 27.436001 27.226999 45.379002

HD13 26.636999 27.586000 46.915001

CD2 29.586000 28.797001 45.646999

HD21 30.572001 29.033001 46.051998

HD22 29.686001 28.021999 44.894001

HD23 29.166000 29.691000 45.188000

C 28.214001 27.865999 49.910999

O 29.261000 27.881001 50.553001

N 27.372999 26.830000 49.914001

H 26.546000 26.934999 49.345001

CA 27.443001 25.577999 50.713001

HA 28.433001 25.406000 51.147999

CB 26.403000 25.632000 51.866001

HB2 25.461000 25.933001 51.409000

HB3 26.290001 24.613001 52.242001

CG 26.750000 26.563000 53.051998

HG2 27.753000 26.326000 53.391998

HG3 26.743999 27.617001 52.759998

CD 25.795000 26.403999 54.240002

HD2 24.827999 26.763000 53.924999

HD3 25.618999 25.382999 54.584999

CE 26.247999 27.263000 55.408001

HE2 27.193001 26.917000 55.826000

HE3 26.409000 28.285000 55.071999

NZ 25.243999 27.268999 56.476002

HZ1 25.603001 27.851000 57.215000

HZ2 24.374001 27.695000 56.167999

HZ3 25.091999 26.343000 56.868999

C 27.120001 24.395000 49.792000

O 26.205000 24.497999 48.972000

N 27.851999 23.285999 49.930000

H 28.596001 23.304001 50.613998

CA 27.527000 21.989000 49.306000

HA2 28.056000 21.229000 49.866001

HA3 26.448999 21.809000 49.363998

C 27.948999 21.754999 47.853001

O 28.830000 22.424999 47.311001

N 27.306000 20.771999 47.202000

H 26.680000 20.162001 47.716999

CA 27.660999 20.292999 45.875000

HA2 26.936001 19.528000 45.588001

HA3 27.648001 21.087999 45.127998

C 29.052000 19.662001 45.962002

O 29.181999 18.700001 46.724998

N 30.059999 20.197001 45.235001

CD 29.959999 21.348000 44.372002

HD2 29.625999 22.167000 45.001999

HD3 29.239000 21.143999 43.590000

CG 31.323999 21.631001 43.789001

HG2 31.723000 22.518999 44.285000

HG3 31.267000 21.745001 42.708000

CB 32.139999 20.384001 44.139000

HB2 33.148998 20.719999 44.379002

HB3 32.092999 19.642000 43.334000

CA 31.441999 19.818001 45.389000

HA 31.482000 18.728001 45.395000

C 32.131001 20.424999 46.632000

O 33.244999 20.030001 46.962002

N 31.500999 21.364000 47.344002

H 30.510000 21.452000 47.166000

CA 32.018002 22.087000 48.516998

HA 33.111000 22.075001 48.480999

CB 31.569000 23.598000 48.462002

HB2 30.488001 23.639000 48.553001

HB3 31.971001 24.142000 49.321999

CG 31.889999 24.386000 47.164001

HG 31.433001 23.907000 46.305000

CD1 31.518000 25.871000 47.195000

HD11 31.267000 26.219999 46.196999

HD12 30.632000 26.004999 47.806999

HD13 32.345001 26.488001 47.556000

CD2 33.337002 24.288000 46.799999

HD21 33.470001 24.730000 45.821999

HD22 33.969002 24.826000 47.498001

HD23 33.629002 23.243000 46.736000

C 31.597000 21.392000 49.834000

O 30.424999 21.188000 50.147999

N 32.583000 21.059999 50.655998

H 33.515999 21.163000 50.292000

CA 32.527000 20.565001 52.033001

HA 31.695999 19.882000 52.150002

CB 33.874001 19.846001 52.280998

HB2 34.619999 20.642000 52.391998

HB3 33.782001 19.301001 53.214001

CG 34.300999 18.900999 51.141998

OD1 35.360001 19.243000 50.549999

OD2 33.636002 17.878000 50.834999

C 32.238998 21.695999 53.006001

O 31.549000 21.542999 54.011002

N 32.727001 22.874001 52.662998

H 33.249001 22.886000 51.793999

CA 32.702000 24.054001 53.508999

HA2 32.563000 23.820999 54.564999

HA3 33.695000 24.469999 53.415001

C 31.681999 25.115999 53.144001

O 31.016001 25.014999 52.119999

N 31.532000 26.113001 54.027000

H 32.113998 26.117001 54.853001

CA 30.837000 27.361000 53.702000

HA 30.063000 27.152000 52.966999

CB 30.257999 28.054001 54.959000

HB 31.087999 28.364000 55.589001

CG2 29.434999 29.318001 54.714001

HG21 29.034000 29.650000 55.667999

HG22 30.065001 30.128000 54.334999

HG23 28.584999 29.091000 54.073002

OG1 29.452999 27.188999 55.709999

HG1 30.020000 26.622999 56.255001

C 31.801001 28.365000 53.113998

O 32.855999 28.552999 53.736000

N 31.382999 29.055000 52.030998

H 30.535000 28.725000 51.573002

CA 32.129002 30.112000 51.313999

HA 33.104000 30.224001 51.778999

CB 32.474998 29.725000 49.844002

HB2 31.611000 29.514999 49.209999

HB3 32.959999 30.572001 49.366001

CG 33.437000 28.577999 49.777000

CD1 33.051998 27.313000 50.250000

HD1 32.049999 27.115000 50.609001

CE1 33.966000 26.240999 50.220001

HE1 33.620998 25.271000 50.549999

CZ 35.238998 26.385000 49.595001

OH 35.993999 25.285000 49.307999

HH 36.778999 25.444000 48.763000

CE2 35.606998 27.662001 49.112000

HE2 36.509998 27.792000 48.539001

CD2 34.729000 28.760000 49.230000

HD2 35.050999 29.716999 48.837002

C 31.347000 31.424000 51.345001

O 30.289000 31.502001 50.726002

N 31.861000 32.438999 52.047001

H 32.744999 32.248001 52.515999

CA 31.306999 33.834999 52.070999

HA 30.235001 33.859001 52.248001

CB 32.000999 34.645000 53.182999

HB2 33.066002 34.445999 53.120998

HB3 31.796000 35.719002 53.133999

CG 31.544001 34.131001 54.508999

HG2 30.469999 34.127998 54.493000

HG3 31.889999 33.108002 54.612000

CD 31.973000 35.104000 55.598000

HD2 33.049000 35.250999 55.534000

HD3 31.487000 36.060001 55.418999

NE 31.627001 34.702999 56.958000

HE 30.826000 34.088001 57.091999

CZ 32.429001 34.921001 57.978001

NH1 33.414001 35.782001 58.018002

HH11 33.484001 36.495998 57.303001

HH12 33.925999 35.952999 58.870998

NH2 32.294998 34.205002 59.023998

HH21 31.466999 33.612000 59.084000

HH22 33.136002 34.091999 59.557999

C 31.419001 34.525002 50.717999

O 32.500999 34.480999 50.143002

N 30.355000 35.148998 50.200001

H 29.499001 35.144001 50.743999

CA 30.386000 35.965000 48.971001

HA 30.914000 35.410000 48.193001

CB 28.934000 36.238998 48.507000

HB2 28.482000 35.303001 48.179001

HB3 28.386999 36.577000 49.382999

CG 28.724001 37.310001 47.408001

HG 29.139000 38.258999 47.743999

CD1 29.368999 36.926998 46.077999

HD11 30.441000 36.834999 46.202000

HD12 28.962999 35.977001 45.729000

HD13 29.180000 37.702000 45.337002

CD2 27.235001 37.528000 47.125999

HD21 26.781000 36.606998 46.757000

HD22 26.725000 37.848999 48.032001

HD23 27.114000 38.310001 46.374001

C 31.141001 37.280998 49.228001

O 30.813000 37.987999 50.182999

N 32.095001 37.632999 48.356998

H 32.310001 36.973999 47.615002

CA 32.896000 38.874001 48.441002

HA 32.630001 39.393002 49.368000

CB 34.398998 38.516998 48.542000

HB 34.483002 37.818001 49.370998

CG2 34.965000 37.834000 47.278999

HG21 34.403999 36.933998 47.054001

HG22 34.935001 38.507000 46.422001

HG23 35.999001 37.532001 47.439999

CG1 35.251999 39.750999 48.896000

HG12 35.305000 40.414001 48.036999

HG13 34.765999 40.284000 49.712002

CD1 36.673000 39.414001 49.358002

HD11 37.172001 40.323002 49.687000

HD12 36.615002 38.721001 50.194000

HD13 37.257999 38.973000 48.553001

C 32.601002 39.872002 47.308998

O 32.596001 41.082001 47.551998

N 32.334000 39.394001 46.087002

H 32.335999 38.391998 45.944000

CA 31.962999 40.228001 44.933998

HA 31.184000 40.903999 45.271000

CB 33.172001 41.056000 44.436001

HB2 32.856998 41.700001 43.613998

HB3 33.494999 41.723000 45.233002

CG 34.376999 40.209999 43.986000

HG2 35.251999 40.514999 44.560001

HG3 34.205002 39.158001 44.199001

CD 34.696999 40.340000 42.500999

OE1 34.180000 39.618000 41.660000

NE2 35.574001 41.237000 42.124001

HE21 36.039001 41.106998 41.230999

HE22 35.953999 41.891998 42.806000

C 31.389999 39.412998 43.763000

O 31.511999 38.187000 43.730000

N 30.827999 40.109001 42.771999

H 30.740000 41.112000 42.884998

CA 30.563999 39.562000 41.436001

HA 31.177999 38.669998 41.317001

CB 29.093000 39.138000 41.306999

HB2 28.990000 38.582001 40.375999

HB3 28.862000 38.449001 42.118999

CG 28.059999 40.254002 41.292999

CD1 27.759001 40.926998 40.091000

HD1 28.278999 40.667000 39.179001

CE1 26.775999 41.931999 40.068001

HE1 26.549000 42.446999 39.146000

CZ 26.075001 42.258999 41.241001

HZ 25.316999 43.028999 41.221001

CE2 26.358999 41.578999 42.438000

HE2 25.809999 41.823002 43.334999

CD2 27.354000 40.584000 42.466000

HD2 27.566999 40.062000 43.388000

C 30.988001 40.527000 40.318001

O 31.045000 41.738998 40.537998

N 31.267000 39.991001 39.123001

H 31.184000 38.980000 39.029999

CA 31.674999 40.729000 37.917000

HA 31.134001 41.679001 37.882000

CB 33.182999 41.030998 37.986000

HB2 33.452000 41.751999 37.199001

HB3 33.339001 41.539001 38.945999

CG 34.044998 39.814999 37.879002

ND1 34.588001 39.155998 38.965000

HD1 34.507999 39.436001 39.951000

CE1 35.251999 38.069000 38.534000

HE1 35.846001 37.412998 39.169998

NE2 35.159000 37.984001 37.208000

CD2 34.423000 39.075001 36.782001

HD2 34.203999 39.256001 35.730999

C 31.348000 39.952999 36.620998

O 31.018000 38.768002 36.674999

N 31.469999 40.595001 35.455002

H 31.784000 41.558998 35.453999

CA 31.174000 40.021000 34.136002

HA 30.871000 38.984001 34.257999

CB 30.000000 40.783001 33.487000

HB2 30.315001 41.808998 33.291000

HB3 29.792999 40.333000 32.514999

CG 28.695000 40.830002 34.269001

CD1 27.627001 39.980999 33.917999

HD1 27.749001 39.261002 33.120998

CE1 26.396000 40.077000 34.594002

HE1 25.577000 39.428001 34.320000

CZ 26.226999 41.013000 35.627998

HZ 25.281000 41.082001 36.146999

CE2 27.291000 41.858002 35.986000

HE2 27.164000 42.583000 36.776001

CD2 28.518000 41.770000 35.303001

HD2 29.326000 42.435001 35.568001

C 32.396999 40.036999 33.195000

O 33.379002 40.740002 33.431999

N 32.299999 39.301998 32.084999

H 31.499001 38.682999 32.009998

CA 33.155998 39.411999 30.896999

HA 33.744999 40.332001 30.962000

CB 34.125000 38.222000 30.796000

HB2 33.523998 37.335999 30.542000

HB3 34.819000 38.401001 29.959999

CG 34.879002 37.984001 32.063000

ND1 36.134998 38.497002 32.319000

HD1 36.708000 39.058998 31.677000

CE1 36.519001 38.117001 33.549999

HE1 37.472000 38.380001 33.997002

NE2 35.564999 37.382999 34.115002

CD2 34.536999 37.284000 33.199001

HD2 33.620998 36.737999 33.410000

C 32.278000 39.500999 29.636000

O 31.169001 38.958000 29.625000

N 32.740002 40.203999 28.598000

H 33.653999 40.643002 28.677000

CA 31.979000 40.479000 27.372999

HA 31.343000 39.618999 27.160999

CB 31.061001 41.693001 27.579000

HB2 30.474001 41.852001 26.673000

HB3 30.355000 41.459999 28.377001

CG 31.757999 42.971001 27.930000

CD1 32.188999 43.903000 27.051001

HD1 32.075001 43.830002 25.976999

NE1 32.813999 44.930000 27.733000

HE1 33.264000 45.733002 27.294001

CE2 32.828999 44.698002 29.087999

CZ2 33.317001 45.432999 30.174000

HZ2 33.762001 46.403000 30.007000

CH2 33.227001 44.889000 31.466000

HH2 33.624001 45.431999 32.310001

CZ3 32.624001 43.632999 31.653000

HZ3 32.562000 43.205002 32.643002

CE3 32.085999 42.929001 30.559999

HE3 31.587000 41.987000 30.724001

CD2 32.185001 43.437000 29.247000

C 32.884998 40.696999 26.150999

O 34.074001 41.013000 26.247000

N 32.310001 40.504002 24.966999

H 31.315001 40.323002 24.931000

CA 32.981998 40.622002 23.677999

HA2 34.043999 40.437000 23.796000

HA3 32.590000 39.873001 22.993999

C 32.786999 41.974998 23.006001

O 31.965000 42.789001 23.423000

N 33.549999 42.198002 21.940001

H 34.277000 41.513000 21.733999

CA 33.396999 43.334000 21.018999

HA 33.166000 44.227001 21.599001

CB 34.724998 43.557999 20.295000

HB2 34.577999 44.341999 19.562000

HB3 35.476002 43.882999 21.014000

OG 35.191002 42.390999 19.646000

HG 35.952000 42.049000 20.180000

C 32.259998 43.152000 19.999001

O 31.618999 44.125000 19.597000

N 31.969000 41.903999 19.632999

H 32.541000 41.193001 20.066000

CA 30.754999 41.465000 18.910000

HA 30.143999 42.346001 18.698000

CB 31.173000 40.910000 17.528000

HB2 30.250000 40.618999 17.030001

HB3 31.641001 41.720001 16.958000

CG 32.112999 39.683998 17.594999

HG 32.102001 39.259998 18.598000

CD1 31.747999 38.557999 16.641001

HD11 30.872000 38.056000 17.024000

HD12 31.497999 38.967999 15.664000

HD13 32.528000 37.791000 16.594999

CD2 33.564999 40.074001 17.306000

HD21 34.153999 39.172001 17.455000

HD22 33.727001 40.525002 16.320999

HD23 33.852001 40.803001 18.055000

C 29.923000 40.451000 19.752001

O 30.459999 39.824001 20.665001

N 28.645000 40.234001 19.420000

H 28.230000 40.875999 18.757000

CA 27.747000 39.353001 20.221001

HA 27.767000 39.728001 21.243000

CB 26.282000 39.425999 19.719000

HB2 26.302000 38.981998 18.729000

HB3 25.603001 38.756001 20.257000

CG 25.657000 40.831001 19.650000

OD1 26.219999 41.801998 20.214001

OD2 24.608999 40.944000 18.976999

C 28.160000 37.858002 20.238001

O 27.857000 37.095001 21.160000

N 28.893999 37.418999 19.205999

H 29.125999 38.111000 18.513000

CA 29.417000 36.051998 18.941000

HA2 28.596001 35.342999 18.787001

HA3 29.976999 36.048000 18.009001

C 30.479000 35.546001 19.926001

O 30.986000 34.442001 19.736000

N 30.825001 36.362999 20.923000

H 30.320999 37.237999 21.009001

CA 31.798000 36.087002 21.972000

HA 31.681999 35.043999 22.268999

CB 33.223000 36.287998 21.420000

HB2 33.938000 35.918999 22.153000

HB3 33.320999 35.662998 20.534000

CG 33.591000 37.747002 21.075001

HG2 32.700001 38.319000 20.823999

HG3 34.039001 38.213001 21.952000

CD 34.541000 37.881001 19.889000

OE1 34.488998 37.150002 18.907000

NE2 35.423000 38.852001 19.917999

HE21 36.021000 38.987999 19.122999

HE22 35.486000 39.473999 20.725000

C 31.549999 36.974998 23.202999

O 30.893000 38.015999 23.122999

N 32.126999 36.575001 24.330000

H 32.618000 35.687000 24.302000

CA 32.089001 37.290001 25.604000

HA2 32.903999 38.007999 25.627001

HA3 31.139000 37.812000 25.695999

C 32.237000 36.369999 26.809000

O 32.837002 36.766998 27.806000

N 31.763000 35.127998 26.700001

H 31.232000 34.879002 25.872000

CA 32.036999 34.084999 27.688000

HA 31.856001 34.521000 28.665001

CB 31.059999 32.915001 27.521999

HB2 31.194000 32.223000 28.354000

HB3 30.042999 33.305000 27.566000

OG 31.233999 32.209999 26.305000

HG 31.843000 31.472000 26.509001

C 33.507000 33.646000 27.669001

O 34.139999 33.539001 26.614000

N 34.070000 33.396000 28.848000

H 33.445000 33.345001 29.650000

CA 35.409000 32.841000 29.027000

HA 36.091000 33.278999 28.292000

CB 35.935001 33.195999 30.433001

HB2 35.366001 32.617001 31.174999

HB3 36.983002 32.868000 30.490999

CG 35.868999 34.703999 30.764000

HG2 35.980000 35.326000 29.867001

HG3 34.901001 34.959999 31.223000

CD 36.999001 35.074001 31.724001

OE1 36.874001 34.707001 32.931999

OE2 37.999001 35.657001 31.247999

C 35.387001 31.319000 28.791000

O 36.167999 30.787001 27.993999

N 34.429001 30.631001 29.416000

H 33.758999 31.160000 29.973000

CA 34.082001 29.250999 29.087999

HA 34.999001 28.674999 28.969000

CB 33.268002 28.617001 30.232000

HB2 32.369999 29.212999 30.393000

HB3 32.958000 27.614000 29.934000

CG 33.994999 28.510000 31.552000

ND1 34.132000 29.506001 32.487999

HD1 33.643002 30.406000 32.469002

CE1 34.844002 29.023001 33.519001

HE1 35.076000 29.573999 34.423000

NE2 35.200001 27.745001 33.286999

CD2 34.639000 27.408001 32.047001

HD2 34.685001 26.440001 31.572001

C 33.313000 29.177999 27.754999

O 32.691002 30.142000 27.298000

N 33.308998 27.990000 27.158001

H 33.838001 27.243999 27.589001

CA 32.408001 27.598000 26.062000

HA 31.646999 28.367001 25.933001

CB 33.148998 27.455000 24.721001

HB 32.497002 26.945000 24.010000

CG2 33.509998 28.820999 24.163000

HG21 33.970001 28.711000 23.184000

HG22 32.598999 29.403000 24.072001

HG23 34.189999 29.329000 24.844999

OG1 34.347000 26.722000 24.820000

HG1 34.070999 25.789000 24.955000

C 31.673000 26.313999 26.438000

O 32.077000 25.634001 27.379999

N 30.591999 25.969999 25.733000

H 30.304001 26.577999 24.971001

CA 29.849001 24.711000 25.938999

HA 30.341999 24.128000 26.718000

CB 28.408001 24.957001 26.430000

HB 27.855000 25.500000 25.664000

CG1 27.686001 23.634001 26.722000

HG11 28.174999 23.115000 27.547001

HG12 26.650999 23.840000 26.988001

HG13 27.690001 22.985001 25.847000

CG2 28.396000 25.770000 27.732000

HG21 27.370001 25.909000 28.075001

HG22 28.965000 25.246000 28.500999

HG23 28.841000 26.752001 27.573999

C 29.909000 23.899000 24.649000

O 29.079000 24.052999 23.756001

N 30.955000 23.090000 24.528000

H 31.591999 23.118000 25.309000

CA 31.441999 22.441999 23.305000

HA 32.469002 22.150999 23.510000

CB 30.624001 21.181999 23.000000

HB2 30.617001 20.517000 23.862000

HB3 29.610001 21.523001 22.790001

CG 31.171000 20.327000 21.857000

OD1 32.407001 20.263000 21.653000

OD2 30.365000 19.591999 21.247000

C 31.511999 23.410999 22.143000

O 30.679001 23.403999 21.242001

N 32.458000 24.319000 22.288000

H 33.084000 24.138000 23.076000

CA 32.700001 25.438000 21.358999

HA 33.470001 26.084999 21.792999

CB 33.159000 24.849001 19.993000

HB2 32.310001 24.346001 19.532000

HB3 33.395000 25.622999 19.261999

CG 34.374001 23.884001 20.134001

HG2 35.254002 24.482000 20.377001

HG3 34.201000 23.146000 20.922001

CD 34.685001 23.122999 18.837999

HD2 34.726002 23.841999 18.013000

HD3 35.604000 22.545000 18.952000

CE 33.655998 22.049000 18.500999

HE2 32.644001 22.461000 18.454000

HE3 33.910000 21.697001 17.507000

NZ 33.757000 20.848000 19.370001

HZ1 33.472000 21.002001 20.333000

HZ2 33.162998 20.103001 19.044001

HZ3 34.678001 20.423000 19.361000

C 31.500000 26.400000 21.216999

O 31.732000 27.452999 20.643000

N 30.268999 26.125999 21.688999

H 30.059000 25.155001 21.885000

CA 29.193001 27.125000 21.804001

HA 29.017000 27.575001 20.830999

CB 27.843000 26.473000 22.212999

HB2 27.702000 25.521999 21.690001

HB3 27.830999 26.275000 23.278000

CG 26.618000 27.368999 21.943001

HG2 26.886999 28.357000 22.313999

HG3 26.464001 27.363001 20.860001

CD 25.325001 26.941000 22.660999

HD2 25.551001 26.600000 23.672001

HD3 24.691999 27.833000 22.665001

CE 24.709000 25.795000 21.886999

HE2 24.653000 26.115999 20.847000

HE3 25.392000 24.941999 21.952000

NZ 23.339001 25.506001 22.363001

HZ1 23.287001 25.322001 23.358999

HZ2 22.757000 26.301001 22.121000

HZ3 22.962000 24.700001 21.872000

C 29.663000 28.288000 22.684000

O 30.250999 28.076000 23.749001

N 29.417000 29.520000 22.222000

H 28.906000 29.573999 21.346001

CA 29.768000 30.799999 22.874001

HA 30.315001 30.591999 23.794001

CB 30.646999 31.662001 21.938999

HB2 30.243000 31.636999 20.924999

HB3 30.625000 32.696999 22.285999

CG 32.110001 31.211000 21.941999

HG2 32.456001 31.249001 22.974001

HG3 32.180000 30.188000 21.569000

CD 33.042999 32.110001 21.127001

HD2 32.819000 32.028999 20.061001

HD3 32.924999 33.148998 21.441000

CE 34.478001 31.662001 21.419001

HE2 34.643002 31.742001 22.496000

HE3 34.599998 30.614000 21.128000

NZ 35.452000 32.521000 20.721001

HZ1 35.375999 33.470001 21.062000

HZ2 36.410000 32.237000 20.913000

HZ3 35.316002 32.502998 19.714001

C 28.533001 31.594999 23.292999

O 27.434999 31.377001 22.775999

N 28.740999 32.560001 24.188999

H 29.680000 32.685001 24.552000

CA 27.701000 33.484001 24.650999

HA 26.881001 33.448002 23.934000

CB 27.136000 33.030998 26.011999

HB2 27.919001 33.039001 26.770000

HB3 26.365999 33.733002 26.329000

CG 26.525999 31.646999 25.995001

CD1 25.135000 31.486000 25.820999

HD1 24.497999 32.354000 25.724001

CE1 24.576000 30.195000 25.805000

HE1 23.509001 30.066000 25.697001

CZ 25.415001 29.068001 25.965000

OH 24.896000 27.815001 25.915001

HH 24.025000 27.805000 25.502001

CE2 26.796000 29.236000 26.193001

HE2 27.417000 28.374001 26.379000

CD2 27.353001 30.527000 26.198000

HD2 28.414000 30.660000 26.368999

C 28.193001 34.939999 24.680000

O 29.382000 35.207001 24.850000

N 27.261999 35.886002 24.542999

H 26.320999 35.584999 24.315001

CA 27.511000 37.330002 24.511000

HA 28.160999 37.546001 23.664000

CB 26.152000 38.016998 24.292000

HB1 26.273001 39.098999 24.229000

HB2 25.705000 37.662998 23.363001

HB3 25.472000 37.794998 25.115000

C 28.201000 37.893002 25.771000

O 28.868000 38.926998 25.700001

N 28.041000 37.223000 26.916000

H 27.492001 36.375999 26.893999

CA 28.683001 37.547001 28.186001

HA 29.714001 37.839001 27.988001

CB 27.945000 38.736000 28.829000

HB1 28.000999 39.606998 28.174999

HB2 26.900000 38.483002 28.999001

HB3 28.412001 38.992001 29.781000

C 28.711000 36.328999 29.131001

O 27.983000 35.351002 28.931999

N 29.518000 36.421001 30.190001

H 30.104000 37.248001 30.261000

CA 29.561001 35.466999 31.309000

HA 28.614000 34.930000 31.351999

CB 30.663000 34.426998 31.052000

HB2 30.400000 33.898998 30.136999

HB3 31.610001 34.949001 30.907000

CG 30.848000 33.377998 32.158001

HG2 31.083000 33.883999 33.096001

HG3 29.914000 32.830002 32.285999

CD 31.974001 32.381001 31.840000

OE1 32.398998 32.251999 30.676001

OE2 32.469002 31.715000 32.780998

C 29.737000 36.203999 32.647999

O 30.540001 37.131001 32.748001

N 28.975000 35.794998 33.665001

H 28.368999 34.997002 33.493000

CA 28.958000 36.337002 35.029999

HA 29.452999 37.308998 35.048000

CB 27.476999 36.536999 35.418999

HB2 27.084000 37.362000 34.828999

HB3 26.934999 35.641998 35.113998

CG 27.134001 36.779999 36.903000

HG 27.417000 35.903999 37.487000

CD1 27.802000 38.014000 37.507999

HD11 27.384001 38.215000 38.493000

HD12 28.867001 37.842999 37.625999

HD13 27.648001 38.880001 36.866001

CD2 25.622000 36.979000 37.033001

HD21 25.347000 37.032001 38.085999

HD22 25.319000 37.897999 36.533001

HD23 25.094000 36.139999 36.580002

C 29.705000 35.396999 35.988998

O 29.472000 34.189999 35.969002

N 30.561001 35.948002 36.855999

H 30.697001 36.953999 36.800999

CA 31.257999 35.245998 37.938000

HA 30.951000 34.199001 37.953999

CB 32.778999 35.299000 37.702999

HB2 33.049999 36.365002 37.645000

HB3 33.311001 34.891998 38.580002

CG 33.226002 34.589001 36.460999

ND1 34.403999 34.917999 35.798000

CE1 34.530998 34.088001 34.757000

HE1 35.334999 34.132999 34.007999

NE2 33.493000 33.237000 34.738998

HE2 33.268002 32.555000 33.999001

CD2 32.655998 33.533001 35.790001

HD2 31.726999 32.997002 35.952000

C 30.914000 35.854000 39.306000

O 31.059999 37.062000 39.493999

N 30.507000 35.020000 40.270000

H 30.365999 34.046001 40.015999

CA 30.327999 35.383999 41.682999

HA 30.475000 36.459000 41.796001

CB 28.899000 35.063999 42.169998

HB2 28.766001 33.986000 42.219002

HB3 28.825001 35.438999 43.191002

CG 27.759001 35.675999 41.325001

HG 28.136999 36.513000 40.743999

CD1 27.143000 34.653999 40.367001

HD11 26.372000 35.133999 39.764000

HD12 27.907000 34.257999 39.699001

HD13 26.695999 33.835999 40.930000

CD2 26.634001 36.187000 42.229000

HD21 26.223000 35.368000 42.820000

HD22 27.018000 36.956001 42.898998

HD23 25.841999 36.624001 41.619999

C 31.389000 34.676998 42.542999

O 31.461000 33.445000 42.570000

N 32.228001 35.452000 43.231998

H 32.048000 36.452999 43.244999

CA 33.423000 34.973999 43.948002

HA 33.716999 34.009998 43.532001

CB 34.606998 35.945999 43.750000

HB 34.365002 36.889000 44.237999

CG1 35.901001 35.405998 44.375999

HG11 36.716999 36.106998 44.194000

HG12 35.790001 35.296001 45.453999

HG13 36.159000 34.442001 43.938000

CG2 34.882000 36.235001 42.265999

HG21 34.028999 36.742001 41.814999

HG22 35.744999 36.894001 42.171001

HG23 35.077999 35.308998 41.727001

C 33.133999 34.779999 45.438000

O 32.584000 35.669998 46.089001

N 33.551998 33.643002 45.998001

H 33.960999 32.918999 45.414001

CA 33.363998 33.299999 47.407001

HA 33.167000 34.219002 47.948002

CB 32.173000 32.330002 47.550999

HB2 32.536999 31.351999 47.233002

HB3 31.917000 32.285999 48.608002

CG 30.889000 32.571999 46.777000

ND1 30.726999 32.459000 45.414001

HD1 31.462999 32.293999 44.737999

CE1 29.431999 32.643002 45.119999

HE1 29.017000 32.639999 44.118999

NE2 28.718000 32.828999 46.242001

CD2 29.639000 32.798000 47.299000

HD2 29.395000 32.886002 48.349998

C 34.631001 32.667999 48.023998

O 35.442001 32.088001 47.298000

N 34.806000 32.714001 49.352001

H 34.153000 33.199001 49.951000

CA 36.005001 32.271000 50.049000

HA 36.533001 31.542000 49.443001

CB 36.904999 33.471001 50.241001

HB2 37.867001 33.148998 50.632000

HB3 37.075001 33.862999 49.247002

CG 36.441002 34.609001 51.113998

CD1 35.937000 35.813999 50.741001

HD1 35.817001 36.172001 49.735001

NE1 35.671001 36.604000 51.844002

HE1 35.398998 37.591000 51.860001

CE2 35.963001 35.932999 52.997002

CZ2 35.895000 36.279999 54.353001

HZ2 35.536999 37.243000 54.681999

CH2 36.464001 35.431999 55.306999

HH2 36.453999 35.743000 56.335999

CZ3 36.944000 34.171001 54.932999

HZ3 37.368999 33.480000 55.647999

CE3 36.875999 33.794998 53.591000

HE3 37.227001 32.817001 53.338001

CD2 36.491001 34.685001 52.569000

C 35.696999 31.535000 51.349998

O 34.708000 31.799000 52.021999

N 36.535999 30.566999 51.712002

H 37.362000 30.430000 51.146999

CA 36.154999 29.478001 52.631001

HA 35.115002 29.235001 52.410999

CB 37.026001 28.216000 52.327000

HB2 36.966999 28.018999 51.262001

HB3 38.049000 28.419001 52.631001

CG 36.693001 26.905001 53.020000

OD1 35.889999 26.902000 53.936001

ND2 37.342999 25.802000 52.681000

HD21 37.279999 24.996000 53.284000

HD22 38.028999 25.813999 51.931999

C 36.258999 29.900000 54.105999

O 37.306000 30.361000 54.561001

N 35.243999 29.690001 54.945999

H 34.389000 29.254999 54.608002

CA 35.234001 30.330000 56.278000

HA 35.584000 31.355000 56.130001

CB 33.821999 30.388000 56.838001

HB 33.867001 30.857000 57.820000

CG2 32.869999 31.193001 55.973000

HG21 31.927999 31.264000 56.526001

HG22 33.313999 32.175999 55.832001

HG23 32.777000 30.743000 54.981998

OG1 33.285000 29.099001 56.966999

HG1 32.612000 29.208000 57.657001

C 36.175999 29.695999 57.320999

O 36.428001 30.264999 58.380001

N 36.782001 28.548000 56.974998

H 36.305000 28.051001 56.243999

CA 37.937000 27.806999 57.526001

HA 37.694000 27.184999 58.384998

CB 38.252998 26.794001 56.416000

HB2 37.338001 26.202000 56.320999

HB3 38.341000 27.337000 55.472000

CG 39.521999 25.915001 56.645000

HG2 40.375000 26.485001 56.290001

HG3 39.666000 25.684999 57.705002

CD 39.480999 24.598000 55.855999

HD2 38.633999 24.041000 56.243000

HD3 39.217999 24.771999 54.820999

CE 40.755001 23.745001 55.882999

HE2 41.638000 24.330999 55.595001

HE3 40.883999 23.427000 56.912998

NZ 40.605999 22.540001 55.023998

HZ1 41.391998 21.914000 55.127998

HZ2 39.728001 22.058001 55.221001

HZ3 40.521999 22.792000 54.042999

C 39.133999 28.691999 57.897999

O 39.834999 28.466999 58.884998

N 39.348999 29.712000 57.091000

H 38.727001 29.733999 56.300999

CA 40.550999 30.544001 57.007999

HA 41.341999 30.080000 57.598000

CB 41.005001 30.483999 55.535999

HB2 40.172001 30.782000 54.888000

HB3 41.811001 31.214001 55.403999

CG 41.455002 29.052999 55.145000

CD1 42.585999 28.475000 55.789001

HD1 43.159000 28.964001 56.570000

CE1 43.049999 27.215000 55.382000

HE1 43.981998 26.830000 55.783001

CZ 42.359001 26.504000 54.380001

OH 42.929001 25.368999 53.931000

HH 42.431999 25.004000 53.179001

CE2 41.167999 26.999001 53.799000

HE2 40.698002 26.459999 52.980999

CD2 40.721001 28.285999 54.194000

HD2 39.846001 28.753000 53.759998

C 40.381001 31.971001 57.578999

O 41.324001 32.743000 57.587002

N 39.187000 32.324001 58.051998

H 38.453999 31.649000 57.903999

CA 38.816002 33.596001 58.689999

HA2 37.841000 33.393002 59.131001

HA3 39.514000 33.833000 59.492001

C 38.765999 34.875999 57.825001

O 38.047001 35.793999 58.234001

N 39.540001 34.990002 56.730000

H 40.141998 34.223000 56.455002

CA 39.820999 36.229000 55.990002

HA 38.929001 36.848000 56.004002

CB 41.001999 36.993999 56.616001

HB2 41.863998 36.334999 56.787998

HB3 41.293999 37.727001 55.875999

CG 40.589001 37.772999 57.875000

OD1 39.730999 38.700001 57.764999

OD2 41.108002 37.433998 58.966000

C 40.077000 35.917999 54.525002

O 40.467999 34.798000 54.195999

N 39.769001 36.847000 53.608002

H 39.363998 37.730999 53.911999

CA 39.919998 36.632000 52.160000

HA 39.376999 35.736000 51.854000

CB 39.318001 37.820000 51.400002

HB2 38.236000 37.814999 51.519001

HB3 39.689999 38.750000 51.832001

CG 39.636002 37.811001 49.922001

CD1 40.494999 38.785999 49.382000

HD1 40.931999 39.541000 50.023998

CE1 40.790001 38.776001 48.008999

HE1 41.452000 39.527000 47.597000

CZ 40.226002 37.792000 47.174999

HZ 40.448002 37.792999 46.116001

CE2 39.374001 36.811001 47.716000

HE2 38.939999 36.056000 47.075001

CD2 39.083000 36.818001 49.091000

HD2 38.431999 36.064999 49.509998

C 41.395000 36.430000 51.791000

O 41.749001 35.445000 51.143002

N 42.265999 37.323002 52.276001

H 41.875000 38.118000 52.761002

CA 43.703999 37.250000 52.051998

HA2 43.825001 37.131001 50.976002

HA3 44.191002 38.167999 52.382000

C 44.403999 36.070999 52.728001

O 45.521000 35.783001 52.306000

N 43.758999 35.345001 53.655998

H 42.778000 35.561001 53.755001

CA 44.202000 34.002998 54.062000

HA 45.300999 33.962002 54.035999

CB 43.735001 33.674999 55.487999

HB2 42.655998 33.747002 55.674000

HB3 43.928001 32.619999 55.622002

CG 44.601002 34.446999 56.501999

HG2 45.638000 34.146000 56.407001

HG3 44.561001 35.519001 56.282001

CD 44.212002 34.130001 57.939999

HD2 44.861000 34.727001 58.582001

HD3 43.171001 34.451000 58.000000

CE 44.388000 32.625000 58.312000

HE2 43.816002 32.008999 57.618000

HE3 45.426998 32.299999 58.202000

NZ 43.931000 32.265999 59.653000

HZ1 42.928001 32.388000 59.757000

HZ2 44.123001 31.275000 59.686001

HZ3 44.445999 32.680000 60.423000

C 43.669998 32.957001 53.096001

O 44.476002 32.314999 52.423000

N 42.341999 32.848000 52.941002

H 41.736000 33.337002 53.595001

CA 41.695999 31.917000 52.006001

HA 41.762001 30.936001 52.487999

CB 40.206001 32.306999 51.911999

HB1 39.896000 32.681999 52.883999

HB2 40.054001 33.122002 51.205002

HB3 39.571999 31.462999 51.625999

C 42.404999 31.878000 50.598999

O 42.800999 30.816999 50.119999

N 42.625999 33.019001 49.932999

H 42.328999 33.890999 50.367001

CA 43.201000 33.063999 48.567001

HA 42.543999 32.471001 47.930000

CB 43.191002 34.500999 48.001999

HB 42.200001 34.926998 48.168999

CG1 44.231998 35.395000 48.681000

HG11 44.116001 36.423000 48.340000

HG12 44.094002 35.361000 49.759998

HG13 45.240002 35.057999 48.446999

CG2 43.466000 34.522999 46.492001

HG21 42.859001 33.770000 45.993000

HG22 43.212002 35.505001 46.091999

HG23 44.516998 34.312000 46.293999

C 44.591999 32.418999 48.445000

O 44.959999 31.976999 47.359001

N 45.348999 32.277000 49.542000

H 44.971001 32.557999 50.439999

CA 46.620998 31.539000 49.591000

HA 47.160000 31.743999 48.673000

CB 47.478001 32.109001 50.744999

HB2 46.999001 31.783001 51.668999

HB3 48.491001 31.723000 50.674000

CG 47.630001 33.630001 50.847000

HG2 46.623001 34.009998 50.894001

HG3 48.105999 33.884998 51.799999

CD 48.298000 34.346001 49.669998

OE1 48.382000 33.872002 48.549999

NE2 48.783001 35.551998 49.846001

HE21 49.205002 36.049000 49.067001

HE22 48.693001 35.967999 50.763000

C 46.452999 29.986000 49.658001

O 47.446999 29.275999 49.861000

N 45.230999 29.424000 49.530998

H 44.445999 30.007000 49.275002

CA 44.855999 28.098000 50.041000

HA 45.787998 27.681999 50.396999

CB 43.992001 28.167000 51.324001

HB2 42.988998 28.598000 51.209000

HB3 43.799999 27.138000 51.598999

CG 44.805000 28.896999 52.432999

HG2 45.171001 29.830999 52.001999

HG3 44.167999 29.230000 53.244999

CD 45.949001 28.113001 53.125000

OE1 46.056000 26.888000 53.160999

NE2 46.911999 28.847000 53.636002

HE21 47.653000 28.437000 54.182999

HE22 46.911999 29.846001 53.512001

C 44.273998 27.164000 48.948002

O 43.570000 27.663000 48.070999

N 44.569000 25.841999 48.966000

CD 45.530998 25.187000 49.831001

HD2 45.098000 25.075001 50.821999

HD3 46.411999 25.808001 49.946999

CG 45.868000 23.823000 49.217999

HG2 45.763000 23.082001 50.006001

HG3 46.881001 23.781000 48.799999

CB 44.764999 23.559999 48.178001

HB2 44.094002 22.775999 48.530998

HB3 45.101002 23.337000 47.167000

CA 44.035000 24.881001 48.013000

HA 44.275002 25.235001 47.018002

C 42.522999 24.745001 48.019001

O 41.952999 24.781000 46.938999

N 41.846001 24.704000 49.173000

H 42.393002 24.813999 50.002998

CA 40.403999 24.940001 49.391998

HA 39.909000 24.799000 48.443001

CB 39.653000 23.940001 50.334000

HB2 38.594002 24.233999 50.376999

HB3 39.709000 22.907000 49.980000

CG 40.198002 23.860001 51.754002

OD1 41.327999 24.284000 52.053001

OD2 39.508999 23.319000 52.632999

C 40.154999 26.416000 49.762001

O 39.284000 26.742001 50.568001

N 40.876999 27.312000 49.094002

H 41.555000 26.973000 48.425999

CA 40.803001 28.754000 49.243000

HA2 40.973000 29.010000 50.285000

HA3 41.577000 29.216000 48.618000

C 39.441002 29.313999 48.879002

O 38.757999 29.885000 49.734001

N 39.082001 29.219999 47.596001

H 39.691002 28.694000 46.973999

CA 37.977001 29.950001 46.964001

HA 37.405998 30.466000 47.730999

CB 38.516998 31.009001 45.980999

HB2 38.903000 30.481001 45.110001

HB3 37.669998 31.605000 45.646999

CG 39.612999 31.975000 46.459999

HG 40.522999 31.419001 46.682999

CD1 39.897999 32.953999 45.323002

HD11 40.235001 32.403999 44.443001

HD12 38.995998 33.518002 45.073002

HD13 40.673000 33.653000 45.625000

CD2 39.216000 32.801998 47.680000

HD21 38.312000 33.366001 47.453999

HD22 39.040001 32.146999 48.532001

HD23 40.021000 33.488998 47.935001

C 37.016998 29.031000 46.193001

O 37.372002 27.917000 45.793999

N 35.820999 29.542000 45.904999

H 35.597000 30.466000 46.264000

CA 34.911999 28.982000 44.910999

HA 35.490002 28.354000 44.235001

CB 33.862000 28.097000 45.598000

HB1 33.271000 28.688999 46.299000

HB2 33.201000 27.660999 44.847000

HB3 34.360001 27.290001 46.138000

C 34.279999 30.104000 44.069000

O 33.910999 31.156000 44.598000

N 34.157001 29.867001 42.761002

H 34.432999 28.954000 42.409000

CA 33.570999 30.820000 41.808998

HA 33.203999 31.681999 42.362000

CB 34.603001 31.364000 40.801998

HB 35.025002 30.538000 40.229000

CG1 33.952999 32.368000 39.838001

HG11 34.713001 32.787998 39.179001

HG12 33.208000 31.871000 39.217999

HG13 33.478001 33.174000 40.397999

CG2 35.740002 32.105000 41.521999

HG21 36.458000 32.477001 40.790001

HG22 35.338001 32.943001 42.089001

HG23 36.261002 31.437000 42.202999

C 32.368999 30.186001 41.118999

O 32.466999 29.107000 40.528000

N 31.229000 30.867001 41.222000

H 31.254999 31.766001 41.692001

CA 29.969000 30.479000 40.599998

HA 29.993000 29.406000 40.438000

CB 28.837999 30.773001 41.602001

HB2 29.186001 30.471001 42.591000

HB3 28.645000 31.843000 41.627998

CG 27.523001 30.021000 41.335999

HG 27.731001 28.958000 41.206001

CD1 26.598000 30.183001 42.542999

HD11 27.087999 29.805000 43.438999

HD12 26.344999 31.233999 42.686001

HD13 25.681999 29.612000 42.387001

CD2 26.781000 30.540001 40.109001

HD21 27.275000 30.208000 39.199001

HD22 25.775000 30.136000 40.105000

HD23 26.728001 31.627001 40.122002

C 29.837000 31.198000 39.245998

O 29.903999 32.426998 39.207001

N 29.697001 30.448000 38.150002

H 29.613001 29.445999 38.280998

CA 29.650999 30.952999 36.771999

HA2 29.927999 32.000999 36.765999

HA3 30.386999 30.416000 36.176998

C 28.285999 30.799999 36.090000

O 27.598000 29.799000 36.300999

N 27.884001 31.789000 35.279999

H 28.495001 32.594002 35.182999

CA 26.572001 31.851999 34.604000

HA 26.194000 30.834000 34.495998

CB 25.542000 32.660999 35.445000

HB 25.774000 33.719002 35.332001

CG2 24.125000 32.436001 34.884998

HG21 24.086000 32.671001 33.823002

HG22 23.820000 31.399000 35.032001

HG23 23.419001 33.096001 35.383999

CG1 25.596001 32.346001 36.963001

HG12 25.481001 31.274000 37.105999

HG13 26.575001 32.633999 37.347000

CD1 24.562000 33.070000 37.838001

HD11 23.554001 32.734001 37.603001

HD12 24.761999 32.849998 38.886002

HD13 24.631001 34.146000 37.685001

C 26.716999 32.452000 33.188999

O 27.351999 33.495998 33.028000

N 26.127001 31.822001 32.167000

H 25.594000 30.981001 32.374001

CA 26.084999 32.313999 30.775000

HA 26.983999 32.901001 30.580000

CB 26.083000 31.118000 29.813999

HB2 25.179001 30.531000 29.986000

HB3 26.033001 31.492001 28.792000

CG 27.295000 30.211000 29.923000

CD1 27.179001 28.934999 30.506001

HD1 26.232000 28.606001 30.907000

CE1 28.295000 28.080999 30.565001

HE1 28.197001 27.097000 31.000999

CZ 29.532000 28.504000 30.049000

HZ 30.384001 27.841000 30.084000

CE2 29.656000 29.785999 29.485001

HE2 30.607000 30.115999 29.091999

CD2 28.540001 30.638000 29.422001

HD2 28.635000 31.618000 28.979000

C 24.868999 33.223000 30.500000

O 23.872999 33.159000 31.225000

N 24.923000 34.070999 29.455999

H 25.771000 34.131001 28.906000

CA 23.900000 35.104000 29.240999

HA 23.062000 34.910000 29.907000

CB 24.472000 36.477001 29.628000

HB2 25.417999 36.636002 29.108000

HB3 23.766001 37.226002 29.274000

CG 24.656000 36.659000 31.148001

HG 23.941999 36.030998 31.677999

CD1 26.059999 36.319000 31.638000

HD11 26.327999 35.306999 31.351000

HD12 26.788000 37.013000 31.226000

HD13 26.072001 36.375000 32.722000

CD2 24.403999 38.110001 31.546000

HD21 23.372999 38.367001 31.311001

HD22 24.549999 38.228001 32.618999

HD23 25.080999 38.772999 31.006001

C 23.330999 35.127998 27.816000

O 24.091999 35.085999 26.843000

N 22.004000 35.233002 27.680000

H 21.436001 35.217999 28.524000

CA 21.306000 35.396000 26.407000

HA 21.950001 35.129002 25.573000

CB 19.990000 34.609001 26.375999

HB2 19.356001 35.105999 27.103001

HB3 19.500999 34.698002 25.403999

CG 19.980000 33.144001 26.768000

HG2 20.318001 33.026001 27.796000

HG3 18.933001 32.853001 26.770000

CD 20.768000 32.200001 25.847000

HD2 21.743999 32.615002 25.622000

HD3 20.924000 31.260000 26.388000

CE 20.076000 31.962999 24.481001

HE2 19.921000 32.904999 23.931000

HE3 20.652000 31.302999 23.836000

NZ 18.846001 31.198999 24.705999

HZ1 19.040001 30.452999 25.372000

HZ2 18.128000 31.820000 25.049000

HZ3 18.433001 30.812000 23.864000

C 20.931000 36.844002 26.190001

O 20.422001 37.490002 27.108000

N 21.104000 37.319000 24.965000

H 21.568001 36.717999 24.295000

CA 20.537001 38.611000 24.524000

HA 20.825001 39.375999 25.243999

CB 21.075001 39.035999 23.131001

HB 20.811001 38.279999 22.389999

CG1 20.511999 40.394001 22.632000

HG11 20.955999 40.669998 21.676001

HG12 19.434999 40.356998 22.434000

HG13 20.790001 41.166000 23.350000

CG2 22.614000 39.144001 23.143999

HG21 22.945000 39.853001 23.906000

HG22 23.059999 38.176998 23.381001

HG23 22.926001 39.442001 22.139999

C 19.002001 38.548000 24.549000

O 18.375999 37.929001 23.688999

N 18.416000 39.126999 25.597000

H 19.031000 39.658001 26.200001

CA 17.063999 38.875000 26.115999

HA2 16.399000 38.569000 25.318001

HA3 17.201000 38.125000 26.898001

C 16.355000 40.046001 26.767000

O 16.995001 41.037998 27.099001

N 15.080000 39.855000 27.127001

H 14.595000 39.032001 26.789000

CA 14.369000 40.705002 28.097000

HA 14.458000 41.754002 27.816999

CB 12.884000 40.388000 28.018999

HB2 12.485000 40.528999 27.013000

HB3 12.702000 39.367001 28.327999

OG 12.105000 41.160000 28.882000

HG 11.210000 41.071999 28.511999

C 14.913000 40.481998 29.518999

O 14.902000 39.372002 30.039000

N 15.512000 41.499001 30.120001

H 15.480000 42.396000 29.666000

CA 16.509001 41.331001 31.171000

HA 17.077000 40.445000 30.905001

CB 17.427000 42.548000 31.138000

HB1 16.833000 43.465000 31.084999

HB2 18.020000 42.591000 32.049000

HB3 18.089001 42.408001 30.284000

C 15.995000 41.030998 32.587002

O 14.843000 41.308998 32.911999

N 16.871000 40.477001 33.437000

H 17.805000 40.286999 33.094002

CA 16.594999 40.187000 34.859001

HA 15.599000 39.723999 34.833000

CB 17.702999 39.264000 35.411999

HB2 18.684000 39.709999 35.231998

HB3 17.643999 39.198002 36.505001

CG 17.645000 37.903999 34.667999

HG2 16.694000 37.745998 34.136002

HG3 18.445000 37.938999 33.931999

CD 17.823000 36.709000 35.605999

HD2 17.849001 35.799999 35.004002

HD3 18.730000 36.737999 36.223000

CE 16.517000 36.747002 36.418999

HE2 16.458000 37.615002 37.084999

HE3 15.699000 36.834000 35.695999

NZ 16.403999 35.558998 37.273998

HZ1 17.236000 35.467999 37.853001

HZ2 15.630000 35.646999 37.916000

HZ3 16.208000 34.714001 36.754002

C 16.594000 41.458000 35.731998

O 17.389999 42.361000 35.463001

N 15.772000 41.528999 36.796001

CD 14.912000 40.465000 37.314999

HD2 15.267000 39.462002 37.055000

HD3 13.914000 40.683998 36.936001

CG 14.929000 40.657001 38.832001

HG2 15.874000 40.270000 39.217999

HG3 14.070000 40.209999 39.333000

CB 14.887000 42.175999 38.897999

HB2 15.208000 42.549000 39.868000

HB3 13.877000 42.506001 38.653999

CA 15.875000 42.595001 37.799000

HA 15.579000 43.533001 37.330002

C 17.289000 42.737000 38.410999

O 18.037001 41.764000 38.414001

N 17.688000 43.931000 38.879002

H 17.052999 44.716000 38.868000

CA 19.047001 44.304001 39.279999

HA2 18.980000 45.221001 39.863998

HA3 19.431000 43.514999 39.929001

C 20.054001 44.605000 38.133999

O 21.009001 45.358002 38.314999

N 19.823999 44.127998 36.909000

H 18.929001 43.669998 36.813000

CA 20.757999 44.402000 35.784000

HA 21.787001 44.286999 36.134998

CB 20.579000 43.491001 34.561001

HB2 19.552999 43.490002 34.240002

HB3 21.061001 44.007000 33.721001

CG 20.948000 41.980000 34.698002

HG 20.176001 41.396000 35.194000

CD1 21.028000 41.375999 33.312000

HD11 20.018999 41.215000 32.932999

HD12 21.566000 42.073002 32.680000

HD13 21.551001 40.421001 33.320999

CD2 22.275999 41.740002 35.382000

HD21 22.541000 40.679001 35.425999

HD22 23.024000 42.320000 34.859001

HD23 22.247999 42.159000 36.380001

C 20.719999 45.893002 35.368999

O 21.750999 46.549999 35.383999

N 19.561001 46.507000 35.092999

H 18.715000 45.959000 35.041000

CA 19.469000 47.917999 34.702999

HA 20.125999 48.026001 33.845001

CB 18.056000 48.283001 34.222000

HB2 17.695000 47.493999 33.558998

HB3 17.403999 48.359001 35.092999

CG 17.988001 49.664001 33.516998

HG2 16.934000 49.909000 33.346001

HG3 18.372000 50.404999 34.216000

CD 18.757000 49.772999 32.188999

OE1 18.634001 48.929001 31.295000

NE2 19.429001 50.884998 31.969000

HE21 19.625999 51.143002 31.016001

HE22 19.601999 51.577999 32.696999

C 19.943001 48.887001 35.807999

O 20.579000 49.895000 35.500000

N 19.737000 48.592999 37.099998

H 19.068001 47.866001 37.330002

CA 20.302999 49.415001 38.189999

HA 20.111000 50.469002 37.951000

CB 19.716000 49.056999 39.563999

HB2 18.652000 48.875999 39.409000

HB3 20.238001 48.174999 39.957001

CG 19.903999 50.257000 40.527000

HG2 20.959999 50.487000 40.659000

HG3 19.400000 51.111000 40.090000

CD 19.370001 50.015999 41.908001

HD2 18.396999 49.532001 41.819000

HD3 20.104000 49.348999 42.342999

CE 19.330000 51.243999 42.834000

HE2 20.287001 51.766998 42.761002

HE3 18.554001 51.951000 42.540001

NZ 19.087999 50.824001 44.223000

HZ1 19.879000 50.255001 44.513000

HZ2 18.971001 51.660000 44.789001

HZ3 18.263000 50.227001 44.275002

C 21.805000 49.257000 38.293999

O 22.424000 50.223000 38.698002

N 22.410000 48.111000 37.955002

H 21.827999 47.323002 37.686001

CA 23.885000 47.964001 37.867001

HA 24.340000 48.459999 38.723999

CB 24.288000 46.469002 37.901001

HB 23.530001 45.879002 37.386002

CG1 25.648001 46.130001 37.272999

HG11 25.863001 45.071999 37.409000

HG12 25.634001 46.334000 36.202000

HG13 26.434000 46.723999 37.742001

CG2 24.379999 46.000999 39.355999

HG21 25.160000 46.556000 39.882000

HG22 23.427999 46.171001 39.848000

HG23 24.603001 44.933998 39.394001

C 24.468000 48.675999 36.644001

O 25.558001 49.230999 36.728001

N 23.740999 48.699001 35.525002

H 22.836000 48.241001 35.562000

CA 24.128000 49.445999 34.293999

HA 25.150999 49.198002 34.008999

CB 23.231001 49.054001 33.084000

HB 22.174999 49.026001 33.362000

CG1 23.368000 50.067001 31.931000

HG11 24.424999 50.250999 31.737000

HG12 22.802999 49.813000 31.033001

HG13 22.954000 51.037998 32.193001

CG2 23.617001 47.637001 32.603001

HG21 22.737000 47.237999 32.104000

HG22 24.500000 47.648998 31.961000

HG23 23.871000 46.999001 33.451000

C 24.108000 50.971001 34.502998

O 24.972000 51.688999 33.993000

N 23.073999 51.519001 35.167000

H 22.320000 50.919998 35.491001

CA 22.778000 52.971001 35.127998

HA 22.687000 53.273998 34.083000

CB 21.409000 53.226002 35.820000

HB2 21.289000 52.636002 36.735001

HB3 21.379000 54.290001 36.046001

CG 20.152000 52.990002 34.969002

OD1 20.193001 53.185001 33.737000

OD2 19.034000 52.813000 35.514999

C 23.892000 53.862999 35.712002

O 24.160000 54.936001 35.176998

N 24.525999 53.424999 36.793999

H 24.214001 52.553001 37.199001

CA 25.576000 54.127998 37.522999

HA 25.275000 55.174999 37.584999

CB 25.549999 53.469002 38.925999

HB 24.493000 53.471001 39.233002

CG1 26.082001 52.011002 38.886002

HG11 26.915001 51.863998 38.188000

HG12 26.332001 51.745998 39.909000

HG13 25.332001 51.278000 38.606998

CG2 26.433001 54.112999 40.026001

HG21 26.239000 53.590000 40.965000

HG22 27.481001 53.969002 39.771000

HG23 26.271000 55.176998 40.188000

C 26.975000 54.106998 36.842999

O 27.924000 54.706001 37.344002

N 27.160000 53.369999 35.730000

H 26.341000 52.928001 35.326000

CA 28.487000 52.932999 35.233002

HA 28.999001 52.470001 36.076000

CB 28.327000 51.852001 34.139000

HB2 27.629000 52.221001 33.388000

HB3 29.285999 51.710999 33.643002

CG 27.864000 50.463001 34.617001

HG 26.920000 50.570000 35.136002

CD1 27.687000 49.527000 33.416000

HD11 27.318001 48.558998 33.755001

HD12 26.976000 49.956001 32.710999

HD13 28.639000 49.374001 32.914001

CD2 28.864000 49.792000 35.557999

HD21 28.927000 50.352001 36.488998

HD22 28.516001 48.787998 35.797001

HD23 29.841000 49.733002 35.082001

C 29.474001 54.018002 34.738998

O 30.676001 53.745998 34.715000

N 29.070000 55.224998 34.340000

H 28.075001 55.424999 34.286999

CA 30.020000 56.299999 33.973999

HA 30.802000 55.827999 33.370998

CB 29.313999 57.397999 33.120998

HB2 28.504999 57.858002 33.683998

HB3 29.985001 58.243999 32.974998

CG 28.740000 56.904999 31.780001

OD1 29.120001 55.796001 31.327999

OD2 27.865000 57.625000 31.222000

C 30.764000 56.919998 35.180000

O 31.924000 57.314999 35.046001

N 30.198000 56.882999 36.388000

H 29.237000 56.576000 36.438000

CA 30.982000 57.022999 37.639999

HA 31.358000 58.050999 37.676998

CB 30.094999 56.848999 38.887001

HB2 30.665001 57.076000 39.787998

HB3 29.180000 57.445999 38.806999

OG 29.733000 55.511002 39.115002

HG 28.992001 55.284000 38.528999

C 32.191002 56.040001 37.720001

O 33.137001 56.341999 38.438999

N 32.257000 54.952000 36.937000

H 31.471001 54.730999 36.340000

CA 33.389000 54.002998 36.945000

HA 34.191002 54.467999 37.508999

CB 33.027000 52.722000 37.733002

HB 33.945000 52.144001 37.827000

CG2 32.557999 53.047001 39.164001

HG21 33.202999 53.806000 39.609001

HG22 31.534000 53.423000 39.145000

HG23 32.595001 52.152000 39.785999

CG1 31.988001 51.841000 37.014999

HG12 31.006001 52.310001 37.077999

HG13 32.254002 51.743999 35.966999

CD1 31.929001 50.424000 37.584999

HD11 32.887001 49.937000 37.426998

HD12 31.702999 50.431000 38.648998

HD13 31.162001 49.862999 37.062000

C 34.042000 53.748001 35.566002

O 34.632999 52.692001 35.347000

N 33.973999 54.691002 34.612000

H 33.433998 55.529999 34.806999

CA 34.424000 54.457001 33.220001

HA 33.817001 53.661999 32.800999

CB 34.081001 55.716999 32.437000

HB2 33.166000 56.134998 32.868999

HB3 34.901001 56.433998 32.486000

CG 33.806999 55.377998 30.962999

HG2 34.648998 54.883999 30.480000

HG3 32.999001 54.657001 30.950001

CD 33.412998 56.577999 30.094999

HD2 32.506001 57.042000 30.485001

HD3 34.278999 57.237999 30.030001

CE 33.027000 56.125000 28.705000

HE2 32.367001 55.271000 28.853001

HE3 32.412998 56.900002 28.229000

NZ 34.223999 55.709000 27.910000

HZ1 34.002998 54.914001 27.316999

HZ2 34.639999 56.409000 27.312000

HZ3 35.030998 55.445000 28.464001

C 35.889000 54.042999 33.019001

O 36.193001 53.126999 32.247002

N 36.743999 54.742001 33.745998

H 36.365002 55.459000 34.347000

CA 38.185001 54.548000 33.783001

HA 38.483002 53.980999 32.901001

CB 38.896000 55.903999 33.705002

HB 39.967999 55.734001 33.777000

CG2 38.629002 56.620998 32.389000

HG21 39.214001 57.536999 32.362000

HG22 38.932999 55.980999 31.559999

HG23 37.573002 56.870998 32.294998

OG1 38.495998 56.757000 34.751999

HG1 39.178001 56.617001 35.445000

C 38.598999 53.748001 35.022999

O 37.905998 53.730999 36.046001

N 39.734001 53.051998 34.929001

H 40.255001 53.154999 34.063000

CA 40.282001 52.145000 35.949001

HA 39.604000 51.297001 36.055000

CB 41.640999 51.639000 35.430000

HB2 41.455002 50.994999 34.571999

HB3 42.235001 52.492001 35.098000

CG 42.464001 50.863998 36.472000

HG2 42.764999 51.530998 37.279999

HG3 41.861000 50.063999 36.890999

CD 43.723999 50.275002 35.835999

HD2 43.457001 49.681999 34.962002

HD3 44.384998 51.084999 35.521999

CE 44.446999 49.381001 36.841000

HE2 44.737000 49.976002 37.714001

HE3 43.764000 48.596001 37.181000

NZ 45.639999 48.764000 36.223000

HZ1 46.226002 48.334999 36.931999

HZ2 45.368000 48.063000 35.535000

HZ3 46.214001 49.463001 35.758999

C 40.432999 52.813999 37.319000

O 41.025002 53.887001 37.436001

N 39.979000 52.132999 38.370998

H 39.465000 51.278000 38.209999

CA 40.249001 52.490002 39.769001

HA2 40.327999 51.568001 40.338001

HA3 41.202000 53.005001 39.820000

C 39.209000 53.374001 40.462002

O 39.425999 53.808998 41.598000

N 38.095001 53.652000 39.779999

H 38.014000 53.265999 38.849998

CA 36.944000 54.391998 40.313000

HA 37.293999 55.092999 41.067001

CB 36.294998 55.199001 39.186001

HB2 36.148998 54.556999 38.318001

HB3 35.328999 55.563999 39.533001

CG 37.148998 56.410999 38.801998

HG2 37.194000 57.084000 39.660000

HG3 38.161999 56.099998 38.537998

CD 36.514999 57.150002 37.624001

HD2 36.506001 56.506001 36.741001

HD3 35.488998 57.419998 37.882000

CE 37.298000 58.428001 37.333000

HE2 37.386002 59.006001 38.256001

HE3 38.306000 58.173000 36.988998

NZ 36.598999 59.243000 36.321999

HZ1 36.356998 58.687000 35.508999

HZ2 35.728001 59.598000 36.713001

HZ3 37.188000 60.033001 36.053001

C 35.931999 53.467999 40.991001

O 35.700001 52.349998 40.525002

N 35.314999 53.937000 42.075001

H 35.535999 54.881001 42.386002

CA 34.283001 53.205002 42.819000

HA 33.689999 52.650002 42.095001

CB 34.917000 52.196999 43.786999

HB2 34.130001 51.541000 44.166000

HB3 35.654999 51.601002 43.249001

OG 35.556999 52.806000 44.891998

HG 36.457001 53.088001 44.624001

C 33.319000 54.134998 43.561001

O 33.695000 55.228001 43.991001

N 32.077999 53.676998 43.727001

H 31.875000 52.744999 43.376999

CA 30.951000 54.419998 44.301998

HA 31.327999 55.243000 44.907001

CB 30.117001 54.997002 43.145000

HB1 29.747000 54.188999 42.508999

HB2 29.266001 55.547001 43.548000

HB3 30.724001 55.669998 42.537998

C 30.091000 53.511002 45.199001

O 29.875999 52.347000 44.855999

N 29.568001 54.007999 46.327000

H 29.707001 54.964001 46.631001

CA 28.664000 53.250000 47.188999

HA 29.183001 52.332001 47.488998

CB 28.379999 54.075001 48.446999

HB2 27.642000 53.535999 49.042000

HB3 29.302999 54.153000 49.018002

CG 27.908001 55.497002 48.166000

OD1 26.683001 55.750000 48.234001

OD2 28.747999 56.398998 47.939999

C 27.334999 52.928001 46.474998

O 26.808001 53.738998 45.701000

N 26.875999 51.695999 46.671001

H 27.371000 51.125999 47.352001

CA 25.849001 51.040001 45.858002

HA 25.101000 51.773998 45.550999

CB 26.518999 50.460999 44.598999

HB2 27.035000 51.271000 44.084999

HB3 27.275000 49.733002 44.894001

CG 25.573000 49.819000 43.604000

CD1 24.857000 50.623001 42.698002

HD1 24.989000 51.695000 42.705002

CE1 23.981001 50.034000 41.771000

HE1 23.434999 50.654999 41.075001

CZ 23.833000 48.639000 41.733002

HZ 23.177999 48.189999 41.004002

CE2 24.556999 47.832001 42.625999

HE2 24.455999 46.757000 42.589001

CD2 25.427000 48.421001 43.556999

HD2 25.989000 47.798000 44.235001

C 25.150000 49.994999 46.731998

O 25.378000 48.782001 46.636002

N 24.416000 50.493000 47.726002

H 24.259001 51.491001 47.793999

CA 23.964001 49.673000 48.835999

HA 24.782000 48.978001 49.039001

CB 23.837000 50.507000 50.098999

HB 23.540001 49.807999 50.877998

CG2 25.184999 51.144001 50.494999

HG21 25.132999 51.702000 51.428001

HG22 26.002001 50.424999 50.594002

HG23 25.465000 51.847000 49.721001

OG1 22.860001 51.519001 49.856998

HG1 22.914000 52.155998 50.580002

C 22.660999 48.896999 48.541000

O 21.830000 49.217999 47.667000

N 22.542000 47.806000 49.286999

H 23.353001 47.484001 49.797001

CA 21.351000 46.974998 49.366001

HA 21.620001 46.171001 50.054001

CB 20.233999 47.763000 50.055000

HB2 19.832001 48.525002 49.393002

HB3 19.399000 47.082001 50.176998

CG 20.596001 48.439999 51.375000

OD1 20.542999 49.657001 51.504002

ND2 20.903000 47.715000 52.424999

HD21 20.997999 48.203999 53.297001

HD22 20.902000 46.703999 52.387001

C 20.895000 46.367001 48.008999

O 19.701000 46.345001 47.681000

N 21.850000 45.945000 47.167000

H 22.787001 45.919998 47.539001

CA 21.656000 45.252998 45.876999

HA 20.719999 45.596001 45.430000

CB 22.816999 45.590000 44.916000

HB2 23.035999 46.660000 44.902000

HB3 23.768000 45.220001 45.299000

CG 22.530001 45.019001 43.521999

CD1 21.797001 45.784000 42.596001

HD1 21.483000 46.778000 42.861000

CE1 21.471001 45.291000 41.328999

HE1 21.032000 45.988998 40.617001

CZ 21.898001 43.980000 40.993000

HZ 21.795000 43.665001 39.965000

CE2 22.528999 43.139999 41.962002

HE2 22.849001 42.112999 41.785999

CD2 22.851000 43.687000 43.210999

HD2 23.322001 43.089001 43.973000

C 21.554001 43.717999 46.105000

O 22.521000 43.112000 46.550999

N 20.370001 43.109001 45.924000

H 19.615000 43.693001 45.596001

CA 20.083000 41.653999 46.040001

HA 20.340000 41.368000 47.056999

CB 18.577999 41.394001 45.928001

HB2 18.061001 41.894001 46.736000

HB3 18.226999 41.805000 44.986000

CG 18.160999 39.928001 46.063999

OD1 18.933001 39.146000 46.643002

OD2 17.027000 39.540001 45.722000

C 20.740999 40.719002 44.979000

O 20.381001 40.754002 43.799000

N 21.635000 39.797001 45.376999

CD 22.193001 39.692001 46.716000

HD2 21.648001 38.957001 47.299000

HD3 22.197001 40.633999 47.258999

CG 23.568001 39.084000 46.549999

HG2 23.950001 38.637001 47.466999

HG3 24.302000 39.792999 46.174000

CB 23.224001 38.063000 45.480000

HB2 22.646000 37.224998 45.891998

HB3 24.171000 37.780998 45.028999

CA 22.284000 38.818001 44.504002

HA 22.844000 39.355999 43.737999

C 21.344999 37.838001 43.765999

O 21.695000 37.354000 42.691002

N 20.158001 37.548000 44.311001

H 19.934999 38.091999 45.145000

CA 19.273001 36.430000 43.875000

HA 19.874001 35.536999 43.654999

CB 18.302000 36.146999 45.035999

HB2 17.660999 37.029999 45.071999

HB3 17.684000 35.282001 44.789001

CG 18.987000 35.952000 46.424999

HG2 19.528999 35.014000 46.500999

HG3 19.757999 36.698002 46.582001

CD 17.997999 35.970001 47.573002

HD2 17.423000 35.062000 47.422001

HD3 18.542000 36.020000 48.522999

NE 17.066999 37.081001 47.490002

HE 17.156000 37.750000 46.736000

CZ 16.111000 37.393002 48.320000

NH1 15.866000 36.748001 49.414001

HH11 16.478001 36.018002 49.747002

HH12 15.102000 36.986000 50.036999

NH2 15.398000 38.436001 48.075001

HH21 15.534000 38.884998 47.181999

HH22 14.747000 38.799000 48.756001

C 18.462999 36.782001 42.596001

O 17.950001 35.904999 41.903999

N 18.305000 38.062000 42.230000

H 18.612000 38.764000 42.889999

CA 17.660999 38.425999 40.933998

HA2 16.729000 37.875999 40.773998

HA3 17.466999 39.493000 40.853001

C 18.546000 38.108002 39.743000

O 18.049000 37.889999 38.644001

N 19.867001 38.006001 39.956001

H 20.211000 38.213001 40.884998

CA 20.848000 37.584999 38.944000

HA 20.488001 37.886002 37.958000

CB 22.193001 38.297001 39.209000

HB2 22.361000 38.382999 40.282001

HB3 22.990999 37.674000 38.805000

CG 22.319000 39.681000 38.542000

HG 22.233999 39.547001 37.464001

CD1 21.260000 40.686001 38.993000

HD11 20.281000 40.376999 38.627998

HD12 21.237000 40.750000 40.082001

HD13 21.471001 41.667999 38.577999

CD2 23.695999 40.276001 38.839001

HD21 23.818001 40.422001 39.911999

HD22 24.475000 39.605000 38.476002

HD23 23.798000 41.234001 38.332001

C 21.025999 36.055000 38.869999

O 21.677000 35.571999 37.945999

N 20.455000 35.278000 39.799999

H 19.882000 35.719002 40.506001

CA 20.405001 33.814999 39.678001

HA 21.364000 33.486000 39.282001

CB 20.193001 33.159000 41.053001

HB2 19.263000 33.532001 41.478001

HB3 20.083000 32.081001 40.907001

CG 21.309999 33.380001 42.082001

HG 21.361000 34.431999 42.353001

CD1 20.955999 32.573002 43.333000

HD11 19.962999 32.841999 43.688999

HD12 20.976000 31.507000 43.112999

HD13 21.667999 32.782001 44.125999

CD2 22.677000 32.931000 41.556000

HD21 22.591999 31.959999 41.068001

HD22 23.045000 33.669998 40.844002

HD23 23.392000 32.861000 42.373001

C 19.284000 33.368000 38.712002

O 18.284000 34.081001 38.597000

N 19.379000 32.182999 38.080002

CD 20.548000 31.327999 38.028000

HD2 20.441000 30.546000 38.778999

HD3 21.497000 31.827000 38.181000

CG 20.586000 30.728001 36.646000

HG2 21.193001 29.819000 36.674000

HG3 20.938000 31.434000 35.895000

CB 19.106001 30.539000 36.320999

HB2 18.827000 29.513000 36.574001

HB3 18.959999 30.740999 35.256001

CA 18.364000 31.589001 37.216000

HA 17.893000 32.384998 36.633999

C 17.264000 30.931999 38.073002

O 17.266001 31.098000 39.292999

N 16.278999 30.249001 37.480000

H 16.368999 30.099001 36.472000

CA 15.267000 29.459999 38.243999

HA 14.898000 30.035999 39.102001

CB 14.049000 29.181999 37.314999

HB2 13.654000 30.139999 36.993000

HB3 14.367000 28.688999 36.400002

CG 12.864000 28.372999 37.905998

HG2 13.172000 27.341999 38.083000

HG3 12.566000 28.877001 38.831001

CD 11.629000 28.207001 36.985001

OE1 11.308000 29.048000 36.106998

OE2 10.889000 27.216000 37.202999

C 15.873000 28.156000 38.792000

O 15.745000 27.864000 39.980999

N 16.549999 27.375000 37.937000

H 16.552999 27.666000 36.972000

CA 17.271999 26.129999 38.264000

HA 16.712000 25.563999 38.998001

CB 17.511000 25.228001 37.043999

HB2 16.563999 24.966000 36.571999

HB3 18.091999 25.860001 36.367001

OG 18.198000 24.033001 37.457001

HG 17.527000 23.400000 37.783001

C 18.639000 26.415001 38.840000

O 19.292999 27.372000 38.417000

N 19.106001 25.554001 39.757000

H 18.587999 24.711000 39.979000

CA 20.431000 25.660999 40.362999

HA 20.877001 26.542000 39.926998

CB 20.320999 25.812000 41.887001

HB2 19.573999 25.094999 42.229000

HB3 21.268000 25.499001 42.323002

CG 19.997999 27.229000 42.417999

HG 18.962999 27.473000 42.191002

CD1 20.080999 27.298000 43.939999

HD11 19.749001 28.280001 44.294998

HD12 19.405001 26.535999 44.324001

HD13 21.114000 27.122999 44.243999

CD2 21.007999 28.288000 41.925999

HD21 20.864000 29.197001 42.504002

HD22 22.013000 27.875000 42.056000

HD23 20.863001 28.597000 40.889999

C 21.417999 24.563999 39.994999

O 22.490999 24.419001 40.574001

N 21.028000 23.753000 39.035000

H 20.080000 23.827999 38.691002

CA 21.878000 22.666000 38.573002

HA 22.124001 22.055000 39.431000

CB 21.163000 21.875000 37.471001

HB2 20.702000 22.600000 36.804001

HB3 21.947001 21.290001 36.990002

CG 19.973000 20.997000 37.820000

OD1 19.743000 20.728001 39.016998

OD2 19.207001 20.649000 36.886002

C 23.240999 23.125000 38.007000

O 23.325001 24.277000 37.606998

N 24.268999 22.284000 37.839001

H 24.204000 21.313000 38.124001

CA 25.601000 22.799000 37.491001

HA 25.476999 23.566000 36.723999

CB 26.190001 23.403000 38.807999

HB2 27.049999 24.068001 38.640999

HB3 25.459999 24.061001 39.266998

CG 26.393000 22.365999 39.905998

CD1 27.611000 21.691999 40.019001

HD1 28.355000 21.882999 39.263000

CE1 27.832001 20.806000 41.104000

HE1 28.761999 20.295000 41.264999

CZ 26.813999 20.535000 42.034000

OH 27.027000 19.722000 43.105000

HH 26.167000 19.499001 43.500000

CE2 25.566000 21.173000 41.881001

HE2 24.771000 20.990999 42.590000

CD2 25.379999 22.115000 40.845001

HD2 24.466999 22.687000 40.798000

C 26.548000 21.757999 36.841999

O 26.264999 20.562000 36.765999

N 27.715000 22.256001 36.431999

H 27.788000 23.268999 36.443001

CA 28.966000 21.523001 36.220001

HA 28.785000 20.464001 36.391998

CB 29.473000 21.715000 34.785999

HB2 29.673000 22.780001 34.638000

HB3 30.424000 21.188999 34.691002

CG 28.575001 21.254000 33.682999

CD1 28.520000 19.993999 33.203999

HD1 29.118999 19.170000 33.570999

NE1 27.591999 19.921000 32.181999

HE1 27.400000 19.073000 31.650000

CE2 26.987000 21.136999 31.965000

CZ2 25.996000 21.559999 31.066000

HZ2 25.545000 20.853001 30.385000

CH2 25.591999 22.905001 31.084999

HH2 24.825001 23.253000 30.406000

CZ3 26.181999 23.805000 31.992001

HZ3 25.879000 24.844000 32.000000

CE3 27.160000 23.360001 32.903000

HE3 27.587000 24.054001 33.611000

CD2 27.591999 22.016001 32.912998

C 30.048000 22.013000 37.201000

O 29.965000 23.136000 37.695999

N 31.097000 21.226999 37.451000

H 31.149000 20.343000 36.950001

CA 32.271999 21.660000 38.250000

HA 32.383999 22.754000 38.215000

CB 32.064999 21.211000 39.720001

HB 31.139000 21.681999 40.029999

CG2 31.996000 19.711000 39.967999

HG21 31.782000 19.476000 41.012001

HG22 31.171000 19.278999 39.423000

HG23 32.903999 19.240999 39.596001

OG1 33.088001 21.663000 40.578999

HG1 32.994999 22.631001 40.549999

C 33.553001 21.070000 37.700001

O 33.518002 19.996000 37.082001

N 34.683998 21.719000 37.994999

H 34.611000 22.587000 38.511002

CA 36.047001 21.191999 37.855999

HA 36.041000 20.158001 38.201000

CB 36.469002 21.201000 36.372002

HB2 37.384998 20.618000 36.276001

HB3 35.716000 20.686001 35.777000

CG 36.705002 22.576000 35.771999

CD1 35.623001 23.437000 35.493000

HD1 34.609001 23.110001 35.668999

CE1 35.859001 24.737000 35.005001

HE1 35.035000 25.409000 34.816002

CZ 37.182999 25.167000 34.775002

OH 37.429001 26.422001 34.327999

HH 36.598000 26.877001 34.092999

CE2 38.261002 24.291000 35.007000

HE2 39.268002 24.617001 34.800999

CD2 38.020000 22.999001 35.505001

HD2 38.848000 22.333000 35.695000

C 37.034000 22.000999 38.743000

O 36.764000 23.174999 39.034000

N 38.168999 21.417000 39.183998

CD 38.511002 20.004000 39.104000

HD2 38.355999 19.601999 38.103001

HD3 37.914001 19.451000 39.833000

CG 39.985001 19.898001 39.493000

HG2 40.612000 20.004999 38.609001

HG3 40.199001 18.959000 40.000000

CB 40.174999 21.084999 40.433998

HB2 41.212002 21.427999 40.466999

HB3 39.838001 20.805000 41.431999

CA 39.236000 22.149000 39.862999

HA 38.821999 22.747999 40.675999

C 39.977001 23.070000 38.889999

O 40.285999 22.674999 37.758999

N 40.279999 24.296000 39.319000

H 40.035000 24.568001 40.265999

CA 40.750999 25.337999 38.410999

HA2 41.320000 24.896000 37.595001

HA3 39.868000 25.801001 37.980999

C 41.598000 26.447001 39.035000

O 41.895000 26.447001 40.234001

N 41.922001 27.430000 38.195000

H 41.603001 27.348000 37.237999

CA 42.608002 28.674000 38.556999

HA 42.995998 28.584000 39.569000

CB 43.799000 28.893000 37.606998

HB2 44.425999 29.695999 37.994999

HB3 44.397999 27.982000 37.573002

OG 43.377998 29.232000 36.291000

HG 43.125000 30.180000 36.284000

C 41.667000 29.886000 38.519001

O 40.519001 29.792000 38.080002

N 42.196999 31.054001 38.894001

H 43.102001 31.024000 39.346001

CA 41.700001 32.362000 38.433998

HA 40.661999 32.487999 38.745998

CB 42.567001 33.466999 39.073002

HB2 43.591000 33.321999 38.735001

HB3 42.245998 34.438000 38.700001

CG 42.570999 33.514999 40.613998

HG 42.798000 32.528000 41.016998

CD1 43.660999 34.478001 41.085999

HD11 44.634998 34.132999 40.745998

HD12 43.469002 35.473999 40.686001

HD13 43.661999 34.526001 42.174999

CD2 41.230000 33.987000 41.175999

HD21 40.437000 33.303001 40.882000

HD22 41.277000 34.018002 42.264999

HD23 40.995998 34.984001 40.799999

C 41.750000 32.469002 36.880001

O 42.604000 31.802000 36.262001

N 40.876999 33.316002 36.278999

H 40.313000 33.917999 36.898998

CA 40.960999 33.605000 34.858002

HA 41.546001 32.824001 34.347000

CB 39.602001 33.625000 34.130001

HB 39.858002 33.717999 33.057999

CG2 38.792000 32.356998 34.358002

HG21 39.383999 31.462000 34.112999

HG22 38.493999 32.293999 35.412998

HG23 37.887001 32.375999 33.734001

OG1 38.883999 34.785999 34.498001

HG1 38.112999 34.869999 33.834999

C 41.681000 34.926998 34.525002

O 41.769001 35.335999 33.366001

N 42.206001 35.567001 35.583000

H 42.119999 35.122002 36.490002

CA 42.995998 36.763000 35.490002

HA 43.277000 36.896000 34.433998

CB 42.159000 38.020000 35.907001

HB 42.853001 38.860001 36.087002

CG2 41.174999 38.405998 34.813999

HG21 40.508999 37.570999 34.556000

HG22 40.554001 39.257000 35.130001

HG23 41.708000 38.701000 33.900002

OG1 41.428001 37.743999 37.105999

HG1 41.981998 37.868000 37.921001

C 44.233002 36.608002 36.374001

O 44.185001 35.856998 37.340000

N 45.346001 37.298000 36.109001

CD 45.629002 38.020000 34.886002

HD2 45.173000 39.007999 34.931000

HD3 45.279999 37.487000 34.001999

CG 47.150002 38.145000 34.837002

HG2 47.473000 39.012001 34.264000

HG3 47.582001 37.231998 34.424999

CB 47.504002 38.264999 36.318001

HB2 47.330002 39.285999 36.659000

HB3 48.528999 37.959000 36.508999

CA 46.514000 37.301998 36.973000

HA 46.951000 36.312000 36.955002

C 46.185001 37.672001 38.429001

O 45.382000 38.589001 38.640999

N 46.754002 36.985001 39.442001

CD 46.417000 37.294998 40.823002

HD2 45.408001 36.963001 41.061001

HD3 46.498001 38.372002 40.973999

CG 47.442001 36.588001 41.702999

HG2 47.063000 35.617001 42.013000

HG3 47.701000 37.195000 42.564999

CB 48.634998 36.415001 40.771000

HB2 49.284000 35.602001 41.099998

HB3 49.195999 37.352001 40.745998

CA 47.974998 36.164001 39.402000

HA 48.639999 36.577000 38.646000

C 47.811001 34.653000 39.075001

O 48.755001 33.896999 39.311001

N 46.669998 34.193001 38.532001

H 45.916000 34.855000 38.374001

CA 46.473000 32.821999 37.987999

HA 45.426998 32.756001 37.694000

CB 47.294998 32.625000 36.689999

HB2 48.355000 32.665001 36.944000

HB3 47.095001 31.627001 36.299999

CG 47.028999 33.620998 35.548000

HG 47.264000 34.625999 35.876999

CD1 47.945999 33.304001 34.372002

HD11 47.742001 32.298000 34.008999

HD12 47.786999 34.021000 33.568001

HD13 48.985001 33.367001 34.694000

CD2 45.582001 33.574001 35.058998

HD21 44.902000 33.879002 35.849998

HD22 45.449001 34.243000 34.209000

HD23 45.332001 32.556999 34.770000

C 46.650002 31.632999 38.973000

O 46.863998 30.500000 38.536999

N 46.548000 31.872999 40.285999

H 46.390999 32.825001 40.567001

CA 46.738998 30.871000 41.351002

HA 47.702999 30.388000 41.183998

CB 46.778000 31.587000 42.721001

HB2 45.787998 32.004002 42.902000

HB3 46.973000 30.850000 43.500000

CG 47.808998 32.729000 42.842999

HG 47.637001 33.452999 42.048000

CD1 47.654999 33.457001 44.176998

HD11 46.634998 33.828999 44.270000

HD12 47.879002 32.776001 45.001999

HD13 48.348999 34.299000 44.202000

CD2 49.249001 32.234001 42.737000

HD21 49.429001 31.459999 43.480999

HD22 49.433998 31.833000 41.742001

HD23 49.937000 33.058998 42.917999

C 45.675999 29.749001 41.335999

O 44.543999 29.952000 40.890999

N 46.043999 28.566999 41.848999

H 46.965000 28.514999 42.263000

CA 45.285999 27.294001 41.773998

HA 44.707001 27.312000 40.848999

CB 46.275002 26.098000 41.675999

HB2 46.828999 26.010000 42.612999

HB3 45.700001 25.183001 41.523998

CG 47.283001 26.228001 40.511002

HG2 46.723999 26.419001 39.594002

HG3 47.923000 27.091000 40.696999

CD 48.195999 25.006001 40.277000

OE1 48.195000 24.028000 41.063000

OE2 48.949001 24.983000 39.276001

C 44.233002 27.094999 42.904999

O 43.840000 25.964001 43.219002

N 43.778000 28.184999 43.535999

H 44.136002 29.082001 43.238998

CA 42.914001 28.201000 44.726002

HA 43.172001 27.344000 45.347000

CB 43.257999 29.469000 45.535999

HB2 42.570000 29.577000 46.376999

HB3 44.263000 29.356001 45.946999

SG 43.214001 30.981001 44.528000

HG 43.830002 31.740000 45.446999

C 41.394001 28.073000 44.466999

O 40.620998 28.171000 45.422001

N 40.938000 27.858000 43.222000

H 41.615002 27.698999 42.488998

CA 39.512001 27.965000 42.832001

HA 38.959999 28.412001 43.659000

CB 39.348000 28.914000 41.623001

HB 39.886002 28.502001 40.769001

CG1 37.872002 29.079000 41.230000

HG11 37.282001 29.365999 42.098999

HG12 37.775002 29.841999 40.456001

HG13 37.481998 28.148001 40.820999

CG2 39.909000 30.315001 41.905998

HG21 39.777000 30.944000 41.026001

HG22 39.387001 30.766001 42.749001

HG23 40.974998 30.263000 42.125000

C 38.846001 26.614000 42.523998

O 39.278999 25.889999 41.626999

N 37.712002 26.315001 43.169998

H 37.411999 26.920000 43.924999

CA 36.742001 25.330000 42.653000

HA 37.256001 24.627001 42.000000

CB 36.109001 24.525999 43.792000

HB 35.492001 25.188999 44.397999

CG2 35.264999 23.356001 43.280998

HG21 35.870998 22.716999 42.637001

HG22 34.900002 22.768999 44.124001

HG23 34.407001 23.726999 42.717999

OG1 37.138000 23.992001 44.585999

HG1 36.707001 23.499001 45.318001

C 35.654999 26.031000 41.834000

O 35.020000 26.966000 42.325001

N 35.430000 25.606001 40.589001

H 35.979000 24.839001 40.216000

CA 34.395000 26.184999 39.722000

HA 34.247002 27.233000 39.991001

CB 34.880001 26.158001 38.268002

HB2 35.248001 25.162001 38.016998

HB3 34.035000 26.370001 37.612000

CG 35.941002 27.174000 37.980999

CD1 37.273998 26.996000 38.124001

HD1 37.737000 26.066999 38.436001

NE1 37.930000 28.188000 37.868999

HE1 38.937000 28.323999 37.932999

CE2 37.044998 29.193001 37.549999

CZ2 37.214001 30.549000 37.245998

HZ2 38.205002 30.980000 37.251999

CH2 36.083000 31.330999 36.955002

HH2 36.193001 32.382000 36.723000

CZ3 34.804001 30.745001 36.984001

HZ3 33.932999 31.348000 36.778000

CE3 34.645000 29.385000 37.312000

HE3 33.653999 28.955000 37.351002

CD2 35.763000 28.573000 37.601002

C 33.043999 25.480000 39.884998

O 32.993999 24.253000 39.987000

N 31.948999 26.243999 39.856998

H 32.080002 27.253000 39.873001

CA 30.559999 25.759001 39.791000

HA 30.549999 24.715000 39.487999

CB 29.871000 25.875000 41.173000

HB 30.021999 26.893000 41.534000

CG2 28.357000 25.614000 41.087002

HG21 27.868999 26.327000 40.424000

HG22 28.172001 24.600000 40.730000

HG23 27.906000 25.731001 42.068001

CG1 30.479000 24.888000 42.194000

HG12 30.169001 23.870001 41.950001

HG13 31.563999 24.934999 42.139000

CD1 30.128000 25.204000 43.652000

HD11 29.055000 25.160999 43.842999

HD12 30.613001 24.478001 44.293999

HD13 30.509001 26.193001 43.896999

C 29.829000 26.572001 38.717999

O 29.535000 27.743000 38.923000

N 29.576000 25.981001 37.548000

H 29.785999 24.992001 37.463001

CA 28.997000 26.663000 36.369999

HA 28.962000 27.736000 36.554001

CB 29.872000 26.448000 35.118999

HB 30.004999 25.377001 34.955002

CG1 29.229000 27.047001 33.862000

HG11 28.950001 28.084999 34.046001

HG12 29.929001 27.011000 33.028999

HG13 28.336000 26.486000 33.584999

CG2 31.259001 27.080000 35.307999

HG21 31.861000 26.933001 34.410000

HG22 31.156000 28.150000 35.488998

HG23 31.775000 26.620001 36.148998

C 27.563999 26.191000 36.132999

O 27.334000 24.990000 36.032001

N 26.598000 27.107000 36.055000

H 26.858000 28.087000 36.131001

CA 25.166000 26.784000 36.028000

HA 24.999001 25.980000 36.742001

CB 24.348000 28.004999 36.493999

HB2 24.569000 28.843000 35.832001

HB3 23.295000 27.771999 36.361000

CG 24.559999 28.455999 37.955002

HG 25.545000 28.908001 38.051998

CD1 23.507999 29.502001 38.330002

HD11 23.454000 30.266001 37.555000

HD12 22.533001 29.025999 38.429001

HD13 23.775999 29.973000 39.271999

CD2 24.437000 27.319000 38.974998

HD21 25.313000 26.674999 38.915001

HD22 24.374001 27.725000 39.986000

HD23 23.542000 26.730000 38.770000

C 24.643000 26.275999 34.667000

O 24.985001 26.813000 33.609001

N 23.715000 25.309999 34.722000

H 23.462000 25.039000 35.668999

CA 22.950001 24.754999 33.564999

HA 23.570999 24.298000 32.786999

CB 21.951000 23.719999 34.089001

HB2 22.527000 23.044001 34.717999

HB3 21.165001 24.205000 34.662998

CG 21.235001 22.927999 32.990002

HG2 20.789000 23.601000 32.252998

HG3 22.004999 22.333000 32.516998

CD 20.157000 21.966999 33.490002

HD2 19.806000 21.419001 32.622002

HD3 20.591000 21.277000 34.214001

CE 18.964001 22.687000 34.092999

HE2 19.346001 23.299999 34.901001

HE3 18.514999 23.326000 33.332001

NZ 17.971001 21.764999 34.652000

HZ1 17.583000 21.198000 33.903999

HZ2 18.451000 21.204000 35.349998

HZ3 17.238001 22.256001 35.151001

C 22.165001 25.841000 32.870998

O 22.072001 25.830999 31.646999

N 21.483999 26.683001 33.641998

H 21.563000 26.575001 34.648998

CA 20.525000 27.643999 33.112999

HA 20.164000 27.242001 32.168999

CB 19.336000 27.785000 34.098999

HB2 18.886999 26.806000 34.229000

HB3 19.738001 28.096001 35.075001

CG 18.150000 28.636999 33.543999

HG2 18.371000 29.704000 33.459999

HG3 17.875999 28.292000 32.546001

CD 16.844000 28.502001 34.356998

OE1 16.344000 27.368999 34.590000

OE2 16.343000 29.593000 34.749001

C 21.174999 29.011000 32.862999

O 21.836000 29.518999 33.765999

N 21.020000 29.643000 31.690001

CD 20.472000 29.052999 30.465000

HD2 19.417999 28.805000 30.600000

HD3 21.000000 28.132000 30.187000

CG 20.591000 30.186001 29.430000

HG2 19.635000 30.709000 29.480000

HG3 20.820000 29.882000 28.410999

CB 21.731001 31.020000 29.971001

HB2 21.739000 32.026001 29.570000

HB3 22.686001 30.513000 29.794001

CA 21.461000 31.018999 31.458000

HA 22.406000 31.254999 31.934000

C 20.488001 32.110001 31.952999

O 19.315001 31.871000 32.255001

N 20.952999 33.362999 31.997000

H 21.917999 33.527000 31.721001

CA 20.086000 34.521999 32.294998

HA 19.077999 34.151001 32.473999

CB 20.458000 35.237999 33.608002

HB 19.646999 35.937000 33.773998

CG2 20.452000 34.228001 34.770000

HG21 19.532000 33.641998 34.750999

HG22 21.299999 33.551998 34.671001

HG23 20.520000 34.738998 35.729000

CG1 21.740999 36.087002 33.573002

HG12 22.601000 35.443001 33.737000

HG13 21.850000 36.550999 32.597000

CD1 21.716999 37.210999 34.619999

HD11 21.530001 36.799000 35.609001

HD12 22.674000 37.730000 34.627998

HD13 20.927999 37.924999 34.379002

C 19.931999 35.472000 31.101000

O 20.819000 35.560001 30.252001

N 18.816000 36.200001 31.040001

H 18.181999 36.153999 31.836000

CA 18.593000 37.205002 29.990000

HA 19.004999 36.868999 29.032000

CB 17.104000 37.382999 29.787001

HB2 16.658001 37.651001 30.746000

HB3 16.950001 38.175999 29.049999

OG 16.535999 36.159000 29.343000

HG 16.823999 35.452999 29.945000

C 19.209999 38.574001 30.344999

O 19.142000 39.046001 31.487000

N 19.754000 39.256001 29.332001

H 19.829000 38.823002 28.419001

CA 20.327999 40.598000 29.416000

HA 19.787001 41.105000 30.207001

CB 21.844000 40.534000 29.731001

HB 21.976000 39.986000 30.669001

CG1 22.721001 39.879002 28.639000

HG11 23.722000 39.813999 29.058001

HG12 22.417000 38.879002 28.315001

HG13 22.737000 40.514999 27.754999

CG2 22.372999 41.984001 29.871000

HG21 21.812000 42.516998 30.624001

HG22 23.416000 42.082001 30.167000

HG23 22.223000 42.554001 28.955000

C 20.077000 41.368000 28.134001

O 20.158001 40.777000 27.066999

N 19.705000 42.643002 28.181000

H 19.594999 43.145000 29.055000

CA 19.325001 43.355000 26.971001

HA 18.667999 42.688999 26.402000

CB 18.646999 44.643002 27.388000

HB2 17.997999 44.407001 28.226000

HB3 19.455999 45.300999 27.718000

OG 17.907000 45.243000 26.341999

HG 17.482000 46.033001 26.743000

C 20.549999 43.787998 26.152000

O 21.631001 44.002998 26.697001

N 20.431000 43.955002 24.834999

H 19.562000 43.679001 24.388000

CA 21.521999 44.438000 23.961000

HA 22.385000 43.783001 24.096001

CB 20.972000 44.248001 22.545000

HB2 20.719999 43.187000 22.431999

HB3 20.084000 44.873001 22.410000

OG 21.961000 44.679001 21.646000

HG 22.767000 44.160000 21.801001

C 22.011000 45.877998 24.233000

O 23.198000 46.203999 24.135000

N 21.135000 46.712002 24.795000

H 20.157000 46.450001 24.787001

CA 21.499001 48.028000 25.358000

HA 22.010000 48.594002 24.587000

CB 20.156000 48.716000 25.743000

HB2 19.520000 48.824001 24.861000

HB3 19.641001 48.057999 26.452999

CG 20.253000 50.103001 26.410999

HG2 19.246000 50.463001 26.613001

HG3 20.739000 49.971001 27.368999

CD 20.958000 51.209999 25.629999

OE1 20.974001 51.112000 24.393999

OE2 21.355000 52.250000 26.195999

C 22.486000 47.993999 26.535999

O 23.445999 48.755001 26.646000

N 22.291000 47.039001 27.424000

H 21.627001 46.324001 27.177999

CA 23.027000 46.907001 28.670000

HA 23.045000 47.867001 29.201000

CB 22.188000 45.981998 29.525000

HB2 21.867001 45.176998 28.881001

HB3 22.750999 45.502998 30.327999

CG 20.898001 46.660000 30.034000

HG2 21.108000 47.400002 30.805000

HG3 20.329000 47.119999 29.229000

CD 19.927999 45.647999 30.584000

OE1 20.035999 44.465000 30.320999

NE2 18.952999 46.069000 31.356001

HE21 18.360001 45.398998 31.819000

HE22 18.806999 47.069000 31.441999

C 24.405001 46.306000 28.351999

O 25.417000 46.785999 28.846001

N 24.483999 45.358002 27.396999

H 23.622999 45.002998 26.997999

CA 25.754999 44.855000 26.844999

HA 26.381001 44.549000 27.681000

CB 25.566999 43.598999 25.962000

HB 25.030001 43.863998 25.049999

CG1 26.931999 43.009998 25.577999

HG11 27.485001 43.710999 24.955999

HG12 27.507000 42.798000 26.480000

HG13 26.801001 42.084000 25.014999

CG2 24.791000 42.491001 26.691000

HG21 23.742001 42.761002 26.771999

HG22 24.844000 41.554001 26.136000

HG23 25.194000 42.341999 27.691000

C 26.540001 45.952999 26.114000

O 27.732000 46.102001 26.384001

N 25.933001 46.779999 25.249001

H 24.950001 46.654999 25.021000

CA 26.684000 47.875000 24.618999

HA 27.538000 47.400002 24.129999

CB 25.846001 48.532001 23.507000

HB2 24.943001 48.944000 23.950001

HB3 26.363001 49.382999 23.075001

CG 25.577999 47.591999 22.320999

HG 25.540001 46.567001 22.686001

CD1 24.290001 47.816002 21.531000

HD11 24.230000 48.840000 21.153000

HD12 24.333000 47.158001 20.667999

HD13 23.434999 47.502998 22.132000

CD2 26.705999 47.749001 21.280001

HD21 27.666000 47.854000 21.788000

HD22 26.674000 46.918999 20.573999

HD23 26.542000 48.631001 20.662001

C 27.188000 48.965000 25.621000

O 28.289000 49.480000 25.427999

N 26.461000 49.202999 26.729000

H 25.562000 48.748001 26.834999

CA 26.908001 49.930000 27.916000

HA 27.348000 50.869999 27.603001

CB 25.677000 50.227001 28.747999

HB2 25.075001 49.327000 28.705999

HB3 25.987000 50.354000 29.780001

CG 24.906000 51.465000 28.267000

HG2 25.594999 52.303001 28.330000

HG3 24.632999 51.332001 27.219999

CD 23.667999 51.827000 29.110001

HD2 22.785000 51.245998 28.830999

HD3 23.920000 51.584999 30.134001

CE 23.341999 53.337002 29.018000

HE2 22.452000 53.583000 29.594000

HE3 24.165001 53.919998 29.431000

NZ 22.986000 53.769001 27.660999

HZ1 22.350000 53.071999 27.292999

HZ2 22.471001 54.637001 27.648001

HZ3 23.726999 53.834000 26.982000

C 27.945000 49.171001 28.738001

O 28.625999 49.820000 29.513000

N 28.172001 47.863998 28.582001

H 27.533001 47.313000 28.021000

CA 29.426001 47.237999 29.047001

HA 29.698999 47.657001 30.018000

CB 29.323000 45.709000 29.201000

HB2 29.341000 45.263000 28.208000

HB3 30.230000 45.388000 29.709000

CG 28.164000 45.078999 29.955999

CD1 27.405001 45.794998 30.902000

HD1 27.612000 46.835999 31.098000

CE1 26.339001 45.166000 31.570000

HE1 25.754000 45.722000 32.285999

CZ 26.033001 43.819000 31.312000

HZ 25.204000 43.346001 31.818001

CE2 26.805000 43.091999 30.391001

HE2 26.577999 42.053001 30.195000

CD2 27.868999 43.721001 29.719999

HD2 28.452000 43.162998 29.000999

C 30.573000 47.558998 28.073999

O 31.650000 47.978001 28.500000

N 30.329000 47.456001 26.756001

H 29.407000 47.110001 26.493999

CA 31.274000 47.758999 25.656000

HA 32.209000 47.243000 25.872999

CB 30.711000 47.236000 24.320000

HB2 29.799999 47.789001 24.098000

HB3 31.424999 47.462002 23.531000

CG 30.410000 45.736000 24.228001

HG2 31.309000 45.167000 24.434999

HG3 29.646000 45.459000 24.947001

CD 29.933001 45.409000 22.809999

HD2 29.107000 46.077999 22.559999

HD3 30.759001 45.598999 22.118999

NE 29.514000 43.998001 22.698999

HE 30.087000 43.316002 23.184999

CZ 28.511000 43.520000 21.988001

NH1 27.850000 44.233002 21.128000

HH11 28.264000 45.105999 20.822001

HH12 27.105000 43.797001 20.605000

NH2 28.143999 42.285000 22.099001

HH21 28.650000 41.644001 22.705000

HH22 27.392000 41.958000 21.496000

C 31.646000 49.254002 25.506001

O 31.917999 49.712002 24.393000

N 31.625999 50.021999 26.600000

H 31.417999 49.537998 27.466000

CA 31.931000 51.469002 26.635000

HA 32.793999 51.657001 25.997000

CB 30.726000 52.271000 26.058001

HB2 31.107000 53.254002 25.785000

HB3 30.385000 51.851002 25.115000

CG 29.415001 52.327999 26.872000

HG2 28.545000 52.480000 26.219999

HG3 29.291000 51.390999 27.408001

CD 29.436001 53.487999 27.841999

HD2 30.094000 53.262001 28.681000

HD3 29.882000 54.334000 27.343000

CE 27.993999 53.888000 28.155001

HE2 27.496000 54.096001 27.200001

HE3 27.500999 53.036999 28.629999

NZ 27.923000 55.134998 28.952000

HZ1 28.273001 55.904999 28.396000

HZ2 26.958000 55.363998 29.124001

HZ3 28.381001 55.145000 29.868999

C 32.563999 51.965000 27.931000

O 32.747002 53.165001 28.099001

N 32.963001 51.044998 28.813999

H 32.743999 50.082001 28.605000

CA 33.986000 51.285000 29.844000

HA 33.854000 52.285999 30.260000

CB 33.818001 50.255001 30.985001

HB2 33.764000 49.244999 30.573999

HB3 34.703999 50.305000 31.615999

CG 32.596001 50.541000 31.882000

HG 32.363998 51.602001 31.834000

CD1 31.363001 49.748001 31.457001

HD11 31.500000 48.685001 31.656000

HD12 30.504999 50.118999 32.009998

HD13 31.164000 49.893002 30.398001

CD2 32.884998 50.194000 33.345001

HD21 33.707001 50.807999 33.712002

HD22 32.004002 50.404999 33.949001

HD23 33.151001 49.140999 33.442001

C 35.409000 51.237000 29.238001

O 35.583000 51.027000 28.034000

N 36.441002 51.418999 30.065001

H 36.254002 51.664001 31.033001

CA 37.833000 51.523998 29.650000

HA 37.938999 51.083000 28.659000

CB 38.196999 53.011002 29.577000

HB2 37.929001 53.516998 30.503000

HB3 39.266998 53.106998 29.448999

CG 37.528000 53.707001 28.419001

OD1 36.695000 54.589001 28.582001

ND2 37.918999 53.363998 27.219999

HD21 37.590000 53.897999 26.420000

HD22 38.673000 52.695000 27.136000

C 38.799000 50.772999 30.573000

O 38.629002 50.742001 31.794001

N 39.846001 50.201000 29.968000

H 39.910999 50.284000 28.959000

CA 40.983002 49.608002 30.681000

HA 40.618999 48.999001 31.507000

CB 41.792999 48.721001 29.719999

HB2 42.235001 49.362000 28.959000

HB3 42.625999 48.297001 30.282000

CG 41.084999 47.562000 29.027000

CD1 40.569000 46.491001 29.780001

HD1 40.564999 46.547001 30.854000

CE1 40.108002 45.323002 29.146000

HE1 39.752998 44.492001 29.739000

CZ 40.126999 45.228001 27.743999

HZ 39.785999 44.326000 27.250000

CE2 40.615002 46.304001 26.985001

HE2 40.651001 46.224998 25.907000

CD2 41.087002 47.465000 27.622000

HD2 41.491001 48.263000 27.018999

C 41.925999 50.669998 31.285000

O 42.640999 50.386002 32.249001

N 41.959000 51.876999 30.709000

H 41.366001 52.040001 29.908001

CA 42.813999 52.985001 31.132999

HA 43.765999 52.569000 31.472000

CB 43.097000 53.880001 29.916000

HB2 43.719002 54.717999 30.209999

HB3 43.646000 53.299000 29.176001

CG 41.839001 54.409000 29.256001

OD1 41.291000 53.773998 28.375000

ND2 41.304001 55.528999 29.667999

HD21 40.472000 55.852001 29.174999

HD22 41.674999 56.019001 30.469000

C 42.233002 53.813000 32.298000

O 41.030998 53.799000 32.556999

N 43.108002 54.580002 32.945000

H 44.049000 54.493999 32.575001

CA 42.799000 55.694000 33.872002

HA2 42.080002 55.333000 34.612999

HA3 43.708000 55.917000 34.433998

C 42.287998 57.021000 33.195000

O 42.007999 57.076000 31.982000

N 42.101002 58.071999 34.018002

H 42.415001 57.969002 34.973000

CA 41.582001 59.431000 33.783001

HA 40.612999 59.424999 33.279999

CB 41.264999 60.035999 35.169998

HB2 40.519001 59.459999 35.717999

HB3 42.147999 59.958000 35.786999

CG 40.814999 61.479000 35.167999

HG2 41.119999 61.928001 36.117001

HG3 41.278999 62.008999 34.338001

CD 39.282001 61.532001 35.067001

OE1 38.578999 61.140999 36.021000

OE2 38.701000 61.931999 34.035999

C 42.639999 60.285000 33.008999

O 43.797001 60.380001 33.408001

N 42.307999 60.907001 31.885000

H 41.333000 60.893002 31.625999

CA 43.238998 61.699001 31.054001

HA2 42.715000 62.516998 30.546000

HA3 43.988998 62.118000 31.724001

C 44.069000 60.894001 30.024000

O 44.582001 61.487999 29.079000

N 44.181000 59.570000 30.169001

H 43.582001 59.145000 30.863001

CA 45.012001 58.629002 29.360001

HA 46.023998 59.015999 29.181000

CB 45.117001 57.318001 30.153000

HB2 44.147999 57.035999 30.563000

HB3 45.472000 56.484001 29.548000

CG 46.085999 57.506001 31.297001

HG2 46.991001 57.985001 30.914000

HG3 45.660999 58.189999 32.028000

CD 46.382000 56.173000 32.002998

OE1 45.669998 55.155998 31.806999

OE2 47.348999 56.161999 32.805000

C 44.355999 58.359001 27.997000

O 43.127998 58.333000 27.910999

N 45.131001 58.158001 26.910999

CD 46.575001 58.424999 26.778000

HD2 47.111000 57.834999 27.517000

HD3 46.816002 59.465000 26.989000

CG 47.018002 58.083000 25.347000

HG2 47.903000 57.445000 25.360001

HG3 47.206001 58.966999 24.726999

CB 45.840000 57.280998 24.789000

HB2 46.053001 56.222000 24.938999

HB3 45.703999 57.477001 23.726999

CA 44.598000 57.641998 25.642000

HA 43.985001 58.443001 25.229000

C 43.653000 56.442001 25.730000

O 43.811001 55.564999 26.579000

N 42.648998 56.469002 24.863001

H 42.616001 57.308998 24.292999

CA 41.504002 55.544998 24.848000

HA 41.055000 55.664001 25.830999

CB 40.462002 56.021000 23.792000

HB2 40.153999 57.056000 23.952000

HB3 41.004002 56.071999 22.851000

CG 39.169998 55.167000 23.608000

HG2 38.726002 55.414001 22.641001

HG3 39.421001 54.115002 23.513000

CD 38.132000 55.319000 24.742001

OE1 38.356998 56.174999 25.625000

OE2 37.117001 54.570000 24.774000

C 41.909000 54.054001 24.650999

O 42.299000 53.653999 23.551001

N 41.714001 53.223000 25.681000

H 41.473000 53.634998 26.576000

CA 41.675999 51.754002 25.624001

HA 41.948002 51.424000 24.624001

CB 42.634998 51.103001 26.635000

HB2 42.285999 51.312000 27.646000

HB3 42.612000 50.023998 26.480000

CG 44.084000 51.555000 26.570000

HG2 44.117001 52.609001 26.850000

HG3 44.653000 50.993000 27.313999

CD 44.742001 51.372002 25.204000

OE1 45.729000 52.092999 24.966000

OE2 44.346001 50.519001 24.371000

C 40.257999 51.259998 25.930000

O 39.823002 51.201000 27.087000

N 39.506001 50.929001 24.885000

H 39.925999 50.967999 23.961000

CA 38.101002 50.528999 24.978001

HA 37.575001 51.264000 25.590000

CB 37.556999 50.580002 23.539000

HB2 37.667000 51.603001 23.171000

HB3 38.176998 49.933998 22.917000

CG 36.103001 50.145000 23.333000

HG 35.987999 49.085999 23.558001

CD1 35.157001 50.951000 24.209999

HD11 35.257000 50.645000 25.247999

HD12 35.376999 52.008999 24.099001

HD13 34.132999 50.771000 23.892000

CD2 35.740002 50.398998 21.872999

HD21 34.743000 50.012001 21.669001

HD22 35.755001 51.469002 21.659000

HD23 36.448002 49.893002 21.218000

C 37.941002 49.139999 25.629999

O 38.535000 48.173000 25.150000

N 37.139999 49.012001 26.697001

H 36.632999 49.821999 27.034000

CA 36.932999 47.737000 27.402000

HA 37.887001 47.205002 27.400999

CB 36.556999 47.952000 28.879999

HB2 37.222000 48.698002 29.309000

HB3 35.526001 48.297001 28.964001

CG 36.709999 46.633999 29.655001

HG2 36.083000 45.882999 29.177000

HG3 37.742001 46.300999 29.566999

SD 36.266998 46.637001 31.417000

CE 37.345001 47.926998 32.074001

HE1 38.367001 47.777000 31.729000

HE2 36.980999 48.896000 31.740999

HE3 37.320999 47.897999 33.162998

C 35.929001 46.838001 26.667000

O 34.717999 46.889000 26.886999

N 36.476002 45.981998 25.809000

H 37.478001 46.043999 25.683001

CA 35.806000 44.875999 25.113001

HA 34.992001 44.509998 25.739000

CB 35.221001 45.293999 23.745001

HB 34.803001 44.396000 23.295000

CG1 34.056000 46.271999 23.875000

HG11 33.375000 45.895000 24.631001

HG12 34.403999 47.262001 24.166000

HG13 33.528000 46.348999 22.924000

CG2 36.252998 45.870998 22.768999

HG21 36.763000 46.726002 23.212000

HG22 36.984001 45.104000 22.516001

HG23 35.764000 46.192001 21.848000

C 36.791000 43.723000 24.924999

O 38.005001 43.931999 24.958000

N 36.269001 42.512001 24.724001

H 35.266998 42.404999 24.804001

CA 37.049999 41.272999 24.632999

HA 36.301998 40.493000 24.646999

CB 37.799999 41.146000 23.285999

HB2 38.466999 41.998001 23.159000

HB3 38.403000 40.237999 23.296000

CG 36.873001 41.049999 22.073999

OD1 35.776001 40.450001 22.181000

OD2 37.234001 41.555000 20.983000

C 37.890999 40.972000 25.886999

O 38.952000 40.353001 25.819000

N 37.384998 41.363998 27.065001

H 36.452999 41.758999 27.059999

CA 37.991001 41.125000 28.384001

HA 39.068001 41.245998 28.266001

CB 37.521999 42.209000 29.379999

HB2 38.118000 42.118999 30.285999

HB3 37.708000 43.195000 28.957001

CG 36.050999 42.131001 29.767000

OD1 35.228001 41.528999 29.101000

ND2 35.671001 42.751999 30.856001

HD21 34.681999 42.783001 31.052999

HD22 36.348999 43.207001 31.454000

C 37.785999 39.679001 28.899000

O 37.546001 39.451000 30.087000

N 37.855000 38.702000 27.993000

H 38.105000 38.974998 27.052000

CA 37.698002 37.266998 28.243999

HA 37.466000 37.115002 29.297001

CB 36.512001 36.730000 27.419001

HB2 36.480000 35.644001 27.516001

HB3 35.594002 37.115002 27.864000

CG 36.494999 37.069000 25.952999

CD1 35.750999 38.050999 25.395000

HD1 35.084999 38.701000 25.952000

NE1 36.008999 38.130001 24.039000

HE1 35.648998 38.865002 23.427999

CE2 36.950001 37.205002 23.656000

CZ2 37.554001 36.922001 22.424999

HZ2 37.307999 37.505001 21.549000

CH2 38.502998 35.890999 22.350000

HH2 38.988998 35.681999 21.409000

CZ3 38.820000 35.143002 23.497000

HZ3 39.541000 34.338001 23.431000

CE3 38.203999 35.438999 24.729000

HE3 38.456001 34.863998 25.606001

CD2 37.264999 36.490002 24.851000

C 38.998001 36.504002 27.948000

O 39.713001 36.812000 26.989000

N 39.298000 35.478001 28.753000

H 38.708000 35.342999 29.572001

CA 40.388000 34.516998 28.513000

HA 41.209000 35.077000 28.069000

CB 40.866001 33.942001 29.865999

HB2 40.898998 34.750999 30.598000

HB3 40.141998 33.206001 30.216000

CG 42.271000 33.310001 29.813999

HG2 42.289001 32.509998 29.073999

HG3 42.987000 34.078999 29.524000

CD 42.695999 32.724998 31.169001

HD2 42.543999 33.474998 31.943001

HD3 42.063999 31.870001 31.392000

NE 44.113998 32.313000 31.176001

HE 44.730000 32.714001 30.475000

CZ 44.729000 31.559000 32.068001

NH1 44.133999 30.959999 33.053001

HH11 43.122002 30.930000 33.095001

HH12 44.702999 30.474001 33.735001

NH2 46.009998 31.385000 32.001999

HH21 46.506001 31.802000 31.218000

HH22 46.457001 30.787001 32.679001

C 39.937000 33.411999 27.528000

O 38.775002 32.994999 27.580000

N 40.813999 32.916000 26.632999

CD 42.097000 33.492001 26.270000

HD2 42.655998 33.799000 27.146999

HD3 41.924000 34.342999 25.610001

CG 42.868000 32.405998 25.532000

HG2 43.443001 31.811001 26.245001

HG3 43.515999 32.844002 24.777000

CB 41.764000 31.566999 24.893999

HB2 42.083000 30.531000 24.759001

HB3 41.495998 32.001999 23.931000

CA 40.581001 31.695000 25.863001

HA 39.662998 31.785000 25.282000

C 40.490002 30.455999 26.763000

O 41.108002 30.399000 27.833000

N 39.784000 29.436001 26.270000

H 39.271999 29.584000 25.412001

CA 39.773998 28.093000 26.839001

HA 39.333000 28.146999 27.832001

CB 38.876999 27.184000 25.990000

HB1 37.839001 27.506001 26.086000

HB2 39.179001 27.221001 24.941999

HB3 38.959999 26.160999 26.353001

C 41.202999 27.538000 26.992001

O 42.012001 27.552000 26.061001

N 41.512001 27.068001 28.195000

H 40.803001 27.097000 28.923000

CA 42.769001 26.417000 28.552000

HA 43.577999 26.822001 27.941000

CB 43.061001 26.725000 30.035999

HB2 42.303001 26.252001 30.662001

HB3 44.025002 26.304001 30.316000

CG 43.078999 28.233999 30.346001

HG2 42.117001 28.690001 30.117001

HG3 43.241001 28.372999 31.412001

CD 44.185001 28.962999 29.597000

OE1 45.361000 28.690001 29.774000

NE2 43.862000 29.903999 28.738001

HE21 44.624001 30.403999 28.290001

HE22 42.888000 30.080999 28.511000

C 42.679001 24.907000 28.270000

O 41.569000 24.355000 28.222000

N 43.813999 24.204000 28.101000

CD 45.200001 24.666000 28.042999

HD2 45.402000 25.438999 28.787001

HD3 45.546001 24.976999 27.052999

CG 45.987000 23.400000 28.313000

HG2 46.021999 23.181000 29.375000

HG3 46.983002 23.495001 27.910000

CB 45.219002 22.264000 27.671000

HB2 45.506001 21.306999 28.120001

HB3 45.325001 22.243999 26.587000

CA 43.793999 22.735001 27.985001

HA 43.174000 22.443001 27.143000

C 43.271000 22.100000 29.275999

O 43.668999 22.545000 30.362000

N 42.450001 21.038000 29.198999

H 42.300999 20.570000 28.305000

CA 41.969002 20.412001 30.457001

HA 41.730999 21.236000 31.124001

CB 40.602001 19.719999 30.306000

HB2 39.938000 20.496000 29.924000

HB3 40.730000 18.927000 29.568001

CG 39.943001 19.096001 31.563999

HG 40.646000 18.535999 32.172001

CD1 39.255001 20.016001 32.555000

HD11 39.991001 20.695999 32.969002

HD12 38.387001 20.535000 32.139999

HD13 38.930000 19.410999 33.396000

CD2 38.921001 18.086000 31.045000

HD21 39.411999 17.414000 30.344000

HD22 38.569000 17.462999 31.857000

HD23 38.056000 18.542000 30.559999

C 43.036999 19.573999 31.212999

O 43.113998 19.733999 32.437000

N 43.963001 18.885000 30.525000

H 43.876999 18.903999 29.511000

CA 45.129002 18.165001 31.121000

HA 45.599998 17.628000 30.292999

CB 46.167000 19.132000 31.768999

HB2 45.712002 19.650000 32.613998

HB3 46.999001 18.521999 32.146999

CG 46.789001 20.216999 30.836000

HG2 46.077000 20.973000 30.490000

HG3 47.495998 20.771999 31.451000

CD 47.498001 19.517000 29.646000

HD2 48.143002 18.740000 30.054001

HD3 46.749001 18.990999 29.059000

CE 48.308998 20.365999 28.669001

HE2 47.730999 21.184999 28.219000

HE3 49.159000 20.819000 29.172001

NZ 48.786999 19.480000 27.594999

HZ1 47.978001 19.212000 27.049999

HZ2 49.431999 19.995001 27.014000

HZ3 49.283001 18.627001 27.870001

C 44.608002 17.090000 32.084999

O 43.550999 16.511999 31.846001

N 45.354000 16.737000 33.119999

H 46.171001 17.292000 33.325001

CA 44.963001 15.683000 34.063000

HA 44.578999 14.847000 33.490002

CB 46.175999 15.082000 34.729000

HB2 45.748001 14.406000 35.464001

HB3 46.751999 14.489000 34.014999

CG 47.084999 16.066999 35.418999

OD1 46.856998 17.247999 35.574001

ND2 48.131001 15.524000 35.949001

HD21 48.714001 16.010000 36.617001

HD22 48.245998 14.541000 35.755001

C 43.838001 16.076000 35.028999

O 44.049999 16.301001 36.222000

N 42.622002 16.173000 34.488998

H 42.563000 15.979000 33.492001

CA 41.368000 16.527000 35.167000

HA 41.418999 16.212999 36.214001

CB 41.173000 18.054001 35.130001

HB2 41.390999 18.412001 34.129002

HB3 40.132999 18.285999 35.360001

CG 42.039001 18.825001 36.131001

HG2 41.830002 18.445999 37.130001

HG3 43.096001 18.683001 35.914001

CD 41.717999 20.323999 36.085999

HD2 40.639000 20.454000 36.193001

HD3 42.208000 20.802000 36.935001

NE 42.189999 20.950001 34.835999

HE 42.651001 20.351000 34.157001

CZ 42.118000 22.223000 34.498001

NH1 41.576000 23.135000 35.249001

HH11 41.175999 22.892000 36.151001

HH12 41.631001 24.100000 34.946999

NH2 42.629002 22.631001 33.377998

HH21 43.137001 21.982000 32.794998

HH22 42.598000 23.618999 33.150002

C 40.169998 15.825000 34.498001

O 40.276001 15.271000 33.403000

N 39.011002 15.912000 35.145000

H 39.056999 16.400000 36.028000

CA 37.678001 15.464000 34.685001

HA 37.632999 15.404000 33.596001

CB 37.429001 14.049000 35.257999

HB2 36.484001 13.638000 34.909000

HB3 38.202999 13.384000 34.882999

CG 37.438000 13.959000 36.784000

HG2 38.396000 14.256000 37.194000

HG3 36.713001 14.670000 37.181000

CD 37.250999 12.535000 37.328999

OE1 37.412998 11.511000 36.688999

NE2 36.901001 12.384000 38.573002

HE21 36.914001 11.460000 38.981998

HE22 36.817001 13.216000 39.139000

C 36.535999 16.475000 34.987000

O 36.256001 16.853001 36.130001

N 35.881001 16.974001 33.931000

H 36.118999 16.670000 32.995998

CA 34.643002 17.728001 34.092999

HA 34.719002 18.393000 34.957001

CB 34.303001 18.594000 32.875000

HB 33.706001 17.987000 32.199001

CG2 33.456001 19.746000 33.394001

HG21 32.661999 19.350000 34.019001

HG22 34.111000 20.379000 33.997002

HG23 32.977001 20.261000 32.561001

CG1 35.570999 19.080000 32.145000

HG12 36.187000 19.666000 32.831001

HG13 36.154999 18.225000 31.832001

CD1 35.293999 19.846001 30.861000

HD11 34.812000 19.226999 30.099001

HD12 34.652000 20.676001 31.138000

HD13 36.249001 20.174999 30.454000

C 33.490002 16.759001 34.387001

O 33.370998 15.688000 33.793999

N 32.629002 17.153999 35.327000

H 32.771000 18.059999 35.759998

CA 31.447001 16.400999 35.796001

HA 31.177999 15.607000 35.099998

CB 31.791000 15.710000 37.116001

HB2 32.306999 16.415001 37.778000

HB3 30.849001 15.356000 37.541000

CG 32.754002 14.511000 36.959999

HG2 33.627998 14.777000 36.368000

HG3 33.148998 14.294000 37.950001

CD 32.144001 13.217000 36.387001

HD2 31.263000 12.968000 36.963001

HD3 31.933001 13.321000 35.319000

CE 33.133999 12.076000 36.589001

HE2 34.022999 12.417000 36.062000

HE3 33.360001 12.046000 37.655998

NZ 32.606998 10.771000 36.104000

HZ1 32.591999 10.708000 35.092999

HZ2 33.146999 9.974000 36.419998

HZ3 31.658001 10.634000 36.438000

C 30.219999 17.334000 35.945000

O 30.405001 18.514000 36.280998

N 29.007000 16.818001 35.674000

H 28.944000 15.833000 35.452999

CA 27.723000 17.503000 35.862000

HA 27.891001 18.577999 35.909000

CB 26.836000 17.266001 34.644001

HB1 25.878000 17.778000 34.737999

HB2 27.315001 17.625999 33.737000

HB3 26.621000 16.205999 34.598000

C 26.947001 17.013000 37.113998

O 27.056999 15.863000 37.542999

N 26.115000 17.881001 37.691002

H 26.103001 18.823000 37.306000

CA 25.214001 17.605000 38.860001

HA 25.652000 16.900999 39.568001

CB 25.017000 18.879000 39.666000

HB2 24.611000 18.625999 40.643002

HB3 25.959999 19.396999 39.818001

OG 24.059000 19.719000 39.083000

HG 23.363001 19.729000 39.758999

C 23.837999 17.035999 38.519001

O 23.075001 16.733000 39.438999

N 23.525000 16.912001 37.224998

H 24.229000 17.142000 36.547001

CA 22.226000 16.531000 36.657001

HA 21.712000 15.960000 37.424999

CB 21.462999 17.851999 36.333000

HB2 20.409000 17.659000 36.122002

HB3 21.452999 18.379000 37.282001

CG 22.028000 18.736000 35.195000

CD1 23.195000 19.530001 35.370998

HD1 23.716999 19.652000 36.312000

CE1 23.719999 20.228001 34.283001

HE1 24.575001 20.878000 34.390999

CZ 23.257999 19.980000 32.993999

HZ 23.798000 20.504999 32.222000

CE2 22.138000 19.134001 32.757999

HE2 21.840000 18.853001 31.747000

CD2 21.497000 18.562000 33.889000

HD2 20.673000 17.867001 33.783001

C 22.341000 15.615000 35.419998

O 23.260000 15.831000 34.639000

N 21.427000 14.660000 35.188999

H 20.690001 14.534000 35.868999

CA 21.367001 13.831000 33.966000

HA 22.358999 13.394000 33.834999

CB 20.259001 12.755000 34.094002

HB2 19.318001 13.268000 34.310001

HB3 20.129999 12.180000 33.167000

CG 20.587999 11.742000 35.191002

HG2 21.400999 11.124000 34.818001

HG3 20.841000 12.226000 36.134998

CD 19.406000 10.804000 35.372002

HD2 18.521999 11.335000 35.730999

HD3 19.195000 10.380000 34.395000

CE 19.681000 9.557000 36.206001

HE2 18.858000 8.890000 35.981998

HE3 20.582001 9.053000 35.848000

NZ 19.656000 9.761000 37.660000

HZ1 20.384001 10.417000 37.931000

HZ2 18.726999 10.049000 37.943001

HZ3 19.823999 8.906000 38.171001

C 21.032000 14.687000 32.743000

O 21.802999 14.691000 31.743999

OXT 19.947001 15.316000 32.764000

ZN 35.644001 36.504002 35.926998

O1 37.509998 36.007999 36.323002

H1 38.056999 36.825001 36.484001

H2 38.069000 35.396000 35.766998

Cl- 18.551001 28.125999 22.837999

O 35.544998 42.633999 46.708000

H1 34.730000 42.233002 47.056000

H2 35.655998 43.379002 47.355000

O 40.108002 37.150002 31.893000

H1 40.721001 36.570000 32.383999

H2 39.287998 36.606998 31.750999

O 11.167000 27.872000 56.980999

H1 10.421000 27.427999 56.521999

H2 11.710000 27.090000 57.223999

O 46.766998 28.164000 44.938000

H1 46.625999 28.958000 45.492001

H2 47.554001 28.423000 44.426998

O 33.595001 52.342999 54.250999

H1 32.768002 52.619999 54.694000

H2 33.412998 51.403000 54.076000

O 51.821999 32.035999 63.653999

H1 51.757000 32.698002 64.358002

H2 51.925999 31.209000 64.168999

O 41.492001 45.578999 57.238998

H1 41.841999 45.903999 58.091000

H2 40.651001 45.166000 57.544998

O 66.260002 24.672001 28.468000

H1 67.127998 24.257000 28.580999

H2 65.990997 24.344000 27.593000

O 25.878000 61.987999 49.019001

H1 26.346001 62.844002 49.033001

H2 25.823999 61.817001 48.062000

O 50.633999 5.177000 37.176998

H1 51.515999 5.416000 37.528999

H2 50.666000 4.205000 37.250999

O 43.685001 48.898998 45.766998

H1 43.146000 49.570999 46.240002

H2 44.543999 49.402000 45.723999

O 47.881001 50.764999 14.207000

H1 48.236000 50.047001 13.651000

H2 48.620998 51.394001 14.219000

O 31.714001 43.457001 34.965000

H1 31.055000 44.103001 34.666000

H2 31.837999 43.728001 35.900002

O 53.705002 24.315001 29.076000

H1 53.282001 24.983999 28.496000

H2 53.014000 23.618000 29.076000

O 30.417999 16.938000 54.327999

H1 30.879999 16.433001 55.035000

H2 30.469999 16.323000 53.588001

O 49.625000 25.135000 54.991001

H1 50.347000 25.708000 54.676998

H2 50.035000 24.261000 54.966999

O 8.351000 38.480999 18.679001

H1 8.596000 38.214001 17.774000

H2 8.362000 37.633999 19.146999

O 12.674000 52.528000 44.376999

H1 13.069000 53.285999 44.860001

H2 13.349000 52.394001 43.681000

O 29.568001 40.383999 23.600000

H1 30.007999 39.632999 23.136999

H2 29.228001 39.909000 24.392000

O 27.813000 29.797001 19.801001

H1 27.318001 30.601000 20.049999

H2 27.346001 29.528999 18.992001

O 64.875999 36.257999 41.966999

H1 65.295998 35.688000 42.623001

H2 63.926998 36.123001 42.152000

O 28.975000 35.668999 15.736000

H1 28.754000 34.981998 15.067000

H2 29.815001 35.306000 16.077000

O 7.750000 30.211000 39.237999

H1 7.189000 30.650000 38.583000

H2 8.292000 29.628000 38.683998

O 24.483000 29.429001 32.824001

H1 24.726000 28.514000 33.074001

H2 23.593000 29.496000 33.220001

O 32.667999 12.889000 43.216999

H1 31.771999 12.505000 43.108002

H2 32.544998 13.353000 44.077999

O 36.948002 12.901000 49.473000

H1 36.327999 12.867000 50.221001

H2 37.791000 13.077000 49.928001

O 43.176998 33.028999 66.700996

H1 43.890999 32.686001 67.271004

H2 43.606998 33.057999 65.836998

O 15.351000 45.584000 59.751999

H1 14.639000 46.257000 59.783001

H2 14.919000 44.872002 59.250000

O 53.384998 29.358000 43.139000

H1 53.096001 28.608000 42.608002

H2 53.331001 29.018000 44.043999

O 44.167000 32.306999 22.202000

H1 45.138000 32.410999 22.041000

H2 44.178001 31.441999 22.663000

O 21.753000 34.172001 53.564999

H1 21.454000 34.144001 54.486000

H2 22.375999 34.923000 53.576000

O 29.886999 58.188000 46.484001

H1 29.410999 57.567001 47.089001

H2 29.469000 57.965000 45.636002

O 37.458000 18.360001 63.207001

H1 37.438000 18.212999 62.237999

H2 38.408001 18.323999 63.386002

O 14.598000 6.507000 36.301998

H1 14.395000 6.123000 35.432999

H2 15.134000 7.277000 36.055000

O 32.528000 11.431000 14.119000

H1 33.088001 11.683000 14.875000

H2 31.954000 12.211000 14.035000

O 11.991000 42.782001 45.352001

H1 11.070000 42.577000 45.592999

H2 11.897000 42.949001 44.391998

O 63.402000 49.049000 23.233000

H1 63.402000 48.748001 24.157000

H2 64.342003 48.998001 23.002001

O 57.966000 31.268000 24.506001

H1 57.544998 31.350000 23.638000

H2 57.998001 32.186001 24.812000

O 55.639999 40.846001 50.020000

H1 55.041000 41.583000 50.242001

H2 55.622002 40.327999 50.845001

O 63.941002 33.104000 41.483002

H1 63.361000 33.838001 41.730999

H2 64.801003 33.398998 41.848999

O 29.299999 8.064000 33.264000

H1 28.358000 8.234000 33.417999

H2 29.281000 7.512000 32.459000

O 23.632000 49.941002 62.632000

H1 23.653999 50.236000 63.563999

H2 24.003000 50.719002 62.181000

O 36.668999 0.911000 38.873001

H1 36.842999 1.741000 38.383999

H2 37.548000 0.484000 38.831001

O 9.903000 32.327000 23.480000

H1 9.639000 31.708000 22.791000

H2 10.612000 32.833000 23.056000

O 59.537998 25.181000 42.929001

H1 59.362000 25.820000 42.206001

H2 60.512001 25.313999 43.032001

O 30.740999 2.213000 20.851999

H1 31.047001 1.510000 20.247999

H2 31.066999 1.880000 21.712000

O 30.016001 29.474001 61.492001

H1 30.209999 30.377001 61.801998

H2 30.468000 29.469000 60.629002

O 13.974000 40.176998 52.014999

H1 14.606000 39.837002 52.665001

H2 14.582000 40.460999 51.293999

O 11.704000 47.237999 21.018999

H1 12.000000 48.161999 21.063999

H2 11.778000 47.043999 20.072001

O 11.576000 12.896000 41.046001

H1 10.688000 13.041000 40.679001

H2 11.841000 12.062000 40.629002

O 21.495001 56.751999 45.209999

H1 22.363001 57.091999 44.914001

H2 21.087000 57.547001 45.580002

O 42.583000 41.022999 59.589001

H1 42.069000 41.675999 60.091999

H2 43.430000 41.497002 59.466999

O 64.602997 29.934999 32.082001

H1 65.431000 30.389000 31.879999

H2 64.639000 29.184999 31.436001

O 16.663000 14.109000 45.426998

H1 17.464001 13.936000 44.876999

H2 16.973000 14.838000 45.981998

O 49.641998 32.680000 53.643002

H1 50.360001 33.247002 53.275002

H2 49.863998 31.820000 53.244999

O 41.487000 50.601002 50.426998

H1 41.345001 50.041000 51.208000

H2 40.633999 50.509998 49.959999

O 16.013000 8.364000 25.105000

H1 15.118000 8.687000 24.892000

H2 16.565001 9.092000 24.756001

O 48.612000 62.360001 40.053001

H1 49.410000 62.480999 39.516998

H2 47.916000 62.688000 39.474998

O 40.612000 57.066002 17.257999

H1 41.539001 57.181000 17.034000

H2 40.243999 56.634998 16.465000

O 54.807999 13.140000 21.924000

H1 55.479000 12.473000 22.146000

H2 54.016998 12.581000 21.812000

O 34.442001 8.734000 52.105000

H1 34.337002 8.887000 53.056000

H2 34.750999 7.818000 52.070999

O 18.746000 6.985000 46.937000

H1 19.052000 7.140000 47.846001

H2 18.809999 6.013000 46.876999

O 26.224001 55.917999 51.140999

H1 27.070999 56.375999 51.263000

H2 26.263000 55.671001 50.202000

O 47.223999 47.761002 33.674000

H1 47.890999 48.459000 33.487000

H2 46.416000 48.209000 33.351002

O 15.111000 52.388000 51.026001

H1 14.188000 52.125000 50.848999

H2 15.378000 51.733002 51.695000

O 31.672001 17.990999 9.206000

H1 31.797001 18.191999 8.260000

H2 31.370001 17.065001 9.162000

O 30.750999 4.247000 40.186001

H1 30.791000 4.594000 39.278999

H2 31.573000 4.572000 40.574001

O 43.796001 63.387001 40.244999

H1 43.641998 63.094002 39.328999

H2 44.035999 62.547001 40.681999

O 11.955000 23.575001 49.676998

H1 11.383000 23.229000 50.375999

H2 12.591000 24.106001 50.191002

O 32.389000 34.292999 11.835000

H1 32.745998 33.758999 11.118000

H2 31.573999 34.653999 11.428000

O 18.860001 63.798000 30.511000

H1 19.347000 63.485001 29.728001

H2 19.228001 64.679001 30.653000

O 20.972000 46.680000 60.347000

H1 20.229000 46.196999 59.953999

H2 20.584999 47.548000 60.522999

O 37.556000 27.714001 9.397000

H1 37.174999 28.506001 9.791000

H2 38.080002 27.349001 10.147000

O 22.150999 6.155000 33.688000

H1 22.641001 6.235000 34.528999

H2 22.700001 5.539000 33.188000

O 16.931000 27.849001 56.789001

H1 16.888000 28.746000 56.401001

H2 17.657000 27.455000 56.257000

O 8.044000 46.451000 39.136002

H1 7.331000 46.925999 39.578999

H2 7.765000 45.520000 39.238998

O 45.556999 21.909000 18.465000

H1 46.473000 21.639999 18.271999

H2 45.638000 22.209000 19.393000

O 48.209000 28.806999 59.570999

H1 47.598000 29.480000 59.929001

H2 48.605000 29.284000 58.820000

O 16.139000 42.340000 42.950001

H1 16.483999 42.682999 43.792999

H2 16.962999 42.066002 42.504002

O 35.872002 14.405000 40.324001

H1 34.955002 14.064000 40.308998

H2 36.014999 14.494000 41.292000

O 50.004002 32.256001 18.084000

H1 50.160000 32.671001 18.938999

H2 50.466000 31.402000 18.180000

O 47.028999 31.954000 19.150999

H1 46.778000 31.860001 18.219999

H2 47.992001 31.884001 19.115999

O 44.130001 44.521999 63.959000

H1 43.887001 44.676998 64.883003

H2 43.626999 43.701000 63.763000

O 45.340000 27.921000 61.405998

H1 44.834000 28.621000 60.973000

H2 45.400002 27.247000 60.702000

O 27.472000 13.259000 56.307999

H1 27.065001 12.875000 57.098999

H2 28.413000 13.261000 56.541000

O 42.730000 42.657001 46.278000

H1 42.235001 43.264999 45.703999

H2 43.638000 42.773998 45.965000

O 49.667000 21.229000 52.644001

H1 50.349998 21.618999 52.096001

H2 48.867001 21.316000 52.087002

O 36.757000 29.163000 20.618999

H1 35.841000 28.914000 20.358000

H2 36.876999 28.617001 21.414000

O 27.608000 60.106998 50.000999

H1 26.943001 60.714001 49.602001

H2 27.855000 60.638000 50.792000

O 36.129002 11.243000 56.005001

H1 35.518002 10.993000 55.282001

H2 35.729000 10.753000 56.751999

O 39.219002 18.263000 13.376000

H1 39.655998 19.087999 13.140000

H2 38.599998 18.139000 12.633000

O 25.761000 65.772003 44.604000

H1 25.677000 66.593002 45.115002

H2 26.650999 65.466003 44.903999

O 12.119000 33.320000 37.488998

H1 11.772000 34.042000 36.930000

H2 12.019000 32.556000 36.884998

O 4.157000 42.424999 35.025002

H1 4.268000 43.208000 35.590000

H2 3.845000 41.761002 35.673000

O 17.728001 54.617001 16.638000

H1 17.327000 53.730999 16.531000

H2 17.226000 54.953999 17.402000

O 34.055000 10.306000 54.488998

H1 33.178001 9.996000 54.792999

H2 33.813999 10.796000 53.685001

O 10.901000 23.587999 29.587000

H1 11.166000 22.687000 29.820999

H2 11.757000 24.007000 29.393999

O 25.743000 5.330000 54.630001

H1 26.650000 5.233000 54.300999

H2 25.333000 4.502000 54.344002

O 21.598000 14.393000 49.130001

H1 20.673000 14.116000 49.229000

H2 21.541000 15.350000 49.249001

O 39.507999 44.216000 8.217000

H1 39.403999 43.617001 7.457000

H2 39.137001 43.665001 8.937000

O 45.674999 14.890000 30.236000

H1 45.158001 14.234000 30.736000

H2 46.355999 14.324000 29.820999

O 29.714001 55.181999 51.803001

H1 29.070000 55.622002 51.229000

H2 29.625999 54.245998 51.528000

O 48.709999 24.193001 51.234001

H1 49.522999 23.819000 50.852001

H2 48.394001 23.471001 51.794998

O 29.243000 57.144001 27.237000

H1 29.906000 57.737000 27.653999

H2 28.555000 57.794998 26.996000

O 32.842999 15.857000 12.452000

H1 32.727001 15.201000 13.171000

H2 33.685001 16.263000 12.682000

O 41.763000 25.416000 61.731998

H1 42.398998 25.813999 62.348999

H2 40.938999 25.436001 62.242001

O 48.855999 8.406000 34.525002

H1 48.229000 7.764000 34.111000

H2 48.226002 9.131000 34.737000

O 31.631001 54.639999 20.993999

H1 31.573000 55.599998 21.118000

H2 30.988001 54.317001 21.664000

O 65.081001 26.860001 35.363998

H1 64.612999 26.083000 35.688000

H2 64.362999 27.469000 35.132000

O 43.278000 18.746000 52.462002

H1 43.813999 18.306000 51.776001

H2 43.161999 18.039000 53.112000

O 65.588997 49.923000 31.100000

H1 65.331001 49.241001 31.753000

H2 66.223999 49.417999 30.554001

O 42.589001 39.331001 20.171000

H1 41.863998 39.304001 20.837999

H2 42.682999 38.358002 20.007999

O 57.063999 40.118000 24.584999

H1 56.110001 40.174999 24.813000

H2 57.404999 40.907001 25.058001

O 44.702000 40.036999 42.158001

H1 45.668999 40.096001 42.033001

H2 44.473999 40.997002 42.116001

O 63.736000 52.053001 31.105000

H1 63.388000 51.780998 30.245001

H2 64.545998 51.513000 31.172001

O 16.104000 40.151001 21.434999

H1 16.368999 39.238998 21.226000

H2 16.319000 40.209999 22.382999

O 31.419001 22.684999 16.127001

H1 32.076000 22.115999 15.689000

H2 31.419001 23.466000 15.556000

O 36.418999 24.691999 11.113000

H1 36.838001 25.541000 11.330000

H2 36.445000 24.233999 11.971000

O 34.200001 43.622002 51.554001

H1 34.243000 43.316002 52.485001

H2 33.358002 43.192001 51.276001

O 39.848000 33.757000 64.042000

H1 40.334999 34.116001 64.797997

H2 40.122002 32.827999 64.039001

O 25.794001 64.224998 42.143002

H1 26.608999 64.043999 42.654999

H2 25.349001 64.874001 42.709999

O 26.441000 61.612999 31.441000

H1 25.797001 61.407001 30.738001

H2 25.941999 62.290001 31.951000

O 20.358000 12.140000 38.766998

H1 20.768999 12.636000 39.501999

H2 20.278999 12.845000 38.096001

O 38.778000 16.487000 21.101999

H1 38.923000 16.181000 22.007000

H2 39.256001 15.832000 20.575001

O 25.090000 65.879997 36.358002

H1 25.639000 65.255997 36.862999

H2 25.528999 66.721001 36.514000

O 39.374001 22.830999 43.207001

H1 38.695999 23.487000 43.424000

H2 40.097000 23.381001 42.862000

O 22.646000 52.977001 46.444000

H1 23.214001 53.764999 46.549000

H2 22.153000 52.980000 47.278000

O 20.825001 38.570000 16.906000

H1 21.094000 38.797001 17.805000

H2 21.664000 38.584999 16.419001

O 53.956001 8.201000 39.452999

H1 54.130001 9.135000 39.261002

H2 52.986000 8.154000 39.383999

O 46.665001 19.440001 48.685001

H1 47.168999 20.254000 48.811001

H2 46.751999 19.003000 49.544998

O 20.233000 62.995998 34.535999

H1 19.343000 63.286999 34.280998

H2 20.450001 63.632999 35.247002

O 0.885000 39.754002 29.337999

H1 1.574000 39.078999 29.233999

H2 0.078000 39.217999 29.400000

O 36.564999 11.063000 27.455999

H1 35.793999 10.539000 27.209000

H2 36.445000 11.870000 26.905001

O 46.803001 49.431000 9.083000

H1 46.035000 49.027000 8.639000

H2 46.779999 50.335999 8.742000

O 37.937000 64.778000 25.679001

H1 38.238998 65.470001 25.077999

H2 37.783001 64.028999 25.094999

O 46.862999 10.849000 28.080999

H1 45.909000 10.693000 28.249001

H2 46.933998 10.525000 27.157000

O 7.251000 36.596001 48.187000

H1 6.520000 36.278000 48.750000

H2 8.026000 36.238998 48.637001

O 19.427999 16.884001 18.715000

H1 18.737000 16.316000 19.114000

H2 20.232000 16.368000 18.913000

O 63.221001 28.566000 26.181999

H1 62.571999 28.576000 26.900000

H2 62.737000 28.122999 25.468000

O 49.560001 51.665001 46.137001

H1 50.293999 52.215000 46.459000

H2 49.183998 51.324001 46.966999

O 48.122002 61.118000 42.351002

H1 48.433998 61.368999 41.451000

H2 48.743000 61.631001 42.900002

O 36.980000 22.410000 53.590000

H1 36.800999 21.815001 52.835999

H2 37.925999 22.601000 53.452999

O 21.094999 9.116000 25.872000

H1 20.417000 9.624000 25.403999

H2 20.879999 8.203000 25.608999

O 13.712000 27.360001 20.539000

H1 13.663000 26.686001 19.833000

H2 12.777000 27.645000 20.584000

O 22.372999 8.327000 21.879999

H1 21.997999 8.766000 22.653999

H2 23.277000 8.124000 22.180000

O 8.620000 26.445000 33.499001

H1 7.966000 26.318001 34.207001

H2 9.339000 25.857000 33.785000

O 5.938000 36.845001 44.903999

H1 6.478000 36.097000 45.209000

H2 5.833000 36.633999 43.957001

O 24.069000 13.420000 54.332001

H1 24.055000 13.103000 53.411999

H2 24.688000 12.774000 54.737999

O 62.798000 29.252001 43.310001

H1 62.589001 29.818001 42.549999

H2 63.368000 28.577000 42.921001

O 33.637001 52.972000 50.074001

H1 33.771000 52.688000 49.152000

H2 33.174999 52.192001 50.445000

O 23.683001 62.136002 42.325001

H1 23.337000 62.174000 41.414001

H2 24.288000 62.894001 42.339001

O 30.273001 17.631001 12.239000

H1 31.188000 17.386999 12.450000

H2 30.018000 16.944000 11.606000

O 56.404999 38.637001 44.119999

H1 55.458000 38.867001 44.174999

H2 56.806000 39.512001 43.987000

O 16.896000 3.057000 44.162998

H1 17.872000 3.055000 44.105000

H2 16.677999 3.811000 43.577000

O 32.577000 27.809000 15.520000

H1 32.575001 28.781000 15.569000

H2 31.983999 27.570000 16.249001

O 53.583000 41.766998 20.298000

H1 52.804001 42.252998 19.979000

H2 53.959000 42.404999 20.931999

O 50.034000 20.402000 19.927000

H1 49.367001 20.452999 19.222000

H2 50.820999 20.112000 19.441000

O 27.180000 52.490002 11.336000

H1 27.759001 53.259998 11.122000

H2 27.587999 52.188000 12.168000

O 45.009998 15.073000 40.926998

H1 45.702000 15.309000 40.292999

H2 45.485001 15.051000 41.765999

O 21.028000 20.048000 48.599998

H1 20.575001 20.865000 48.882000

H2 21.796000 20.393000 48.126999

O 49.692001 37.480000 47.713001

H1 50.424000 37.907001 48.199001

H2 50.021999 37.509998 46.801998

O 45.487999 37.508999 8.900000

H1 45.221001 36.618000 8.619000

H2 45.036999 38.073002 8.252000

O 26.594999 20.315001 23.792000

H1 26.969000 19.725000 24.487000

H2 27.174999 20.096001 23.039000

O 23.171000 65.456001 39.270000

H1 23.187000 64.526001 39.027000

H2 22.839001 65.441002 40.182999

O 49.994999 48.676998 43.077999

H1 50.702999 48.251999 42.555000

H2 50.252998 48.415001 43.985001

O 18.181000 42.152000 64.052002

H1 18.993000 42.014000 64.585999

H2 18.350000 43.022999 63.668999

O 50.587002 40.535999 18.875000

H1 50.574001 40.990002 19.750000

H2 50.229000 39.660999 19.146000

O 61.554001 24.601999 23.888000

H1 62.198002 24.903000 24.556999

H2 62.023998 24.811001 23.061001

O 39.686001 46.744999 16.349001

H1 39.439999 46.480999 17.260000

H2 39.212002 47.578999 16.252001

O 32.123001 45.872002 69.358002

H1 31.474001 45.459999 68.745003

H2 31.645000 46.675999 69.616997

O 19.174999 7.077000 54.605000

H1 19.360001 7.625000 55.389000

H2 18.815001 6.267000 55.022999

O 35.104000 51.566002 16.431000

H1 36.022999 51.728001 16.158001

H2 35.228001 51.078999 17.261000

O 26.358000 2.523000 23.912001

H1 26.405001 3.287000 24.525000

H2 26.056000 1.821000 24.525999

O 14.732000 21.379000 53.785999

H1 15.091000 20.563000 53.377998

H2 14.970000 22.049999 53.137001

O 46.949001 42.523998 60.344002

H1 47.203999 41.596001 60.514999

H2 46.893002 42.873001 61.249001

O 45.776001 49.229000 11.881000

H1 46.585999 49.110001 11.359000

H2 45.151001 48.647999 11.411000

O 51.945999 26.872999 54.886002

H1 52.477001 27.497000 54.355999

H2 52.467999 26.816000 55.702999

O 13.060000 43.562000 29.736000

H1 12.844000 42.646000 29.471001

H2 13.319000 43.426998 30.673000

O 57.347000 45.798000 18.462000

H1 56.715000 45.194000 18.034000

H2 56.966999 45.875000 19.357000

O 25.766001 46.067001 66.405998

H1 25.624001 46.959999 66.029999

H2 26.722000 46.041000 66.514000

O 27.993000 7.528000 56.617001

H1 28.124001 8.327000 56.088001

H2 27.090000 7.637000 56.945999

O 45.945999 53.306000 17.927999

H1 46.178001 53.710999 18.788000

H2 46.588001 52.564999 17.900999

O 66.124001 35.367001 39.527000

H1 65.582001 35.395000 40.332001

H2 66.969002 35.021999 39.868000

O 31.757000 2.254000 50.494999

H1 31.122000 1.521000 50.414001

H2 31.834000 2.555000 49.575001

O 32.487000 22.594000 57.563000

H1 33.368000 22.988001 57.424999

H2 32.630001 21.680000 57.258999

O 29.523001 40.417000 66.434998

H1 28.549999 40.500000 66.481003

H2 29.649000 40.171001 65.503998

O 48.729000 53.841999 32.882000

H1 48.206001 54.650002 32.692001

H2 49.430000 54.213001 33.452999

O 5.373000 47.695000 38.851002

H1 5.430000 48.547001 39.325001

H2 5.715000 47.931000 37.973000

O 42.422001 22.648001 14.958000

H1 41.651001 22.764000 15.550000

H2 42.377998 23.476999 14.444000

O 8.563000 32.652000 48.866001

H1 8.978000 32.185001 49.622002

H2 9.222000 33.330002 48.668999

O 60.500999 46.092999 23.648001

H1 60.437000 47.049999 23.853001

H2 61.020000 45.770000 24.407000

O 63.094002 36.755001 35.964001

H1 63.759998 37.433998 36.125999

H2 63.230000 36.152000 36.709999

O 48.055000 23.552000 25.018999

H1 48.154999 23.613001 24.054001

H2 47.101002 23.726999 25.127001

O 27.419001 27.545000 61.056000

H1 27.077999 28.011000 60.284000

H2 26.761999 27.778999 61.740002

O 8.565000 37.825001 50.569000

H1 8.780000 37.284000 51.355000

H2 9.233000 37.509998 49.931000

O 32.598999 44.174000 13.337000

H1 32.192001 43.632000 12.640000

H2 32.698002 43.527000 14.056000

O 30.132999 48.556000 21.309999

H1 30.080999 47.827999 20.667000

H2 31.062000 48.542999 21.565001

O 33.341000 1.932000 29.459000

H1 33.340000 1.004000 29.136000

H2 33.078999 1.803000 30.386999

O 51.952999 49.437000 27.184000

H1 51.226002 49.013000 27.669001

H2 52.011002 50.301998 27.627001

O 62.657001 47.854000 35.775002

H1 63.457001 47.498001 36.219002

H2 61.949001 47.398998 36.271000

O 52.919998 63.229000 32.460999

H1 53.084999 64.079002 32.891998

H2 51.997002 63.345001 32.146000

O 15.794000 34.234001 58.520000

H1 16.716999 34.020000 58.254002

H2 15.941000 34.755001 59.325001

O 56.491001 17.853001 32.511002

H1 55.657001 17.768000 32.014999

H2 56.187000 18.330000 33.310001

O 53.099998 33.556000 55.092999

H1 53.917000 33.752998 55.584000

H2 52.518002 33.231998 55.803001

O 58.081001 51.534000 21.156000

H1 58.389999 50.613998 21.000999

H2 57.361000 51.400002 21.782000

O 44.256001 17.577000 17.518999

H1 43.778000 17.787001 16.705999

H2 45.069000 18.113001 17.417999

O 32.501999 14.627000 14.786000

H1 32.705002 14.095000 15.577000

H2 32.308998 15.497000 15.165000

O 32.380001 15.706000 52.418999

H1 33.063999 16.239000 52.882999

H2 32.469002 16.033001 51.513000

O 3.601000 34.651001 39.214001

H1 3.285000 35.312000 39.849998

H2 3.914000 35.195999 38.477001

O 31.813999 15.734000 20.204000

H1 30.971001 15.288000 20.378000

H2 31.606001 16.658001 20.398001

O 16.451000 35.426998 20.914000

H1 15.750000 35.088001 20.315001

H2 16.527000 34.709000 21.552999

O 7.754000 30.027000 44.789001

H1 6.957000 29.503000 44.570000

H2 7.551000 30.268000 45.720001

O 51.202000 60.816002 25.929001

H1 51.487999 59.901001 25.785999

H2 50.901001 60.791000 26.857000

O 22.875000 32.125999 20.344000

H1 23.198999 31.375999 19.806999

H2 22.750999 32.806000 19.639999

O 1.756000 28.177000 38.787998

H1 2.491000 27.657000 39.139999

H2 1.578000 27.746000 37.939999

O 64.412003 27.153999 41.504002

H1 65.351997 27.115999 41.716000

H2 64.391998 27.719000 40.714001

O 37.985001 65.434998 28.684000

H1 37.973999 64.624001 28.142000

H2 37.071999 65.475998 28.997000

O 15.172000 34.286999 56.000000

H1 15.221000 35.256001 55.886002

H2 15.226000 34.226002 56.983002

O 27.996000 23.971001 66.391998

H1 28.809999 24.327999 65.992996

H2 27.528999 24.777000 66.656998

O 33.111000 62.473999 32.776001

H1 33.866001 62.519001 33.374001

H2 32.471001 63.076000 33.191002

O 29.532000 18.792999 55.894001

H1 30.323999 19.257999 56.229000

H2 29.962000 18.082001 55.358002

O 35.504002 62.950001 20.278000

H1 35.330002 63.578999 20.997999

H2 35.924999 63.547001 19.614000

O 49.436001 38.659000 20.480000

H1 49.220001 39.057999 21.358999

H2 48.561001 38.242001 20.291000

O 21.129000 40.500000 61.834999

H1 21.858999 40.789001 62.397999

H2 20.952999 41.284000 61.294998

O 44.362000 28.968000 65.674004

H1 43.394001 29.076000 65.575996

H2 44.504002 28.077000 65.338997

O 24.667999 34.618000 24.264999

H1 24.361000 34.924999 25.138000

H2 24.333000 33.712002 24.235001

O 42.240002 39.958000 17.542999

H1 42.886002 39.323002 17.174000

H2 42.348999 39.768002 18.507000

O 12.920000 36.276001 21.552999

H1 12.199000 36.918999 21.438999

H2 13.577000 36.790001 22.045000

O 40.351002 18.951000 61.194000

H1 40.410000 18.143000 61.743000

H2 39.598999 18.728001 60.617001

O 20.857000 20.017000 27.302000

H1 21.737000 19.747999 27.614000

H2 20.900999 20.988001 27.393000

O 8.909000 45.936001 28.556000

H1 9.225000 46.464001 29.301001

H2 9.753000 45.589001 28.190001

O 47.375999 18.462000 54.617001

H1 47.703999 19.207001 55.143002

H2 47.661999 17.700001 55.147999

O 38.980999 14.290000 25.583000

H1 39.369999 15.083000 25.973000

H2 39.606998 13.596000 25.847000

O 40.891998 30.565001 68.137001

H1 41.080002 30.980000 68.998001

H2 40.370998 31.270000 67.697998

O 26.865000 10.604000 21.850000

H1 25.913000 10.779000 21.867001

H2 26.941999 9.811000 22.406000

O 8.299000 42.480999 19.347000

H1 7.957000 41.799000 18.746000

H2 8.664000 43.131001 18.725000

O 18.812000 27.408001 28.254999

H1 17.841999 27.291000 28.229000

H2 19.077000 26.649000 28.809999

O 49.568001 33.522999 29.017000

H1 49.433998 32.543999 29.000000

H2 48.933998 33.790001 28.318001

O 54.660999 26.129999 51.654999

H1 54.847000 25.472000 52.337002

H2 53.737000 26.368000 51.840000

O 51.785000 58.333000 28.049999

H1 51.320999 57.924000 27.309999

H2 51.243000 59.123001 28.228001

O 14.333000 50.110001 28.684999

H1 15.259000 50.318001 28.923000

H2 13.883000 50.944000 28.938999

O 41.673000 18.514000 25.820999

H1 41.325001 17.618000 25.893000

H2 40.882000 19.059999 25.997000

O 47.058998 9.104000 42.188000

H1 47.124001 10.047000 41.948002

H2 47.127998 8.673000 41.311001

O 23.448999 54.688000 31.983999

H1 24.127001 54.002998 31.829000

H2 23.941999 55.493000 31.726000

O 55.141998 46.028000 36.953999

H1 55.617001 45.259998 36.588001

H2 54.903999 45.713001 37.842999

O 42.861000 22.285999 51.641998

H1 42.169998 21.691999 51.972000

H2 42.422001 23.163000 51.776001

O 52.938000 41.016998 13.825000

H1 52.476002 40.959999 14.678000

H2 53.347000 40.132999 13.767000

O 34.896999 15.615000 50.952999

H1 35.598999 15.811000 51.584000

H2 34.446999 16.483000 50.873001

O 19.740999 29.079000 61.340000

H1 19.714001 28.107000 61.342999

H2 19.618000 29.285999 60.403000

O 21.929001 2.712000 34.655998

H1 22.794001 3.152000 34.740002

H2 21.624001 2.712000 35.585999

O 18.950001 32.324001 63.630001

H1 19.489000 32.159000 64.429001

H2 19.049000 31.499001 63.146000

O 18.041000 10.739000 43.393002

H1 18.013000 10.860000 42.425999

H2 18.167000 11.651000 43.705002

O 32.324001 60.772999 24.677999

H1 32.334999 59.921001 25.127001

H2 31.806000 61.332001 25.281000

O 50.153999 58.995998 14.632000

H1 50.997002 59.081001 14.146000

H2 50.373001 58.278999 15.262000

O 45.064999 18.417999 58.136002

H1 44.869999 18.841999 58.991001

H2 45.241001 19.184999 57.569000

O 42.446999 58.257999 41.964001

H1 43.118000 57.935001 42.589001

H2 41.841000 57.493000 41.931999

O 24.198000 59.306000 46.571999

H1 25.044001 59.254002 47.071999

H2 24.143000 58.400002 46.217999

O 5.656000 30.263000 35.283001

H1 4.892000 30.129000 34.688000

H2 5.777000 29.362000 35.639000

O 53.856998 16.718000 28.906000

H1 53.645000 16.049999 29.573999

H2 54.733002 17.025000 29.183001

O 38.104000 31.686001 69.886002

H1 38.290001 31.964001 68.970001

H2 37.890999 32.549999 70.302002

O 34.075001 2.620000 20.465000

H1 33.514000 2.572000 21.238001

H2 34.929001 2.291000 20.777000

O 23.870001 47.402000 63.158001

H1 23.827999 48.317001 62.798000

H2 23.112000 46.988998 62.730999

O 51.327999 18.150000 28.054001

H1 52.183998 17.704000 27.941999

H2 51.055000 18.282000 27.122999

O 14.268000 19.195000 44.005001

H1 14.038000 19.607000 44.866001

H2 14.579000 19.964001 43.505001

O 19.377001 8.706000 56.979000

H1 18.879000 9.549000 57.007999

H2 18.976000 8.216000 57.712002

O 43.770000 7.403000 45.025002

H1 43.314999 8.238000 45.243999

H2 43.556999 6.862000 45.803001

O 59.349998 53.936001 26.847000

H1 60.091000 53.417999 27.195000

H2 59.610001 54.056000 25.915001

O 32.154999 2.694000 36.875000

H1 32.460999 1.983000 37.467999

H2 32.990002 3.027000 36.518002

O 18.871000 21.966999 16.535999

H1 19.011000 22.084000 15.574000

H2 19.180000 21.049999 16.660999

O 26.966999 6.668000 36.073002

H1 26.429001 6.109000 36.647999

H2 27.260000 6.018000 35.393002

O 61.639999 44.546001 19.195999

H1 62.061001 45.011002 19.950001

H2 61.806000 43.619999 19.426001

O 23.577999 58.835999 50.165001

H1 24.375999 58.578999 49.689999

H2 23.723000 58.460999 51.049000

O 52.293999 10.291000 33.581001

H1 52.116001 11.186000 33.914001

H2 51.442001 10.078000 33.139000

O 15.209000 17.802999 50.919998

H1 15.324000 18.294001 50.082001

H2 15.686000 16.974001 50.705002

O 11.858000 32.178001 52.312000

H1 11.054000 31.669001 52.136002

H2 12.454000 31.500000 52.678001

O 22.238001 11.040000 57.126999

H1 21.673000 11.795000 57.360001

H2 23.030001 11.469000 56.785000

O 36.228001 41.835999 67.963997

H1 36.223000 42.412998 67.178001

H2 36.710999 41.060001 67.634003

O 36.360001 45.199001 51.667999

H1 35.529999 44.665001 51.549000

H2 36.950001 44.693001 51.070000

O 26.118000 26.504000 66.660004

H1 25.283001 26.937000 66.897003

H2 26.771999 27.125999 67.030998

O 62.187000 21.879000 31.481001

H1 61.549999 21.459000 30.893000

H2 61.803001 22.763000 31.608000

O 28.940001 47.773998 17.743000

H1 28.398001 48.551998 18.006001

H2 28.516001 47.546001 16.888000

O 32.866001 49.480000 53.445999

H1 33.358002 48.654999 53.585999

H2 32.201000 49.438999 54.157001

O 15.196000 31.580999 42.855999

H1 15.178000 30.754000 42.345001

H2 15.722000 32.157001 42.264999

O 25.051001 58.049999 20.114000

H1 25.724001 57.484001 19.683001

H2 24.250999 57.797001 19.622000

O 47.159000 64.882004 20.492001

H1 46.223999 64.891998 20.230000

H2 47.173000 64.125999 21.113001

O 15.551000 46.262001 35.846001

H1 15.089000 47.048000 35.500000

H2 14.803000 45.623001 35.900002

O 67.396004 33.292000 34.924999

H1 67.924004 34.068001 34.661999

H2 68.042999 32.570999 34.807999

O 36.188999 9.079000 43.374001

H1 36.424999 10.012000 43.508999

H2 35.726002 8.869000 44.198002

O 60.787998 28.524000 39.977001

H1 61.597000 28.091999 39.647999

H2 61.111000 29.410000 40.192001

O 25.674000 53.380001 55.215000

H1 25.914000 54.299999 54.976002

H2 25.073000 53.146000 54.480000

O 19.983999 17.796000 60.755001

H1 19.243000 17.624001 60.146999

H2 20.398001 18.573999 60.348999

O 7.849000 35.536999 27.798000

H1 7.618000 35.873001 26.924999

H2 6.989000 35.480999 28.243999

O 48.478001 50.925999 43.808998

H1 49.125000 50.333000 43.391998

H2 48.910999 51.104000 44.671001

O 19.620001 47.922001 63.062000

H1 19.716999 47.127998 63.613998

H2 18.788000 47.712002 62.583000

O 22.992001 50.933998 16.117001

H1 23.763000 51.028999 15.526000

H2 22.739000 51.860001 16.259001

O 46.477001 61.584999 19.858000

H1 47.196999 61.986000 20.384001

H2 45.701000 62.030998 20.240000

O 52.806000 17.042000 24.612000

H1 53.116001 16.516001 23.837999

H2 53.651001 17.372000 24.957001

O 13.345000 10.950000 27.365000

H1 13.524000 10.956000 28.313999

H2 13.629000 10.061000 27.101999

O 41.179001 60.297001 40.480999

H1 40.865002 60.796001 41.273998

H2 41.693001 59.589001 40.925999

O 11.387000 40.053001 42.179001

H1 10.802000 40.658001 41.705002

H2 10.843000 39.772999 42.926998

O 54.247002 20.068001 28.365999

H1 54.555000 19.610001 29.170000

H2 54.391998 19.386999 27.686001

O 11.785000 34.041000 55.236000

H1 11.700000 34.120998 56.195999

H2 10.883000 33.865002 54.949001

O 60.769001 41.713001 22.395000

H1 60.673000 41.962002 23.334000

H2 59.938999 42.058998 22.020000

O 45.560001 38.132999 55.138000

H1 46.027000 38.946999 54.923000

H2 44.650002 38.453999 55.297001

O 42.296001 20.014000 63.692001

H1 41.905998 20.903999 63.647999

H2 41.525002 19.448000 63.556000

O 16.378000 8.008000 48.563000

H1 16.898001 7.700000 47.804001

H2 15.533000 7.557000 48.430000

O 49.380001 28.917000 63.238998

H1 50.243000 28.860001 62.796001

H2 48.800999 29.201000 62.518002

O 58.998001 24.822001 45.761002

H1 58.980999 25.007999 44.801998

H2 59.056999 25.728001 46.125999

O 11.639000 39.424999 57.674999

H1 11.947000 38.994999 56.865002

H2 10.899000 38.841999 57.946999

O 19.361000 24.271000 56.693001

H1 20.232000 23.990000 56.339001

H2 19.510000 24.198999 57.646000

O 21.716999 23.398001 55.590000

H1 22.361000 22.827000 56.046001

H2 22.202999 23.653999 54.794998

O 46.549999 56.280998 18.039000

H1 46.816002 55.400002 18.344000

H2 46.938999 56.339001 17.164000

O 9.678000 33.541000 63.088001

H1 9.419000 32.602001 63.061001

H2 10.648000 33.465000 62.939999

O 59.897999 29.091000 30.781000

H1 60.638000 29.618999 31.136999

H2 59.914001 29.355000 29.843000

O 33.518002 19.554001 11.334000

H1 32.655998 19.655001 10.887000

H2 34.132999 19.868000 10.649000

O 24.937000 61.063000 29.212999

H1 24.535999 61.505001 28.452999

H2 25.867001 60.965000 28.917000

O 47.271000 16.528000 22.441000

H1 47.082001 16.754000 21.518000

H2 48.234001 16.389000 22.420000

O 25.190001 11.703000 52.394001

H1 24.879999 10.786000 52.400002

H2 24.718000 12.081000 51.637001

O 10.513000 47.755001 45.076000

H1 10.059000 46.890999 44.995998

H2 11.351000 47.492001 45.491001

O 26.160000 42.939999 11.446000

H1 26.041000 43.076000 12.403000

H2 25.246000 42.719002 11.178000

O 30.844999 28.618000 12.743000

H1 31.070999 29.393999 13.300000

H2 31.747999 28.302000 12.513000

O 9.607000 31.218000 50.780998

H1 9.313000 30.577999 51.449001

H2 9.883000 30.649000 50.048000

O 37.986000 42.049999 62.980000

H1 38.363998 42.717999 63.594002

H2 38.167999 42.452999 62.117001

O 59.452000 21.697001 21.885000

H1 59.910999 21.657000 21.032000

H2 59.027000 22.563999 21.857000

O 44.476002 39.220001 6.575000

H1 43.657001 38.777000 6.285000

H2 44.119999 39.998001 7.041000

O 35.994999 63.706001 43.034000

H1 36.613998 63.692001 42.291000

H2 35.537998 64.553001 42.911999

O 19.004999 41.122002 57.488998

H1 18.688000 40.199001 57.388000

H2 18.285000 41.618000 57.073002

O 62.424999 33.717999 36.723000

H1 62.556999 34.290001 35.952000

H2 63.292999 33.771000 37.160000

O 28.513000 31.037001 14.515000

H1 27.625999 30.662001 14.383000

H2 28.346001 31.700001 15.215000

O 44.301998 48.485001 17.695999

H1 45.069000 49.013000 17.400000

H2 44.221001 48.773998 18.621000

O 29.879999 13.894000 60.388000

H1 29.677000 14.244000 61.278000

H2 29.330999 13.091000 60.376999

O 37.476002 37.141998 63.650002

H1 37.416000 38.115002 63.554001

H2 37.507000 36.867001 62.715000

O 54.289001 24.672001 35.460999

H1 54.851002 23.903000 35.612000

H2 53.598999 24.336000 34.879002

O 25.427000 47.605999 17.613001

H1 24.591999 47.132999 17.809999

H2 25.264999 48.459000 18.058001

O 31.930000 24.757999 12.716000

H1 31.188999 25.341000 12.494000

H2 32.528000 24.907000 11.951000

O 48.269001 17.172001 19.202000

H1 49.136002 16.882000 19.527000

H2 47.743000 16.349001 19.323000

O 13.678000 59.312000 44.210999

H1 13.409000 60.256001 44.250999

H2 14.464000 59.333000 44.805000

O 25.010000 5.417000 37.970001

H1 24.611000 4.702000 38.505001

H2 25.915001 5.438000 38.320000

O 7.708000 51.915001 31.347000

H1 6.772000 51.727001 31.120001

H2 8.158000 51.583000 30.547001

O 4.433000 35.666000 36.687000

H1 4.719000 35.105000 35.953999

H2 5.255000 36.132999 36.921001

O 34.443001 59.051998 49.189999

H1 34.616001 58.551998 50.002998

H2 33.636002 58.620998 48.856998

O 21.302999 47.985001 66.029999

H1 22.139000 48.178001 66.476997

H2 21.556000 47.980999 65.099998

O 25.216999 36.321999 10.507000

H1 24.677999 35.953999 11.219000

H2 24.577000 36.853001 10.005000

O 35.959000 44.599998 15.623000

H1 36.921001 44.487999 15.772000

H2 35.942001 44.833000 14.676000

O 28.584000 15.701000 14.554000

H1 28.225000 15.066000 15.205000

H2 29.059999 15.102000 13.947000

O 34.209999 36.299999 15.048000

H1 35.035000 36.778000 14.839000

H2 34.055000 35.799999 14.237000

O 37.712002 33.433998 19.323999

H1 38.630001 33.728001 19.521999

H2 37.349998 34.263000 18.940001

O 50.513000 18.868000 22.525999

H1 50.915001 19.743000 22.721001

H2 50.091000 19.049999 21.670000

O 14.156000 34.310001 61.716999

H1 14.732000 34.937000 61.237999

H2 14.821000 33.764000 62.175999

O 43.811001 47.839001 21.101000

H1 43.124001 47.264000 20.724001

H2 43.636002 48.681000 20.629000

O 44.217999 10.404000 28.563999

H1 43.775002 9.875000 27.868000

H2 43.553001 10.373000 29.274000

O 54.181000 63.985001 29.676001

H1 54.067001 63.298000 30.348000

H2 55.102001 63.831001 29.386000

O 46.366001 50.136002 50.332001

H1 45.675999 49.491001 50.083000

H2 46.939999 49.577999 50.893002

O 24.533001 57.236000 56.693001

H1 24.198999 57.306999 57.611000

H2 23.708000 57.199001 56.189999

O 34.852001 64.247002 29.285999

H1 35.653999 63.889999 28.860001

H2 34.498001 63.441002 29.714001

O 14.942000 45.375000 14.033000

H1 14.686000 46.209000 13.597000

H2 14.094000 44.889999 14.029000

O 17.488001 44.742001 34.346001

H1 17.514000 43.838001 34.715000

H2 16.742001 45.133999 34.820999

O 55.278999 52.275002 21.905001

H1 55.505001 52.159000 20.958000

H2 54.938000 53.188000 21.903000

O 50.412998 36.222000 56.122002

H1 50.640999 35.279999 56.174999

H2 49.498001 36.186001 55.791000

O 49.195000 24.315001 45.519001

H1 48.412998 24.836000 45.799999

H2 49.285000 24.583000 44.591999

O 19.631001 53.365002 45.476002

H1 19.900999 53.967999 46.193001

H2 20.042999 53.785999 44.702000

O 47.881001 29.056000 67.952003

H1 47.566002 28.433001 68.628998

H2 48.841000 28.995001 68.057999

O 34.963001 10.154000 32.499001

H1 35.171001 10.268000 33.449001

H2 34.039001 10.447000 32.469002

O 48.304001 51.990002 31.000000

H1 48.369999 52.650002 31.726000

H2 47.613998 52.408001 30.447001

O 15.519000 44.755001 41.966000

H1 15.695000 43.887001 42.382000

H2 15.502000 44.521000 41.023998

O 46.766998 52.223999 11.626000

H1 46.827999 53.104000 12.029000

H2 46.644001 51.639999 12.382000

O 17.959000 13.772000 55.433998

H1 18.767000 13.485000 54.972000

H2 17.586000 12.925000 55.720001

O 24.989000 42.061001 61.908001

H1 24.726999 41.610001 62.730000

H2 24.388000 42.821999 61.898998

O 46.118000 26.016001 23.128000

H1 45.487000 26.528000 23.663000

H2 46.741001 26.698000 22.840000

O 33.262001 66.258003 28.370001

H1 33.683998 66.463997 27.516001

H2 33.840000 65.539001 28.700001

O 26.628000 36.754002 7.781000

H1 27.358000 37.101002 7.249000

H2 27.073999 36.470001 8.589000

O 53.769001 28.691999 25.632999

H1 53.319000 28.955000 24.813000

H2 53.146999 28.997999 26.315001

O 47.833000 13.082000 22.066999

H1 47.384998 12.774000 22.870001

H2 47.548000 13.999000 21.999001

O 43.779999 26.931000 21.464001

H1 43.479000 27.576000 20.806000

H2 44.740002 27.056000 21.452000

O 52.388000 28.386999 58.873001

H1 52.437000 29.073000 58.188999

H2 52.070000 28.881001 59.643002

O 60.706001 24.341999 31.236000

H1 60.889999 25.305000 31.136999

H2 60.004002 24.365000 31.912001

O 43.034000 12.903000 37.463001

H1 43.349998 13.349000 36.662998

H2 42.675999 12.075000 37.106998

O 19.827999 35.535999 21.059000

H1 19.635000 35.084999 20.211000

H2 19.372000 36.377998 20.944000

O 38.312000 66.271004 34.882000

H1 38.465000 65.521004 35.484001

H2 37.398998 66.103996 34.591999

O 6.535000 25.721001 39.362000

H1 6.179000 26.497999 38.877998

H2 7.487000 25.968000 39.395000

O 17.504999 15.566000 20.205000

H1 17.346001 15.605000 21.163000

H2 16.618999 15.388000 19.857000

O 23.319000 7.177000 27.525000

H1 22.667999 6.486000 27.711000

H2 23.084999 7.857000 28.177000

O 15.142000 48.391998 51.897999

H1 15.882000 47.866001 51.556000

H2 15.608000 49.127998 52.340000

O 13.763000 32.675999 17.146000

H1 13.641000 32.384998 18.056000

H2 14.676000 33.001999 17.143999

O 54.452000 45.888000 39.671001

H1 54.230000 46.785999 39.355000

H2 55.020000 46.087002 40.435001

O 38.904999 41.301998 13.813000

H1 39.610001 40.855999 14.315000

H2 39.405998 41.570000 13.006000

O 35.958000 32.534000 62.546001

H1 36.355999 33.424000 62.609001

H2 35.188000 32.706001 61.973999

O 46.825001 10.191000 34.342999

H1 45.872002 10.318000 34.493999

H2 46.905998 10.415000 33.400002

O 26.298000 55.500000 23.708000

H1 25.330999 55.589001 23.607000

H2 26.551001 55.119999 22.849001

O 30.743000 6.583000 47.478001

H1 29.862000 6.841000 47.160999

H2 30.795000 5.655000 47.203999

O 50.859001 33.865002 46.974998

H1 50.075001 34.144001 47.460999

H2 51.426998 34.653999 47.013000

O 29.545000 57.995998 22.754999

H1 29.309000 57.303001 23.389000

H2 30.219999 57.567001 22.211000

O 59.743999 18.740000 28.502001

H1 59.419998 18.006001 29.056999

H2 59.466000 18.437000 27.617001

O 40.668999 16.976000 17.172001

H1 40.591999 15.999000 17.257999

H2 41.473999 17.051001 16.632999

O 51.195999 41.644001 59.087002

H1 51.502998 42.410000 58.561001

H2 52.022999 41.345001 59.490002

O 40.127998 56.327000 19.749001

H1 40.549000 56.556999 18.889999

H2 39.222000 56.647999 19.590000

O 54.959000 34.747002 14.681000

H1 54.595001 34.928001 15.562000

H2 55.870998 35.063000 14.763000

O 51.306999 39.297001 45.780998

H1 51.480999 39.456001 46.736000

H2 50.678001 40.022999 45.595001

O 21.826000 6.652000 40.088001

H1 22.605000 6.174000 40.421001

H2 21.285999 5.933000 39.719002

O 17.186001 62.591999 40.416000

H1 17.937000 63.201000 40.306999

H2 17.517000 61.782001 40.005001

O 19.517000 35.483002 65.509003

H1 20.434999 35.770000 65.332001

H2 19.277000 36.032001 66.264999

O 35.398998 15.653000 14.471000

H1 34.671001 15.053000 14.674000

H2 34.972000 16.520000 14.449000

O 22.457001 58.872002 22.917999

H1 21.648001 58.613998 23.409000

H2 22.096001 59.495998 22.271000

O 23.679001 62.047001 45.085999

H1 23.642000 61.943001 44.115002

H2 24.076000 61.205002 45.361000

O 22.549000 27.364000 65.838997

H1 21.820999 27.419001 65.198997

H2 22.462999 28.209999 66.310997

O 11.423000 22.483000 46.515999

H1 10.645000 23.054001 46.394001

H2 11.403000 22.309000 47.465000

O 36.497002 44.561001 57.860001

H1 36.194000 44.508999 56.943001

H2 36.029999 43.790001 58.257000

O 24.714001 45.931999 10.927000

H1 23.938000 46.514999 11.037000

H2 24.372000 45.092999 11.278000

O 38.818001 31.638000 61.967999

H1 38.372002 30.792000 61.810001

H2 38.070999 32.230000 62.160999

O 16.149000 62.071999 34.637001

H1 15.473000 62.785000 34.612999

H2 16.920000 62.542999 34.271000

O 23.094999 9.811000 54.652000

H1 23.476000 10.699000 54.782001

H2 22.865000 9.569000 55.566002

O 14.144000 36.536999 38.845001

H1 13.518000 35.881001 39.209000

H2 14.180000 37.181999 39.577999

O 41.476002 41.896000 21.983000

H1 41.013000 41.028999 21.988001

H2 41.818001 41.924000 22.891001

O 12.425000 58.094002 25.024000

H1 13.091000 58.459000 25.629000

H2 12.972000 57.507000 24.465000

O 19.403999 57.063000 29.443001

H1 18.736000 56.759998 30.086000

H2 18.955999 56.895000 28.593000

O 63.216000 44.422001 28.358000

H1 63.485001 44.110001 29.249001

H2 63.993000 44.176998 27.833000

O 49.441002 17.927999 49.396000

H1 49.505001 18.336000 50.268002

H2 49.831001 18.620001 48.820999

O 38.660000 39.319000 60.339001

H1 38.973000 40.240002 60.311001

H2 38.959000 38.991001 59.467999

O 23.046000 42.213001 59.526001

H1 22.790001 43.096001 59.834999

H2 22.534000 42.137001 58.695000

O 42.778000 57.618999 44.935001

H1 42.261002 56.790001 45.019001

H2 43.592999 57.259998 44.500999

O 39.465000 66.259003 44.907001

H1 39.215000 65.346001 45.103001

H2 40.049999 66.477997 45.654999

O 5.865000 40.837002 40.681999

H1 5.997000 41.605000 41.280998

H2 5.196000 40.333000 41.167999

O 10.133000 36.941002 45.414001

H1 10.965000 36.710999 44.946999

H2 9.480000 36.786999 44.705002

O 44.728001 61.132000 41.616001

H1 44.327999 60.629002 42.339001

H2 45.673000 61.066002 41.812000

O 48.058998 45.145000 59.171001

H1 47.993999 44.213001 59.429001

H2 47.174999 45.330002 58.827999

O 53.161999 35.584000 46.935001

H1 53.923000 35.082001 47.261002

H2 53.375000 35.695000 45.993000

O 62.105000 35.721001 41.762001

H1 61.243999 35.271999 41.837002

H2 61.951000 36.514999 42.318001

O 39.602001 24.200001 9.876000

H1 38.977001 24.113001 9.132000

H2 39.728001 23.275999 10.139000

O 36.150002 24.493000 15.904000

H1 35.369999 24.958000 15.545000

H2 36.221001 24.860001 16.791000

O 28.562000 8.167000 38.131001

H1 29.177999 7.509000 38.488998

H2 27.823999 7.617000 37.830002

O 22.402000 15.567000 55.446999

H1 22.697001 16.398001 55.026001

H2 23.091999 14.946000 55.154999

O 57.214001 41.050999 41.283001

H1 57.832001 40.365002 41.605999

H2 56.768002 41.314999 42.098999

O 36.012001 45.505001 10.468000

H1 36.258999 44.564999 10.516000

H2 35.794998 45.686001 11.406000

O 48.446999 28.033001 47.715000

H1 47.987999 28.416000 48.491001

H2 48.096001 28.572001 46.993999

O 56.060001 19.228001 34.881001

H1 56.301998 20.163000 34.958000

H2 56.241001 18.910999 35.791000

O 36.615002 55.889999 54.310001

H1 36.603001 55.984001 53.335999

H2 37.577999 55.867001 54.473999

O 26.094999 14.747000 30.084999

H1 25.608999 15.016000 30.884001

H2 25.556000 15.145000 29.382999

O 36.091999 46.955002 59.394001

H1 35.793999 47.472000 58.634998

H2 36.431000 46.146999 58.967999

O 9.375000 40.004002 24.566999

H1 9.112000 40.598000 23.841000

H2 8.653000 39.346001 24.548000

O 35.098999 45.828999 13.135000

H1 35.069000 46.791000 13.284000

H2 34.168999 45.577000 13.258000

O 55.495998 48.605000 43.167000

H1 56.245998 48.153999 42.749001

H2 55.251999 49.257000 42.491001

O 17.093000 14.794000 52.084999

H1 16.990999 14.476000 52.990002

H2 16.438999 14.259000 51.602001

O 16.226999 24.464001 29.801001

H1 16.855000 24.122000 29.128000

H2 16.124001 23.684999 30.375999

O 20.458000 10.405000 23.337999

H1 20.129999 11.249000 22.962999

H2 21.379000 10.633000 23.548000

O 41.780998 50.768002 42.208000

H1 41.771999 51.041000 43.144001

H2 42.346001 51.480000 41.826000

O 33.518002 35.158001 17.375999

H1 33.812000 35.423000 16.479000

H2 33.698002 35.985001 17.872999

O 39.820000 17.004000 41.818001

H1 39.050999 17.429001 41.421001

H2 40.256001 16.597000 41.049999

O 24.183001 15.773000 32.133999

H1 24.093000 15.976000 33.090000

H2 23.304001 15.342000 31.980000

O 39.087002 32.412998 67.343002

H1 38.201000 32.609001 66.967003

H2 39.597000 33.176998 67.004997

O 54.807999 48.485001 27.584999

H1 53.938999 48.816002 27.325001

H2 55.047001 47.908001 26.839001

O 29.337999 37.216999 70.650002

H1 29.409000 37.827000 69.903000

H2 30.200001 37.360001 71.098000

O 57.360001 56.047001 30.403999

H1 57.712002 56.263000 31.290001

H2 58.172001 55.852001 29.915001

O 45.108002 22.077000 15.501000

H1 44.140999 22.059999 15.382000

H2 45.193001 22.282000 16.443001

O 48.081001 36.959000 52.435001

H1 47.905998 37.902000 52.290001

H2 47.209999 36.563999 52.248001

O 47.487000 28.163000 65.240997

H1 48.111000 28.704000 64.731003

H2 47.535000 28.552999 66.124001

O 37.740002 53.334000 13.863000

H1 37.564999 53.160999 12.928000

H2 37.110001 54.042000 14.070000

O 61.950001 45.806999 33.902000

H1 62.160000 46.426998 34.627998

H2 61.028000 45.577000 34.117001

O 57.201000 39.056000 55.266998

H1 56.596001 39.564999 55.835999

H2 57.095001 38.157001 55.636002

O 50.181000 21.649000 43.402000

H1 49.448002 21.783001 44.046001

H2 50.625000 22.507999 43.442001

O 13.261000 38.998001 45.695999

H1 12.379000 39.293999 45.379002

H2 13.330000 39.506001 46.528999

O 12.478000 46.219002 38.895000

H1 11.905000 46.583000 39.587002

H2 12.359000 46.852001 38.169998

O 48.132000 57.709999 37.306000

H1 47.457001 58.398998 37.223999

H2 47.730000 57.092999 37.928001

O 30.297001 61.091999 46.875000

H1 30.563999 61.276001 45.948002

H2 30.021999 60.155998 46.792000

O 41.800999 28.983999 62.242001

H1 40.831001 28.886999 62.265999

H2 41.902000 29.924999 62.037998

O 35.675999 58.958000 41.938000

H1 35.997002 59.812000 41.591999

H2 34.953999 58.761002 41.301998

O 59.830002 37.111000 17.712999

H1 60.240002 36.249001 17.562000

H2 59.833000 37.507999 16.826000

O 26.408001 11.256000 57.973000

H1 26.549000 10.298000 58.081001

H2 26.818001 11.613000 58.771999

O 20.570999 32.603001 16.406000

H1 20.431000 31.820000 16.982000

H2 21.510000 32.777000 16.537001

O 24.851999 38.071999 70.018997

H1 25.315001 38.159000 70.866997

H2 25.386000 38.651001 69.441002

O 16.816000 22.381001 55.219002

H1 15.995000 22.042999 54.813000

H2 16.471001 22.941999 55.930000

O 21.754000 22.415001 42.361000

H1 20.818001 22.455999 42.055000

H2 22.191000 22.973000 41.698002

O 50.058998 34.448002 16.222000

H1 50.542000 34.076000 15.464000

H2 50.173000 33.743999 16.888000

O 10.994000 35.661999 58.162998

H1 10.516000 36.511002 58.235001

H2 11.809000 35.868999 58.674999

O 13.773000 17.688000 39.937000

H1 13.692000 17.059000 40.695000

H2 12.835000 17.806000 39.708000

O 43.018002 45.035000 31.381001

H1 42.431000 45.575001 31.955000

H2 43.639999 44.689999 32.063999

O 25.896000 64.771004 52.556000

H1 25.573999 64.067001 53.140999

H2 25.329000 64.665001 51.776001

O 13.098000 32.574001 57.004002

H1 14.051000 32.737000 57.008999

H2 12.882000 32.723000 56.058998

O 45.368000 24.320000 53.734001

H1 45.695000 25.225000 53.573002

H2 44.453999 24.500000 54.026001

O 14.049000 44.848999 45.436001

H1 13.482000 45.415001 45.993000

H2 13.511000 44.036999 45.381001

O 44.297001 42.617001 55.595001

H1 44.404999 43.286999 56.278999

H2 43.358002 42.655998 55.387001

O 52.925999 51.771000 28.486000

H1 53.883999 51.865002 28.589001

H2 52.591999 52.098000 29.336000

O 42.291000 15.957000 22.191999

H1 42.129002 16.552999 21.437000

H2 43.176998 16.256001 22.479000

O 52.334999 47.575001 41.854000

H1 52.828999 47.895000 41.087002

H2 52.973000 46.952000 42.258999

O 11.516000 37.037998 61.610001

H1 10.750000 36.521999 61.287998

H2 12.233000 36.689999 61.060001

O 35.882000 8.870000 20.139999

H1 36.088001 9.476000 20.891001

H2 35.691002 8.045000 20.631001

O 10.699000 20.645000 27.910000

H1 10.451000 20.659000 28.864000

H2 9.922000 21.101000 27.521000

O 52.462002 20.125999 44.285999

H1 52.705002 21.041000 44.034000

H2 51.507999 20.125000 44.084999

O 26.551001 42.861000 59.908001

H1 25.777000 42.596001 60.451000

H2 27.221001 42.234001 60.237000

O 51.639999 25.693001 59.575001

H1 52.061001 26.570000 59.513000

H2 50.729000 25.931000 59.823002

O 37.245998 12.928000 23.037001

H1 36.443001 13.367000 22.698999

H2 37.613998 13.631000 23.603001

O 12.150000 34.583000 39.948002

H1 11.922000 34.258999 39.055000

H2 11.452000 35.261002 40.081001

O 35.931000 19.750000 16.745001

H1 36.141998 19.337000 17.594000

H2 34.987000 19.558001 16.646000

O 45.181000 32.355000 68.458000

H1 45.495998 31.683001 69.109001

H2 45.911999 32.334000 67.815002

O 43.865002 62.786999 37.562000

H1 43.522999 63.376999 36.866001

H2 44.825001 62.917000 37.480000

O 27.981001 8.660000 27.181999

H1 27.829000 9.157000 26.363001

H2 27.493000 9.202000 27.833000

O 9.140000 19.888000 32.995998

H1 8.884000 18.987000 33.250000

H2 9.019000 20.368999 33.832001

O 13.313000 28.094000 23.341000

H1 13.818000 27.599001 22.680000

H2 13.921000 28.146999 24.087999

O 53.459000 18.018000 34.575001

H1 54.317001 18.476000 34.608002

H2 52.860001 18.736000 34.294998

O 30.077999 32.208000 61.050999

H1 29.834999 32.029999 60.127998

H2 29.739000 33.110001 61.180000

O 47.000999 37.812000 45.087002

H1 47.047001 37.050999 45.717999

H2 47.909000 37.755001 44.708000

O 18.823999 18.621000 21.289000

H1 18.955999 18.063999 20.514000

H2 19.712999 18.739000 21.636999

O 53.890999 45.542000 42.908001

H1 53.560001 44.945000 43.602001

H2 54.854000 45.493999 43.054001

O 46.523998 49.464001 23.105000

H1 45.750999 49.813999 23.603001

H2 47.256001 49.811001 23.657000

O 25.386000 24.535000 57.169998

H1 26.280001 24.485001 56.758999

H2 25.513000 23.968000 57.949001

O 55.459999 35.473000 51.827999

H1 56.153000 34.813000 51.959999

H2 55.959999 36.266998 51.596001

O 45.230000 66.240997 36.221001

H1 44.803001 66.516998 37.056000

H2 45.504002 67.098000 35.856998

O 32.548000 48.999001 11.383000

H1 31.785000 49.592999 11.517000

H2 32.340000 48.285999 12.016000

O 50.681999 45.898998 18.541000

H1 50.669998 46.216000 17.625000

H2 50.049999 46.507999 18.969000

O 22.188000 43.648998 49.333000

H1 22.334999 43.487000 48.387001

H2 21.518000 42.967999 49.546001

O 32.084999 9.730000 42.279999

H1 31.791000 10.415000 41.660000

H2 31.291000 9.568000 42.804001

O 15.374000 59.497002 46.195999

H1 16.195000 59.645000 46.710999

H2 14.716000 59.932999 46.776001

O 51.924999 28.806999 27.829000

H1 51.351002 28.618999 28.597000

H2 51.352001 28.539000 27.094999

O 27.305000 37.935001 15.774000

H1 26.719000 37.411999 16.344999

H2 28.039000 37.303001 15.630000

O 40.594002 12.110000 41.388000

H1 39.629002 12.293000 41.473000

H2 40.958000 12.815000 41.952999

O 25.440001 42.344002 67.871002

H1 25.638000 42.105000 68.792999

H2 25.962000 43.166000 67.772003

O 65.176003 34.213001 24.290001

H1 64.922997 35.150002 24.431999

H2 64.804001 33.798000 25.089001

O 32.316002 15.122000 9.938000

H1 32.347000 15.768000 10.678000

H2 33.187000 14.685000 10.065000

O 58.229000 49.151001 41.688000

H1 58.317001 49.000000 42.664001

H2 58.672001 50.018002 41.613998

O 57.966999 36.073002 49.991001

H1 58.929001 36.020000 49.818001

H2 57.644001 36.438000 49.147999

O 6.367000 47.444000 35.987999

H1 5.942000 48.180000 35.507999

H2 7.252000 47.431000 35.585999

O 47.827000 29.441999 16.629999

H1 46.918999 29.694000 16.405001

H2 48.348000 30.075001 16.105000

O 53.272999 23.046000 53.917999

H1 53.577000 23.770000 54.493000

H2 52.419998 22.820999 54.335999

O 45.271000 60.316002 44.963001

H1 44.845001 61.126999 45.282001

H2 45.165001 59.722000 45.734001

O 51.848000 29.604000 61.870998

H1 51.810001 30.573999 61.845001

H2 52.286999 29.455000 62.734001

O 65.684998 45.472000 29.771000

H1 64.943001 45.756001 30.318001

H2 66.406998 45.383999 30.422001

O 22.316000 56.474998 21.288000

H1 22.514999 57.280998 21.787001

H2 22.840000 56.584999 20.482000

O 46.361000 41.898998 54.188999

H1 46.608002 41.312000 54.922001

H2 45.535999 42.301998 54.551998

O 19.535999 57.323002 40.307999

H1 20.259001 57.941002 40.459000

H2 19.187000 57.589001 39.451000

O 65.969002 38.001999 22.218000

H1 65.785004 38.855999 21.767000

H2 66.033997 37.398998 21.452999

O 20.724001 58.174999 24.931999

H1 20.143000 58.917999 25.166000

H2 20.193001 57.410000 25.197001

O 9.261000 50.275002 29.645000

H1 10.137000 50.042000 29.996000

H2 9.422000 50.264000 28.688999

O 39.577999 28.184000 68.822998

H1 38.945000 28.514000 69.483002

H2 40.000000 29.010000 68.519997

O 26.774000 2.601000 47.509998

H1 26.235001 1.839000 47.227001

H2 26.164000 3.064000 48.103001

O 17.157000 46.918999 46.467999

H1 17.885000 46.590000 47.014000

H2 16.472000 47.123001 47.131001

O 16.664000 32.426998 18.340000

H1 16.252001 31.569000 18.487000

H2 17.607000 32.229000 18.351999

O 13.138000 40.366001 48.144001

H1 12.206000 40.407001 48.410999

H2 13.478000 41.209000 48.504002

O 21.023001 59.782001 45.263000

H1 21.059000 60.397999 44.514000

H2 21.797001 60.009998 45.778999

O 21.209000 54.665001 22.841000

H1 21.548000 53.833000 22.478001

H2 21.562000 55.318001 22.200001

O 36.680000 13.758000 56.560001

H1 36.569000 12.899000 56.095001

H2 37.627998 13.921000 56.431000

O 48.573002 35.316002 65.003998

H1 47.688000 34.977001 64.789001

H2 48.558998 36.175999 64.531998

O 11.354000 43.966000 43.044998

H1 11.782000 43.665001 42.234001

H2 10.902000 44.776001 42.764000

O 31.205000 30.868999 64.191002

H1 31.586000 30.237000 63.560001

H2 31.110001 30.306000 64.986000

O 30.271000 7.973000 44.840000

H1 29.551001 8.174000 45.459999

H2 29.830000 8.121000 43.977001

O 60.852001 37.073002 22.079000

H1 60.457001 36.183998 22.156000

H2 60.115002 37.634998 22.379000

O 12.252000 22.219000 43.745998

H1 12.052000 22.250999 44.701000

H2 11.659000 21.502001 43.453999

O 17.395000 60.660999 47.443001

H1 18.176001 61.243000 47.487000

H2 16.889000 60.966000 48.217999

O 25.520000 51.172001 14.812000

H1 25.593000 50.215000 14.945000

H2 26.157000 51.519001 15.470000

O 38.584000 35.561001 15.536000

H1 38.117001 34.731998 15.699000

H2 37.866001 36.139000 15.204000

O 49.639999 28.525000 54.611000

H1 49.782001 29.120001 53.855000

H2 50.303001 27.830999 54.443001

O 46.388000 15.456000 53.682999

H1 46.785000 15.598000 52.811001

H2 45.484001 15.194000 53.464001

O 15.687000 59.508999 24.544001

H1 15.839000 58.833000 23.865999

H2 16.195999 59.167999 25.292000

O 51.369999 15.546000 42.514999

H1 52.116001 15.526000 43.146000

H2 51.139999 14.607000 42.452999

O 65.639999 40.722000 23.686001

H1 66.282997 40.991001 23.000000

H2 65.935997 39.813000 23.867001

O 39.444000 38.417000 20.014000

H1 39.520000 37.703999 19.344000

H2 39.036999 39.123001 19.468000

O 4.342000 45.532001 35.418999

H1 4.761000 45.104000 36.187000

H2 4.911000 46.313000 35.303001

O 5.363000 27.371000 30.943001

H1 5.308000 26.573000 31.483000

H2 6.333000 27.469999 30.829000

O 47.542999 59.102001 14.033000

H1 47.314999 59.786999 14.697000

H2 48.521999 59.077000 14.141000

O 23.476000 55.587002 24.191999

H1 23.254999 56.352001 24.753000

H2 22.587999 55.233002 23.990999

O 29.381001 6.102000 52.176998

H1 28.561001 6.598000 52.027000

H2 30.048000 6.805000 52.176998

O 36.403000 61.151001 50.806000

H1 36.488998 60.272999 50.415001

H2 36.174000 61.703999 50.047001

O 52.976002 36.127998 51.585999

H1 53.903999 35.873001 51.812000

H2 53.102001 37.078999 51.398998

O 29.742001 37.249001 65.935997

H1 30.257999 37.632999 65.213997

H2 30.445000 37.063999 66.599998

O 28.627001 14.902000 19.611000

H1 28.622999 14.816000 20.583000

H2 27.917000 14.284000 19.362000

O 35.620998 20.280001 47.995998

H1 34.751999 20.105000 47.576000

H2 35.514000 19.797001 48.841000

O 16.283001 28.558001 21.181000

H1 16.534000 27.677000 20.813999

H2 15.327000 28.559000 21.000999

O 45.904999 52.964001 33.318001

H1 45.903000 52.223999 32.688999

H2 45.870998 53.730999 32.695999

O 16.368000 24.045000 57.623001

H1 16.257000 24.723000 58.320999

H2 17.105000 23.520000 57.987999

O 54.337002 7.307000 35.645000

H1 53.403999 7.559000 35.514999

H2 54.303001 6.347000 35.484001

O 19.969000 42.117001 53.820000

H1 20.691999 42.244999 53.181000

H2 20.129000 42.842999 54.451000

O 14.856000 9.936000 50.723000

H1 15.329000 10.136000 49.902000

H2 14.108000 9.403000 50.403999

O 64.919998 40.181000 37.146000

H1 64.200996 40.445000 37.743999

H2 65.193001 39.327999 37.536999

O 55.026001 46.896999 45.150002

H1 55.144001 47.497002 44.374001

H2 55.743999 46.257000 44.978001

O 39.537998 33.370998 11.479000

H1 39.897999 32.455002 11.417000

H2 40.280998 33.812000 11.938000

O 46.269001 18.341999 26.621000

H1 46.764000 18.285999 25.778999

H2 46.306999 17.421000 26.930000

O 30.320000 22.620001 12.206000

H1 31.003000 23.240000 12.522000

H2 30.857000 21.913000 11.813000

O 49.575001 7.892000 37.185001

H1 49.838001 6.958000 37.220001

H2 49.358002 8.001000 36.240002

O 24.594000 47.883999 52.765999

H1 25.205000 47.731998 52.027000

H2 25.113001 47.556000 53.523998

O 31.479000 8.144000 20.493999

H1 31.672001 7.854000 19.580999

H2 30.722000 8.730000 20.363001

O 45.452000 64.094002 24.493000

H1 45.101002 64.885002 24.045000

H2 44.848000 64.001999 25.242001

O 38.250999 59.483002 13.664000

H1 39.117001 59.435001 14.127000

H2 38.230999 58.625000 13.210000

O 55.173000 32.119999 51.205002

H1 55.751999 31.403000 50.910999

H2 55.673000 32.470001 51.971001

O 11.825000 38.730999 51.441002

H1 11.926000 37.898998 51.912998

H2 12.618000 39.230000 51.744999

O 65.362000 36.348000 30.818001

H1 65.573997 36.487000 31.750000

H2 64.449997 36.016998 30.858999

O 21.583000 1.757000 31.988001

H1 21.497999 2.620000 31.552999

H2 21.711000 2.021000 32.919998

O 50.535999 44.699001 53.705002

H1 49.653000 44.507000 54.074001

H2 51.042000 44.924000 54.494999

O 9.478000 10.753000 38.527000

H1 9.942000 11.341000 37.902000

H2 9.187000 10.035000 37.953999

O 6.271000 24.035999 44.202999

H1 5.459000 24.540001 43.988998

H2 6.879000 24.382000 43.514999

O 57.495998 27.250000 38.702000

H1 56.683998 27.584000 38.289001

H2 57.771999 27.989000 39.255001

O 24.002001 35.706001 21.993000

H1 24.316000 35.273998 22.818001

H2 23.114000 35.334999 21.908001

O 44.637001 42.750000 41.870998

H1 44.146999 43.476002 42.337002

H2 45.554001 43.091999 41.984001

O 15.393000 55.549999 35.252998

H1 14.994000 56.146999 35.904999

H2 14.611000 55.074001 34.910000

O 14.812000 37.090000 56.273998

H1 14.171000 37.671001 55.821999

H2 14.481000 37.104000 57.185001

O 60.372002 48.681999 30.629000

H1 60.387001 49.521000 30.115000

H2 60.146999 49.006001 31.511000

O 16.612000 54.893002 26.379000

H1 16.653999 54.026001 26.806999

H2 15.648000 55.070000 26.381001

O 46.710999 54.914001 35.077000

H1 47.008999 55.588001 34.429001

H2 46.471001 54.182999 34.474998

O 41.187000 11.673000 16.353001

H1 40.514999 11.156000 16.827000

H2 41.812000 11.882000 17.079000

O 62.459000 30.910999 41.001999

H1 63.167000 30.378000 40.598999

H2 62.870998 31.806000 40.980999

O 7.584000 46.694000 26.287001

H1 8.087000 46.532001 27.108999

H2 7.100000 45.855999 26.188000

O 31.433001 46.895000 12.948000

H1 31.562000 46.018002 13.356000

H2 31.389999 47.464001 13.739000

O 18.353001 19.816000 50.414001

H1 18.787001 19.087999 50.888000

H2 18.987000 20.540001 50.522999

O 14.311000 9.359000 42.719002

H1 14.253000 9.718000 43.613998

H2 13.564000 9.780000 42.269001

O 11.857000 43.283001 54.644001

H1 12.268000 42.404999 54.672001

H2 12.587000 43.852001 54.949001

O 8.653000 47.522999 32.359001

H1 8.830000 46.566002 32.428001

H2 8.847000 47.813000 33.277000

O 50.810001 30.052000 45.301998

H1 50.301998 30.257999 46.101002

H2 51.518002 30.725000 45.342999

O 15.634000 10.203000 35.229000

H1 15.691000 9.475000 34.569000

H2 14.925000 10.744000 34.840000

O 9.791000 38.473999 38.028999

H1 9.520000 38.477001 37.096001

H2 9.553000 39.362999 38.320000

O 59.160000 13.538000 36.088001

H1 58.576000 13.924000 36.759998

H2 59.970001 14.063000 36.206001

O 30.153999 50.028999 12.514000

H1 29.271999 49.606998 12.463000

H2 29.964001 50.783001 13.105000

O 25.844999 35.731998 20.020000

H1 26.650999 36.077000 20.445999

H2 25.219000 35.737000 20.777000

O 45.105000 25.313999 67.262001

H1 45.661999 25.961000 66.803001

H2 45.116001 24.562000 66.656998

O 35.910999 34.057999 23.225000

H1 36.256001 33.528000 23.985001

H2 36.591000 34.761002 23.195999

O 29.337999 24.593000 18.914000

H1 29.419001 23.924000 19.614000

H2 28.409000 24.490999 18.645000

O 39.613998 8.397000 42.473000

H1 38.903999 9.034000 42.647999

H2 39.451000 7.738000 43.186001

O 55.366001 53.431000 16.895000

H1 55.672001 52.507999 16.952000

H2 54.752998 53.487000 17.642000

O 50.424999 44.547001 35.380001

H1 51.006001 43.833000 35.706001

H2 50.108002 44.922001 36.230000

O 39.738998 52.077000 53.466999

H1 40.674000 52.217999 53.699001

H2 39.588001 51.172001 53.770000

O 19.171000 14.925000 27.094999

H1 18.495001 15.322000 27.671000

H2 19.139999 15.517000 26.320000

O 56.220001 35.640999 43.813999

H1 55.273998 35.806999 43.993000

H2 56.550999 36.540001 43.666000

O 19.415001 26.605000 48.717999

H1 18.941999 26.549000 47.881001

H2 19.273001 27.531000 48.980000

O 24.115999 14.446000 41.606998

H1 24.622999 15.258000 41.782001

H2 24.646999 14.021000 40.916000

O 31.636000 54.511002 15.693000

H1 32.036999 54.882000 16.509001

H2 32.380001 53.963001 15.363000

O 48.438000 22.737000 38.062000

H1 48.910999 22.101000 38.613998

H2 48.632000 23.580999 38.528000

O 40.276001 14.362000 16.806000

H1 39.404999 14.462000 16.384001

H2 40.603001 13.543000 16.395000

O 44.291000 53.812000 12.151000

H1 44.610001 53.167999 11.509000

H2 44.683998 53.511002 12.981000

O 37.112999 38.337002 57.527000

H1 37.115002 37.417999 57.223999

H2 38.071999 38.492001 57.666000

O 38.841999 7.271000 17.360001

H1 38.231998 6.786000 17.959999

H2 39.020000 6.573000 16.695999

O 13.071000 6.727000 29.886000

H1 12.459000 6.417000 29.204000

H2 12.773000 6.228000 30.667000

O 27.639999 61.119999 44.341000

H1 27.219000 61.202000 45.209000

H2 28.330999 60.466999 44.492001

O 55.666000 11.515000 30.233999

H1 55.188000 11.111000 30.993999

H2 55.011002 12.192000 29.962000

O 13.267000 38.845001 32.394001

H1 13.914000 39.535000 32.171001

H2 13.379000 38.227001 31.645000

O 55.754002 51.688000 19.395000

H1 55.797001 50.748001 19.181999

H2 56.688000 51.952999 19.360001

O 66.723000 36.209999 26.868000

H1 65.807999 35.917999 26.698000

H2 66.900002 35.742001 27.718000

O 19.639000 52.501999 55.219002

H1 20.316999 53.145000 55.478001

H2 18.907000 52.741001 55.817001

O 31.549000 22.920000 7.763000

H1 31.268000 23.853001 7.766000

H2 32.518002 23.016001 7.724000

O 49.007999 32.563000 60.261002

H1 49.178001 32.944000 61.150002

H2 49.924000 32.373001 59.973000

O 33.327999 5.727000 35.716999

H1 33.198002 6.239000 34.904999

H2 32.779999 6.206000 36.353001

O 56.818001 33.444000 39.404999

H1 57.560001 32.846001 39.185001

H2 56.658001 33.202999 40.337002

O 55.813000 43.778000 16.884001

H1 55.992001 43.813999 15.937000

H2 55.042000 44.375999 16.972000

O 26.024000 29.146000 17.337000

H1 26.459999 29.247000 16.472000

H2 26.079000 28.183001 17.472000

O 29.108000 27.327000 14.697000

H1 29.013000 26.368000 14.784000

H2 29.914000 27.419001 14.171000

O 9.915000 36.824001 64.469002

H1 9.931000 37.783001 64.344002

H2 10.772000 36.551998 64.114998

O 33.039001 59.770000 33.051998

H1 32.446999 59.736000 32.287998

H2 33.320999 60.705002 33.039001

O 6.334000 43.411999 21.077000

H1 7.042000 43.202000 20.443001

H2 5.543000 43.124001 20.607000

O 28.214001 63.203999 42.854000

H1 27.958000 62.382000 43.346001

H2 28.743000 63.654999 43.528000

O 12.301000 40.091000 60.637001

H1 12.029000 40.745998 59.972000

H2 12.688000 39.395000 60.082001

O 38.000000 46.321999 53.601002

H1 38.001999 47.278999 53.787998

H2 37.520000 46.299000 52.750000

O 1.514000 43.598999 29.424999

H1 2.128000 43.881001 30.122000

H2 0.826000 43.141998 29.938000

O 49.213001 43.159000 7.421000

H1 49.305000 43.528999 6.521000

H2 49.953999 43.587002 7.881000

O 51.264000 16.136999 34.881001

H1 50.931999 16.010000 35.776001

H2 52.037998 16.705999 35.014999

O 56.268002 43.930000 35.706001

H1 55.605999 43.331001 35.334999

H2 57.087002 43.403000 35.605000

O 50.007999 9.987000 32.338001

H1 49.381001 9.375000 32.750000

H2 49.431000 10.539000 31.785999

O 13.179000 10.655000 34.143002

H1 13.426000 10.125000 33.358002

H2 12.726000 11.409000 33.722000

O 34.646000 24.105000 56.759998

H1 35.217999 24.475000 57.457001

H2 34.269001 24.927000 56.377998

O 51.778000 37.460999 64.043999

H1 51.849998 38.330002 63.611000

H2 50.921001 37.143002 63.691002

O 41.775002 27.413000 23.344999

H1 42.646000 27.148001 23.006001

H2 41.919998 27.417999 24.305000

O 14.951000 20.242001 26.731001

H1 14.332000 20.607000 27.392000

H2 15.793000 20.638000 27.027000

O 39.473999 30.996000 15.308000

H1 38.825001 31.398001 14.709000

H2 39.966999 31.780001 15.620000

O 14.099000 43.092999 61.262001

H1 13.933000 43.881001 61.792999

H2 13.616000 43.286999 60.443001

O 25.652000 62.459999 39.735001

H1 26.378000 61.846001 39.952000

H2 25.879000 63.230999 40.280998

O 46.118999 37.888000 23.398001

H1 45.317001 38.459999 23.351000

H2 45.719002 37.041000 23.066000

O 47.901001 53.792000 46.416000

H1 48.603001 53.139999 46.229000

H2 48.205002 54.158001 47.270000

O 28.014000 32.707001 11.031000

H1 28.819000 32.237000 11.341000

H2 28.107000 33.557999 11.497000

O 46.877998 44.448002 51.963001

H1 46.130001 44.077999 52.444000

H2 47.348000 43.634998 51.667999

O 7.373000 35.581001 22.677999

H1 6.863000 34.908001 22.188000

H2 7.325000 35.216999 23.587999

O 31.750999 16.018999 56.377998

H1 30.879999 15.893000 56.799999

H2 32.147999 16.691999 56.966000

O 66.816002 39.124001 27.142000

H1 66.231003 38.370998 26.983000

H2 67.188004 38.914001 28.014999

O 65.765999 31.191000 35.835999

H1 66.344002 31.826000 35.376999

H2 65.128998 30.955000 35.148998

O 24.813000 31.267000 66.621002

H1 24.055000 30.754000 66.958000

H2 25.541000 30.921000 67.169998

O 4.891000 23.826000 38.230000

H1 5.450000 24.474001 38.709999

H2 5.331000 22.993999 38.453999

O 11.449000 30.690001 60.587002

H1 11.872000 31.212000 61.293999

H2 11.083000 31.389000 60.021999

O 50.528000 42.833000 38.066002

H1 51.042000 42.499001 37.305000

H2 50.993999 42.412998 38.807999

O 20.045000 4.726000 39.570000

H1 20.263000 3.932000 40.077999

H2 19.087999 4.808000 39.716999

O 25.372999 2.743000 29.229000

H1 26.157000 3.175000 28.863001

H2 24.660999 3.356000 28.972000

O 51.342999 36.558998 19.454000

H1 50.660999 37.033001 18.957001

H2 52.041000 37.241001 19.528999

O 27.750000 24.238001 56.007999

H1 27.886000 23.983999 55.077999

H2 28.660000 24.216999 56.351002

O 18.402000 48.215000 44.550999

H1 18.054001 47.673000 45.292000

H2 18.033001 47.737999 43.786999

O 16.341999 16.851000 38.158001

H1 15.406000 17.049000 38.290001

H2 16.714001 17.021000 39.039001

O 55.233002 20.992001 54.408001

H1 54.712002 21.792000 54.226002

H2 55.712002 21.250000 55.222000

O 42.297001 15.994000 51.668999

H1 41.662998 16.073999 52.397999

H2 41.724998 16.158001 50.891998

O 56.243999 13.967000 27.551001

H1 56.080002 14.344000 26.667000

H2 55.806999 14.611000 28.125999

O 42.207001 11.528000 44.166000

H1 41.592999 12.247000 43.970001

H2 43.058998 11.895000 43.856998

O 35.236000 18.195999 13.024000

H1 34.618000 18.664000 12.430000

H2 36.061001 18.222000 12.513000

O 44.402000 15.349000 44.002998

H1 43.889000 15.439000 43.188999

H2 44.541000 16.284000 44.254002

O 61.683998 37.355999 39.009998

H1 61.520000 36.549999 38.488998

H2 60.823002 37.805000 38.938999

O 19.827999 8.336000 43.568001

H1 20.174000 8.864000 42.813999

H2 18.966999 8.769000 43.706001

O 43.508999 14.898000 48.712002

H1 42.993999 15.701000 48.512001

H2 43.292000 14.344000 47.933998

O 36.094002 57.528000 26.419001

H1 36.813999 57.063999 25.941000

H2 35.757000 58.116001 25.715000

O 17.952000 55.479000 36.115002

H1 18.243999 54.640999 35.723999

H2 16.985001 55.362999 36.143002

O 40.141998 49.772999 56.722000

H1 39.370998 49.214001 56.504002

H2 40.186001 49.624001 57.699001

O 3.799000 29.273001 43.018002

H1 4.541000 28.681000 43.243000

H2 3.497000 29.551001 43.894001

O 47.379002 60.946999 37.421001

H1 47.278999 61.917000 37.335999

H2 47.852001 60.731998 36.601002

O 61.507999 28.621000 35.912998

H1 62.396000 28.995001 35.784000

H2 60.986000 29.164000 35.278000

O 53.426998 48.375000 38.999001

H1 52.563999 48.306000 38.551998

H2 53.886002 49.026001 38.445999

O 14.049000 15.134000 28.771999

H1 13.077000 15.123000 28.650000

H2 14.129000 15.452000 29.684000

O 57.804001 29.964001 32.192001

H1 57.717999 30.892000 31.909000

H2 58.594002 29.681999 31.691000

O 18.679001 16.479000 47.326000

H1 19.087999 15.596000 47.278999

H2 19.284000 16.922001 47.969002

O 38.520000 38.407001 10.838000

H1 38.669998 38.438000 9.880000

H2 38.061001 39.241001 11.003000

O 57.979000 24.396000 20.992001

H1 58.160999 24.288000 21.948999

H2 57.228001 25.025999 21.028000

O 47.445999 15.662000 25.846001

H1 46.723999 15.029000 25.833000

H2 47.597000 15.805000 26.796000

O 17.021999 53.868999 49.382000

H1 16.360001 53.487999 49.985001

H2 17.728001 54.126999 49.991001

O 22.629999 33.723999 66.029999

H1 22.044001 33.724998 66.795998

H2 22.330999 34.507000 65.531998

O 13.297000 50.737999 38.320999

H1 12.426000 50.728001 37.875999

H2 13.583000 51.653999 38.130001

O 29.933001 27.525000 63.469002

H1 29.052000 27.117001 63.577999

H2 29.756001 28.170000 62.757000

O 60.354000 39.612999 35.230999

H1 61.183998 39.876999 34.806000

H2 59.841000 39.271999 34.466000

O 42.048000 65.524002 40.669998

H1 42.752998 64.908997 40.952999

H2 41.666000 65.028999 39.917999

O 18.398001 41.019001 52.058998

H1 18.458000 40.043999 52.176998

H2 18.919001 41.321999 52.835999

O 3.778000 29.184000 33.532001

H1 3.758000 29.266001 32.561001

H2 3.660000 28.224001 33.653000

O 25.077999 36.417999 14.072000

H1 25.141001 36.127998 15.004000

H2 24.827000 35.595001 13.627000

O 18.014999 10.579000 40.632999

H1 17.465000 10.864000 39.887001

H2 18.348000 9.714000 40.313000

O 14.489000 56.820000 46.436001

H1 14.609000 56.584000 47.384998

H2 14.992000 57.645000 46.375999

O 32.181999 58.129002 42.694000

H1 32.681000 58.277000 41.866001

H2 32.847000 57.710999 43.255001

O 58.117001 19.226000 21.809000

H1 58.625999 18.416000 21.976999

H2 58.777000 19.921000 21.974001

O 59.013000 41.714001 37.595001

H1 59.374001 40.919998 38.018002

H2 58.064999 41.633999 37.812000

O 16.297001 50.667000 53.006001

H1 16.436001 50.599998 53.962002

H2 17.187000 50.953999 52.700001

O 16.712999 16.080999 28.061001

H1 16.936001 16.580999 28.865000

H2 15.752000 15.941000 28.191000

O 67.317001 32.201000 23.740999

H1 66.681999 31.459999 23.589001

H2 66.707001 32.884998 24.066999

O 42.005001 33.602001 12.564000

H1 42.112999 32.910000 13.244000

H2 41.814999 34.383999 13.118000

O 12.966000 38.959999 55.313000

H1 12.405000 39.067001 54.528000

H2 13.485000 39.786999 55.292999

O 20.947001 33.174999 61.346001

H1 21.127001 33.039001 62.299999

H2 20.591000 34.074001 61.340000

O 41.118999 4.500000 28.084999

H1 40.349998 5.079000 28.239000

H2 40.757999 3.623000 28.302999

O 12.184000 49.229000 58.183998

H1 12.456000 48.951000 59.070000

H2 12.874000 49.870998 57.949001

O 14.975000 51.803001 35.423000

H1 14.970000 52.758999 35.270000

H2 15.777000 51.692001 35.966000

O 16.681000 44.299000 45.169998

H1 16.775000 44.404999 46.130001

H2 15.709000 44.401001 45.068001

O 3.295000 39.966000 32.053001

H1 3.366000 40.432999 31.202999

H2 4.218000 40.018002 32.379002

O 23.167000 18.066000 54.534000

H1 23.003000 18.205999 53.591000

H2 22.764999 18.870001 54.928001

O 14.247000 8.342000 38.243999

H1 14.296000 7.445000 37.862000

H2 13.512000 8.726000 37.727001

O 31.452000 8.348000 52.042000

H1 31.438000 8.482000 51.071999

H2 31.902000 9.168000 52.331001

O 17.993999 21.173000 23.841999

H1 18.610001 21.357000 23.106001

H2 17.205999 20.867001 23.362000

O 45.217999 23.864000 24.478001

H1 44.258999 24.044001 24.601000

H2 45.449001 24.561001 23.832001

O 34.863998 53.758999 25.983999

H1 35.227001 52.883999 26.171000

H2 35.667000 54.249001 25.704000

O 38.632000 16.504000 9.938000

H1 38.096001 16.094999 9.232000

H2 38.301998 16.049000 10.729000

O 30.745001 16.340000 46.720001

H1 29.872000 16.715000 46.926998

H2 31.327000 16.912001 47.264999

O 14.282000 53.685001 53.650002

H1 13.621000 54.389000 53.799999

H2 14.332000 53.661999 52.681999

O 45.660999 58.594002 46.959000

H1 46.537998 58.699001 47.373001

H2 45.636002 57.618999 46.832001

O 44.825001 42.439999 58.720001

H1 45.625999 42.493999 59.285999

H2 44.691002 43.390999 58.514999

O 30.992001 25.299000 57.265999

H1 31.459000 24.448000 57.349998

H2 31.705999 25.938000 57.327000

O 45.223000 61.280998 23.632999

H1 44.275002 61.201000 23.423000

H2 45.216000 61.925999 24.353001

O 33.380001 5.303000 31.098000

H1 33.262001 4.340000 31.117001

H2 34.353001 5.366000 31.254999

O 42.729000 44.126999 21.143999

H1 42.057999 43.446999 21.378000

H2 42.127998 44.875000 20.907000

O 31.618000 37.091999 67.815002

H1 31.926001 38.013000 67.938004

H2 32.278000 36.591999 68.304001

O 34.826000 46.389999 67.703003

H1 33.973999 46.806000 67.504997

H2 34.983002 46.662998 68.616997

O 40.403000 3.224000 36.555000

H1 39.866001 3.767000 37.158001

H2 39.723000 2.763000 36.039001

O 23.290001 64.253998 26.895000

H1 23.672001 64.130997 27.785000

H2 23.931000 63.768002 26.341000

O 49.327999 42.585999 56.512001

H1 48.908001 41.832001 56.955002

H2 50.220001 42.578999 56.869999

O 29.159000 10.082000 20.433001

H1 28.427999 10.399000 21.006001

H2 29.933001 10.407000 20.917000

O 27.393000 44.265999 68.137001

H1 28.017000 43.854000 68.764999

H2 27.115000 45.056000 68.638000

O 44.625000 49.738998 14.304000

H1 45.395000 49.946999 14.860000

H2 45.050999 49.591999 13.433000

O 9.208000 18.266001 41.171001

H1 8.356000 18.636000 40.866001

H2 9.609000 19.032000 41.609001

O 26.997999 17.792999 15.499000

H1 27.642000 17.287001 14.969000

H2 27.413000 17.761999 16.379999

O 14.477000 36.140999 33.521000

H1 14.131000 35.396999 34.035000

H2 13.702000 36.443001 33.033001

O 18.634001 51.027000 20.000000

H1 19.033001 50.374001 19.400999

H2 18.576000 50.516998 20.830999

O 23.270000 26.965000 15.201000

H1 24.218000 26.781000 15.142000

H2 23.230000 27.924000 15.101000

O 52.749001 40.216999 10.846000

H1 52.470001 39.353001 10.517000

H2 52.575001 40.146999 11.797000

O 34.715000 66.607002 31.913000

H1 34.235001 66.164001 31.204000

H2 35.588001 66.761002 31.523001

O 37.923000 31.157000 21.966999

H1 37.471001 30.429001 21.479000

H2 38.542000 30.622999 22.513000

O 58.881001 27.743999 41.902000

H1 59.445999 28.063999 41.175999

H2 58.487999 28.566000 42.230000

O 36.303001 41.723999 17.208000

H1 35.810001 42.026001 18.007000

H2 35.863998 42.259998 16.524000

O 57.421001 27.028999 51.598000

H1 56.467999 27.042000 51.417000

H2 57.799000 27.058001 50.707001

O 28.590000 10.716000 51.384998

H1 28.136000 11.385000 51.931000

H2 29.424000 11.161000 51.175999

O 9.254000 41.002998 58.256001

H1 9.054000 41.160999 57.304001

H2 9.075000 40.048000 58.317001

O 19.849001 49.694000 22.363001

H1 20.322001 50.125000 23.104000

H2 20.544001 49.605999 21.702000

O 33.955002 4.210000 47.101002

H1 33.066002 3.980000 47.412998

H2 34.513000 3.878000 47.841000

O 34.318001 64.793999 24.684999

H1 34.581001 63.911999 25.007999

H2 33.716000 64.566002 23.958000

O 22.666000 4.022000 43.907001

H1 23.014000 4.593000 44.610001

H2 22.157000 4.659000 43.369999

O 5.825000 47.431000 23.570999

H1 6.793000 47.305000 23.499001

H2 5.663000 48.118999 22.913000

O 46.181999 10.451000 31.539000

H1 46.957001 10.932000 31.195000

H2 45.578999 10.463000 30.788000

O 33.837002 42.986000 15.706000

H1 33.201000 43.598999 16.122999

H2 34.668999 43.492001 15.785000

O 56.478001 28.879999 43.757999

H1 56.438000 29.046000 42.805000

H2 55.813999 28.191999 43.889000

O 51.405998 52.987000 42.372002

H1 50.659000 52.910000 41.755001

H2 51.872002 52.139000 42.215000

O 63.285999 25.775000 25.653000

H1 63.407001 26.497999 26.290001

H2 63.592999 25.006001 26.150999

O 42.289001 44.881001 24.070000

H1 41.577999 45.066002 23.431999

H2 42.691002 44.084999 23.677000

O 8.892000 48.148998 34.900002

H1 8.748000 49.080002 34.617001

H2 9.180000 48.297001 35.827999

O 34.590000 57.764999 34.806999

H1 34.476002 58.134998 33.917000

H2 33.665001 57.539001 35.035999

O 49.799999 25.351000 26.452000

H1 49.624001 24.837999 27.261999

H2 49.154999 24.969999 25.836000

O 24.948999 8.659000 51.604000

H1 25.577999 8.818000 50.875999

H2 25.552999 8.294000 52.285000

O 27.099001 49.334000 56.575001

H1 27.056000 48.585999 57.202999

H2 26.468000 49.955002 56.977001

O 8.682000 26.044001 44.729000

H1 8.219000 25.761000 43.915001

H2 8.606000 27.007999 44.665001

O 49.688000 62.221001 23.853001

H1 49.959999 61.500000 24.450001

H2 50.530998 62.474998 23.451000

O 16.275999 51.080002 61.851002

H1 16.403999 50.914001 60.891998

H2 15.466000 50.583000 62.026001

O 41.859001 9.516000 45.723999

H1 41.612000 10.055000 46.488998

H2 41.889000 10.208000 45.015999

O 27.232000 45.900002 11.572000

H1 27.253000 44.978001 11.863000

H2 26.287001 45.988998 11.301000

O 32.347000 33.327000 64.773003

H1 33.158001 32.785999 64.660004

H2 31.666000 32.629002 64.763000

O 58.840000 38.855999 33.278000

H1 58.490002 38.112000 32.751999

H2 58.106998 38.995998 33.914001

O 55.305000 54.063999 29.676001

H1 55.938000 54.665001 30.112000

H2 54.710999 53.844002 30.414000

O 63.625999 26.165001 38.067001

H1 62.896999 25.523001 38.053001

H2 64.387001 25.601999 37.842999

O 27.035000 41.296001 70.463997

H1 27.034000 40.737000 69.669998

H2 26.792000 40.641998 71.154999

O 61.344002 46.518002 28.808001

H1 62.077000 45.907001 28.639999

H2 61.665001 47.018002 29.576000

O 28.386000 17.142000 18.179001

H1 28.419001 16.216999 18.516001

H2 29.212999 17.493999 18.573000

O 4.284000 29.749001 30.882999

H1 5.094000 30.268999 30.711000

H2 4.675000 28.849001 30.975000

O 59.763000 25.275999 28.094000

H1 60.404999 26.004000 28.055000

H2 60.076000 24.698999 27.367001

O 13.447000 22.804001 21.577000

H1 13.032000 22.290001 22.284000

H2 13.270000 23.718000 21.856001

O 16.368999 20.400000 46.062000

H1 15.404000 20.540001 46.195999

H2 16.665001 21.278000 45.805000

O 28.634001 35.882999 54.132000

H1 28.691000 36.556999 53.433998

H2 28.868000 36.391998 54.926998

O 52.185001 65.810997 18.396999

H1 52.154999 66.102997 17.476000

H2 52.098000 64.844002 18.306000

O 24.851000 59.540001 42.752998

H1 25.709000 59.299000 42.372002

H2 24.846001 60.507000 42.625000

O 32.154999 7.131000 17.969999

H1 32.416000 6.200000 18.125000

H2 32.910000 7.438000 17.423000

O 53.751999 37.950001 58.575001

H1 53.492001 38.494999 57.800999

H2 53.220001 37.150002 58.432999

O 17.575001 33.418999 30.017000

H1 18.035999 32.757000 30.551001

H2 16.653000 33.286999 30.266001

O 58.789001 32.618999 19.900999

H1 58.902000 31.716999 20.256001

H2 58.806000 32.449001 18.941999

O 6.699000 45.757999 42.273998

H1 6.853000 46.709999 42.403999

H2 5.879000 45.752998 41.754002

O 15.821000 40.740002 64.836998

H1 16.563000 41.229000 64.441002

H2 15.269000 40.549999 64.064003

O 46.830002 40.340000 66.695000

H1 47.664001 39.839001 66.749001

H2 46.504002 40.069000 65.819000

O 53.459000 13.078000 29.566000

H1 52.883999 12.470000 29.073000

H2 52.826000 13.575000 30.103001

O 10.509000 28.836000 33.519001

H1 10.890000 28.625999 34.393002

H2 9.761000 28.224001 33.473000

O 53.554001 36.287998 44.106998

H1 53.626999 37.251999 43.956001

H2 53.368000 35.957001 43.221001

O 56.578999 30.712000 48.168999

H1 56.332001 31.158001 47.342999

H2 57.411999 31.153999 48.394001

O 16.139999 35.854000 60.719002

H1 16.983000 35.530998 61.081001

H2 16.167000 36.793999 60.976002

O 35.407001 3.893000 23.438999

H1 36.200001 4.032000 22.896999

H2 35.374001 2.917000 23.493000

O 57.858002 30.916000 50.758999

H1 57.265999 30.414000 50.176998

H2 58.693001 30.891001 50.254002

O 14.157000 51.431999 58.805000

H1 15.053000 51.792999 58.750000

H2 13.606000 52.202999 58.591999

O 58.873001 42.433998 19.759001

H1 58.708000 43.355000 19.530001

H2 58.099998 42.209000 20.301001

O 13.089000 34.807999 65.555000

H1 13.787000 34.428001 65.004997

H2 12.912000 35.653999 65.117996

O 30.978001 10.064000 17.627001

H1 31.816000 9.665000 17.895000

H2 30.952999 9.858000 16.672001

O 46.810001 32.365002 21.754999

H1 47.756001 32.563999 21.934999

H2 46.884998 31.893999 20.900000

O 31.069000 46.563000 56.423000

H1 31.497000 46.569000 57.299000

H2 31.549999 45.847000 55.973999

O 12.357000 25.864000 26.972000

H1 12.472000 25.754000 27.927999

H2 12.769000 26.743000 26.830999

O 10.690000 48.096001 23.620001

H1 11.265000 47.811001 22.893000

H2 10.036000 47.375999 23.650999

O 23.386000 28.617001 54.646999

H1 22.961000 29.482000 54.591999

H2 23.653999 28.469000 53.716000

O 14.246000 41.610001 54.872002

H1 14.412000 42.122002 54.053001

H2 15.149000 41.310001 55.083000

O 59.159000 31.882000 39.469002

H1 59.810001 31.313999 39.037998

H2 59.333000 31.716000 40.411999

O 31.333000 42.577999 66.396004

H1 31.375999 42.588001 65.420998

H2 30.877001 41.728001 66.555000

O 14.318000 50.962002 32.712002

H1 14.765000 51.666000 32.202999

H2 14.554000 51.207001 33.624001

O 26.087999 47.078999 54.978001

H1 26.464001 46.301998 55.431000

H2 26.480000 47.806000 55.497002

O 38.549999 46.286999 18.802999

H1 38.455002 45.396999 19.204000

H2 38.618999 46.834000 19.618000

O 58.931000 44.644001 33.917000

H1 58.300999 43.905998 33.948002

H2 58.409000 45.348999 33.511002

O 55.194000 48.344002 16.252001

H1 54.476002 48.063000 16.839001

H2 55.247002 49.296001 16.436001

O 55.757999 40.251999 19.952999

H1 55.006001 40.817001 20.219999

H2 56.092999 39.938999 20.798000

O 31.540001 54.985001 58.151001

H1 32.487999 54.787998 58.047001

H2 31.559000 55.946999 58.306000

O 2.388000 44.327000 32.145000

H1 2.308000 43.370998 32.334999

H2 2.215000 44.710999 33.018002

O 43.978001 46.636002 14.839000

H1 44.104000 47.583000 14.674000

H2 43.804001 46.610001 15.789000

O 48.416000 19.125000 57.292000

H1 49.078999 19.486000 56.671001

H2 47.702000 19.795000 57.207001

O 43.931999 58.776001 20.667999

H1 43.596001 59.625000 20.325001

H2 43.598999 58.153999 19.990999

O 21.850000 41.123001 14.917000

H1 22.591000 41.199001 14.310000

H2 21.437000 42.007000 14.867000

O 34.671001 32.430000 17.834000

H1 35.414001 32.403000 17.201000

H2 34.338001 33.335999 17.697001

O 53.216000 39.720001 56.641998

H1 54.104000 40.125000 56.720001

H2 53.007999 39.895000 55.710999

O 44.727001 45.068001 58.299999

H1 44.875000 45.884998 57.785000

H2 44.362999 45.423000 59.125999

O 27.510000 16.971001 61.558998

H1 26.673000 16.565001 61.285999

H2 27.847000 16.327999 62.203999

O 18.048000 18.927999 39.723000

H1 18.587000 19.693001 39.425999

H2 17.870001 19.181999 40.652000

O 21.509001 60.438000 28.465000

H1 20.606001 60.124001 28.662001

H2 21.987000 60.165001 29.271000

O 59.831001 20.632000 39.692001

H1 58.980999 20.679001 39.213001

H2 60.460999 20.638000 38.949001

O 34.417999 8.845000 27.052999

H1 35.141998 8.543000 26.483999

H2 33.674000 8.297000 26.739000

O 21.445000 10.531000 52.375999

H1 20.688999 10.907000 52.845001

H2 21.943001 10.100000 53.089001

O 58.794998 39.493000 29.236000

H1 59.036999 39.948002 30.066000

H2 59.577000 38.929001 29.087000

O 22.830000 32.463001 22.957001

H1 22.844000 31.490999 23.084999

H2 22.708000 32.498001 21.978001

O 23.357000 38.028000 15.773000

H1 23.722000 38.048000 14.876000

H2 23.823000 37.255001 16.153000

O 20.075001 22.011999 51.985001

H1 19.386000 22.691000 52.110001

H2 20.822001 22.396000 52.460999

O 18.462999 7.996000 31.966999

H1 19.333000 8.360000 32.226002

H2 17.992001 8.812000 31.716000

O 19.945000 41.521999 19.084000

H1 20.153000 42.400002 18.695000

H2 19.316000 41.819000 19.792999

O 36.882999 22.061001 64.364998

H1 36.316002 22.683001 64.863998

H2 37.171001 21.455000 65.057999

O 27.597000 43.269001 7.930000

H1 26.652000 43.237999 8.167000

H2 27.605000 43.897999 7.200000

O 9.433000 45.507000 41.876999

H1 8.756000 45.798000 42.512001

H2 9.012000 45.710999 41.028000

O 45.397999 32.716999 62.549999

H1 45.834000 33.500999 62.921001

H2 45.356998 32.133999 63.342999

O 42.480000 17.811001 15.198000

H1 42.817001 18.579000 14.682000

H2 41.674999 17.596001 14.701000

O 25.554001 48.304001 14.961000

H1 26.444000 48.039001 14.666000

H2 25.601000 48.113998 15.916000

O 56.734001 46.868999 41.166000

H1 57.282001 47.673000 41.258999

H2 57.159000 46.448002 40.389000

O 33.501999 42.102001 68.365997

H1 34.435001 41.839001 68.246002

H2 33.442001 42.872002 67.778999

O 21.980000 63.448002 23.431999

H1 21.080999 63.657001 23.721001

H2 22.323999 62.924999 24.167000

O 52.094002 10.640000 40.625999

H1 52.823002 10.858000 40.009998

H2 51.797001 9.780000 40.278999

O 16.195000 33.278999 45.145000

H1 16.063999 34.222000 44.898998

H2 15.794000 32.841000 44.370998

O 2.323000 23.903000 37.056000

H1 2.189000 23.056000 36.599998

H2 3.247000 23.840000 37.348999

O 17.941999 4.795000 31.719999

H1 17.500999 3.954000 31.909000

H2 18.735001 4.734000 32.290001

O 29.444000 24.471001 59.523998

H1 28.528000 24.153000 59.421001

H2 29.745001 24.502001 58.599998

O 30.059999 52.591000 51.255001

H1 30.077999 51.813000 50.688000

H2 30.195000 52.202000 52.143002

O 43.417000 15.072000 24.726000

H1 42.542999 15.096000 24.312000

H2 43.919998 15.682000 24.141001

O 47.165001 18.688999 51.761002

H1 47.459000 18.490000 52.676998

H2 46.907001 17.809999 51.450001

O 5.692000 28.009001 44.625000

H1 6.041000 27.650000 45.472000

H2 5.307000 27.202999 44.237999

O 46.180000 49.537998 30.408001

H1 46.409000 48.576000 30.485001

H2 46.467999 49.680000 29.474001

O 31.677000 50.851002 15.439000

H1 32.176998 51.631001 15.149000

H2 31.270000 51.154999 16.260000

O 42.375999 17.341000 42.772999

H1 42.143002 17.289000 43.720001

H2 41.528999 17.627001 42.386002

O 32.625000 36.164001 61.775002

H1 32.655998 36.911999 61.136002

H2 33.359001 35.616001 61.467999

O 13.940000 23.921000 37.741001

H1 14.201000 24.250999 38.620998

H2 13.646000 23.017000 37.956001

O 54.956001 58.022999 30.600000

H1 54.330002 57.707001 29.930000

H2 55.660999 57.359001 30.551001

O 20.799999 54.958000 43.361000

H1 21.194000 55.560001 44.022999

H2 21.483999 54.275002 43.278000

O 16.961000 35.243999 64.383003

H1 16.496000 36.089001 64.540001

H2 17.768999 35.353001 64.903000

O 25.429001 6.850000 30.540001

H1 25.658001 7.357000 31.340000

H2 24.455999 6.876000 30.568001

O 53.255001 16.035000 47.529999

H1 53.266998 16.900999 47.082001

H2 52.311001 15.820000 47.528000

O 36.953999 25.121000 22.285999

H1 36.141998 24.684999 22.591000

H2 36.863998 26.013000 22.646000

O 17.850000 16.395000 34.660999

H1 18.580000 16.382000 34.026001

H2 17.836000 17.311001 34.965000

O 6.090000 42.769001 42.449001

H1 6.193000 43.588001 42.957001

H2 5.141000 42.574001 42.577000

O 13.961000 2.995000 32.231998

H1 14.791000 2.830000 31.752001

H2 13.327000 2.473000 31.715000

O 37.084000 15.278000 12.344000

H1 37.921001 15.495000 12.779000

H2 36.432999 15.517000 13.031000

O 23.136999 57.104000 59.014000

H1 23.299999 56.904999 59.959000

H2 22.327000 57.625000 59.051998

O 39.792000 10.717000 31.466000

H1 39.070999 10.836000 30.801001

H2 39.269001 10.502000 32.258999

O 33.639999 62.507999 42.285999

H1 33.748001 61.640999 42.717999

H2 34.462002 62.958000 42.561001

O 53.450001 18.598000 46.472000

H1 53.326000 19.103001 47.292000

H2 53.050999 19.200001 45.811001

O 59.244999 16.392000 30.219000

H1 59.248001 15.469000 29.899000

H2 59.581001 16.270000 31.125000

O 26.926001 58.400002 58.640999

H1 26.521999 57.869999 57.938999

H2 26.163000 58.950001 58.929001

O 58.320000 49.327999 33.030998

H1 58.880001 49.730000 33.712002

H2 57.549999 49.036999 33.548000

O 54.993999 30.450001 39.646999

H1 54.745998 29.528999 39.433998

H2 54.143002 30.900000 39.653999

O 36.681000 61.851002 17.399000

H1 36.806999 62.685001 17.888000

H2 37.583000 61.486000 17.389999

O 23.913000 13.942000 50.978001

H1 23.072001 14.417000 50.849998

H2 24.566000 14.640000 50.854000

O 54.733002 10.788000 32.691002

H1 55.292999 10.163000 33.196999

H2 53.841000 10.509000 33.007999

O 30.500999 0.090000 34.797001

H1 31.045000 0.654000 34.231998

H2 29.610001 0.462000 34.654999

O 48.055000 4.979000 31.436001

H1 48.414001 5.885000 31.441000

H2 47.161999 5.121000 31.093000

O 15.356000 43.512001 53.183998

H1 15.035000 44.424000 53.213001

H2 16.108000 43.542999 53.804001

O 2.682000 34.556000 33.622002

H1 2.925000 35.396999 33.216999

H2 3.463000 34.002998 33.450001

O 55.075001 35.812000 39.007999

H1 55.743999 35.118999 38.893002

H2 54.830002 36.011002 38.085999

O 13.424000 43.183998 19.146000

H1 12.650000 43.147999 19.740000

H2 13.707000 42.250000 19.146000

O 21.412001 53.112999 31.448000

H1 20.893999 53.257000 32.269001

H2 22.267000 53.521000 31.708000

O 42.487000 37.359001 12.565000

H1 42.076000 36.980000 11.763000

H2 41.689999 37.556000 13.101000

O 57.083000 15.099000 38.020000

H1 56.313999 15.069000 38.626999

H2 56.938000 15.941000 37.567001

O 50.473000 19.657000 39.221001

H1 50.110001 20.419001 39.719002

H2 50.159000 18.914000 39.763000

O 33.738998 52.459999 19.732000

H1 33.169998 51.922001 20.304001

H2 34.209000 51.780998 19.218000

O 55.881001 43.814999 24.997999

H1 56.637001 43.285000 25.347000

H2 55.437000 44.062000 25.822001

O 55.764000 8.660000 34.012001

H1 56.641998 8.290000 34.210999

H2 55.243999 8.276000 34.768002

O 43.305000 46.270000 46.754002

H1 43.459000 47.131001 46.318001

H2 42.948002 45.742001 46.022999

O 48.407001 16.080999 45.881001

H1 47.914001 15.299000 46.213001

H2 49.198002 16.042000 46.453999

O 59.965000 29.617001 34.047001

H1 60.187000 30.313999 33.400002

H2 59.167000 29.233000 33.657001

O 49.928001 26.108999 62.971001

H1 50.890999 26.086000 62.792000

H2 49.883999 26.796000 63.657001

O 49.341999 38.091000 8.877000

H1 49.481998 38.592999 9.692000

H2 49.085999 38.799999 8.253000

O 20.622999 41.623001 65.421997

H1 21.243000 42.352001 65.561996

H2 21.079000 40.875000 65.834000

O 37.935001 3.787000 26.356001

H1 38.272999 2.913000 26.105000

H2 37.180000 3.556000 26.917999

O 19.135000 58.285999 46.500999

H1 18.625999 58.722000 47.202000

H2 19.704000 59.012001 46.181000

O 60.676998 38.716999 19.650000

H1 60.306000 38.077000 19.000999

H2 60.854000 38.139000 20.406000

O 36.761002 41.448002 52.299000

H1 36.279999 40.618999 52.521999

H2 36.692001 41.941002 53.126999

O 55.205002 31.825001 43.321999

H1 54.300999 31.987000 42.992001

H2 55.271000 30.860001 43.254002

O 23.111000 10.920000 24.357000

H1 22.851000 11.314000 25.219999

H2 23.737000 10.239000 24.642000

O 44.527000 66.194000 18.209000

H1 44.160000 67.085999 18.163000

H2 45.372002 66.331001 18.652000

O 22.312000 10.563000 19.892000

H1 22.815001 9.916000 19.372000

H2 22.058001 10.038000 20.669001

O 28.548000 1.126000 43.147999

H1 29.205999 1.295000 42.460999

H2 27.740000 1.493000 42.768002

O 63.334000 48.012001 30.792000

H1 63.254002 47.087002 31.096001

H2 62.602001 48.441002 31.260000

O 37.620998 6.067000 34.862999

H1 37.125999 5.274000 35.123001

H2 37.073002 6.438000 34.162998

O 32.087002 17.659000 58.591000

H1 31.240999 17.721001 59.064999

H2 32.683998 18.141001 59.195999

O 15.281000 38.493000 44.054001

H1 14.509000 38.813000 44.575001

H2 16.007999 38.946999 44.546001

O 20.049999 32.127998 58.742001

H1 19.830999 31.186001 58.605999

H2 20.233000 32.159000 59.692001

O 13.390000 54.048000 34.233002

H1 12.873000 54.384998 33.469002

H2 12.873000 53.264999 34.476002

O 11.256000 47.071999 26.544001

H1 11.373000 46.373001 27.208000

H2 11.242000 47.875000 27.091000

O 43.521000 50.229000 19.757000

H1 42.800999 50.734001 19.315001

H2 43.970001 50.945000 20.245001

O 27.948999 59.080002 25.568001

H1 27.125000 58.542999 25.590000

H2 27.968000 59.375000 24.650999

O 12.027000 13.019000 32.916000

H1 11.333000 12.790000 32.277000

H2 11.860000 13.971000 33.044998

O 36.431000 54.013000 47.618999

H1 35.618999 54.358002 48.002998

H2 36.494999 54.528000 46.784000

O 40.702000 33.005001 16.774000

H1 40.930000 32.658001 17.650999

H2 40.612999 33.955002 16.948999

O 29.716000 58.855000 41.808998

H1 29.084999 58.118999 41.722000

H2 30.462000 58.431000 42.268002

O 59.646999 34.388000 42.360001

H1 59.630001 33.422001 42.456001

H2 58.706001 34.604000 42.299000

O 57.993999 23.767000 36.852001

H1 57.813000 24.583000 36.359001

H2 57.196999 23.240999 36.679001

O 43.584000 44.735001 53.103001

H1 43.248001 43.914001 53.508999

H2 43.013000 44.805000 52.318001

O 54.756001 51.353001 38.908001

H1 54.320999 51.722000 38.130001

H2 55.695000 51.499001 38.715000

O 58.044998 37.028999 40.034000

H1 57.706001 36.126999 40.070000

H2 57.450001 37.455002 39.398998

O 31.743999 57.554001 20.879000

H1 32.278999 58.167000 21.402000

H2 31.709000 57.995998 20.013000

O 53.063999 46.853001 8.829000

H1 52.678001 47.410000 9.524000

H2 54.018002 46.991001 8.969000

O 43.714001 19.971001 55.286999

H1 44.188999 20.108000 54.453999

H2 43.723999 20.865999 55.674000

O 6.134000 43.557999 33.839001

H1 5.355000 43.075001 34.207001

H2 6.818000 43.326000 34.480000

O 31.030001 57.608002 18.023001

H1 31.851999 57.243999 17.663000

H2 30.414000 56.865002 17.924000

O 46.286999 54.076000 26.933001

H1 45.606998 54.763000 26.816000

H2 45.894001 53.334999 26.430000

O 47.518002 39.250999 47.744999

H1 47.633999 39.285999 46.785000

H2 48.228001 38.645000 48.014000

O 31.943001 20.127001 56.160000

H1 32.631001 19.525000 55.804001

H2 31.834000 20.740999 55.403000

O 20.490999 53.599998 58.000999

H1 21.431999 53.712002 57.838001

H2 20.393999 52.636002 58.066002

O 44.160000 39.799000 57.672001

H1 44.511002 40.692001 57.502998

H2 43.228001 40.002998 57.882000

O 50.191002 52.493999 14.291000

H1 50.157001 53.425999 14.016000

H2 50.778000 52.111000 13.621000

O 50.139000 51.083000 36.852001

H1 50.986000 51.437000 36.535999

H2 49.535999 51.834000 36.676998

O 16.250000 59.374001 35.522999

H1 16.223000 58.789001 34.748001

H2 16.205000 60.256001 35.116001

O 28.014000 62.214001 51.723000

H1 27.941999 62.285999 52.688000

H2 28.902000 62.577000 51.558998

O 29.228001 53.844002 58.841999

H1 30.063999 54.327999 58.678001

H2 29.018999 53.535000 57.936001

O 49.783001 18.732000 44.763000

H1 50.007000 19.083000 45.632999

H2 49.443001 17.851999 44.959999

O 14.611000 34.249001 23.222000

H1 13.781000 34.660999 22.940001

H2 14.447000 33.310001 23.014000

O 37.393002 47.810001 49.743000

H1 36.595001 47.275002 49.528000

H2 36.966999 48.577999 50.181999

O 40.720001 66.138000 28.908001

H1 40.966999 65.597000 29.673000

H2 39.784000 65.884003 28.783001

O 12.118000 46.416000 46.895000

H1 11.453000 45.945000 47.426998

H2 12.246000 47.228001 47.409000

O 37.294998 39.450001 66.227997

H1 36.855000 38.630001 66.480003

H2 36.879002 39.674999 65.388000

O 30.766001 66.181000 50.141998

H1 29.948999 65.662003 50.152000

H2 31.045000 66.066002 49.200001

O 56.668999 22.407000 23.413000

H1 55.824001 22.614000 23.844999

H2 57.237999 23.129999 23.723000

O 18.886999 52.146000 28.958000

H1 19.671000 52.716000 29.086000

H2 18.577000 52.458000 28.087000

O 55.832001 29.910999 19.818001

H1 55.502998 30.754000 19.447001

H2 55.219002 29.278999 19.389000

O 52.011002 44.168999 7.510000

H1 51.984001 44.779999 8.264000

H2 52.592999 44.653000 6.890000

O 11.875000 33.811001 21.934000

H1 11.137000 33.689999 21.299000

H2 12.037000 34.771999 21.844999

O 9.503000 35.341000 60.950001

H1 9.237000 34.721001 61.654999

H2 9.857000 34.742001 60.279999

O 33.706001 62.214001 39.632999

H1 34.071999 63.048000 39.318001

H2 33.667999 62.359001 40.602001

O 47.588001 40.839001 42.321999

H1 47.285999 40.695000 43.251999

H2 48.264000 41.539001 42.493000

O 50.870998 49.042999 15.901000

H1 51.097000 49.469002 15.051000

H2 50.800999 48.110001 15.631000

O 15.320000 43.268002 25.636999

H1 15.782000 43.506001 26.462000

H2 14.396000 43.509998 25.870001

O 36.040001 12.763000 31.858999

H1 36.883999 13.152000 31.575001

H2 36.219002 11.814000 31.802000

O 16.341000 46.613998 54.081001

H1 15.573000 47.194000 53.971001

H2 16.354000 46.143002 53.223000

O 18.933001 46.841999 55.562000

H1 19.437000 47.669998 55.526001

H2 18.087999 47.098999 55.159000

O 49.360001 47.501999 20.860001

H1 49.407001 48.334999 21.384001

H2 48.423000 47.261002 21.017000

O 16.077999 21.608999 21.297001

H1 15.190000 22.011000 21.297001

H2 15.850000 20.656000 21.313999

O 14.432000 12.276000 25.056000

H1 14.045000 12.037000 25.915001

H2 15.358000 12.035000 25.167000

O 42.223000 50.797001 47.299999

H1 41.382999 51.103001 46.930000

H2 41.932999 50.126999 47.938999

O 14.758000 38.429001 58.995998

H1 14.750000 39.070000 58.264999

H2 15.480000 38.791000 59.554001

O 26.063999 4.318000 50.064999

H1 25.548000 3.629000 50.507999

H2 25.961000 5.071000 50.664001

O 62.226002 45.992001 21.400000

H1 62.792999 46.777000 21.419001

H2 61.722000 46.075001 22.225000

O 52.334000 21.959000 25.892000

H1 52.893002 21.259001 25.486000

H2 52.817001 22.145000 26.708000

O 50.348999 63.312000 32.035000

H1 49.509998 63.249001 32.535000

H2 50.056999 63.747002 31.219000

O 6.472000 49.054001 27.222000

H1 6.522000 48.943001 28.174999

H2 7.054000 48.345001 26.889000

O 17.187000 6.116000 26.188000

H1 16.719000 5.530000 25.582001

H2 16.886999 6.998000 25.879999

O 21.462000 44.426998 65.985001

H1 20.877001 44.500999 66.772003

H2 22.254999 44.889999 66.310997

O 44.631001 56.499001 15.731000

H1 44.955002 55.667000 16.110001

H2 45.459999 56.882999 15.381000

O 36.235001 14.938000 42.882000

H1 37.082001 15.401000 43.001999

H2 35.741001 15.219000 43.668999

O 56.237000 26.569000 20.940001

H1 55.313999 26.822001 21.157000

H2 56.181000 26.472000 19.973000

O 66.453003 31.004000 41.659000

H1 66.011002 31.457001 40.929001

H2 67.011002 30.355000 41.181999

O 34.012001 40.034000 11.335000

H1 33.979000 40.667999 12.083000

H2 33.103001 40.116001 10.991000

O 10.946000 24.827000 38.376999

H1 11.032000 25.620001 37.807999

H2 11.852000 24.712999 38.695000

O 20.921000 50.193001 62.201000

H1 20.476999 49.379002 62.523998

H2 21.856001 49.966999 62.355000

O 37.752998 47.740002 63.573002

H1 37.035999 47.807999 64.231003

H2 38.541000 47.865002 64.123001

O 23.212000 46.070999 18.385000

H1 23.666000 45.341000 18.840000

H2 22.514000 46.305000 19.025000

O 37.348000 48.980000 53.224998

H1 36.919998 49.700001 53.722000

H2 37.104000 49.221001 52.307999

O 30.680000 44.834000 61.838001

H1 30.187000 44.057999 62.152000

H2 30.358999 44.889000 60.909000

O 61.973999 26.062000 43.335999

H1 62.077999 26.885000 42.841000

H2 62.889000 25.777000 43.467999

O 48.853001 24.658001 17.983000

H1 49.744999 24.856001 17.684999

H2 48.999001 24.344000 18.893000

O 40.463001 42.785000 56.455002

H1 40.046001 43.341999 57.131001

H2 41.167999 43.347000 56.119999

O 58.020000 48.410999 16.966000

H1 58.360001 47.556999 17.268999

H2 57.112000 48.188000 16.697001

O 67.630997 37.152000 23.941999

H1 66.914001 37.430000 23.319000

H2 67.120003 36.898998 24.719000

O 35.321999 12.288000 58.688000

H1 35.089001 11.335000 58.619999

H2 36.167000 12.300000 58.202000

O 52.233002 32.028000 51.063999

H1 51.844002 32.840000 51.417000

H2 53.181000 32.155998 51.233002

O 56.028999 27.737000 56.036999

H1 56.986000 27.834999 55.945999

H2 55.702999 28.629999 55.854000

O 8.202000 32.242001 57.765999

H1 7.609000 31.480000 57.845001

H2 8.785000 32.133999 58.534000

O 26.388000 57.895000 38.893002

H1 26.551001 57.801998 37.933998

H2 25.721001 57.209999 39.047001

O 52.881001 29.763000 64.473999

H1 53.813999 29.808001 64.780998

H2 52.466000 29.304001 65.235001

O 46.972000 23.075001 59.853001

H1 47.618999 23.172001 60.597000

H2 47.173000 23.889000 59.347000

O 54.228001 54.823002 19.669001

H1 55.178001 54.994999 19.788000

H2 53.840000 55.688999 19.875000

O 25.294001 45.395000 14.523000

H1 25.684000 44.544998 14.266000

H2 25.839001 45.648998 15.282000

O 51.812000 10.984000 28.527000

H1 52.415001 10.347000 28.937000

H2 50.955002 10.524000 28.598000

O 18.886000 44.245998 50.053001

H1 19.250999 44.203999 50.955002

H2 19.330999 43.480000 49.637001

O 30.527000 28.823000 9.677000

H1 30.500000 28.693001 10.637000

H2 30.208000 29.728001 9.579000

O 29.987000 12.553000 17.983999

H1 29.594000 12.393000 18.855000

H2 30.385000 11.679000 17.789000

O 10.394000 41.973000 51.952999

H1 11.331000 41.979000 51.714001

H2 10.336000 42.674000 52.612999

O 53.566002 44.188000 11.064000

H1 54.151001 43.530998 10.657000

H2 54.193001 44.769001 11.523000

O 39.458000 30.299999 64.434998

H1 38.596001 30.121000 64.856003

H2 39.210999 30.325001 63.495998

O 18.823000 23.840000 46.457001

H1 19.632000 24.370001 46.341999

H2 18.125000 24.466000 46.238998

O 55.537998 57.985001 21.077999

H1 55.269001 57.626999 21.938999

H2 54.698002 57.987999 20.589001

O 34.091000 2.084000 41.610001

H1 33.702000 2.984000 41.681000

H2 33.525002 1.593000 42.229000

O 60.358002 26.593000 20.848000

H1 59.598999 26.643000 21.461000

H2 59.908001 26.634001 19.987000

O 48.984001 36.959000 60.375999

H1 48.255001 36.665001 59.789001

H2 48.987000 37.922001 60.188000

O 41.368000 43.555000 66.329002

H1 40.636002 42.929001 66.386002

H2 41.027000 44.223999 65.714996

O 16.510000 52.084000 17.077999

H1 16.837999 51.243000 16.722000

H2 16.601999 51.941002 18.040001

O 23.931999 5.411000 41.351002

H1 24.209000 4.923000 42.138000

H2 23.993999 4.736000 40.655998

O 16.069000 4.810000 46.078999

H1 16.422001 4.235000 46.778999

H2 16.266001 4.281000 45.280998

O 13.620000 20.073000 55.859001

H1 13.991000 20.558001 55.091999

H2 13.800000 20.709000 56.581001

O 39.738998 22.204000 20.985001

H1 39.026001 22.023001 21.620001

H2 40.134998 21.325001 20.872999

O 30.961000 63.919998 20.587000

H1 30.896999 63.334000 19.802000

H2 30.514999 63.365002 21.261000

O 20.481001 2.753000 52.751999

H1 21.033001 2.001000 53.000999

H2 20.878000 3.483000 53.256001

O 35.937000 15.290000 23.469000

H1 36.762001 15.795000 23.533001

H2 35.366001 15.780000 24.104000

O 17.598000 57.617001 48.938000

H1 18.472000 57.888000 49.230999

H2 17.013000 58.284000 49.334000

O 41.694000 36.792000 63.001999

H1 41.577000 37.755001 62.911999

H2 41.285999 36.605999 63.851002

O 26.426001 18.788000 59.740002

H1 26.906000 18.219000 60.372002

H2 26.687000 18.367001 58.888000

O 40.250000 47.365002 61.782001

H1 40.790001 48.167000 61.922001

H2 39.367001 47.738998 61.650002

O 41.583000 44.463001 11.758000

H1 41.379002 44.535999 12.717000

H2 41.250000 45.337002 11.450000

O 13.833000 43.098999 48.939999

H1 13.945000 43.948002 49.401001

H2 14.399000 43.217999 48.165001

O 49.368000 20.549999 55.282001

H1 49.199001 20.715000 54.333000

H2 49.091999 21.406000 55.669998

O 33.971001 47.930000 50.680000

H1 34.084999 47.411999 51.500000

H2 34.353001 47.311001 50.019001

O 2.847000 23.749001 32.933998

H1 2.501000 23.757999 32.016998

H2 2.023000 23.917999 33.438999

O 8.885000 43.770000 23.275000

H1 9.712000 43.680000 23.773001

H2 8.840000 42.907001 22.819000

O 56.287998 29.561001 26.120001

H1 55.483002 29.086000 25.834000

H2 56.537998 30.028999 25.305000

O 48.056000 48.433998 47.169998

H1 47.386002 48.237999 47.860001

H2 48.640999 49.046001 47.648998

O 14.385000 21.940001 57.653999

H1 14.913000 22.753000 57.674000

H2 13.702000 22.112000 58.316002

O 11.741000 50.230999 24.799999

H1 11.177000 50.595001 25.506001

H2 11.183000 49.486000 24.478001

O 10.797000 15.185000 49.513000

H1 11.487000 14.530000 49.691002

H2 11.258000 16.024000 49.664001

O 24.162001 31.361000 14.497000

H1 23.962000 30.438000 14.254000

H2 24.488001 31.716999 13.655000

O 35.561001 54.495998 22.584999

H1 36.203999 54.717999 23.298000

H2 34.939999 53.931999 23.080000

O 26.136999 66.702003 28.733000

H1 26.066000 67.637001 29.006001

H2 25.264999 66.359001 28.974001

O 32.066002 9.381000 33.554001

H1 32.292000 8.422000 33.521000

H2 31.099001 9.336000 33.473000

O 13.460000 24.794001 29.836000

H1 13.078000 25.496000 30.392000

H2 14.396000 24.816000 30.113001

O 19.021999 13.844000 30.572001

H1 19.388000 14.430000 31.264000

H2 18.096001 13.758000 30.872000

O 13.940000 21.021999 32.647999

H1 13.305000 21.759001 32.514999

H2 13.940000 20.945999 33.616001

O 30.815001 2.274000 27.914000

H1 31.728001 2.081000 28.184000

H2 30.938999 2.563000 26.990000

O 24.032000 28.594000 60.002998

H1 23.066000 28.577000 60.098999

H2 24.118000 28.683001 59.027000

O 50.098000 55.189999 43.841999

H1 49.442001 54.598999 44.238998

H2 50.540001 54.595001 43.207001

O 22.336000 11.916000 26.738001

H1 21.559999 12.166000 27.280001

H2 22.729000 11.210000 27.284000

O 37.766998 30.742001 17.834000

H1 37.979000 31.145000 18.688000

H2 38.631001 30.698000 17.400000

O 61.130001 40.077999 39.367001

H1 61.780998 39.376999 39.547001

H2 60.666000 39.709999 38.587002

O 25.858999 6.958000 48.819000

H1 26.500999 6.266000 48.618999

H2 26.424999 7.649000 49.222000

O 7.538000 43.755001 39.345001

H1 8.266000 43.106998 39.448002

H2 7.172000 43.762001 40.244999

O 41.806000 40.471001 10.157000

H1 42.331001 40.930000 9.488000

H2 41.577000 39.639000 9.706000

O 17.229000 48.824001 21.052999

H1 16.396999 48.347000 21.243999

H2 17.426001 48.532001 20.150999

O 30.674000 50.469002 64.053001

H1 31.246000 49.777000 63.687000

H2 30.850000 50.391998 65.009003

O 31.787001 44.433998 16.933001

H1 31.629000 44.341999 17.893000

H2 30.872999 44.501999 16.596001

O 54.375000 19.018999 22.181000

H1 54.548000 18.351999 21.492001

H2 54.637001 19.839001 21.721001

O 56.229000 41.450001 44.301998

H1 56.091999 42.401001 44.433998

H2 55.595001 41.077000 44.945999

O 10.793000 32.212002 27.025999

H1 11.645000 31.865999 26.716000

H2 10.165000 31.600000 26.599001

O 12.157000 18.306999 48.055000

H1 12.338000 18.027000 48.967999

H2 11.271000 18.712999 48.148998

O 44.099998 46.063999 61.825001

H1 43.862999 45.608002 62.664001

H2 45.064999 46.159000 61.946999

O 39.498001 38.470001 40.679001

H1 39.740002 38.258999 41.620998

H2 40.213001 39.113998 40.483002

O 63.992001 39.630001 28.610001

H1 63.390999 39.987999 29.298000

H2 64.829002 39.581001 29.110001

O 51.993999 25.371000 48.997002

H1 52.838001 24.908001 48.806999

H2 51.848999 25.844999 48.157001

O 32.699001 18.877001 63.196999

H1 32.301998 19.334000 63.955002

H2 33.548000 18.573999 63.583000

O 6.803000 34.382000 25.044001

H1 7.125000 33.687000 25.636000

H2 5.972000 34.652000 25.472000

O 47.276001 45.266998 13.476000

H1 46.358002 45.423000 13.174000

H2 47.602001 44.676998 12.771000

O 26.964001 2.971000 41.098999

H1 26.948000 2.713000 40.167000

H2 26.091000 2.685000 41.410999

O 41.852001 9.399000 41.515999

H1 41.035000 9.043000 41.941002

H2 41.742001 10.349000 41.685001

O 58.550999 30.393000 44.483002

H1 57.900002 29.665001 44.377998

H2 59.256001 29.943001 44.988998

O 47.282001 58.174000 44.443001

H1 47.203999 58.188999 45.410999

H2 46.835999 58.998001 44.193001

O 50.570999 60.792999 33.037998

H1 51.492001 60.452999 33.044998

H2 50.731998 61.755001 33.021999

O 40.719002 16.750000 54.846001

H1 41.567001 17.028999 55.238998

H2 40.091999 17.018999 55.558998

O 27.920000 13.865000 16.618999

H1 28.594000 13.570000 17.266001

H2 27.653999 13.005000 16.232000

O 55.893002 39.258999 47.696999

H1 56.567001 38.570999 47.793999

H2 55.930000 39.713001 48.555000

O 30.233999 7.587000 25.113001

H1 29.750000 6.738000 25.027000

H2 29.497999 8.219000 25.160999

O 22.320999 48.290001 56.504002

H1 22.780001 48.685001 55.736000

H2 21.502001 48.806999 56.526001

O 40.639000 30.889999 11.201000

H1 41.021000 30.298000 10.540000

H2 39.903000 30.356001 11.552000

O 13.565000 30.216999 53.387001

H1 14.138000 29.434999 53.500000

H2 14.163000 30.936001 53.680000

O 49.801998 48.452999 8.501000

H1 49.716999 48.717999 9.430000

H2 48.887001 48.236000 8.269000

O 42.862999 36.169998 16.849001

H1 43.270000 36.841000 16.280001

H2 43.195999 35.346001 16.472000

O 24.386000 11.385000 17.254000

H1 24.389999 10.424000 17.077999

H2 25.055000 11.698000 16.618000

O 43.901001 55.256001 21.916000

H1 44.125000 56.150002 22.195999

H2 43.108002 55.053001 22.431000

O 15.983000 30.339001 30.427999

H1 16.277000 30.589001 31.316000

H2 15.375000 31.063999 30.205999

O 20.728001 3.327000 49.898998

H1 20.841000 2.848000 50.742001

H2 20.509001 4.224000 50.237000

O 11.045000 35.390999 35.868999

H1 10.368000 36.002998 36.200001

H2 10.719000 35.213001 34.967999

O 20.007000 56.233002 52.029999

H1 19.302999 56.035999 52.678001

H2 20.120001 55.360001 51.609001

O 41.668999 24.326000 42.195000

H1 42.508999 24.607000 42.610001

H2 41.520000 25.075001 41.581001

O 14.905000 20.018999 40.575001

H1 15.702000 19.933001 41.112999

H2 14.744000 19.091000 40.303001

O 30.122000 3.553000 35.389999

H1 29.931000 2.974000 34.645000

H2 30.893999 3.109000 35.803001

O 56.348000 32.006001 15.425000

H1 55.893002 31.205999 15.712000

H2 55.623001 32.554001 15.086000

O 12.932000 35.291000 53.067001

H1 12.817000 34.889000 53.948002

H2 12.224000 34.844002 52.562000

O 56.198002 62.622002 32.340000

H1 55.432999 63.212002 32.264000

H2 56.054001 62.007999 31.596001

O 27.341999 40.605000 16.174000

H1 27.327999 39.631001 16.105000

H2 26.395000 40.812000 16.260000

O 52.674999 56.783001 17.517000

H1 52.904999 57.653999 17.143999

H2 53.278999 56.200001 17.027000

O 40.217999 43.584999 17.966000

H1 39.813999 43.573002 18.864000

H2 39.445999 43.335999 17.426001

O 32.014999 4.188000 18.292999

H1 31.195999 4.014000 17.809000

H2 31.712000 4.216000 19.218000

O 37.622002 52.783001 16.731001

H1 38.084000 52.431999 15.955000

H2 37.382999 53.674000 16.445000

O 63.258999 39.994999 35.041000

H1 63.467999 39.092999 34.772999

H2 63.868999 40.132000 35.792999

O 37.879002 2.037000 29.761000

H1 37.075001 2.226000 29.242001

H2 37.605999 1.247000 30.261999

O 26.631001 46.605999 62.749001

H1 26.989000 47.062000 63.529999

H2 25.691000 46.854000 62.792000

O 57.660999 52.854000 35.506001

H1 58.415001 53.032001 34.918999

H2 57.484001 53.744999 35.868999

O 45.048000 64.306000 32.300999

H1 45.611000 65.100998 32.453999

H2 45.527000 63.882999 31.568001

O 19.455000 52.643002 13.347000

H1 19.402000 53.618999 13.341000

H2 20.350000 52.490002 13.018000

O 51.959000 27.431000 51.147999

H1 51.069000 27.139999 51.439999

H2 52.034000 26.969000 50.297001

O 9.251000 25.643000 47.782001

H1 8.839000 25.841000 46.924000

H2 10.105000 26.089001 47.707001

O 33.535000 47.297001 19.903000

H1 33.952999 46.492001 19.544001

H2 33.137001 47.680000 19.108999

O 27.548000 64.285004 30.524000

H1 26.804001 64.611000 29.996000

H2 27.084999 63.884998 31.287001

O 38.117001 40.473000 35.575001

H1 38.035000 40.497002 34.589001

H2 38.827000 41.164001 35.756001

O 42.340000 58.647999 22.882000

H1 41.542000 58.389999 22.396000

H2 42.959000 58.851002 22.153000

O 48.856998 48.769001 12.448000

H1 48.671001 47.847000 12.184000

H2 49.806000 48.733002 12.641000

O 21.079000 1.620000 39.916000

H1 21.533001 0.767000 40.034000

H2 20.985001 1.917000 40.835999

O 35.236000 15.781000 45.907001

H1 35.127998 15.526000 46.844002

H2 35.599998 16.684000 45.995998

O 16.544001 25.930000 50.541000

H1 16.705999 26.136999 49.612000

H2 15.797000 26.530001 50.749001

O 62.361000 23.613001 28.770000

H1 61.778000 23.024000 28.266001

H2 61.812000 23.844999 29.533001

O 9.496000 49.112000 48.207001

H1 8.982000 49.001999 47.397999

H2 10.291000 49.564999 47.896999

O 18.658001 31.650999 14.701000

H1 19.254999 32.077999 15.353000

H2 19.086000 30.764999 14.634000

O 34.161999 57.618000 45.050999

H1 34.139999 56.778000 44.554001

H2 35.102001 57.870998 44.966000

O 55.688999 56.387001 23.506001

H1 55.013000 55.706001 23.290001

H2 55.463001 56.587002 24.431999

O 9.670000 21.226999 43.693001

H1 9.452000 21.973000 44.291000

H2 8.810000 21.059000 43.278000

O 40.492001 53.515999 13.551000

H1 39.574001 53.576000 13.876000

H2 40.992001 53.865002 14.316000

O 48.799000 38.715000 65.675003

H1 48.757999 38.880001 64.724998

H2 49.729000 38.465000 65.808998

O 39.089001 14.657000 58.224998

H1 39.011002 13.702000 57.998001

H2 40.053001 14.721000 58.370998

O 11.018000 36.983002 39.798000

H1 11.309000 37.772999 40.268002

H2 10.762000 37.355000 38.926998

O 16.306000 27.614000 14.658000

H1 16.011000 26.863001 15.206000

H2 16.674999 28.215000 15.315000

O 39.804001 21.273001 64.283997

H1 39.083000 21.615999 63.737000

H2 40.056999 22.049000 64.808998

O 25.548000 24.575001 62.818001

H1 25.302000 25.180000 63.535000

H2 25.201000 25.034000 62.035000

O 12.612000 35.691002 49.116001

H1 12.960000 35.773998 48.214001

H2 13.171000 34.990002 49.487999

O 25.128000 53.973999 17.455000

H1 25.333000 54.812000 17.010000

H2 24.311001 54.186001 17.927999

O 56.122002 25.006001 49.566002

H1 55.301998 25.165001 49.064999

H2 55.831001 25.195000 50.473999

O 43.659000 48.069000 51.358002

H1 44.053001 47.570000 52.097000

H2 42.997002 48.611000 51.812000

O 27.931000 6.141000 21.773001

H1 27.225000 6.004000 21.118000

H2 28.209000 5.233000 21.972000

O 44.401001 19.590000 24.767000

H1 44.771000 19.289000 25.613001

H2 43.456001 19.660999 24.968000

O 28.889999 5.362000 24.868999

H1 29.080000 5.047000 23.971001

H2 27.966999 5.079000 24.990000

O 45.172001 44.679001 48.544998

H1 44.808998 44.257999 49.341000

H2 44.377998 45.080002 48.151001

O 29.438999 35.719002 7.467000

H1 29.858999 34.845001 7.431000

H2 29.309000 35.919998 6.523000

O 33.019001 12.736000 16.677000

H1 33.620998 12.510000 17.405001

H2 32.173000 12.850000 17.132000

O 41.035999 24.580999 16.443001

H1 41.198002 24.410000 17.386999

H2 40.241001 25.134001 16.469000

O 32.573002 39.536999 68.157997

H1 32.796001 40.487999 68.135002

H2 33.125999 39.192001 67.432999

O 37.659000 20.195000 43.563999

H1 37.118000 20.457001 42.804001

H2 38.351002 20.872999 43.568001

O 58.304001 22.868999 42.584999

H1 58.889000 23.650000 42.702999

H2 58.749001 22.211000 43.134998

O 26.715000 51.877998 23.281000

H1 26.523001 51.825001 22.325001

H2 27.646000 51.562000 23.289000

O 48.379002 12.659000 35.492001

H1 48.921001 11.836000 35.421001

H2 47.561001 12.295000 35.874001

O 47.740002 54.685001 12.534000

H1 48.455002 54.527000 13.183000

H2 47.264999 55.418999 12.958000

O 56.080002 39.978001 29.521000

H1 56.974998 39.594002 29.443001

H2 56.278000 40.859001 29.881001

O 42.561001 36.687000 19.566000

H1 42.688999 36.504002 18.611000

H2 42.831001 35.834000 19.955000

O 10.306000 12.636000 36.575001

H1 10.010000 12.090000 35.821999

H2 10.044000 13.522000 36.287998

O 51.862999 27.468000 13.569000

H1 51.243999 27.455999 14.327000

H2 51.346001 27.948000 12.905000

O 45.407001 30.094999 23.806000

H1 46.313000 29.837000 23.555000

H2 45.124001 29.330999 24.332001

O 4.805000 46.931000 29.158001

H1 4.264000 47.641998 29.530001

H2 5.667000 47.355999 29.056000

O 33.570999 63.668999 52.321999

H1 33.527000 64.629997 52.431000

H2 33.604000 63.576000 51.349998

O 13.020000 25.806000 18.285999

H1 12.090000 25.615999 18.084000

H2 13.455000 24.971001 18.046000

O 52.910000 63.317001 22.480000

H1 52.436001 64.058998 22.868999

H2 53.266998 63.700001 21.659000

O 49.279999 16.525999 38.305000

H1 49.897999 16.348000 39.036999

H2 48.634998 17.114000 38.723999

O 13.216000 18.163000 36.179001

H1 12.740000 18.728001 36.818001

H2 13.439000 17.392000 36.709000

O 8.716000 24.907000 28.464001

H1 8.374000 24.072001 28.125999

H2 9.525000 24.614000 28.931000

O 33.082001 30.634001 60.868000

H1 32.469002 30.379000 60.157001

H2 33.715000 29.893999 60.853001

O 52.494999 31.555000 20.975000

H1 53.256001 31.355000 21.541000

H2 51.995998 32.179001 21.563999

O 23.889000 22.239000 14.481000

H1 24.440001 21.437000 14.490000

H2 23.858999 22.454000 13.539000

O 37.158001 3.100000 37.396000

H1 36.799999 3.527000 36.599998

H2 37.681999 3.830000 37.786999

O 28.254000 20.597000 52.290001

H1 29.020000 20.752001 51.707001

H2 27.865000 19.802999 51.856998

O 61.007000 30.309000 22.712000

H1 60.332001 30.382999 22.018000

H2 60.977001 29.353001 22.910000

O 41.518002 12.821000 32.271000

H1 40.889000 12.156000 31.926001

H2 40.917000 13.492000 32.629002

O 38.186001 17.266001 24.153000

H1 37.522999 17.850000 23.716000

H2 38.667999 17.910000 24.691999

O 41.229000 3.072000 23.149000

H1 41.873001 3.790000 23.274000

H2 40.384998 3.584000 23.193001

O 62.723999 35.648998 31.598000

H1 62.208000 36.307999 32.103001

H2 63.000000 35.042000 32.303001

O 30.448000 54.096001 12.846000

H1 31.190001 54.487000 12.320000

H2 30.636999 54.459999 13.723000

O 25.042999 56.171001 28.795000

H1 25.528999 57.028000 28.922001

H2 24.143999 56.504002 28.645000

O 49.601002 16.733999 52.918999

H1 49.127998 16.811001 53.766998

H2 49.585999 15.776000 52.769001

O 39.666000 13.112000 46.382999

H1 40.356998 12.759000 46.962002

H2 39.317001 12.326000 45.953999

O 34.728001 0.287000 26.134001

H1 34.484001 0.765000 25.308001

H2 34.681000 -0.641000 25.798000

O 26.403999 58.741001 47.894001

H1 26.975000 59.012001 48.633999

H2 26.590000 57.792999 47.826000

O 53.056000 7.666000 32.187000

H1 53.577999 8.031000 31.450001

H2 53.534000 8.009000 32.958000

O 18.556999 8.354000 52.244999

H1 17.691999 7.925000 52.118000

H2 18.886999 7.879000 53.039001

O 65.555000 30.231001 23.766001

H1 66.195000 29.555000 23.488001

H2 65.277000 29.900000 24.634001

O 14.017000 55.460999 25.840000

H1 13.160000 55.016998 25.686001

H2 14.220000 55.762001 24.923000

O 7.314000 39.433998 42.498001

H1 7.225000 39.632999 41.549999

H2 7.594000 40.300999 42.851002

O 14.813000 27.966000 54.272999

H1 15.438000 27.582001 53.641998

H2 15.308000 27.945999 55.103001

O 42.167000 51.507000 12.947000

H1 42.772999 51.335999 13.678000

H2 41.712002 52.321999 13.240000

O 11.578000 4.785000 31.202999

H1 10.922000 4.123000 31.455999

H2 11.262000 5.068000 30.330000

O 24.306999 2.148000 42.553001

H1 23.747999 1.550000 42.042000

H2 23.697001 2.479000 43.231998

O 6.230000 39.945999 54.661999

H1 6.401000 39.441002 53.862999

H2 5.291000 39.744999 54.844002

O 31.048000 39.317001 63.737000

H1 31.959999 39.605999 63.944000

H2 31.010000 39.462002 62.782001

O 46.235001 31.601000 27.451000

H1 46.123001 32.098000 26.601000

H2 47.022999 31.063999 27.205999

O 52.675999 25.771000 27.038000

H1 51.884998 25.665001 26.490000

H2 53.283001 26.243999 26.458000

O 24.819000 50.990002 57.200001

H1 24.957001 51.872002 56.820000

H2 24.466000 50.497002 56.431999

O 52.568001 21.857000 29.285999

H1 52.014999 21.372999 29.910999

H2 53.174000 21.153000 28.966999

O 49.493000 59.846001 17.712000

H1 48.512001 59.887001 17.787001

H2 49.682999 60.696999 17.292000

O 49.491001 47.048000 52.353001

H1 49.659000 46.212002 52.812000

H2 50.012001 47.681000 52.882999

O 11.512000 49.335999 28.160999

H1 12.305000 49.817001 28.452000

H2 11.087000 49.994999 27.577999

O 60.488998 42.573002 25.042999

H1 60.962002 42.105999 25.771000

H2 60.452999 43.474998 25.408001

O 51.134998 52.407001 32.858002

H1 50.212002 52.492001 32.563000

H2 51.258999 53.238998 33.347000

O 10.030000 41.452000 38.167000

H1 10.162000 42.238998 37.617001

H2 10.923000 41.280998 38.498001

O 51.053001 34.290001 65.458000

H1 50.167999 34.708000 65.454002

H2 51.047001 33.816002 66.301003

O 43.352001 24.957001 57.865002

H1 43.826000 25.408001 58.596001

H2 42.814999 25.686001 57.518002

O 49.282001 30.877001 28.768000

H1 49.402000 29.936001 29.032000

H2 49.115002 30.742001 27.799999

O 58.658001 46.084000 27.663000

H1 58.290001 46.762001 27.070000

H2 59.576000 46.396999 27.767000

O 57.782001 55.216000 22.094999

H1 57.213001 55.676998 22.743999

H2 58.258999 54.596001 22.671000

O 49.936001 10.721000 36.178001

H1 50.132999 10.605000 37.113998

H2 50.172001 9.868000 35.794998

O 7.195000 48.486000 42.889999

H1 7.460000 48.528999 43.826000

H2 6.573000 49.234001 42.828999

O 51.535000 22.113001 37.709999

H1 51.294998 21.316999 38.207001

H2 52.384998 21.858000 37.313000

O 16.170000 19.261999 17.712000

H1 15.483000 19.080000 18.379999

H2 15.686000 19.851000 17.104000

O 40.932999 20.148001 16.457001

H1 40.757999 19.212999 16.632000

H2 41.812000 20.271999 16.849001

O 45.264000 49.826000 32.869999

H1 45.578999 49.735001 31.934999

H2 44.307999 49.967999 32.706001

O 39.200001 28.084999 19.898001

H1 38.417000 28.657000 19.990999

H2 38.817001 27.329000 19.403999

O 36.118999 60.563000 31.466000

H1 35.269001 60.840000 31.090000

H2 36.659000 60.425999 30.671000

O 9.264000 38.231998 35.361000

H1 8.925000 38.353001 34.446999

H2 10.158000 38.630001 35.269001

O 40.935001 12.379000 26.089001

H1 41.251999 13.237000 26.393999

H2 41.779999 11.928000 25.868000

O 16.386999 5.051000 42.381001

H1 16.834999 5.917000 42.433998

H2 15.447000 5.341000 42.344002

O 54.973999 49.766998 23.268999

H1 55.235001 50.555000 22.761999

H2 55.417000 49.914001 24.121000

O 47.696999 35.055000 11.318000

H1 47.202999 34.221001 11.193000

H2 47.566002 35.195999 12.280000

O 51.951000 52.903000 16.495001

H1 51.036999 52.915001 16.170000

H2 51.876999 53.438000 17.311001

O 16.011000 18.969000 53.294998

H1 16.936001 18.709999 53.400002

H2 15.788000 18.601000 52.421001

O 11.408000 15.888000 35.785000

H1 11.792000 16.783001 35.721001

H2 10.620000 16.003000 35.198002

O 61.036999 43.367001 16.912001

H1 61.282001 43.570999 17.834000

H2 60.973999 42.403999 16.924999

O 57.751999 52.095001 43.791000

H1 56.995998 51.873001 43.222000

H2 57.755001 53.061001 43.764000

O 50.424999 63.546001 38.087002

H1 50.541000 63.443001 37.131001

H2 50.625000 64.481003 38.226002

O 34.570999 31.823000 64.984001

H1 34.550999 30.857000 64.861000

H2 35.096001 32.105000 64.210999

O 35.067001 46.043999 18.013000

H1 35.021999 45.682999 17.117001

H2 35.509998 46.893002 17.879999

O 30.796000 33.808998 16.889999

H1 31.570999 34.235001 16.497000

H2 31.062000 33.716999 17.820000

O 56.368999 41.983002 31.461000

H1 57.248001 41.583000 31.375000

H2 56.092999 41.646000 32.344002

O 51.548000 46.157001 15.653000

H1 50.582001 46.214001 15.491000

H2 51.807999 45.518002 14.955000

O 61.971001 35.237999 29.070999

H1 61.195000 34.751999 29.436001

H2 62.481998 35.382000 29.896999

O 11.719000 47.903999 36.617001

H1 10.993000 48.421001 37.008999

H2 11.253000 47.125000 36.277000

O 34.537998 57.374001 51.467999

H1 33.851002 58.002998 51.745998

H2 34.028999 56.535999 51.450001

O 34.925999 21.025000 22.469000

H1 34.015999 20.704000 22.569000

H2 34.852001 21.938000 22.826000

O 30.325001 57.747002 52.958000

H1 30.914000 58.317001 52.416000

H2 30.004000 57.118000 52.293999

O 38.530998 21.323999 17.584000

H1 38.094002 20.893999 16.837999

H2 39.387001 20.868999 17.615000

O 33.969002 60.963001 47.478001

H1 34.376999 60.250000 48.021000

H2 34.026001 60.583000 46.591000

O 42.160000 20.450001 21.294001

H1 42.584999 20.462000 20.420000

H2 41.507000 19.747999 21.204000

O 17.434999 12.873000 26.792999

H1 18.211000 13.457000 26.851000

H2 17.011999 13.026000 27.662001

O 20.910000 29.194000 56.803001

H1 20.389999 28.813000 57.527000

H2 20.221001 29.444000 56.168999

O 39.145000 15.747000 46.637001

H1 38.591999 15.936000 47.410999

H2 39.228001 14.768000 46.695000

O 34.626999 5.521000 18.893999

H1 34.492001 5.690000 19.846001

H2 34.674999 4.549000 18.879000

O 36.361000 18.236000 45.948002

H1 35.971001 19.059999 46.266998

H2 36.855000 18.520000 45.167000

O 52.957001 36.265999 40.855000

H1 52.480000 35.431000 40.658001

H2 53.775002 36.124001 40.331001

O 58.674999 20.483000 24.482000

H1 58.597000 21.157000 23.788000

H2 58.263000 20.934999 25.243999

O 11.469000 15.530000 28.063999

H1 11.606000 16.497000 28.007999

H2 11.453000 15.284000 27.117001

O 29.292999 33.646999 13.875000

H1 28.447001 33.230999 13.642000

H2 29.920000 32.922001 13.743000

O 40.513000 38.415001 14.047000

H1 40.990002 39.191002 13.684000

H2 40.169998 38.799000 14.883000

O 42.568001 24.275999 24.712000

H1 42.626999 23.556999 24.034000

H2 41.669998 24.600000 24.538000

O 54.909000 54.191002 40.209999

H1 55.344002 54.921001 39.722000

H2 55.272999 53.415001 39.752998

O 11.962000 47.056999 56.327999

H1 11.330000 47.300999 55.616001

H2 12.073000 47.903999 56.785999

O 60.401001 46.493000 36.294998

H1 60.060001 45.702999 36.761002

H2 59.589001 47.028000 36.219002

O 26.646000 51.403999 20.507999

H1 26.895000 50.831001 19.763000

H2 25.673000 51.438999 20.407000

O 36.590000 61.709000 44.596001

H1 36.612999 62.483002 43.993000

H2 37.318001 61.903000 45.196999

O 42.568001 32.153999 14.872000

H1 41.841000 32.352001 15.485000

H2 43.283001 32.707001 15.220000

O 17.549999 16.416000 23.034000

H1 17.018000 16.517000 23.841999

H2 17.634001 17.332001 22.730000

O 19.285000 52.881001 18.278000

H1 19.039000 52.222000 18.962999

H2 18.566000 53.525002 18.367001

O 23.042000 29.017000 13.130000

H1 23.402000 28.101999 13.165000

H2 22.118000 28.837000 12.879000

O 42.723000 57.845001 37.056000

H1 43.661999 57.999001 36.827999

H2 42.549999 58.606998 37.640999

O 45.848000 55.949001 46.592999

H1 45.175999 55.743000 45.928001

H2 46.535000 55.285999 46.403000

O 15.394000 56.858002 29.766001

H1 16.129999 56.816002 30.399000

H2 15.235000 57.813000 29.705000

O 19.129999 51.292999 15.731000

H1 19.254000 52.078999 16.290001

H2 19.107000 51.688999 14.840000

O 46.683998 36.507000 18.215000

H1 46.712002 37.063999 19.042999

H2 46.523998 35.633999 18.646999

O 8.865000 37.029999 42.875999

H1 8.356000 37.819000 42.622002

H2 8.377000 36.323002 42.404999

O 51.615002 25.253000 20.093000

H1 50.943001 24.542000 20.068001

H2 52.374001 24.785999 20.476000

O 20.266001 55.915001 33.096001

H1 21.184000 56.155998 33.317001

H2 20.266001 54.957001 33.259998

O 16.570000 57.979000 39.773998

H1 16.104000 58.365002 40.542000

H2 17.143000 57.324001 40.201000

O 17.270000 13.101000 24.188000

H1 16.507000 13.687000 24.108000

H2 17.288000 12.926000 25.155001

O 37.140999 17.657000 51.631001

H1 36.445999 18.162001 51.150002

H2 37.148998 18.156000 52.480999

O 37.317001 51.286999 55.112999

H1 36.820999 52.098000 55.331001

H2 38.155998 51.431000 55.564999

O 36.471001 32.716999 25.573000

H1 37.082001 32.733002 26.334999

H2 35.629002 32.963001 26.023001

O 52.258999 15.296000 20.426001

H1 52.119999 14.502000 19.877001

H2 51.334000 15.521000 20.665001

O 21.385000 15.535000 51.792999

H1 20.575001 15.404000 51.283001

H2 21.479000 16.496000 51.806000

O 38.207001 64.078003 32.756001

H1 38.426998 63.299000 33.331001

H2 37.327000 64.305000 33.117001

O 23.791000 42.341000 21.857000

H1 23.402000 41.504002 21.573000

H2 24.445999 42.512001 21.164000

O 50.077000 60.491001 28.393000

H1 49.826000 61.436001 28.377001

H2 49.515999 60.165001 29.118999

O 28.086000 53.402000 61.339001

H1 28.082001 53.495998 60.359001

H2 28.738001 52.674999 61.432999

O 27.487000 49.914001 18.073000

H1 26.587000 49.806000 17.730000

H2 27.768999 50.744999 17.667999

O 19.704000 29.243999 14.522000

H1 19.341999 28.450001 14.957000

H2 20.146999 28.865000 13.748000

O 37.224998 35.609001 68.675003

H1 38.056999 36.060001 68.922997

H2 37.321999 35.583000 67.700996

O 39.874001 49.083000 59.256001

H1 40.618000 48.841999 59.833000

H2 39.123001 48.659000 59.694000

O 39.105000 60.650002 38.750999

H1 39.923000 60.808998 39.250000

H2 39.362999 60.859001 37.841999

O 22.250999 60.813000 36.299000

H1 21.570999 60.209999 36.610001

H2 21.990999 60.976002 35.376999

O 54.546001 40.368000 25.374001

H1 53.993000 39.653999 25.719999

H2 53.921001 41.120998 25.379999

O 54.051998 46.945000 19.375000

H1 53.334999 47.290001 19.938000

H2 54.820999 47.020000 19.961000

O 31.775999 6.713000 22.792000

H1 31.693001 7.335000 22.034000

H2 31.152000 7.092000 23.426001

O 18.975000 17.113001 51.367001

H1 18.812000 16.752001 52.250999

H2 18.125000 16.931999 50.921001

O 48.136002 62.563000 17.941999

H1 48.500999 63.474998 17.896999

H2 47.335999 62.701000 18.471001

O 36.414001 9.949000 40.930000

H1 36.924000 9.369000 40.347000

H2 36.238998 9.351000 41.693001

O 44.160000 37.574001 69.360001

H1 44.484001 36.662998 69.174004

H2 44.948002 37.957001 69.785004

O 51.632000 23.724001 61.985001

H1 51.039001 23.837999 62.747002

H2 51.629002 24.617001 61.598000

O 39.308998 50.127998 48.855000

H1 38.594002 50.805000 48.855000

H2 38.789001 49.313999 48.762001

O 48.662998 60.174000 31.164000

H1 48.667000 59.264999 31.518999

H2 49.507999 60.509998 31.528000

O 19.796000 56.013000 55.034000

H1 20.639999 55.564999 55.223000

H2 19.704000 56.611000 55.785000

O 23.702999 52.798000 53.459000

H1 23.660000 53.313000 52.633999

H2 22.857000 52.306000 53.428001

O 13.890000 43.749001 32.284000

H1 14.353000 43.013000 32.724998

H2 13.865000 44.419998 32.976002

O 29.966999 35.398998 10.627000

H1 29.931000 36.188999 10.057000

H2 29.018999 35.155998 10.666000

O 41.382000 44.643002 14.509000

H1 42.112999 44.577999 15.148000

H2 41.001999 45.506001 14.732000

O 3.118000 45.418999 27.108999

H1 2.595000 45.041000 27.830000

H2 3.750000 45.981998 27.582001

O 11.529000 46.674999 17.955999

H1 12.126000 46.042000 17.500000

H2 11.920000 47.527000 17.657000

O 46.569000 38.933998 58.480000

H1 45.694000 39.112000 58.070999

H2 46.431000 39.313999 59.369999

O 29.811001 63.209999 48.799000

H1 30.200001 62.837002 49.616001

H2 29.818001 62.448002 48.202999

O 64.239998 34.952000 27.409000

H1 64.476997 34.132999 27.889999

H2 63.479000 35.257999 27.938999

O 38.987000 9.411000 36.472000

H1 38.514999 10.261000 36.619999

H2 38.410999 8.796000 36.953999

O 19.039000 38.902000 19.016001

H1 18.886999 38.842999 19.972000

H2 19.305000 39.831001 18.908001

O 30.198999 9.746000 36.792999

H1 29.459000 10.317000 36.516998

H2 29.754000 9.187000 37.470001

O 22.959999 56.737999 18.507000

H1 22.122999 57.013000 18.094000

H2 23.514999 56.571999 17.728001

O 48.676998 50.020000 33.113998

H1 48.098000 50.473000 33.762001

H2 48.460999 50.512001 32.299000

O 59.118000 40.882000 31.458000

H1 59.349998 41.647999 32.019001

H2 59.027000 40.178001 32.131001

O 12.977000 28.489000 17.761000

H1 13.283000 28.254000 16.865999

H2 13.006000 27.618000 18.204000

O 45.117001 62.691002 34.598999

H1 44.881001 63.382999 33.957001

H2 44.466999 61.998001 34.389999

O 53.132000 41.384998 53.713001

H1 53.109001 41.564999 52.764000

H2 52.693001 40.521000 53.776001

O 60.508999 15.940000 36.877998

H1 60.046001 16.659000 36.417999

H2 60.551998 16.312000 37.793999

O 53.209999 19.438000 18.535000

H1 52.676998 18.926001 17.899000

H2 53.830002 18.774000 18.863001

O 17.111000 10.545000 45.869999

H1 17.136000 9.588000 45.653000

H2 17.377001 10.920000 45.004002

O 34.967999 47.145000 56.194000

H1 34.619999 48.042000 56.375999

H2 34.245998 46.584000 56.501999

O 53.056999 42.431000 40.106998

H1 52.686001 43.338001 40.209999

H2 53.639999 42.549000 39.345001

O 21.479000 26.774000 59.645000

H1 21.365000 26.348000 60.518002

H2 20.549000 26.971001 59.412998

O 24.434999 48.215000 65.603996

H1 24.254999 47.928001 64.685997

H2 24.790001 49.106998 65.469002

O 37.601002 22.167000 23.136999

H1 37.123001 23.003000 23.302000

H2 36.865002 21.587000 22.865999

O 51.933998 41.415001 7.816000

H1 51.980000 42.374001 7.616000

H2 51.831001 41.428001 8.781000

O 49.425999 39.854000 62.824001

H1 50.402000 39.984001 62.908001

H2 49.119999 40.716000 63.203999

O 27.260000 54.244999 21.038000

H1 27.169001 54.811001 20.254000

H2 26.789000 53.442001 20.764999

O 60.900002 37.797001 28.702000

H1 61.312000 37.055000 29.174000

H2 61.412998 37.771000 27.855000

O 26.302000 16.295000 55.549999

H1 26.070999 16.201000 54.605999

H2 26.100000 15.413000 55.891998

O 40.432999 59.674999 15.180000

H1 41.257000 59.187000 14.997000

H2 40.778999 60.486000 15.597000

O 16.910000 10.403000 28.624001

H1 16.035999 10.042000 28.426001

H2 16.823999 11.324000 28.325001

O 50.408001 66.024002 29.625000

H1 51.113998 66.074997 28.951000

H2 49.772999 65.428001 29.198999

O 8.492000 55.513000 42.210999

H1 9.468000 55.428001 42.250999

H2 8.273000 54.883999 41.511002

O 25.184000 1.520000 32.583000

H1 24.534000 1.043000 32.032001

H2 25.975000 0.955000 32.452999

O 35.521999 53.640999 52.299000

H1 34.895000 53.667999 51.558998

H2 34.967999 53.299000 53.018002

O 58.737999 34.021999 24.773001

H1 59.693001 34.091000 24.950001

H2 58.691002 34.270000 23.836000

O 31.862000 11.305000 52.752998

H1 31.544001 11.771000 51.959000

H2 32.104000 12.058000 53.328999

O 22.938000 14.590000 26.028000

H1 22.974001 13.633000 26.212999

H2 23.063000 14.967000 26.915001

O 21.157000 23.684999 65.198997

H1 22.073000 23.926001 65.382004

H2 21.105000 22.771000 65.521004

O 42.179001 64.985001 21.409000

H1 42.081001 65.954002 21.478001

H2 42.717999 64.791000 22.193001

O 18.457001 56.973999 44.109001

H1 18.566000 57.441002 44.959000

H2 19.072001 56.227001 44.219002

O 30.164000 24.429001 64.804001

H1 30.714001 24.965000 64.209000

H2 29.959000 23.660000 64.233002

O 38.910999 43.665001 45.421001

H1 38.817001 42.794998 45.867001

H2 38.564999 44.250999 46.143002

O 25.944000 26.259001 17.332001

H1 25.926001 25.941999 16.399000

H2 26.313999 25.459000 17.766001

O 29.559999 14.752000 22.253000

H1 29.823999 15.677000 22.382000

H2 28.792000 14.677000 22.847000

O 43.826000 19.794001 48.675999

H1 43.840000 19.146999 49.414001

H2 44.782001 19.927999 48.537998

O 35.282001 44.103001 66.390999

H1 34.623001 44.254002 65.679001

H2 35.102001 44.873001 66.969002

O 15.748000 30.233000 15.992000

H1 15.980000 31.171000 15.864000

H2 15.166000 30.066999 15.240000

O 16.229000 13.580000 49.374001

H1 15.260000 13.694000 49.417999

H2 16.309999 12.874000 48.710999

O 46.551998 47.189999 21.538000

H1 45.603001 47.261002 21.292000

H2 46.623001 47.987000 22.117001

O 46.814999 37.881001 20.431999

H1 46.442001 38.792000 20.562000

H2 46.526001 37.466000 21.264000

O 35.683998 65.060997 37.314999

H1 35.098999 64.567001 36.715000

H2 35.257999 65.921997 37.373001

O 10.959000 43.852001 20.544001

H1 10.898000 44.784000 20.809000

H2 10.235000 43.780998 19.900999

O 47.395000 31.549999 66.842003

H1 47.330002 30.802999 67.467003

H2 48.360001 31.722000 66.864998

O 17.434000 6.108000 36.667999

H1 17.308001 6.838000 37.293999

H2 17.863001 6.562000 35.919998

O 49.362000 64.792000 17.695999

H1 49.316002 65.269997 16.834999

H2 49.166000 65.528000 18.311001

O 37.644001 57.605999 19.110001

H1 36.754002 57.966000 18.927999

H2 37.542999 56.698002 18.750999

O 47.036999 44.043999 42.305000

H1 47.452999 43.937000 41.409000

H2 47.820000 43.796001 42.853001

O 39.972000 6.625000 40.231998

H1 40.931000 6.574000 40.368999

H2 39.688000 7.121000 41.021999

O 46.393002 54.792000 20.371000

H1 46.066002 55.645000 20.735001

H2 46.993999 54.508999 21.073999

O 65.126999 34.375999 37.195000

H1 65.458000 34.728001 38.053001

H2 65.723999 33.618000 37.071999

O 8.845000 33.786999 31.576000

H1 8.956000 33.664001 30.618000

H2 9.228000 32.967999 31.933001

O 39.921001 58.891998 21.149000

H1 39.071999 59.278999 20.895000

H2 39.931999 58.063999 20.643000

O 57.888000 25.636999 29.961000

H1 58.494999 25.382000 29.232000

H2 57.450001 24.790001 30.152000

O 65.429001 31.516001 21.224001

H1 65.148003 30.919001 21.930000

H2 64.634003 31.566000 20.660999

O 62.827999 31.730000 34.587002

H1 63.313999 32.522999 34.298000

H2 61.908001 32.020000 34.535999

O 14.011000 52.036999 47.368000

H1 14.118000 52.830002 46.808998

H2 13.900000 52.424999 48.247002

O 30.268000 27.125000 17.306000

H1 30.097000 26.278999 17.750000

H2 29.872000 26.975000 16.429001

O 41.800999 64.271004 43.150002

H1 42.747002 64.474998 43.049000

H2 41.389000 65.109001 42.894001

O 42.770000 53.393002 43.808998

H1 42.992001 53.290001 42.862999

H2 43.662998 53.417000 44.206001

O 22.514999 18.667000 51.737000

H1 21.816999 19.278999 52.007000

H2 23.034000 19.224001 51.120998

O 27.832001 22.285000 20.252001

H1 27.981001 22.902000 20.980000

H2 27.739000 21.434999 20.725000

O 39.743999 36.394001 69.053001

H1 39.827999 35.608002 69.626999

H2 40.660999 36.500999 68.741997

O 58.560001 49.905998 36.224998

H1 58.164001 50.742001 35.939999

H2 59.205002 50.209000 36.893002

O 28.705999 57.421001 50.441002

H1 28.966000 57.147999 49.536999

H2 28.409000 58.335999 50.284000

O 29.777000 59.256001 56.605999

H1 29.167000 58.695000 56.109001

H2 30.608999 59.144001 56.122002

O 50.509998 55.842999 26.295000

H1 49.762001 55.551998 25.738001

H2 50.873001 54.987000 26.584000

O 5.522000 40.813999 24.120001

H1 5.109000 40.589001 24.982000

H2 5.438000 41.787998 24.131001

O 49.543999 45.546001 37.779999

H1 48.570999 45.535000 37.874001

H2 49.785000 44.769001 38.327000

O 46.660000 46.466999 55.360001

H1 47.480000 46.869999 55.688999

H2 46.987000 45.583000 55.078999

O 47.327000 21.299999 51.516998

H1 47.409000 20.337999 51.346001

H2 46.425999 21.340000 51.893002

O 17.495001 37.839001 21.073000

H1 16.976000 37.012001 20.980000

H2 17.854000 37.742001 21.971001

O 61.540001 18.349001 31.875000

H1 60.682999 18.459000 32.353001

H2 61.324001 17.636999 31.261999

O 48.283001 25.867001 29.200001

H1 47.849998 25.743000 30.076000

H2 48.717999 24.988001 29.105000

O 47.278999 39.841000 60.941002

H1 47.837002 39.639000 61.713001

H2 47.910999 39.695000 60.209000

O 20.292999 30.528999 18.018999

H1 19.840000 29.809000 18.485001

H2 21.135000 30.113001 17.775000

O 63.415001 36.506001 19.782000

H1 63.466999 36.036999 20.631001

H2 62.523998 36.289001 19.478001

O 18.905001 42.352001 42.175999

H1 19.216999 43.202999 42.540001

H2 19.659000 41.770000 42.370998

O 35.554001 65.278000 46.091999

H1 35.803001 64.356003 46.259998

H2 34.999001 65.202003 45.299000

O 55.053001 50.856998 32.438999

H1 55.797001 50.805000 31.813999

H2 55.330002 50.241001 33.136002

O 14.184000 40.499001 19.438999

H1 14.858000 40.477001 20.155001

H2 14.602000 39.910999 18.782000

O 29.221001 14.065000 12.489000

H1 29.796000 13.556000 11.882000

H2 28.825001 13.345000 13.011000

O 46.898998 37.771000 11.281000

H1 47.236000 36.872002 11.104000

H2 46.363998 37.924000 10.472000

O 22.354000 7.921000 45.557999

H1 21.466999 7.802000 45.200001

H2 22.573999 7.015000 45.862999

O 40.153000 54.188999 47.633999

H1 40.726002 53.799000 48.325001

H2 39.382000 54.462002 48.155998

O 25.655001 20.056000 10.494000

H1 26.295000 20.384001 11.150000

H2 25.261999 20.874001 10.163000

O 12.918000 7.420000 44.337002

H1 13.319000 8.301000 44.293999

H2 13.259000 7.004000 43.528999

O 24.327000 10.167000 27.643999

H1 24.819000 10.060000 28.469000

H2 24.698999 9.462000 27.089001

O 35.478001 4.066000 35.235001

H1 34.563999 4.395000 35.365002

H2 35.348000 3.368000 34.582001

O 63.240002 40.290001 24.728001

H1 62.923000 39.772999 23.954000

H2 64.153999 40.493999 24.426001

O 6.974000 20.194000 43.264999

H1 6.873000 19.902000 42.338001

H2 6.993000 19.330999 43.729000

O 13.287000 45.798000 49.938999

H1 12.364000 45.582001 49.702999

H2 13.228000 45.912998 50.896000

O 49.562000 45.998001 41.582001

H1 49.501999 46.876999 41.134998

H2 49.035000 45.459000 40.963001

O 32.591000 14.785000 48.959000

H1 33.498001 14.695000 48.611000

H2 32.195999 15.408000 48.335999

O 22.252001 57.136002 28.687000

H1 22.587999 57.612999 29.469999

H2 21.295000 57.106998 28.885000

O 53.307999 31.834999 16.465000

H1 52.859001 30.988001 16.593000

H2 52.957001 32.115002 15.600000

O 42.910000 20.959000 18.587000

H1 42.493999 21.839001 18.520000

H2 43.840000 21.165001 18.389000

O 47.341999 56.639999 14.933000

H1 47.453999 57.518002 14.506000

H2 48.250000 56.280998 14.841000

O 49.766998 22.017000 26.400999

H1 49.221001 22.483999 25.740999

H2 50.666000 22.129999 26.021000

O 16.256001 19.577999 48.657001

H1 16.400000 19.933001 47.761002

H2 17.150000 19.653000 49.042999

O 47.008999 47.002998 30.757999

H1 46.827000 46.060001 30.958000

H2 47.804001 47.146999 31.298000

O 47.057999 11.738000 41.652000

H1 47.707001 11.651000 40.933998

H2 47.359001 12.568000 42.081001

O 10.893000 33.006001 58.504002

H1 11.671000 32.825001 57.932999

H2 10.798000 33.973000 58.375000

O 57.521999 47.908001 23.218000

H1 57.223999 48.375999 22.429001

H2 58.331001 48.410999 23.455000

O 34.755001 52.929001 11.904000

H1 34.713001 51.966999 11.723000

H2 33.977001 53.256001 11.438000

O 24.976000 8.586000 58.504002

H1 24.794001 8.119000 57.664001

H2 24.871000 7.883000 59.153999

O 62.574001 40.918999 20.320000

H1 62.076000 40.106998 20.122000

H2 62.077999 41.270000 21.082001

O 39.237999 55.523998 49.957001

H1 39.971001 55.839001 50.507000

H2 38.870998 54.810001 50.535999

O 28.004000 61.863998 54.625999

H1 27.368999 62.245998 55.252998

H2 28.825001 61.867001 55.159000

O 17.364000 59.277000 32.027000

H1 16.606001 58.750000 32.331001

H2 16.952000 60.113998 31.778000

O 30.249001 17.462999 50.022999

H1 31.113001 17.575001 49.604000

H2 30.427000 17.653000 50.950001

O 23.191999 54.056000 14.072000

H1 23.648001 53.332001 13.605000

H2 23.750000 54.818001 13.817000

O 35.012001 22.618999 50.500999

H1 35.842999 22.110001 50.598999

H2 35.334999 23.454000 50.136002

O 56.043999 25.674999 46.464001

H1 56.439999 25.933001 47.306000

H2 56.590000 24.930000 46.187000

O 30.517000 64.278999 44.905998

H1 31.313000 63.981998 45.377998

H2 30.893000 64.709999 44.123001

O 41.993999 17.386000 19.783001

H1 41.236000 17.983000 19.725000

H2 42.389999 17.472000 18.896999

O 28.007999 46.980000 15.328000

H1 28.357000 46.080002 15.241000

H2 28.723000 47.506001 14.906000

O 30.700001 20.014000 61.465000

H1 31.606001 20.105000 61.805000

H2 30.672001 19.065001 61.242001

O 48.744999 56.055000 29.368999

H1 48.153999 55.285999 29.410000

H2 49.147999 55.965000 28.496000

O 58.978001 53.145000 31.125999

H1 58.180000 53.687000 31.031000

H2 59.683998 53.777000 30.906000

O 39.834000 4.091000 19.985001

H1 39.391998 3.378000 19.514999

H2 39.221001 4.833000 19.872000

O 17.459999 46.748001 42.293999

H1 17.117001 47.384998 41.630001

H2 16.774000 46.049999 42.236000

O 14.933000 44.766998 22.872000

H1 15.550000 44.445000 23.548000

H2 14.147000 44.980999 23.400000

O 34.327999 54.289001 58.174000

H1 34.602001 53.646999 57.493999

H2 34.629002 55.126999 57.779999

O 27.552999 12.682000 53.077000

H1 26.621000 12.379000 53.015999

H2 27.533001 13.221000 53.876999

O 35.185001 11.809000 43.615002

H1 34.379002 12.279000 43.328999

H2 35.494999 12.353000 44.342999

O 37.320000 51.962002 49.006001

H1 37.527000 52.604000 49.708000

H2 37.011002 52.571999 48.293999

O 22.259001 54.780998 55.474998

H1 22.931000 54.494999 54.837002

H2 22.659000 54.518002 56.320999

O 15.288000 33.546001 34.964001

H1 15.797000 32.880001 34.476002

H2 14.405000 33.451000 34.540001

O 63.971001 29.375999 34.616001

H1 63.506001 30.233000 34.749001

H2 64.148003 29.433001 33.647999

O 28.472000 21.045000 54.991001

H1 28.667999 20.118000 55.264000

H2 28.591999 20.969999 54.025002

O 16.809999 16.223000 49.416000

H1 17.202999 16.323000 48.532001

H2 16.476000 15.306000 49.379002

O 37.646999 49.683998 15.705000

H1 38.369999 50.328999 15.789000

H2 37.695000 49.464001 14.751000

O 36.273998 61.224998 14.682000

H1 36.632999 61.388000 15.571000

H2 36.910999 60.569000 14.332000

O 56.953999 59.666000 28.455999

H1 57.797001 59.384998 28.861000

H2 56.744999 58.923000 27.884001

O 61.150002 31.886000 18.056999

H1 61.259998 31.875999 17.091999

H2 61.299999 30.952000 18.285000

O 42.821999 38.122002 39.307999

H1 42.855999 37.876999 40.266998

H2 43.782001 38.282001 39.153999

O 21.048000 5.821000 50.782001

H1 21.350000 6.187000 51.625999

H2 20.784000 6.633000 50.299000

O 19.313000 12.553000 22.179001

H1 18.423000 12.633000 22.563999

H2 19.639000 13.475000 22.271000

O 46.638000 20.888000 23.681999

H1 45.712002 20.577000 23.639000

H2 46.557999 21.646999 24.278999

O 50.305000 63.735001 35.041000

H1 49.958000 64.615997 35.285000

H2 49.890999 63.618000 34.162998

O 28.740999 0.823000 29.254000

H1 28.381001 0.151000 28.650999

H2 29.493000 1.169000 28.747999

O 44.254002 4.781000 24.361000

H1 43.453999 4.905000 24.896999

H2 44.369999 3.813000 24.406000

O 44.723000 58.526001 35.217999

H1 45.668999 58.433998 35.007999

H2 44.417999 59.112000 34.500999

O 26.153999 38.712002 13.207000

H1 26.517000 38.693001 14.115000

H2 25.726000 37.834000 13.169000

O 17.146000 41.433998 54.694000

H1 17.385000 42.367001 54.855000

H2 17.535000 41.279999 53.820000

O 23.232000 33.862000 59.856998

H1 23.622000 32.984001 60.058998

H2 22.305000 33.715000 60.118000

O 52.932999 56.821999 43.483002

H1 52.226002 56.926998 44.127998

H2 52.959999 55.867001 43.341000

O 43.821999 18.988001 60.601002

H1 42.949001 19.268000 60.284000

H2 44.082001 19.730000 61.165001

O 16.434000 32.544998 62.265999

H1 16.458000 31.722000 61.752998

H2 17.254000 32.485001 62.785000

O 21.791000 9.258000 39.521000

H1 21.426001 9.813000 40.224998

H2 21.579000 8.358000 39.848000

O 26.320999 55.995998 55.066002

H1 25.740999 56.355000 55.764999

H2 26.801001 56.798000 54.785000

O 15.691000 61.060001 42.467999

H1 16.417999 61.179001 41.838001

H2 16.129000 61.216000 43.317001

O 10.828000 16.502001 45.395000

H1 11.795000 16.375000 45.332001

H2 10.778000 17.434000 45.671001

O 61.479000 28.851000 18.295000

H1 61.305000 28.488001 17.408001

H2 60.639999 28.673000 18.750000

O 43.224998 45.537998 40.374001

H1 43.391998 45.257000 41.306000

H2 43.251999 46.507000 40.485001

O 38.602001 12.479000 14.801000

H1 39.235001 12.500000 14.070000

H2 38.719002 11.580000 15.153000

O 14.079000 56.563999 23.403999

H1 13.943000 55.998001 22.614000

H2 14.789000 57.158001 23.059999

O 18.802000 13.369000 44.087002

H1 19.389000 13.703000 43.379002

H2 19.451000 13.152000 44.780998

O 42.374001 27.125000 59.333000

H1 41.492001 27.520000 59.382999

H2 42.383999 26.539000 60.109001

O 48.757000 23.021000 61.639999

H1 49.445999 23.042000 60.963001

H2 49.167999 22.499001 62.346001

O 17.705999 21.194000 26.716000

H1 17.615999 21.226000 25.742001

H2 18.663000 21.042999 26.809999

O 52.576000 49.491001 10.074000

H1 53.256001 50.189999 10.104000

H2 51.757999 50.005001 10.188000

O 56.125000 21.884001 33.139999

H1 55.313000 21.450001 32.807999

H2 56.808998 21.420000 32.618999

O 11.097000 34.423000 51.098999

H1 11.294000 33.473999 51.174999

H2 11.494000 34.654999 50.247002

O 51.618000 39.881001 48.386002

H1 51.506001 40.485001 49.139999

H2 52.098999 39.139000 48.800999

O 21.520000 13.429000 42.903000

H1 21.337000 13.527000 41.955002

H2 22.476000 13.576000 42.939999

O 17.212999 53.693001 33.550999

H1 17.947001 53.319000 34.077000

H2 16.886000 54.395000 34.126999

O 20.915001 15.821000 29.166000

H1 20.476999 15.201000 28.563000

H2 20.958000 15.302000 29.992001

O 52.419998 24.275000 45.736000

H1 51.542999 24.641001 45.564999

H2 52.355000 23.978001 46.653000

O 45.389000 30.226999 56.180000

H1 45.752998 29.649000 56.869999

H2 46.151001 30.780001 55.944000

O 34.248001 2.731000 16.981001

H1 33.764000 2.139000 16.385000

H2 33.528999 3.043000 17.568001

O 34.368000 19.201000 55.701000

H1 34.931000 18.695999 56.292999

H2 34.652000 20.120001 55.873001

O 44.514000 12.627000 53.838001

H1 44.195000 13.093000 53.046001

H2 45.436001 12.427000 53.591000

O 9.454000 11.776000 34.001999

H1 8.966000 10.983000 33.716999

H2 8.963000 12.484000 33.549000

O 12.640000 25.913000 54.387001

H1 13.071000 26.785999 54.432999

H2 13.389000 25.323999 54.591999

O 22.360001 10.807000 44.445999

H1 22.455000 9.857000 44.581001

H2 23.257000 11.139000 44.590000

O 24.927999 63.033001 25.061001

H1 24.638000 62.187000 24.680000

H2 25.146000 63.547001 24.261000

O 34.622002 1.341000 23.629999

H1 33.700001 1.236000 23.351999

H2 35.117001 1.101000 22.837000

O 51.123001 8.431000 39.466999

H1 50.587002 8.211000 40.250999

H2 50.494999 8.218000 38.743999

O 30.125000 51.601002 61.754002

H1 31.038000 51.951000 61.807999

H2 30.028000 51.202000 62.639999

O 17.316000 19.347000 42.308998

H1 17.233000 18.438000 42.659000

H2 16.841000 19.861000 42.974998

O 18.823999 10.541000 50.632000

H1 18.496000 9.766000 51.125000

H2 18.145000 10.647000 49.943001

O 6.514000 39.608002 48.778000

H1 6.694000 38.653000 48.745998

H2 6.958000 39.860001 49.610001

O 17.402000 1.347000 32.424000

H1 17.106001 1.539000 33.333000

H2 16.669001 1.677000 31.889000

O 45.250000 23.733999 44.630001

H1 44.747002 23.323999 45.335999

H2 44.625000 24.364000 44.243999

O 45.914001 65.289001 40.213001

H1 46.258999 65.279999 41.118999

H2 45.326000 64.514999 40.209000

O 32.919998 6.189000 51.452999

H1 33.035999 6.528000 50.550999

H2 32.333000 6.876000 51.837002

O 12.941000 22.016001 39.509998

H1 12.588000 22.500999 40.270000

H2 13.593000 21.424999 39.922001

O 33.076000 50.438999 50.984001

H1 32.771999 50.328999 51.914001

H2 33.196999 49.493999 50.740002

O 24.660000 33.456001 16.097000

H1 24.431000 32.646999 15.595000

H2 25.393999 33.147999 16.649000

O 27.964001 58.067001 54.417999

H1 27.365999 58.823002 54.456001

H2 28.468000 58.241001 53.602001

O 32.284000 55.096001 48.228001

H1 32.492001 56.000000 47.928001

H2 32.851002 55.001999 49.005001

O 16.721001 8.851000 37.203999

H1 15.964000 8.597000 37.763000

H2 16.267000 9.422000 36.537998

O 37.637001 0.912000 26.204000

H1 36.730999 0.567000 26.148001

H2 37.959999 0.493000 27.018000

O 61.855999 50.157001 40.293999

H1 61.236000 49.502998 39.933998

H2 61.368000 50.498001 41.061001

O 8.886000 42.147999 32.243000

H1 8.134000 41.952999 31.665001

H2 9.593000 41.615002 31.827000

O 38.693001 43.738998 20.264999

H1 39.071999 44.001999 21.138000

H2 38.189999 42.938000 20.546000

O 33.497002 47.405998 9.030000

H1 34.333000 46.952000 9.183000

H2 33.424999 47.990002 9.803000

O 13.854000 36.480000 27.507999

H1 13.492000 37.213001 26.969999

H2 13.330000 35.727001 27.172001

O 33.014999 49.393002 62.625999

H1 33.419998 50.138000 62.157001

H2 33.748001 49.046001 63.147999

O 14.202000 61.571999 36.841999

H1 13.291000 61.423000 36.534000

H2 14.715000 61.435001 36.033001

O 3.847000 41.521999 29.679001

H1 3.988000 42.478001 29.813000

H2 3.330000 41.525002 28.848000

O 9.660000 43.591000 56.306999

H1 8.946000 43.116001 55.855999

H2 10.409000 43.446999 55.695000

O 27.372999 0.360000 31.575001

H1 27.864000 0.648000 30.771999

H2 27.398001 -0.608000 31.457001

O 52.866001 43.610001 44.916000

H1 52.832001 43.103001 45.740002

H2 51.988998 44.028000 44.891998

O 46.575001 21.007000 57.532001

H1 46.820999 21.707001 58.154999

H2 46.672001 21.462000 56.672001

O 59.574001 14.428000 32.231998

H1 58.750999 14.495000 32.730999

H2 60.248001 14.551000 32.933998

O 30.344999 51.400002 53.713001

H1 30.540001 50.542000 54.122002

H2 29.896000 51.868999 54.431999

O 18.923000 28.118999 19.424000

H1 18.641001 27.236000 19.730000

H2 18.096001 28.400000 18.955999

O 46.862000 18.309999 17.205999

H1 47.379002 17.818001 16.547001

H2 47.231998 17.957001 18.042999

O 31.237000 3.476000 47.966999

H1 30.337000 3.603000 48.330002

H2 31.079000 2.837000 47.259998

O 20.737000 43.743999 11.365000

H1 20.396000 42.828999 11.308000

H2 20.073999 44.166000 11.930000

O 38.813999 63.638000 23.021000

H1 39.187000 63.535000 22.125000

H2 39.632000 63.629002 23.563999

O 47.025002 14.902000 19.724001

H1 47.230000 13.960000 19.615000

H2 46.397999 15.059000 18.996000

O 4.449000 38.963001 51.229000

H1 5.105000 39.617001 50.952999

H2 5.009000 38.296001 51.673000

O 36.271999 43.176998 44.103001

H1 37.168999 43.501999 44.314999

H2 35.943001 42.978001 45.008999

O 10.807000 51.681000 37.988998

H1 10.995000 52.141998 38.828999

H2 10.043000 51.131001 38.241001

O 16.698000 34.912998 32.668999

H1 15.860000 35.416000 32.616001

H2 16.396000 34.097000 33.101002

O 47.091999 13.915000 47.165001

H1 46.367001 13.464000 46.682999

H2 47.478001 13.156000 47.648998

O 58.754002 24.487000 23.521000

H1 59.698002 24.548000 23.278999

H2 58.682999 25.215000 24.169001

O 26.295000 63.061001 34.837002

H1 26.563000 63.416000 35.716000

H2 26.174000 62.118999 35.060001

O 26.927999 56.436001 18.843000

H1 26.933001 57.028000 18.075001

H2 27.851000 56.102001 18.832001

O 26.819000 47.358002 58.370998

H1 27.308001 46.851002 57.692001

H2 26.976999 46.806000 59.164001

O 45.459000 47.814999 7.368000

H1 45.431999 46.958000 7.827000

H2 45.855000 47.563000 6.512000

O 50.092999 38.953999 56.530998

H1 50.268002 38.040001 56.223000

H2 50.981998 39.339001 56.501999

O 18.493000 39.958000 68.259003

H1 18.480000 39.932999 67.290001

H2 18.635000 40.928001 68.413002

O 34.700001 48.417000 14.139000

H1 35.089001 49.023998 14.782000

H2 33.993999 47.990002 14.657000

O 40.029999 40.632000 44.270000

H1 39.532001 40.816002 45.091999

H2 40.109001 41.519001 43.890999

O 7.543000 22.044001 39.187000

H1 7.951000 22.174000 38.308998

H2 8.160000 22.541000 39.752998

O 44.522999 63.486000 20.476000

H1 43.702000 63.964001 20.677000

H2 44.222000 62.820999 19.841999

O 33.703999 24.986000 14.908000

H1 33.917999 25.898001 14.639000

H2 32.889000 24.825001 14.399000

O 9.132000 44.884998 31.912001

H1 9.998000 44.942001 31.478001

H2 8.951000 43.928001 31.879000

O 51.404999 21.469999 22.687000

H1 52.015999 21.983999 22.135000

H2 50.700001 22.101999 22.863001

O 57.216000 33.019001 52.868000

H1 57.980999 32.992001 52.264999

H2 57.627998 33.332001 53.691002

O 40.543999 46.962002 11.744000

H1 40.688000 47.712002 12.366000

H2 39.604000 46.766998 11.918000

O 16.474001 53.368000 41.591000

H1 16.749001 53.641998 42.487000

H2 15.509000 53.297001 41.701000

O 42.596001 22.761000 22.608000

H1 42.459999 21.816999 22.399000

H2 42.042000 23.188999 21.931000

O 15.507000 11.029000 55.124001

H1 15.713000 11.524000 54.307999

H2 15.057000 11.719000 55.653999

O 42.909000 42.280998 48.972000

H1 42.018002 42.566002 49.261002

H2 42.792000 42.323002 47.993999

O 19.576000 3.602000 44.044998

H1 19.959999 4.431000 43.703999

H2 19.794001 3.648000 44.986000

O 13.053000 59.755001 39.016998

H1 13.819000 60.206001 38.622002

H2 13.470000 59.280998 39.762001

O 5.340000 35.368000 29.108000

H1 4.369000 35.492001 29.143999

H2 5.398000 34.405998 28.987000

O 36.182999 50.053001 50.883999

H1 36.237999 50.813999 50.280998

H2 35.229000 49.873001 50.896999

O 45.904999 12.171000 36.967999

H1 45.289001 12.296000 37.709000

H2 45.306000 11.934000 36.243999

O 43.916000 51.874001 22.076000

H1 44.126999 51.389000 22.906000

H2 43.379002 52.608002 22.438999

O 54.520000 26.865000 44.672001

H1 55.157001 26.458000 45.312000

H2 53.830002 27.167000 45.282001

O 44.478001 35.298000 11.753000

H1 43.686001 35.735001 12.093000

H2 44.729000 35.858002 11.008000

O 19.268999 17.934000 28.283001

H1 19.791000 18.667000 27.902000

H2 19.958000 17.261999 28.429001

O 26.349001 10.739000 25.320000

H1 25.922001 11.580000 25.551001

H2 25.731001 10.083000 25.660999

O 7.936000 41.175999 29.542000

H1 7.689000 42.118999 29.429001

H2 8.587000 41.066002 28.827000

O 16.497999 54.883999 19.167999

H1 16.490999 55.851002 19.112000

H2 16.489000 54.757000 20.149000

O 47.894001 39.080002 17.466000

H1 48.838001 38.905998 17.283001

H2 47.592999 38.187000 17.721001

O 10.158000 38.176998 29.466999

H1 10.428000 37.247002 29.450001

H2 9.528000 38.212002 28.721001

O 5.334000 35.143002 42.818001

H1 6.179000 35.021999 42.338001

H2 5.034000 34.222000 42.901001

O 29.819000 6.241000 31.399000

H1 30.289000 5.483000 31.010000

H2 29.084000 6.359000 30.767000

O 53.255001 22.974001 21.291000

H1 53.587002 22.063999 21.274000

H2 53.556000 23.299000 20.412001

O 37.931999 14.901000 60.929001

H1 36.980999 14.747000 60.758999

H2 38.323002 14.686000 60.067001

O 53.277000 58.495998 41.028000

H1 53.569000 57.630001 41.348999

H2 54.039001 58.790001 40.504002

O 43.620998 50.186001 6.949000

H1 44.209000 49.521000 7.342000

H2 42.793999 49.675999 6.837000

O 33.332001 1.139000 38.875000

H1 33.694000 1.544000 39.679001

H2 34.049999 1.296000 38.231998

O 52.658001 31.818001 42.294998

H1 52.880001 30.865000 42.341999

H2 52.417000 31.996000 43.223000

O 18.249001 47.077000 15.581000

H1 17.893999 47.056999 14.684000

H2 19.205000 47.130001 15.431000

O 23.698999 6.844000 35.824001

H1 23.225000 7.557000 36.303001

H2 24.320999 6.549000 36.518002

O 16.044001 22.107000 31.337999

H1 15.164000 21.833000 31.674000

H2 16.576000 21.323000 31.584000

O 41.382000 4.707000 25.386000

H1 41.321999 5.646000 25.103001

H2 41.242001 4.787000 26.344999

O 31.754000 8.703000 54.806999

H1 31.798000 7.983000 55.451000

H2 31.242001 8.322000 54.083000

O 11.298000 44.771000 27.944000

H1 11.667000 44.270000 27.187000

H2 11.924000 44.527000 28.650000

O 50.943001 43.104000 17.434000

H1 50.854000 42.514999 18.195999

H2 51.055000 43.972000 17.847000

O 16.670000 31.910999 32.972000

H1 16.591000 31.245001 33.673000

H2 17.632999 31.921000 32.812000

O 27.152000 52.469002 16.482000

H1 27.971001 52.921001 16.766001

H2 26.472000 53.005001 16.945000

O 54.000000 32.307999 58.316002

H1 54.917000 32.006001 58.512001

H2 53.627998 31.500999 57.918999

O 11.921000 7.237000 34.640999

H1 11.442000 7.791000 33.981998

H2 12.494000 6.720000 34.041000

O 18.296000 16.972000 31.533001

H1 18.893000 17.270000 30.837999

H2 18.833000 16.266001 31.962999

O 15.874000 45.571999 16.461000

H1 16.778000 45.903999 16.361000

H2 15.591000 45.506001 15.520000

O 63.226002 49.659000 28.606001

H1 63.355000 49.124001 29.417000

H2 64.133003 49.764000 28.294001

O 19.966000 42.868000 14.531000

H1 19.197001 42.974998 15.108000

H2 19.554001 42.754002 13.662000

O 38.563000 41.268002 57.966000

H1 38.091000 41.294998 57.120998

H2 39.049999 40.431999 57.910000

O 26.049999 39.747002 68.223000

H1 25.635000 40.415001 67.648003

H2 26.386999 39.111000 67.550003

O 52.668999 57.450001 20.277000

H1 52.369999 57.257999 19.372000

H2 52.176998 58.256001 20.490999

O 55.598000 58.821999 33.799999

H1 56.526001 58.860001 34.115002

H2 55.710999 58.425999 32.923000

O 34.287998 28.583000 19.684999

H1 33.362000 28.292000 19.732000

H2 34.363998 28.886999 18.768999

O 42.226002 45.212002 17.215000

H1 41.518002 44.654999 17.629000

H2 41.930000 46.094002 17.516001

O 51.633999 14.760000 25.782000

H1 52.252998 14.053000 25.528999

H2 52.186001 15.554000 25.695000

O 6.184000 23.152000 34.801998

H1 5.312000 22.738001 34.874001

H2 6.094000 23.677000 33.986000

O 19.420000 25.573000 23.221001

H1 19.370001 26.287001 22.556000

H2 18.771999 24.938000 22.864000

O 20.424000 54.338001 29.070000

H1 20.017000 55.212002 29.129999

H2 20.362000 54.008999 29.980000

O 29.400999 54.856998 54.439999

H1 29.655001 54.779999 53.494999

H2 28.981001 55.736000 54.439999

O 8.423000 41.782001 43.582001

H1 7.585000 42.188999 43.311001

H2 8.492000 42.049000 44.513000

O 54.995998 41.695999 10.456000

H1 55.558998 41.223999 11.097000

H2 54.111000 41.358002 10.688000

O 26.867001 22.718000 64.253998

H1 27.343000 23.084000 65.025002

H2 26.681000 23.540001 63.750000

O 45.181000 28.135000 68.094002

H1 44.980000 28.466999 67.195000

H2 45.648998 28.891001 68.477997

O 26.549999 57.612999 36.027000

H1 26.365999 57.057999 35.233002

H2 25.743000 58.165001 36.048000

O 41.526001 55.736000 42.442001

H1 40.945000 55.257999 41.834000

H2 41.556999 55.144001 43.209000

O 41.915001 55.248001 45.890999

H1 41.375000 54.882999 46.620998

H2 42.098000 54.451000 45.368000

O 12.564000 56.604000 37.230999

H1 13.413000 57.084999 37.139999

H2 12.150000 57.087002 37.962002

O 40.233002 43.186001 49.719002

H1 40.296001 44.160000 49.703999

H2 39.258999 43.074001 49.648998

O 38.893002 15.476000 43.828999

H1 38.853001 15.660000 44.780998

H2 39.330002 16.275000 43.480999

O 35.459000 29.905001 13.367000

H1 34.660999 30.454000 13.418000

H2 35.125000 29.033001 13.645000

O 17.073000 27.775000 23.771000

H1 16.414000 28.014999 23.098000

H2 16.721001 26.919001 24.090000

O 52.626999 50.660000 31.305000

H1 52.018002 51.250000 31.789000

H2 53.467999 50.833000 31.788000

O 18.698999 37.243999 12.398000

H1 17.781000 37.514000 12.239000

H2 19.045000 37.997002 12.913000

O 47.150002 41.476002 49.172001

H1 46.941002 41.737999 48.249001

H2 47.397999 40.540001 49.034000

O 20.549000 62.081001 50.152000

H1 20.080999 62.224998 49.311001

H2 21.476999 62.220001 49.886002

O 35.807999 30.437000 10.525000

H1 35.061001 29.914000 10.160000

H2 35.430000 30.768999 11.351000

O 16.688999 17.726999 45.682999

H1 16.468000 18.667000 45.847000

H2 17.398001 17.577999 46.330002

O 36.480000 55.492001 15.224000

H1 35.536999 55.643002 15.034000

H2 36.486000 55.452000 16.194000

O 11.611000 40.050999 63.438999

H1 11.233000 40.910000 63.699001

H2 11.631000 40.126999 62.469002

O 57.275002 61.255001 26.096001

H1 57.962002 61.152000 26.777000

H2 56.508999 60.839001 26.535999

O 24.416000 55.069000 46.886002

H1 25.025000 54.652000 46.251999

H2 25.059000 55.509998 47.485001

O 33.849998 63.396000 49.616001

H1 33.375000 62.967999 48.889000

H2 34.146000 64.225998 49.208000

O 41.337002 13.426000 22.257999

H1 41.655998 14.354000 22.316999

H2 40.896999 13.442000 21.386999

O 54.983002 15.359000 39.779999

H1 54.888000 14.974000 40.672001

H2 54.757999 16.292999 39.942001

O 62.209000 20.062000 34.355000

H1 62.136002 19.965000 33.390999

H2 61.606998 20.815001 34.518002

O 35.698002 59.234001 55.169998

H1 35.526001 59.471001 54.250000

H2 35.296001 59.972000 55.653999

O 63.116001 38.512001 22.767000

H1 63.756001 37.980999 22.268000

H2 62.266998 38.146999 22.464001

O 40.693001 61.118000 18.907000

H1 39.910000 60.639000 18.584000

H2 40.549999 61.124001 19.863001

O 35.294998 36.958000 9.163000

H1 34.768002 36.598000 8.431000

H2 34.977001 37.877998 9.198000

O 15.878000 6.320000 31.051001

H1 16.667000 5.789000 31.291000

H2 15.593000 5.881000 30.233999

O 30.702000 41.430000 61.511002

H1 30.705000 41.405998 60.540001

H2 29.773001 41.214001 61.710999

O 55.365002 26.089001 18.216999

H1 55.686001 26.063999 17.295000

H2 55.015999 25.188999 18.330000

O 51.389000 26.847000 17.870001

H1 51.905998 26.155001 17.431000

H2 51.400002 26.546000 18.798000

O 56.598999 18.233000 42.827000

H1 55.658001 18.124001 42.606998

H2 56.571999 18.923000 43.507999

O 56.971001 55.542999 35.896000

H1 57.782001 55.904999 35.491001

H2 56.426998 56.335999 36.002998

O 43.120998 60.335999 38.299000

H1 42.477001 60.550999 38.994999

H2 43.412998 61.231998 38.022999

O 50.566002 12.558000 23.448000

H1 51.070000 12.900000 24.202000

H2 49.798000 13.161000 23.436001

O 37.404999 32.283001 13.739000

H1 37.720001 32.671001 12.906000

H2 36.803001 31.587000 13.418000

O 17.555000 28.292999 46.597000

H1 17.532000 29.120001 46.081001

H2 17.855000 28.605000 47.465000

O 18.000000 55.573002 23.707001

H1 17.735001 55.397999 24.622999

H2 18.253000 54.675999 23.406000

O 31.993000 22.527000 60.505001

H1 31.253000 21.986000 60.820999

H2 31.712000 22.752001 59.605000

O 27.337000 39.106998 10.388000

H1 28.021000 38.502998 10.066000

H2 26.948000 38.618999 11.126000

O 19.789000 20.417999 62.875999

H1 20.072001 21.320000 62.667999

H2 20.530001 19.892000 62.523998

O 18.783001 18.406000 54.831001

H1 18.986000 19.348000 55.012001

H2 19.274000 17.966999 55.548000

O 7.014000 38.533001 24.723000

H1 6.408000 39.266998 24.497999

H2 6.396000 37.820000 24.933001

O 27.759001 4.608000 43.161999

H1 27.547001 3.983000 42.438999

H2 27.929001 5.424000 42.666000

O 19.242001 12.754000 58.556999

H1 19.332001 12.533000 57.609001

H2 19.980000 13.390000 58.666000

O 22.629999 23.146999 20.972000

H1 22.107000 22.399000 21.285000

H2 22.820999 22.906000 20.055000

O 34.931000 35.313000 66.286003

H1 35.716000 35.773998 65.947998

H2 34.268002 35.523998 65.598999

O 47.276001 45.198002 7.305000

H1 46.525002 44.967999 7.893000

H2 47.872002 44.445000 7.456000

O 28.554001 40.271999 12.534000

H1 28.813000 39.341999 12.680000

H2 27.988001 40.180000 11.742000

O 44.651001 5.674000 40.187000

H1 44.346001 6.080000 39.347000

H2 44.256001 6.290000 40.841000

O 25.481001 33.907001 65.680000

H1 24.563999 33.825001 66.007004

H2 25.775999 32.986000 65.700996

O 28.739000 63.122002 33.407001

H1 28.861000 62.198002 33.162998

H2 27.844000 63.125999 33.785000

O 28.764999 56.050999 24.634001

H1 27.809000 55.946999 24.447001

H2 28.754999 56.351002 25.556999

O 44.219002 4.620000 32.835999

H1 43.703999 5.274000 33.342999

H2 43.548000 4.387000 32.139999

O 19.624001 59.395000 43.026001

H1 18.923000 59.962002 43.394001

H2 19.346001 58.514000 43.324001

O 34.186001 6.484000 38.712002

H1 33.657001 6.688000 39.506001

H2 34.689999 5.710000 39.007000

O 59.942001 48.730999 23.966000

H1 59.709999 49.546001 24.468000

H2 60.355000 49.115002 23.174999

O 25.961000 20.179001 14.091000

H1 26.188000 19.315001 14.466000

H2 26.655001 20.304001 13.421000

O 46.819000 30.384001 46.432999

H1 47.641998 30.799999 46.757999

H2 46.146000 31.010000 46.779999

O 14.577000 35.021999 41.287998

H1 13.749000 34.646000 40.938000

H2 15.222000 34.325001 41.035000

O 36.750999 33.081001 66.181000

H1 36.230000 32.349998 65.820000

H2 36.120998 33.821999 66.132004

O 41.644001 29.539000 15.848000

H1 41.418999 29.622000 16.802000

H2 40.868999 29.983999 15.448000

O 17.149000 31.275999 51.158001

H1 16.962000 32.051998 51.751999

H2 17.924999 31.621000 50.682999

O 60.708000 51.091999 37.682999

H1 61.431000 51.354000 38.296001

H2 61.164001 51.185001 36.826000

O 23.382000 27.212000 29.284000

H1 23.298000 26.855000 30.179001

H2 24.202000 27.712999 29.323000

O 33.792999 59.143002 27.931999

H1 34.474998 58.995998 28.624001

H2 34.355999 59.394001 27.181999

O 13.137000 36.353001 46.235001

H1 13.100000 37.328999 46.154999

H2 13.729000 36.126999 45.499001

O 35.497002 20.517000 41.122002

H1 34.668999 21.025999 41.020000

H2 35.269001 19.679001 40.705002

O 49.469002 48.238998 28.322001

H1 48.539001 48.551998 28.295000

H2 49.792999 48.779999 29.089001

O 11.878000 54.527000 32.042999

H1 10.975000 54.287998 31.783001

H2 12.390000 54.306000 31.253000

O 14.627000 3.334000 35.101002

H1 15.493000 2.922000 34.941002

H2 14.238000 3.340000 34.213001

O 37.276001 7.887000 38.973999

H1 36.633999 7.168000 38.882999

H2 38.077000 7.425000 39.249001

O 26.055000 12.091000 31.676001

H1 26.052999 12.858000 31.082001

H2 25.118000 11.865000 31.724001

O 38.904999 17.231001 56.638000

H1 39.090000 16.405001 57.118999

H2 38.268002 17.667999 57.213001

O 19.580999 62.363998 47.553001

H1 19.792000 63.314999 47.414001

H2 20.077000 61.953999 46.830002

O 9.351000 41.459999 21.906000

H1 8.879000 41.818001 21.136999

H2 10.274000 41.474998 21.608999

O 52.133999 23.149000 58.734001

H1 51.730000 23.882000 59.235001

H2 51.898998 22.379000 59.264999

O 48.279999 52.698002 9.587000

H1 47.641998 52.493999 10.308000

H2 47.818001 52.320999 8.818000

O 11.392000 32.054001 41.738998

H1 10.580000 32.539001 41.487999

H2 12.051000 32.487999 41.175999

O 5.791000 39.699001 29.492001

H1 5.261000 40.505001 29.681000

H2 6.700000 40.082001 29.532000

O 44.926998 47.110001 53.548000

H1 45.547001 46.777000 54.240002

H2 44.410999 46.296001 53.372002

O 45.534000 39.361000 63.918999

H1 45.558998 39.891998 63.108002

H2 45.143002 38.527000 63.584999

O 49.383999 41.402000 45.597000

H1 49.224998 42.180000 46.175999

H2 48.453999 41.152000 45.393002

O 14.744000 29.900999 50.153000

H1 14.803000 30.457001 49.365002

H2 15.224000 30.412001 50.814999

O 4.184000 47.889000 32.620998

H1 4.891000 47.208000 32.551998

H2 4.503000 48.412998 33.375999

O 23.344000 0.870000 29.702999

H1 23.798000 1.732000 29.695999

H2 22.878000 0.895000 30.552999

O 37.820999 17.773001 60.577000

H1 37.959999 16.826000 60.757000

H2 37.011002 17.756001 60.040001

O 12.192000 32.827000 62.544998

H1 12.585000 32.421001 63.335999

H2 12.938000 33.384998 62.226002

O 41.337002 22.721001 60.411999

H1 41.841999 22.125000 59.844002

H2 41.898998 23.511999 60.433998

O 48.535000 48.696999 36.841999

H1 49.418999 48.382999 37.133999

H2 48.785999 49.563000 36.460999

O 14.344000 37.101002 51.682999

H1 13.935000 36.285999 52.027000

H2 14.937000 37.352001 52.410000

O 28.906000 4.429000 49.432999

H1 27.997000 4.358000 49.768002

H2 29.382999 4.790000 50.188999

O 15.982000 38.891998 67.241997

H1 16.636999 39.339001 67.807999

H2 16.290001 39.152000 66.355003

O 23.743000 3.319000 39.341000

H1 24.000999 2.390000 39.543999

H2 23.167000 3.182000 38.571999

O 49.127998 9.725000 28.886999

H1 48.324001 10.289000 28.931999

H2 48.723999 8.903000 28.513000

O 22.759001 24.420000 12.807000

H1 23.716999 24.228001 12.730000

H2 22.385000 23.688000 12.273000

O 40.841999 49.088001 13.280000

H1 41.338001 49.918999 13.162000

H2 40.896999 48.946999 14.232000

O 22.288000 39.735001 19.466000

H1 21.677999 40.445000 19.209999

H2 23.157000 40.160000 19.309000

O 13.437000 60.455002 33.226002

H1 13.435000 59.504002 33.036999

H2 14.087000 60.793999 32.592999

O 52.375999 50.532001 41.917999

H1 52.477001 49.634998 42.269001

H2 51.991001 50.370998 41.043999

O 56.094002 20.849001 21.034000

H1 56.325001 21.448999 21.771999

H2 56.827999 20.197001 21.105000

O 66.787003 28.753000 36.151001

H1 66.028999 28.153999 35.990002

H2 66.391998 29.618999 35.932999

O 52.949001 41.611000 32.905998

H1 53.116001 42.515999 32.569000

H2 53.865002 41.305000 33.067001

O 16.341999 25.945999 46.340000

H1 15.429000 25.973000 46.703999

H2 16.632999 26.865999 46.537998

O 47.227001 6.381000 34.263000

H1 46.620998 5.765000 33.852001

H2 47.536999 5.903000 35.047001

O 31.881001 31.534000 9.445000

H1 32.013000 30.750000 8.876000

H2 32.652000 32.076000 9.212000

O 33.919998 57.331001 17.371000

H1 34.393002 58.012001 17.891001

H2 33.819000 57.777000 16.518000

O 38.042999 63.933998 46.750999

H1 38.277000 64.075996 47.674999

H2 38.758999 63.348999 46.438000

O 26.231001 14.703000 22.320999

H1 25.483000 15.268000 22.577999

H2 25.804001 14.029000 21.778999

O 40.905998 48.626999 52.436001

H1 39.993000 48.527000 52.145000

H2 40.981998 47.955002 53.134998

O 23.084000 39.321999 9.989000

H1 23.613001 38.779999 9.355000

H2 22.243000 38.838001 9.977000

O 7.347000 38.922001 45.792000

H1 6.846000 38.115002 45.549999

H2 7.778000 38.653000 46.608002

O 38.577000 59.752998 47.055000

H1 38.657001 58.793999 47.306999

H2 39.168999 60.137001 47.738998

O 56.533001 36.081001 21.896999

H1 57.108002 36.248001 21.136000

H2 56.155998 35.210999 21.676001

O 40.778000 16.466000 49.428001

H1 41.146000 16.836000 48.605000

H2 39.828999 16.667999 49.331001

O 15.835000 38.889000 17.941999

H1 16.056000 38.296001 17.215000

H2 16.544001 39.546001 17.909000

O 53.381001 29.089001 53.842999

H1 54.126999 29.549000 54.285000

H2 53.416000 29.462999 52.956001

O 43.007999 58.441002 14.729000

H1 43.490002 57.646999 15.033000

H2 43.594002 58.745998 14.007000

O 40.692001 9.737000 20.813999

H1 40.203999 10.178000 21.549999

H2 39.965000 9.193000 20.437000

O 53.320000 51.660000 14.191000

H1 54.212002 52.028999 14.255000

H2 52.852001 52.129002 14.903000

O 29.462000 62.991001 22.669001

H1 29.235001 62.062000 22.481001

H2 29.250999 63.069000 23.608000

O 35.215000 14.585000 48.269001

H1 35.293999 15.236000 48.988998

H2 35.991001 14.010000 48.459999

O 13.991000 14.248000 38.603001

H1 14.876000 14.248000 39.007999

H2 13.717000 13.322000 38.755001

O 12.203000 18.481001 42.462002

H1 13.088000 18.816000 42.714001

H2 12.392000 17.547001 42.297001

O 4.171000 25.648001 43.500999

H1 3.957000 26.197001 42.728001

H2 3.316000 25.190001 43.652000

O 49.924000 49.863998 22.083000

H1 49.445999 50.662998 22.341000

H2 50.771999 49.970001 22.541000

O 23.021999 36.771000 18.709999

H1 23.391001 37.495998 19.242001

H2 22.202000 37.161999 18.379000

O 29.239000 13.565000 47.967999

H1 29.403000 13.971000 47.104000

H2 29.934999 12.891000 48.018002

O 55.806999 22.228001 35.846001

H1 55.969002 22.021000 34.907001

H2 54.860001 22.020000 35.932999

O 33.951000 12.005000 21.562000

H1 33.015999 11.806000 21.391001

H2 33.936001 12.976000 21.643999

O 13.775000 26.149000 35.490002

H1 14.475000 26.783001 35.272999

H2 14.152000 25.657000 36.231998

O 9.494000 15.981000 42.900002

H1 10.123000 16.054001 43.639000

H2 9.479000 16.882000 42.550999

O 30.490999 3.601000 30.488001

H1 30.490999 3.430000 29.535999

H2 29.601000 3.291000 30.750999

O 56.189999 35.507999 17.311001

H1 55.283001 35.141998 17.393999

H2 56.088001 36.342999 17.815001

O 10.235000 52.610001 35.458000

H1 10.509000 52.223999 36.313999

H2 9.973000 53.508999 35.735001

O 26.743999 28.908001 14.322000

H1 26.277000 28.407000 13.641000

H2 27.628000 28.497000 14.313000

O 47.397999 58.223999 34.567001

H1 47.875000 57.736000 35.262001

H2 47.534000 57.646999 33.793999

O 13.260000 10.854000 45.112000

H1 13.535000 11.772000 45.319000

H2 12.423000 10.991000 44.660000

O 36.865002 64.378998 18.565001

H1 37.734001 64.500000 18.124001

H2 36.361000 65.114998 18.152000

O 56.178001 45.841000 21.122999

H1 56.776001 45.473999 21.811001

H2 55.408001 46.090000 21.683001

O 23.982000 58.222000 52.909000

H1 24.476000 58.834999 53.479000

H2 24.667999 57.594002 52.643002

O 28.558001 62.794998 39.902000

H1 28.687000 63.027000 40.838001

H2 28.316000 61.855000 39.964001

O 59.134998 56.161999 38.250999

H1 59.542999 57.000999 38.004002

H2 59.799999 55.512001 37.973999

O 49.249001 26.962000 51.303001

H1 48.405998 27.209999 51.706001

H2 49.138000 25.997999 51.188999

O 41.987000 25.003000 21.037001

H1 42.784000 25.474001 21.351999

H2 41.282001 25.504000 21.492001

O 52.597000 21.742001 51.206001

H1 52.799000 22.257999 52.006001

H2 52.771999 20.833000 51.508999

O 37.555000 20.487000 28.129999

H1 37.137001 20.146000 27.316000

H2 36.910999 21.167999 28.398001

O 44.459999 34.382999 14.795000

H1 45.035999 35.168999 14.916000

H2 44.682999 34.132000 13.882000

O 7.148000 48.688999 30.365000

H1 7.539000 48.209999 31.132000

H2 7.930000 49.209000 30.079000

O 28.107000 19.804001 62.338001

H1 28.990000 19.750000 61.938000

H2 28.143999 19.136000 63.034000

O 31.292000 59.842999 39.518002

H1 30.681999 59.355999 40.089001

H2 30.808001 59.868000 38.671001

O 49.414001 15.124000 23.990999

H1 48.616001 15.413000 24.483000

H2 50.104000 15.273000 24.667000

O 16.367001 31.224001 59.382000

H1 16.447001 32.124001 59.048000

H2 15.417000 31.048000 59.301998

O 46.744999 31.056999 60.506001

H1 46.526001 31.493999 61.349998

H2 47.543999 31.549999 60.240002

O 45.228001 22.812000 65.313004

H1 44.366001 22.715000 64.858002

H2 45.730999 23.315001 64.644997

O 38.540001 41.153999 46.589001

H1 37.761002 41.428001 47.099998

H2 38.897999 40.438999 47.138000

O 32.985001 44.430000 55.771999

H1 33.539001 44.139999 55.020000

H2 32.679001 43.576000 56.120998

O 44.631001 45.151001 12.651000

H1 43.819000 44.958000 12.167000

H2 44.303001 45.695000 13.394000

O 50.798000 55.133999 34.230000

H1 50.905998 56.073002 33.992001

H2 51.141998 55.136002 35.145000

O 26.243000 60.528999 21.582001

H1 26.034000 59.667000 21.193001

H2 27.219000 60.471001 21.674000

O 24.573000 30.695999 63.794998

H1 24.576000 31.079000 64.693001

H2 25.546000 30.614000 63.640999

O 38.042999 61.706001 20.660999

H1 37.123001 61.942001 20.454000

H2 38.515999 62.526001 20.431999

O 28.372000 19.865999 28.167000

H1 27.849001 20.424000 28.752001

H2 29.127001 20.445000 27.945999

O 62.952000 19.556999 29.534000

H1 62.564999 18.990999 28.860001

H2 62.248001 19.641001 30.188000

O 36.435001 42.993999 7.059000

H1 37.304001 42.536999 7.106000

H2 36.425999 43.479000 7.894000

O 9.172000 31.539000 55.249001

H1 10.017000 31.250999 55.639999

H2 8.639000 31.715000 56.042999

O 66.655998 46.373001 27.478001

H1 66.239998 45.939999 28.256001

H2 67.509003 45.909000 27.441000

O 30.799999 10.827000 47.313000

H1 31.405001 10.647000 46.568001

H2 29.996000 10.372000 47.044998

O 17.042999 1.783000 35.117001

H1 17.850000 1.248000 35.250999

H2 16.962000 2.218000 35.987999

O 50.763000 12.770000 41.540001

H1 51.116001 12.057000 40.970001

H2 50.189999 13.250000 40.905998

O 35.053001 52.882000 60.358002

H1 34.671001 53.327000 59.577999

H2 34.900002 51.942001 60.143002

O 44.313999 37.417000 62.391998

H1 44.014000 37.681000 61.493999

H2 43.588001 36.827999 62.662998

O 52.949001 50.054001 17.642000

H1 53.110001 50.842999 17.107000

H2 52.270000 49.590000 17.117001

O 61.132999 26.358999 34.063000

H1 60.980000 26.964001 34.805000

H2 60.244999 26.267000 33.683998

O 42.819000 66.507004 26.944000

H1 42.355000 66.049004 27.665001

H2 42.157001 66.489998 26.237000

O 21.525999 36.039001 11.367000

H1 20.726000 36.210999 11.882000

H2 21.221001 36.176998 10.455000

O 18.399000 42.911999 20.622999

H1 18.159000 43.444000 21.391001

H2 17.541000 42.702000 20.228001

O 51.137001 36.188000 58.824001

H1 51.021000 36.453999 57.897999

H2 50.368000 36.598999 59.255001

O 29.125999 37.827999 52.257000

H1 29.784000 37.905998 51.535000

H2 29.382999 38.570999 52.828999

O 45.823002 16.082001 56.763000

H1 45.839001 16.813000 57.407001

H2 46.612000 16.264000 56.230000

O 36.546001 23.076000 8.956000

H1 37.030998 23.648001 8.326000

H2 36.604000 23.624001 9.771000

O 16.073000 21.837000 50.958000

H1 16.586000 21.009001 51.006001

H2 16.638000 22.372000 50.368999

O 33.389999 41.667000 6.226000

H1 34.224998 41.320000 6.573000

H2 33.018002 42.125999 6.992000

O 20.195999 44.384998 55.355000

H1 19.818001 45.215000 55.724998

H2 20.990999 44.293999 55.917000

O 29.834999 4.023000 16.385000

H1 29.483000 4.929000 16.393999

H2 29.797001 3.812000 15.432000

O 54.196999 54.351002 35.203999

H1 55.096001 54.701000 35.308998

H2 53.658001 55.070000 35.589001

O 49.320999 57.000000 22.330000

H1 49.745998 57.828999 22.599001

H2 48.703999 57.314999 21.645000

O 17.339001 42.773998 17.385000

H1 16.671000 43.466999 17.341000

H2 18.155001 43.279999 17.552999

O 42.026001 5.266000 20.934000

H1 41.243999 4.736000 20.673000

H2 42.742001 4.779000 20.490000

O 52.580002 23.853001 41.098999

H1 52.366001 24.037001 40.160999

H2 53.203999 23.113001 41.007999

O 29.997999 32.358002 68.054001

H1 30.405001 32.935001 68.722000

H2 29.247999 32.886002 67.750999

O 10.775000 3.035000 37.424999

H1 10.085000 3.690000 37.210999

H2 11.579000 3.483000 37.125999

O 57.664001 47.179001 30.035999

H1 57.971001 46.806999 29.186001

H2 58.487999 47.547001 30.395000

O 17.224001 51.826000 37.191002

H1 17.084999 52.661999 37.667999

H2 18.003000 52.056000 36.634998

O 8.738000 27.107000 38.692001

H1 8.429000 27.715000 37.993000

H2 9.639000 26.900999 38.355999

O 13.697000 46.526001 42.983002

H1 13.706000 46.192001 43.896000

H2 14.196000 45.814999 42.522999

O 42.707001 58.922001 47.835999

H1 42.514999 58.382999 47.053001

H2 43.521999 59.375000 47.577999

O 24.979000 19.308001 48.243999

H1 24.559000 19.933001 47.626999

H2 24.545000 19.535000 49.082001

O 49.292000 23.471001 28.674000

H1 49.646000 23.091000 29.487000

H2 49.682999 22.905001 27.985001

O 31.195999 60.327000 30.164000

H1 30.895000 60.205002 29.249001

H2 30.341000 60.349998 30.643999

O 37.428001 43.067001 50.000000

H1 36.952999 42.448002 50.589001

H2 36.681000 43.423000 49.469002

O 7.948000 34.651001 42.007999

H1 8.416000 34.098999 42.680000

H2 8.397000 34.327999 41.195999

O 32.658001 46.974998 15.538000

H1 32.176998 47.449001 16.243000

H2 32.513000 46.049000 15.793000

O 14.494000 28.052000 29.823000

H1 13.867000 28.754000 29.572001

H2 15.178000 28.570000 30.288000

O 48.821999 8.286000 44.111000

H1 49.577999 8.770000 43.694000

H2 48.103001 8.525000 43.486000

O 10.462000 29.226999 17.448000

H1 11.441000 29.165001 17.528000

H2 10.186000 28.555000 18.091000

O 10.642000 39.570999 45.165001

H1 10.262000 38.674999 45.102001

H2 9.876000 40.098000 45.425999

O 21.801001 4.940000 54.195999

H1 21.309000 5.776000 54.284000

H2 22.323999 4.933000 55.009998

O 57.356998 53.824001 24.579000

H1 56.403000 53.831001 24.408001

H2 57.402000 53.622002 25.521999

O 54.988998 41.006001 17.146000

H1 54.963001 41.126999 18.108000

H2 55.013000 41.921001 16.825001

O 55.164001 60.862999 30.434999

H1 55.631001 60.634998 29.606001

H2 55.002998 59.974998 30.802999

O 43.217999 9.381000 39.080002

H1 42.480999 9.427000 39.720001

H2 43.835999 10.031000 39.450001

O 10.963000 27.805000 30.016001

H1 10.689000 28.715000 30.211000

H2 11.752000 27.934000 29.473000

O 43.243999 52.869999 41.112000

H1 43.207001 53.823002 40.875000

H2 43.827999 52.556000 40.373001

O 54.410000 47.202000 22.528999

H1 54.759998 48.042999 22.884001

H2 53.571999 47.498001 22.129000

O 63.529999 24.038000 36.020000

H1 62.706001 24.438000 35.709000

H2 63.786999 23.479000 35.263000

O 35.911999 57.103001 21.802000

H1 35.840000 56.132000 21.782000

H2 35.242001 57.373001 21.166000

O 24.224001 4.427000 34.840000

H1 24.094000 5.325000 35.199001

H2 24.398001 4.621000 33.896000

O 16.940001 23.450001 41.375999

H1 16.146000 23.318001 40.846001

H2 16.583000 23.566000 42.270000

O 58.435001 23.662001 33.185001

H1 57.637001 23.110001 33.185001

H2 58.285000 24.235001 33.956001

O 51.287998 16.850000 40.157001

H1 51.853001 16.211000 39.688999

H2 51.415001 16.573999 41.088001

O 22.733999 45.564999 13.390000

H1 23.549000 45.890999 13.820000

H2 22.605000 44.716000 13.828000

O 18.433001 45.213001 59.446999

H1 17.615999 44.819000 59.105999

H2 18.910999 44.439999 59.779999

O 42.020000 48.276001 41.259998

H1 41.895000 49.203999 41.591000

H2 43.019001 48.269001 41.282001

O 21.320999 14.251000 40.061001

H1 22.083000 14.849000 40.070000

H2 20.593000 14.849000 39.828999

O 13.846000 3.827000 37.653000

H1 14.106000 3.464000 36.777000

H2 14.189000 4.735000 37.574001

O 58.445999 38.806000 42.141998

H1 58.601002 38.168999 41.418999

H2 57.959999 38.266998 42.784000

O 42.207001 54.164001 15.578000

H1 41.991001 54.506001 16.452999

H2 42.897999 54.761002 15.270000

O 57.550999 21.037001 38.178001

H1 58.044998 21.632999 37.591999

H2 57.320000 21.646000 38.911999

O 35.949001 40.313999 6.591000

H1 36.875000 40.047001 6.695000

H2 36.008999 41.278999 6.726000

O 21.181999 58.620998 53.105000

H1 20.940001 57.729000 52.791000

H2 22.141001 58.516998 53.254002

O 25.749001 38.764999 64.045998

H1 26.112000 38.912998 64.933998

H2 25.983999 37.819000 63.907001

O 50.852001 18.268999 17.636999

H1 50.953999 17.834999 16.764000

H2 50.042000 17.851999 17.962000

O 6.966000 30.202999 30.167999

H1 7.595000 29.480000 30.037001

H2 7.186000 30.815001 29.458000

O 62.900002 49.273998 33.345001

H1 63.629002 48.748001 32.987000

H2 62.638000 48.755001 34.126999

O 27.344000 32.841000 70.740997

H1 26.566000 32.866001 70.150002

H2 27.892000 33.554001 70.342003

O 42.234001 12.893000 29.056999

H1 42.818001 12.206000 29.407000

H2 42.705002 13.159000 28.243000

O 25.642000 31.797001 11.934000

H1 25.016001 31.313999 11.377000

H2 26.424000 31.867001 11.354000

O 45.228001 44.967999 8.982000

H1 44.335999 44.578999 9.042000

H2 45.613998 44.720001 9.834000

O 16.065001 22.181000 14.994000

H1 16.381001 22.843000 15.630000

H2 16.718000 22.271999 14.277000

O 51.820999 52.926998 23.410999

H1 51.500999 53.075001 24.318001

H2 51.798000 51.950001 23.362000

O 45.571999 32.459999 16.421000

H1 45.041000 33.132999 15.952000

H2 46.123001 32.124001 15.675000

O 31.278999 3.478000 25.330999

H1 30.568001 4.137000 25.357000

H2 32.004002 4.003000 24.916000

O 24.930000 43.773998 8.728000

H1 24.451000 44.451000 8.187000

H2 25.112000 44.290001 9.529000

O 53.504002 25.100000 57.094002

H1 53.054001 24.444000 57.657001

H2 53.890999 25.693001 57.771999

O 23.129999 22.035999 24.908001

H1 22.334999 22.566999 24.681000

H2 23.226000 21.481001 24.120001

O 41.449001 32.255001 61.112000

H1 41.505001 33.222000 61.178001

H2 40.556000 32.088001 61.476002

O 29.197001 42.987000 69.803001

H1 28.642000 42.196999 69.706001

H2 30.023001 42.724998 69.384003

O 24.627001 24.384001 24.785000

H1 24.166000 23.524000 24.766001

H2 25.426001 24.173000 24.252001

O 40.469002 13.650000 49.198002

H1 39.820000 13.468000 49.910999

H2 40.542999 14.618000 49.256001

O 5.551000 19.990000 37.354000

H1 6.027000 19.146000 37.351002

H2 5.034000 19.917999 38.180000

O 16.636000 28.507000 18.207001

H1 15.878000 28.684000 18.797001

H2 16.448000 29.107000 17.468000

O 4.143000 21.120001 35.402000

H1 4.638000 21.037001 36.238998

H2 3.239000 21.273001 35.715000

O 32.081001 6.780000 32.992001

H1 32.708000 6.383000 32.359001

H2 31.229000 6.461000 32.636002

O 13.062000 37.606998 36.706001

H1 12.567000 36.832001 36.402000

H2 13.469000 37.268002 37.530998

O 20.045000 39.264999 13.730000

H1 20.962999 39.542000 13.913000

H2 19.674000 39.270000 14.638000

O 54.001999 23.995001 47.986000

H1 54.389999 23.148001 48.301998

H2 54.161999 23.917999 47.027000

O 13.752000 20.620001 50.047001

H1 14.418000 21.156000 50.514000

H2 14.229000 20.389000 49.231998

O 38.894001 56.294998 28.167000

H1 38.118999 55.778999 28.455999

H2 38.825001 56.181000 27.188999

O 22.292000 60.665001 25.572001

H1 22.996000 60.112999 25.190001

H2 22.260000 60.354000 26.489000

O 31.111000 28.631001 65.646004

H1 30.577000 28.288000 64.896004

H2 31.628000 27.816999 65.870003

O 50.084999 66.587997 37.888000

H1 49.196999 66.353996 38.199001

H2 49.998001 66.509003 36.924999

O 31.351999 38.773998 6.405000

H1 31.424000 38.500999 5.477000

H2 30.891001 39.622002 6.340000

O 28.896999 32.078999 64.969002

H1 28.850000 33.033001 64.748001

H2 29.767000 31.837999 64.614998

O 21.056000 43.665001 62.095001

H1 20.344999 44.117001 62.589001

H2 20.583000 43.345001 61.308998

O 15.886000 24.656000 33.923000

H1 14.936000 24.666000 33.741001

H2 16.051001 25.590000 34.167999

O 23.408001 48.283001 13.508000

H1 22.754999 47.639999 13.804000

H2 24.160999 48.102001 14.117000

O 44.007000 10.084000 34.875999

H1 43.924000 9.444000 34.141998

H2 43.118999 10.013000 35.285000

O 52.452000 32.379002 45.340000

H1 53.387001 32.599998 45.537998

H2 52.007000 32.806000 46.105999

O 54.136002 28.900999 18.030001

H1 54.775002 28.976000 17.295000

H2 54.187000 27.957001 18.239000

O 11.870000 28.423000 44.126999

H1 12.149000 28.837999 43.287998

H2 10.896000 28.395000 43.990002

O 4.498000 25.799000 29.055000

H1 4.708000 26.434000 29.771999

H2 3.707000 26.209000 28.665001

O 44.998001 12.328000 46.141998

H1 45.139000 12.352000 45.179001

H2 44.097000 12.685000 46.219002

O 34.112999 38.313999 66.064003

H1 34.203999 38.999001 65.373001

H2 33.556000 37.658001 65.601997

O 19.517000 24.525000 12.279000

H1 20.332001 24.870001 12.670000

H2 18.941999 25.315001 12.288000

O 35.860001 28.757000 17.226000

H1 36.299999 29.566999 17.541000

H2 36.348999 28.065001 17.690001

O 41.403000 26.629000 65.350998

H1 41.499001 27.607000 65.400002

H2 41.637001 26.478001 64.417999

O 62.694000 25.334000 21.329000

H1 63.243000 25.415001 20.535999

H2 61.831001 25.667999 21.003000

O 13.537000 13.268000 49.056999

H1 12.925000 12.529000 49.248001

H2 13.804000 13.055000 48.141998

O 51.514000 33.869999 40.236000

H1 50.566002 33.797001 40.001999

H2 51.569000 33.255001 40.990002

O 49.491001 30.419001 14.307000

H1 50.422001 30.705999 14.402000

H2 49.594002 29.575001 13.842000

O 41.886002 10.324000 36.529999

H1 40.959999 10.246000 36.803001

H2 42.365002 10.055000 37.328999

O 47.067001 51.631001 21.313999

H1 46.972000 50.905998 21.954000

H2 46.422001 52.276001 21.636999

O 21.347000 63.994999 36.758999

H1 21.176001 64.790001 37.303001

H2 22.037001 63.544998 37.273998

O 46.188999 57.430000 21.319000

H1 45.556999 58.148998 21.110001

H2 47.039001 57.875999 21.163000

O 33.620998 58.542000 40.312000

H1 33.077999 59.317001 40.070000

H2 33.502998 57.977001 39.527000

O 58.213001 33.263000 16.987000

H1 57.841999 33.970001 17.538000

H2 57.444000 33.042999 16.417000

O 18.652000 11.892000 53.012001

H1 17.693001 11.958000 52.849998

H2 18.931999 11.305000 52.289001

O 27.680000 21.051001 12.151000

H1 28.365000 20.615999 12.696000

H2 28.165001 21.827999 11.823000

O 45.057999 42.094002 50.674000

H1 44.348000 42.118000 50.000999

H2 45.853001 42.020000 50.105999

O 37.277000 41.676998 37.910999

H1 36.431999 42.084000 38.208000

H2 37.167000 41.316002 37.008999

O 62.125999 32.987000 22.778999

H1 62.806999 33.118999 22.112000

H2 61.837002 32.071999 22.617001

O 36.237000 4.554000 15.782000

H1 36.632000 5.064000 16.497000

H2 35.688000 3.910000 16.266001

O 22.905001 64.662003 46.716000

H1 23.648001 64.712997 47.345001

H2 22.895000 63.717999 46.495998

O 38.712002 21.243999 61.007000

H1 39.280998 21.975000 60.716000

H2 39.367001 20.549999 61.205002

O 51.756001 32.598999 59.904999

H1 52.571999 32.375000 59.419998

H2 52.063000 33.332001 60.470001

O 56.050999 23.674000 30.625000

H1 55.320999 23.990000 30.063999

H2 55.598999 23.517000 31.468000

O 53.841000 60.271000 38.550999

H1 53.540001 60.650002 37.708000

H2 53.423000 60.896000 39.187000

O 52.734001 20.785000 48.377998

H1 53.526001 21.315001 48.146999

H2 52.775002 20.808001 49.348999

O 20.673000 13.678000 54.299999

H1 20.481001 14.134000 53.466000

H2 21.247999 14.324000 54.750999

O 21.587000 60.570999 33.449001

H1 20.891001 61.119999 33.852001

H2 21.094999 59.736000 33.286999

O 8.817000 30.202999 26.825001

H1 8.607000 29.627001 26.077000

H2 9.099000 29.573999 27.503000

O 29.274000 47.655998 67.181000

H1 29.915001 48.158001 67.723000

H2 29.580000 46.743999 67.325996

O 12.377000 58.679001 46.733002

H1 12.255000 58.523998 45.779999

H2 13.078000 58.036999 46.942001

O 35.807999 47.943001 65.575996

H1 34.845001 48.005001 65.432999

H2 35.851002 47.234001 66.244003

O 18.165001 29.181999 53.874001

H1 18.849001 28.598000 53.471001

H2 17.872999 29.688999 53.106998

O 10.994000 27.631001 20.171000

H1 11.089000 26.701000 20.424999

H2 10.023000 27.730000 20.124001

O 54.210999 19.062000 31.091000

H1 54.130001 19.856001 31.660999

H2 53.272999 18.827999 30.969000

O 56.223000 46.014999 33.550999

H1 56.410000 46.889000 33.938999

H2 55.902000 45.521999 34.323002

O 29.354000 37.519001 12.784000

H1 29.224001 36.597000 12.503000

H2 29.673000 37.417999 13.687000

O 52.312000 40.398998 16.620001

H1 51.555000 40.686001 17.160000

H2 53.000000 41.028000 16.891001

O 43.720001 22.466999 56.646000

H1 43.321999 23.228001 57.111000

H2 44.661999 22.702000 56.674999

O 29.172001 8.697000 42.464001

H1 29.327000 9.497000 41.931000

H2 29.072001 8.016000 41.763000

O 49.839001 39.236000 14.834000

H1 50.462002 39.685001 15.426000

H2 49.991001 38.299999 15.070000

O 6.863000 42.290001 47.408001

H1 6.554000 42.768002 48.193001

H2 6.703000 41.363998 47.657001

O 58.778999 27.945999 18.679001

H1 58.514999 28.709000 18.131001

H2 59.043999 27.297001 18.004999

O 37.275002 39.580002 62.627998

H1 37.452999 40.523998 62.856998

H2 37.764000 39.521999 61.778000

O 51.129002 41.009998 50.957001

H1 51.210999 41.930000 51.251999

H2 50.507999 40.643002 51.608002

O 26.476000 5.167000 45.972000

H1 26.945999 5.066000 45.125000

H2 26.676001 4.316000 46.404999

O 12.738000 37.758999 63.952000

H1 12.370000 38.678001 63.903000

H2 12.398000 37.403999 63.104000

O 26.836000 60.606998 36.139999

H1 27.186001 59.806999 35.723999

H2 25.986000 60.303001 36.492001

O 24.040001 64.643997 29.443001

H1 23.763000 64.074997 30.181999

H2 23.399000 65.372002 29.504000

O 22.336000 52.643002 43.695000

H1 22.166000 51.688000 43.637001

H2 22.323000 52.779999 44.665001

O 23.958000 22.895000 17.113001

H1 24.597000 22.226000 17.412001

H2 23.907000 22.686001 16.153999

O 27.093000 7.801000 53.143002

H1 27.607000 8.589000 53.395000

H2 27.107000 7.276000 53.952000

O 40.331001 38.328999 24.796000

H1 40.067001 37.686001 25.493000

H2 39.827000 39.118000 25.115999

O 17.268000 49.070999 49.820999

H1 17.517000 49.751999 50.451000

H2 17.613001 49.428001 48.979000

O 23.132999 7.719000 49.002998

H1 22.618999 6.918000 48.841000

H2 24.009001 7.361000 49.230000

O 34.773998 43.013000 54.235001

H1 35.703999 43.306999 54.265999

H2 34.875999 42.042999 54.339001

O 3.876000 40.589001 36.900002

H1 4.378000 40.837002 37.693001

H2 3.208000 39.988998 37.264999

O 24.888000 0.845000 39.625999

H1 24.607000 -0.087000 39.527000

H2 25.761999 0.831000 39.203999

O 37.509998 49.431999 13.023000

H1 37.334999 48.685001 12.432000

H2 38.271000 49.855999 12.592000

O 28.285000 32.726002 16.608999

H1 29.167999 33.035000 16.886000

H2 27.711000 33.118999 17.281000

O 11.902000 30.159000 55.842999

H1 11.850000 29.254000 56.208000

H2 12.343000 30.009001 54.993999

O 64.415001 33.972000 34.292999

H1 65.206001 33.660999 33.827000

H2 64.809998 34.451000 35.044998

O 26.458000 21.798000 61.636002

H1 26.968000 20.987000 61.862999

H2 26.379000 22.209999 62.514000

O 9.719000 56.110001 33.570000

H1 9.989000 55.585999 34.341999

H2 9.169000 55.470001 33.080002

O 16.100000 2.961000 30.379000

H1 16.902000 2.747000 29.864000

H2 15.504000 3.275000 29.672001

O 46.943001 59.201000 18.131001

H1 46.466999 58.361000 18.086000

H2 46.324001 59.779999 18.598000

O 31.978001 57.682999 58.736000

H1 32.342999 58.525002 59.056000

H2 31.475000 57.966000 57.958000

O 55.581001 22.100000 45.410999

H1 56.463001 22.114000 45.006001

H2 55.006001 22.333000 44.661999

O 53.868000 45.598999 17.177000

H1 53.825001 46.122002 18.010000

H2 53.141998 45.994999 16.667000

O 25.882000 57.472000 25.924000

H1 26.030001 56.532001 25.782000

H2 24.929001 57.499001 26.143999

O 47.638000 18.420000 24.263000

H1 47.469002 17.669001 23.662001

H2 47.521000 19.180000 23.667000

O 9.246000 48.790001 37.369999

H1 8.918000 47.938000 37.695000

H2 8.748000 49.419998 37.937000

O 35.146999 27.552000 14.877000

H1 34.215000 27.695000 15.156000

H2 35.618999 28.025999 15.591000

O 45.143002 36.020000 66.085999

H1 45.220001 35.644001 66.982002

H2 44.330002 36.537998 66.153000

O 5.558000 24.327000 32.356998

H1 4.769000 23.857000 32.043999

H2 6.136000 24.322001 31.590000

O 51.806000 53.854000 19.044001

H1 51.342999 54.146999 19.865000

H2 52.720001 54.138000 19.254999

O 29.188999 0.922000 46.818001

H1 29.384001 1.118000 45.893002

H2 28.646000 1.682000 47.084999

O 32.487000 6.864000 26.579000

H1 32.894001 6.200000 25.990999

H2 31.552000 6.815000 26.298000

O 23.791000 58.995998 32.859001

H1 23.412001 58.341999 33.480000

H2 23.229000 59.773998 33.049000

O 34.257999 7.374000 16.438000

H1 35.009998 7.976000 16.382000

H2 34.581001 6.670000 17.017000

O 49.438000 39.310001 59.334999

H1 50.292999 39.756001 59.446999

H2 49.441002 39.110001 58.382000

O 11.756000 18.188999 28.445999

H1 12.393000 18.587000 29.072001

H2 11.311000 19.000999 28.113001

O 36.455002 16.205999 17.818001

H1 36.791000 15.725000 17.030001

H2 35.493000 16.084999 17.715000

O 37.666000 48.777000 61.109001

H1 36.983002 48.398998 60.529999

H2 37.407001 48.409000 61.980000

O 23.325001 54.354000 51.199001

H1 24.007000 55.041000 51.264999

H2 22.823999 54.646000 50.415001

O 24.691999 0.809000 47.091999

H1 24.636000 -0.019000 47.591999

H2 23.865000 0.820000 46.598999

O 9.975000 49.563000 51.681999

H1 9.622000 48.816002 51.158001

H2 10.903000 49.290001 51.799999

O 46.862000 43.805000 63.333000

H1 45.935001 43.791000 63.634998

H2 47.061001 44.752998 63.353001

O 46.875000 18.194000 38.217999

H1 46.786999 18.010000 37.264999

H2 47.448002 18.968000 38.229000

O 21.591999 33.388000 56.915001

H1 22.437000 32.917999 56.794998

H2 21.270000 32.990002 57.750000

O 41.116001 35.910999 10.695000

H1 40.320999 36.101002 11.212000

H2 41.243000 34.963001 10.841000

O 21.514999 17.267000 41.929001

H1 22.315001 16.934000 41.500000

H2 21.393000 18.127001 41.480999

O 4.365000 48.605000 25.558001

H1 4.752000 47.995998 24.899000

H2 5.073000 48.622002 26.232000

O 40.933998 8.708000 28.040001

H1 40.183998 9.265000 27.733000

H2 40.501999 7.843000 28.128000

O 35.462002 18.575001 19.924000

H1 34.681999 18.240000 20.413000

H2 35.813999 17.757999 19.539000

O 39.216000 36.332001 18.216999

H1 39.360001 36.353001 17.247000

H2 38.243000 36.178001 18.233999

O 47.312000 48.422001 43.035999

H1 48.277000 48.368000 42.834999

H2 47.209000 49.394001 43.074001

O 13.407000 36.921001 30.212999

H1 13.165000 36.620998 29.316999

H2 14.372000 36.993999 30.129000

O 57.577000 31.783001 29.174000

H1 56.745998 32.168999 28.851999

H2 57.402000 31.712999 30.129999

O 26.181000 18.641001 19.268000

H1 26.936001 18.162001 18.886999

H2 25.427000 18.122999 18.930000

O 18.809000 33.138000 49.528000

H1 18.478001 32.792000 48.687000

H2 18.082001 33.723999 49.813999

O 64.147003 36.800999 24.693001

H1 63.764000 37.542999 24.186001

H2 63.653000 36.858002 25.525000

O 45.578999 53.353001 15.057000

H1 45.735001 53.283001 16.017000

H2 46.344002 52.901001 14.690000

O 22.238001 27.607000 24.632999

H1 22.171000 27.408001 25.598000

H2 21.299000 27.542999 24.382000

O 32.476002 12.640000 59.046001

H1 32.694000 13.418000 59.583000

H2 33.353001 12.328000 58.772999

O 28.622999 44.736000 65.584999

H1 28.889999 43.896999 65.179001

H2 28.017000 44.432999 66.286003

O 55.848000 57.383999 26.115000

H1 55.852001 56.504002 26.548000

H2 56.694000 57.747002 26.421000

O 27.830999 6.906000 29.431999

H1 26.889000 6.811000 29.702000

H2 27.749001 7.501000 28.667999

O 12.606000 48.914001 52.439999

H1 13.485000 48.787998 52.021999

H2 12.800000 49.639999 53.058998

O 7.317000 36.585999 36.431000

H1 7.940000 37.151001 35.936001

H2 6.865000 37.255001 36.990002

O 50.348000 59.955002 38.021000

H1 50.566002 60.886002 37.848000

H2 49.397999 59.928001 37.847000

O 49.547001 55.244999 38.425999

H1 49.905998 55.915001 37.823002

H2 50.355000 54.903999 38.848000

O 38.438999 53.691002 51.604000

H1 39.008999 53.132999 52.167000

H2 37.581001 53.611000 52.058998

O 63.238998 45.493999 31.624001

H1 63.437000 44.542999 31.594000

H2 62.700001 45.548000 32.446999

O 27.410999 8.740000 49.988998

H1 28.058001 8.669000 49.271000

H2 27.811001 9.457000 50.534000

O 57.472000 29.888000 53.332001

H1 57.686001 29.024000 52.944000

H2 57.532001 30.471001 52.547001

O 23.351000 61.613998 49.866001

H1 24.298000 61.668999 49.605000

H2 23.221001 60.646000 49.860001

O 56.192001 28.823000 15.950000

H1 56.396000 27.874001 15.858000

H2 55.813000 29.025000 15.076000

O 17.458000 48.351002 18.229000

H1 16.676001 48.780998 17.826000

H2 17.636999 47.648998 17.580999

O 17.712000 25.209000 32.091000

H1 17.216000 25.146000 31.257999

H2 17.031000 24.899000 32.733002

O 31.565001 38.731998 10.879000

H1 31.214001 38.492001 11.750000

H2 30.888000 38.344002 10.286000

O 17.056000 47.255001 27.851000

H1 16.511999 47.127998 28.653999

H2 17.191999 48.216000 27.867001

O 38.595001 59.561001 42.205002

H1 38.987999 60.425999 42.424999

H2 37.932999 59.823002 41.535999

O 48.297001 64.960999 27.542999

H1 47.568001 65.601997 27.545000

H2 48.595001 64.987999 26.622999

O 67.212997 32.004002 38.580002

H1 68.164001 31.933001 38.719002

H2 67.025002 31.275999 37.976002

O 16.062000 26.632999 28.153000

H1 15.991000 25.671000 28.312000

H2 15.476000 26.973000 28.865999

O 42.867001 42.189999 63.681999

H1 42.369999 42.429001 62.882999

H2 42.188000 41.749001 64.219002

O 18.224001 23.930000 21.267000

H1 17.768999 23.080000 21.395000

H2 18.882999 23.702999 20.591999

O 21.261999 25.124001 15.139000

H1 21.639000 24.582001 14.422000

H2 22.034000 25.688999 15.361000

O 53.230999 29.402000 49.424000

H1 53.112000 28.834999 50.200001

H2 52.557999 30.087999 49.571999

O 66.401001 28.018000 27.997999

H1 66.970001 28.752001 27.721001

H2 66.621002 27.332001 27.353001

O 55.869999 62.849998 24.459000

H1 56.125000 62.257999 25.198000

H2 55.430000 62.226002 23.860001

O 31.867001 59.429001 51.622002

H1 32.577999 59.952999 52.039001

H2 31.844000 59.812000 50.724998

O 11.408000 15.702000 32.255001

H1 11.740000 16.528999 32.653000

H2 10.890000 16.035999 31.509001

O 21.687000 51.056000 53.717999

H1 21.313999 50.277000 53.273998

H2 20.882999 51.520000 54.012001

O 14.751000 33.066002 38.360001

H1 13.819000 33.187000 38.097000

H2 14.930000 32.162998 38.078999

O 15.362000 44.223999 28.289000

H1 15.402000 45.134998 28.614000

H2 14.476000 43.944000 28.570000

O 44.338001 13.859000 56.776001

H1 44.866001 14.667000 56.588001

H2 44.294998 13.458000 55.889000

O 16.105000 39.438999 14.675000

H1 15.675000 38.650002 15.020000

H2 16.955999 39.449001 15.148000

O 54.659000 21.882999 40.394001

H1 55.504002 22.346001 40.261002

H2 54.901001 20.962000 40.240002

O 46.713001 9.550000 25.740999

H1 46.283001 8.683000 25.712000

H2 47.651001 9.319000 25.667999

O 16.930000 26.167000 20.209999

H1 17.120001 25.364000 20.724001

H2 17.017000 25.832001 19.292000

O 49.681999 12.794000 44.062000

H1 49.972000 12.774000 43.133999

H2 49.397999 11.875000 44.206001

O 41.076000 14.031000 43.374001

H1 40.195999 14.378000 43.657001

H2 41.306000 14.673000 42.683998

O 20.459999 0.784000 47.668999

H1 20.907000 1.626000 47.493000

H2 19.893000 1.003000 48.429001

O 31.281000 30.823000 14.311000

H1 31.627001 30.813000 15.222000

H2 30.319000 30.889000 14.468000

O 40.735001 20.285999 53.011002

H1 41.553001 19.820000 52.790001

H2 40.192001 20.156000 52.217999

O 53.873001 54.687000 22.510000

H1 53.585999 53.868999 22.958000

H2 53.243000 54.743000 21.777000

O 11.762000 43.620998 39.886002

H1 12.605000 43.312000 40.249001

H2 12.009000 44.459999 39.464001

O 42.581001 17.437000 47.584999

H1 43.018002 18.240000 47.912998

H2 42.904999 17.396999 46.666000

O 20.549999 13.009000 28.385000

H1 20.075001 13.158000 29.225000

H2 20.122999 13.659000 27.799999

O 29.468000 27.544001 59.007999

H1 29.056999 27.614000 59.891998

H2 29.548000 26.580999 58.910000

O 37.580002 48.582001 56.296001

H1 37.285000 47.750999 55.889999

H2 37.455002 49.216999 55.575001

O 42.549000 64.864998 36.325001

H1 42.554001 64.462997 35.435001

H2 43.159000 65.610001 36.209000

O 46.075001 9.037000 44.683998

H1 46.118000 9.046000 43.708000

H2 45.377998 8.380000 44.842999

O 23.705999 65.886002 32.215000

H1 23.653000 65.931999 33.186001

H2 23.108000 65.138000 32.023998

O 40.075001 6.789000 35.161999

H1 39.859001 7.644000 35.564999

H2 39.162998 6.433000 35.000000

O 14.584000 55.592999 48.743000

H1 15.193000 54.917999 49.062000

H2 13.981000 55.720001 49.487999

O 31.629999 5.719000 44.018002

H1 31.000000 6.376000 44.382000

H2 32.460999 6.018000 44.426998

O 67.070999 36.174000 34.967999

H1 66.857002 35.736000 35.803001

H2 67.810997 36.742001 35.206001

O 38.828999 57.298000 47.824001

H1 38.583000 56.601002 48.453999

H2 39.696999 57.004002 47.521999

O 49.176998 43.786999 47.160999

H1 49.176998 44.727001 46.917000

H2 48.638000 43.800999 47.973999

O 43.026001 39.264000 54.842999

H1 42.171001 39.729000 54.912998

H2 43.409000 39.681000 54.049999

O 9.449000 16.479000 34.133999

H1 8.546000 16.200001 34.292000

H2 9.674000 16.054001 33.293999

O 44.556000 47.053001 44.007000

H1 45.438999 46.974998 44.436001

H2 44.187000 47.799000 44.540001

O 54.644001 17.114000 19.855000

H1 53.771999 16.707001 19.993000

H2 55.026001 16.509001 19.183001

O 31.747999 30.389999 67.871002

H1 31.396000 29.813000 67.172997

H2 31.118999 31.146999 67.824997

O 32.992001 7.338000 41.053001

H1 33.012001 8.099000 41.669998

H2 32.238998 7.600000 40.479000

O 16.593000 45.723999 51.456001

H1 17.475000 45.430000 51.168999

H2 16.021000 45.273998 50.810001

O 14.238000 34.926998 19.593000

H1 13.786000 35.487999 20.254000

H2 13.901000 35.298000 18.760000

O 34.584000 63.320999 35.576000

H1 35.301998 62.794998 35.168999

H2 33.803001 62.796001 35.346001

O 48.261002 39.548000 22.809000

H1 47.782001 40.389999 22.950001

H2 47.558998 38.905998 23.054001

O 53.166000 65.209000 35.412998

H1 53.536999 65.223999 34.516998

H2 52.250000 64.945000 35.255001

O 38.132000 46.220001 13.044000

H1 37.813000 45.382999 12.639000

H2 38.134998 45.981998 13.982000

O 43.754002 13.634000 27.000999

H1 43.848999 12.692000 26.781000

H2 43.665001 14.031000 26.108000

O 67.265999 35.318001 29.271999

H1 66.496002 35.548000 29.837999

H2 67.151001 34.362000 29.173000

O 26.399000 36.234001 63.389999

H1 26.396000 35.303001 63.666000

H2 25.715000 36.226002 62.689999

O 19.940001 25.914000 64.348000

H1 19.326000 26.017000 65.103996

H2 20.416000 25.099001 64.611000

O 36.351002 58.960999 28.677000

H1 36.521999 58.603001 27.788000

H2 37.248001 58.985001 29.058001

O 37.797001 35.733002 65.835999

H1 38.493000 35.116001 65.560997

H2 37.636002 36.217999 64.994003

O 28.143000 15.597000 49.875999

H1 28.684999 15.417000 49.097000

H2 28.707001 16.221001 50.372002

O 21.058001 19.325001 23.362000

H1 20.556999 18.990000 24.117001

H2 21.954000 19.415001 23.733999

O 28.863001 3.629000 22.549000

H1 28.084999 3.110000 22.813999

H2 29.379000 2.969000 22.049000

O 42.006001 53.071999 49.457001

H1 42.952999 53.018002 49.255001

H2 41.835999 52.179001 49.824001

O 53.223999 38.487000 19.412001

H1 54.140999 38.340000 19.129999

H2 53.207001 39.439999 19.576000

O 30.051001 40.125000 69.184998

H1 30.958000 39.903999 68.889999

H2 29.596001 40.219002 68.330002

O 7.399000 36.566002 57.928001

H1 6.955000 37.429001 57.980000

H2 6.906000 36.053001 58.594002

O 24.047001 32.073002 56.916000

H1 24.584999 32.736000 56.430000

H2 24.530001 32.014999 57.752998

O 39.379002 19.240999 20.625000

H1 39.341999 18.304001 20.893000

H2 38.828999 19.224001 19.816999

O 55.389000 30.412001 54.987999

H1 55.612000 31.332001 55.209000

H2 56.167999 30.145000 54.460999

O 9.740000 38.004002 58.747002

H1 9.663000 38.098999 59.713001

H2 8.853000 37.672001 58.514999

O 38.066002 16.666000 49.133999

H1 37.708000 16.820000 50.028000

H2 37.999001 17.545000 48.736000

O 58.019001 35.397999 12.343000

H1 58.498001 34.550999 12.227000

H2 57.882000 35.396999 13.312000

O 18.142000 23.726999 52.969002

H1 17.594999 24.462999 53.292999

H2 17.999001 23.052000 53.647999

O 23.113001 57.589001 26.114000

H1 22.790001 57.551998 27.037001

H2 22.289000 57.833000 25.643000

O 10.407000 35.617001 28.129000

H1 9.424000 35.487999 28.183001

H2 10.452000 36.337002 27.483000

O 17.601000 21.924000 59.000000

H1 17.472000 21.059000 58.590000

H2 17.188999 21.790001 59.881001

O 26.273001 33.457001 18.577999

H1 26.135000 32.852001 19.327000

H2 26.194000 34.327999 19.021999

O 59.282001 38.105999 15.144000

H1 59.367001 38.049999 14.177000

H2 58.867001 38.993999 15.236000

O 11.943000 41.992001 58.445000

H1 12.034000 41.098000 58.042999

H2 11.022000 42.200001 58.189999

O 27.908001 5.313000 38.529999

H1 28.761999 5.533000 38.084999

H2 27.752001 4.424000 38.138000

O 14.171000 49.377998 25.773001

H1 14.059000 49.373001 26.735001

H2 13.287000 49.661999 25.466999

O 31.195000 8.349000 39.331001

H1 30.933001 9.247000 39.612000

H2 31.889999 8.540000 38.687000

O 34.577000 56.551998 56.490002

H1 33.676998 56.863998 56.263000

H2 35.034000 56.679001 55.634998

O 37.275002 46.716000 8.381000

H1 36.764999 46.537998 9.201000

H2 37.916000 45.985001 8.402000

O 31.158001 59.098000 27.332001

H1 32.070999 59.313000 27.607000

H2 31.309000 58.747002 26.433001

O 28.320999 53.293999 56.333000

H1 28.603001 53.898998 55.613998

H2 27.468000 52.979000 55.992001

O 28.708000 52.139000 13.628000

H1 28.284000 52.175999 14.494000

H2 29.135000 53.012001 13.562000

O 38.103001 25.575001 47.109001

H1 37.813999 25.014000 46.375000

H2 38.076000 26.462000 46.710999

O 61.423000 27.604000 28.518000

H1 60.970001 28.448000 28.653999

H2 61.624001 27.343000 29.445000

O 19.003000 56.326000 26.912001

H1 18.174000 55.907001 26.625999

H2 19.670000 55.716000 26.528999

O 22.552999 56.926998 34.271999

H1 23.139000 56.153999 34.404999

H2 22.184000 57.035000 35.167999

O 48.693001 16.535999 28.238001

H1 48.518002 16.596001 29.208000

H2 49.438000 15.919000 28.226000

O 46.536999 63.500000 37.088001

H1 46.519001 64.466003 37.160999

H2 45.986000 63.345001 36.300999

O 41.126999 23.642000 65.777000

H1 41.234001 24.606001 65.835999

H2 41.098000 23.398001 66.726997

O 42.480999 12.149000 18.672001

H1 42.310001 13.088000 18.915001

H2 43.396999 12.047000 19.002001

O 59.612000 55.634998 35.091999

H1 59.754002 55.390999 36.025002

H2 60.384998 56.191002 34.914001

O 47.810001 35.311001 14.100000

H1 48.478001 35.111000 14.776000

H2 47.150002 35.812000 14.627000

O 44.415001 16.819000 62.488998

H1 44.019001 16.860001 63.380001

H2 43.953999 17.538000 62.032001

O 20.566000 20.243999 12.888000

H1 20.927000 19.676001 13.588000

H2 20.025000 19.601000 12.378000

O 19.084000 59.937000 35.688999

H1 19.396000 59.438999 34.907001

H2 18.138000 60.033001 35.493999

O 43.532001 12.815000 34.639000

H1 42.860001 12.873000 33.941002

H2 43.695000 11.854000 34.678001

O 29.035000 43.007999 10.446000

H1 28.820999 42.924000 9.504000

H2 28.250000 42.627998 10.869000

O 15.577000 56.984001 43.025002

H1 15.069000 56.869999 43.839001

H2 16.466999 57.174000 43.356998

O 25.280001 23.768000 12.224000

H1 25.417999 23.346001 11.359000

H2 26.125000 24.235001 12.351000

O 34.894001 14.148000 10.295000

H1 35.553001 14.802000 10.574000

H2 35.476002 13.414000 9.996000

O 8.335000 50.764000 38.841000

H1 7.662000 51.384998 38.527000

H2 8.042000 50.574001 39.743000

O 3.981000 26.575001 40.792999

H1 4.848000 26.844000 40.452999

H2 3.772000 25.804001 40.250000

O 27.502001 0.444000 38.744999

H1 27.511999 0.333000 39.728001

H2 28.188000 -0.206000 38.500999

O 40.075001 20.665001 26.288000

H1 40.451000 21.507999 26.007999

H2 39.508999 20.916000 27.031000

O 50.362999 66.643997 25.326000

H1 51.115002 66.039001 25.466000

H2 49.737000 66.063004 24.847000

O 39.487999 61.539001 31.634001

H1 39.188999 62.320000 31.150999

H2 39.049999 61.672001 32.507999

O 32.334000 1.277000 43.990002

H1 31.693001 1.820000 43.488998

H2 32.941002 1.957000 44.330002

O 33.474998 39.105000 8.719000

H1 33.002998 38.973999 9.562000

H2 32.807999 39.556999 8.184000

O 32.316002 51.522999 58.535000

H1 31.507999 51.437000 58.009998

H2 32.095001 52.270000 59.122002

O 29.841000 37.828999 9.115000

H1 29.948000 38.736000 8.779000

H2 29.865000 37.312000 8.282000

O 40.851002 63.660999 24.867001

H1 41.660999 63.521999 25.377001

H2 40.192001 63.827000 25.555000

O 27.830000 46.221001 8.370000

H1 27.716999 45.569000 9.078000

H2 28.805000 46.291000 8.322000

O 46.603001 46.292999 40.321999

H1 46.590000 45.817001 41.166000

H2 46.520000 45.570999 39.678001

O 23.353001 60.219002 19.809999

H1 23.056999 60.875000 20.459000

H2 23.929001 59.643002 20.337000

O 33.976002 34.233002 68.740997

H1 34.576000 33.472000 68.827003

H2 34.291000 34.632999 67.905998

O 48.412998 60.862999 34.681999

H1 47.888000 60.092999 34.409000

H2 49.180000 60.798000 34.075001

O 34.615002 54.938000 19.853001

H1 34.846001 54.587002 20.733000

H2 34.238998 54.129002 19.436001

O 47.120998 17.290001 61.287998

H1 46.174999 17.297001 61.519001

H2 47.264999 18.243000 61.082001

O 12.317000 9.801000 36.798000

H1 12.443000 10.702000 37.125999

H2 12.517000 9.904000 35.847000

O 52.778999 62.931999 26.542999

H1 52.612999 62.007999 26.273001

H2 53.333000 62.817001 27.325001

O 35.785000 17.801001 58.643002

H1 35.180000 18.082001 59.352001

H2 35.417999 16.924000 58.414001

O 13.818000 52.451000 41.866001

H1 13.683000 51.500999 41.712002

H2 13.016000 52.827999 41.459000

O 43.773998 64.043999 16.620001

H1 44.504002 63.745998 16.059000

H2 44.166000 64.829002 17.056000

O 29.093000 10.091000 23.985001

H1 29.868999 10.669000 23.966000

H2 28.408001 10.682000 24.347000

O 25.429001 8.149000 26.158001

H1 26.204000 7.585000 26.301001

H2 24.743999 7.686000 26.683001

O 33.435001 7.700000 29.448000

H1 33.186001 7.278000 28.613001

H2 33.214001 7.020000 30.101999

O 5.873000 21.146999 30.421000

H1 5.416000 20.304001 30.610001

H2 6.757000 20.971001 30.775000

O 44.117001 17.295000 39.015999

H1 44.284000 17.087999 38.081001

H2 44.995998 17.183001 39.409000

O 14.182000 27.295000 50.870998

H1 13.220000 27.372999 50.696999

H2 14.462000 28.221001 50.723000

O 32.551998 15.144000 60.562000

H1 31.664000 14.756000 60.451000

H2 32.525002 15.872000 59.915001

O 38.542000 59.360001 30.245001

H1 38.839001 60.144001 30.750000

H2 39.410000 58.984001 29.976999

O 38.058998 45.118000 47.499001

H1 38.612000 45.382000 48.259998

H2 37.220001 44.883999 47.967999

O 58.962002 22.507999 29.396999

H1 58.157001 22.538000 29.933001

H2 59.493000 23.225000 29.770000

O 53.643002 52.407001 44.187000

H1 52.712002 52.311001 43.945999

H2 53.896999 53.208000 43.680000

O 23.496000 22.448999 61.930000

H1 23.091999 23.238001 62.318001

H2 24.427000 22.546000 62.195999

O 15.122000 47.509998 22.069000

H1 15.032000 46.580002 22.329000

H2 14.197000 47.805000 22.038000

O 49.338001 21.594999 40.671001

H1 49.804001 21.393000 41.505001

H2 48.951000 22.468000 40.880001

O 53.299000 20.837000 35.861000

H1 52.685001 20.642000 35.113998

H2 53.266998 19.985001 36.337002

O 36.563000 28.945000 60.798000

H1 36.490002 29.478001 59.992001

H2 35.669998 28.995001 61.168999

O 37.696999 53.450001 57.773998

H1 36.856998 53.574001 57.308998

H2 37.945000 54.370998 57.990002

O 38.263000 2.973000 40.507999

H1 37.476002 2.441000 40.307999

H2 38.977001 2.390000 40.198002

O 43.306999 20.587999 44.759998

H1 42.945999 21.496000 44.692001

H2 42.514999 20.107000 45.075001

O 43.668999 42.087002 39.442001

H1 44.535000 41.874001 39.012001

H2 44.013000 42.403000 40.314999

O 38.162998 25.259001 60.396999

H1 38.129002 24.702000 61.193001

H2 39.018002 25.693001 60.480999

O 44.261002 44.150002 5.710000

H1 43.625000 44.831001 5.443000

H2 45.032001 44.664001 5.968000

O 33.609001 52.973000 47.178001

H1 34.084000 53.283001 46.395000

H2 33.014000 53.723999 47.368000

O 66.475998 36.012001 20.382999

H1 66.984001 35.294998 19.971001

H2 65.647003 35.558998 20.615000

O 35.962002 9.939000 35.044998

H1 35.584000 9.478000 35.821999

H2 36.445999 10.667000 35.476002

O 27.108999 32.637001 8.345000

H1 27.301001 32.877998 9.265000

H2 26.488001 33.326000 8.071000

O 45.863998 7.447000 31.427000

H1 45.214001 8.084000 31.774000

H2 45.841000 6.748000 32.085999

O 18.176001 54.037998 59.855999

H1 18.551001 53.320000 60.411999

H2 18.990000 54.469002 59.539001

O 35.771000 8.560000 46.248001

H1 34.847000 8.382000 46.020000

H2 35.966000 7.833000 46.867001

O 38.444000 45.252998 60.797001

H1 38.372002 46.084999 61.277000

H2 37.612999 44.798000 61.060001

O 44.605999 12.309000 43.171001

H1 44.764999 13.255000 43.030998

H2 45.324001 11.908000 42.648998

O 29.718000 19.952000 13.901000

H1 30.618000 20.205999 14.179000

H2 29.834000 19.011999 13.695000

O 48.826000 54.827999 24.174000

H1 48.708000 55.282001 23.323000

H2 47.991001 54.341000 24.256001

O 30.535999 46.349998 7.966000

H1 30.658001 46.326000 8.942000

H2 30.907000 47.235001 7.763000

O 45.639999 47.770000 48.542000

H1 44.981998 47.189999 48.130001

H2 45.558998 47.543999 49.479000

O 49.449001 66.362999 35.105000

H1 49.414001 66.348000 34.123001

H2 48.638000 66.862000 35.305000

O 12.753000 43.778999 25.820999

H1 12.843000 44.675999 25.441000

H2 12.448000 43.282001 25.033001

O 34.277000 41.354000 13.610000

H1 34.044998 41.729000 14.481000

H2 34.904999 40.654999 13.857000

O 50.363998 19.892000 47.719002

H1 50.040001 20.658001 47.201000

H2 51.229000 20.261000 48.028999

O 47.012001 47.884998 51.909000

H1 47.921001 47.550999 52.048000

H2 46.523998 47.404999 52.602001

O 36.617001 24.073000 62.528999

H1 35.917000 23.725000 61.929001

H2 36.701000 23.326000 63.153999

O 28.360001 1.658000 33.849998

H1 28.055000 1.252000 33.019001

H2 27.559999 2.100000 34.168999

O 60.556999 31.629000 32.270000

H1 61.450001 31.858999 31.959000

H2 60.153000 32.500999 32.402000

O 12.091000 26.245001 32.089001

H1 11.592000 26.164000 32.923000

H2 11.602000 26.950001 31.632999

O 17.680000 20.037001 32.217999

H1 17.687000 19.806999 31.264000

H2 18.187000 19.305000 32.595001

O 58.243000 27.562000 22.458000

H1 57.973999 28.500999 22.549999

H2 57.445999 27.204000 22.000999

O 34.067001 65.944000 43.485001

H1 33.419998 65.481003 42.917999

H2 33.514999 66.684998 43.814999

O 34.258999 60.506001 44.296001

H1 34.389999 59.553001 44.390999

H2 35.181000 60.839001 44.403999

O 15.768000 57.551998 33.351002

H1 14.863000 57.653999 32.957001

H2 15.620000 56.747002 33.888000

O 32.858002 47.817001 65.414001

H1 32.268002 47.917000 66.171997

H2 32.448002 47.078999 64.931000

O 29.528999 14.980000 57.798000

H1 28.778000 15.570000 57.921001

H2 29.591000 14.531000 58.659000

O 35.118000 50.222000 11.880000

H1 35.653999 50.053001 12.679000

H2 34.374001 49.610001 12.012000

O 32.362999 52.889999 60.967999

H1 33.316002 52.981998 60.789001

H2 32.230000 53.516998 61.688000

O 20.622999 1.465000 42.749001

H1 20.415001 2.365000 43.078999

H2 20.132000 0.927000 43.408001

O 23.507999 36.667000 67.435997

H1 23.018999 37.118999 68.139999

H2 24.414000 36.959999 67.581001

O 40.648998 27.386999 13.256000

H1 40.285999 28.281000 13.125000

H2 41.535000 27.587000 13.611000

O 32.351002 26.051001 61.109001

H1 31.510000 25.594000 60.973000

H2 32.613998 25.722000 61.992001

O 17.601999 10.397000 24.205999

H1 17.254000 11.296000 24.072001

H2 18.559999 10.553000 24.191999

O 51.908001 35.157001 61.155998

H1 51.502998 35.451000 60.310001

H2 51.198002 35.345001 61.785000

O 17.799999 56.167999 31.437000

H1 17.694000 56.806000 32.160000

H2 18.018000 55.354000 31.917999

O 39.077999 48.091999 20.834000

H1 39.103001 49.049000 20.667000

H2 39.396999 48.053001 21.757000

O 38.605999 44.049000 15.512000

H1 39.410000 44.570999 15.666000

H2 38.931000 43.330002 14.950000

O 49.937000 7.318000 41.810001

H1 49.757000 6.374000 41.743000

H2 49.387001 7.576000 42.576000

O 35.778999 6.308000 21.292000

H1 36.553001 5.728000 21.420000

H2 35.296001 6.186000 22.118000

O 27.472000 57.464001 41.466000

H1 27.077999 56.775002 42.009998

H2 27.118000 57.289001 40.584999

O 2.186000 30.309000 35.556000

H1 2.932000 30.393000 36.173000

H2 2.635000 29.982000 34.750999

O 46.485001 39.449001 52.110001

H1 46.327000 39.646000 51.175999

H2 45.908001 40.097000 52.550999

O 28.979000 6.369000 41.169998

H1 28.475000 5.964000 40.445999

H2 29.586000 5.656000 41.417999

O 54.736000 38.754002 38.734001

H1 54.205002 38.109001 38.245998

H2 54.217999 38.895000 39.534000

O 28.455000 28.233000 67.212997

H1 28.650999 27.955999 68.124001

H2 29.330000 28.223000 66.800003

O 28.711000 60.181999 31.277000

H1 27.902000 60.736000 31.250999

H2 28.316000 59.285000 31.302999

O 4.252000 30.280001 37.790001

H1 4.893000 30.431000 37.078999

H2 4.555000 30.912001 38.470001

O 37.432999 43.673000 54.439999

H1 38.195999 43.813999 55.025002

H2 37.549999 44.389999 53.792000

O 30.391001 45.912998 10.659000

H1 29.794001 45.313999 11.134000

H2 30.834999 46.369999 11.403000

O 13.345000 42.126999 41.914001

H1 14.254000 41.987000 42.228001

H2 12.880000 41.365002 42.304001

O 48.382000 58.931999 20.539000

H1 49.250000 59.355000 20.583000

H2 48.089001 59.155998 19.636000

O 22.716999 13.153000 19.003000

H1 23.583000 12.865000 18.663000

H2 22.346001 12.299000 19.312000

O 18.587999 39.959000 16.027000

H1 18.820999 40.865002 16.280001

H2 19.152000 39.435001 16.628000

O 19.596001 4.344000 47.558998

H1 20.271000 3.905000 48.112000

H2 18.787001 3.887000 47.862999

O 36.683998 37.210999 14.337000

H1 36.563999 37.129002 13.371000

H2 36.473999 38.153000 14.479000

O 31.365999 51.630001 18.260000

H1 30.927999 51.693001 19.135000

H2 32.167999 52.157001 18.408001

O 29.589001 59.521999 19.263000

H1 30.021000 58.827999 18.716000

H2 28.818001 59.737000 18.687000

O 56.592999 12.283000 40.374001

H1 57.216000 12.858000 39.901001

H2 55.938000 12.937000 40.702000

O 18.796000 22.316999 13.748000

H1 19.045000 23.018000 13.111000

H2 19.301001 21.559999 13.410000

O 59.564999 38.804001 37.578999

H1 58.616001 38.687000 37.380001

H2 59.904999 39.062000 36.689999

O 54.530998 52.813999 24.674000

H1 53.598000 52.733002 24.927999

H2 54.762001 51.907001 24.434999

O 43.960999 39.258999 66.094002

H1 44.293999 39.278000 65.171997

H2 44.689999 39.699001 66.561996

O 40.964001 29.902000 18.385000

H1 40.720001 30.455000 19.164000

H2 40.456001 29.094000 18.570000

O 47.765999 45.191002 49.237999

H1 46.874001 45.327999 48.856998

H2 47.534000 44.993999 50.167999

O 21.608000 15.490000 19.766001

H1 21.299000 15.189000 20.638000

H2 22.024000 14.667000 19.423000

O 16.389999 14.386000 36.776001

H1 16.007999 15.204000 37.141998

H2 15.759000 14.166000 36.075001

O 21.599001 26.655001 54.800999

H1 21.917000 26.402000 55.688999

H2 22.176001 27.424999 54.613998

O 13.273000 22.135000 25.295000

H1 13.932000 21.448000 25.480000

H2 13.135000 22.514000 26.183001

O 54.338001 42.014000 37.196999

H1 55.016998 41.450001 37.611000

H2 54.792000 42.862999 37.133999

O 13.179000 38.071999 25.481001

H1 12.598000 38.256001 24.709000

H2 13.895000 37.570999 25.046000

O 34.516998 14.832000 21.257999

H1 34.932999 15.057000 22.113001

H2 33.793999 15.474000 21.209000

O 10.170000 39.299000 54.326000

H1 10.288000 38.334000 54.237999

H2 10.152000 39.592999 53.409000

O 59.998001 52.639000 33.787998

H1 59.730999 52.696999 32.853001

H2 60.498001 53.452999 33.912998

O 37.055000 6.356000 47.807999

H1 37.977001 6.622000 47.637001

H2 37.030998 5.455000 47.469002

O 7.487000 33.386002 52.687000

H1 7.813000 33.176998 53.571999

H2 7.903000 32.708000 52.139000

O 52.958000 41.453999 43.238998

H1 52.535999 41.801998 42.445000

H2 53.209999 42.268002 43.713001

O 7.320000 31.136000 33.374001

H1 6.974000 30.846001 34.242001

H2 6.943000 30.466000 32.783001

O 22.676001 7.480000 53.058998

H1 22.940001 8.132000 53.730999

H2 23.274000 7.697000 52.327999

O 47.469002 13.353000 28.708000

H1 47.337002 12.515000 28.212999

H2 48.334999 13.637000 28.360001

O 51.442001 54.049000 30.200001

H1 51.858002 54.002998 31.068001

H2 51.856998 54.832001 29.802999

O 13.380000 55.912998 20.690001

H1 13.519000 56.645000 20.061001

H2 13.080000 55.201000 20.105000

O 36.348000 43.763000 61.203999

H1 35.855999 43.590000 60.389000

H2 35.674999 43.653000 61.887001

O 24.601000 60.228001 24.052999

H1 24.194000 59.396000 23.750999

H2 25.295000 60.363998 23.393000

O 17.804001 52.320999 57.393002

H1 17.808001 53.022999 58.070000

H2 17.014999 52.553001 56.875000

O 43.112999 9.185000 19.774000

H1 43.553001 10.011000 19.513000

H2 42.208000 9.501000 19.978001

O 30.659000 43.620998 7.311000

H1 30.650000 44.527000 7.669000

H2 30.223000 43.743999 6.451000

O 46.967999 25.614000 64.851997

H1 47.686001 25.177999 65.333000

H2 47.115002 26.555000 65.097000

O 12.188000 23.114000 52.501999

H1 13.047000 23.569000 52.457001

H2 12.175000 22.783001 53.412998

O 12.328000 31.188999 15.358000

H1 12.790000 31.761000 16.002001

H2 12.761000 30.336000 15.496000

O 17.420000 46.952000 61.722000

H1 17.573999 46.903000 60.763000

H2 16.815001 46.213001 61.865002

O 60.777000 22.457001 34.421001

H1 60.185001 22.712000 33.694000

H2 60.855000 23.297001 34.912998

O 29.950001 32.792999 7.906000

H1 30.534000 32.483002 8.618000

H2 29.070000 32.694000 8.298000

O 56.460999 52.081001 28.475000

H1 56.794998 51.480999 29.162001

H2 56.162998 52.845001 29.017000

O 27.436001 8.269000 23.305000

H1 27.819000 7.626000 22.681000

H2 28.219999 8.829000 23.509001

O 24.788000 17.700001 30.257999

H1 25.754000 17.596001 30.374001

H2 24.452999 17.218000 31.039000

O 29.132999 21.820999 9.140000

H1 29.343000 20.996000 9.604000

H2 29.992001 22.264999 9.111000

O 41.576000 16.349001 39.592999

H1 42.448002 16.771000 39.469002

H2 41.825001 15.399000 39.492001

O 24.527000 40.959999 8.541000

H1 23.999001 40.473999 9.206000

H2 24.714001 41.792000 9.000000

O 26.973000 8.953000 34.617001

H1 27.226999 9.701000 35.176998

H2 26.844999 8.243000 35.279999

O 30.157000 3.324000 44.647999

H1 29.403000 3.441000 44.050999

H2 30.857000 3.819000 44.199001

O 8.386000 38.823002 32.869999

H1 7.936000 38.526001 32.050999

H2 9.075000 39.405998 32.508999

O 45.784000 20.796000 63.063000

H1 44.898998 20.739000 63.460999

H2 46.070999 21.681000 63.341000

O 11.483000 47.134998 41.605000

H1 12.213000 46.884998 42.208000

H2 10.776000 46.526001 41.904999

O 10.249000 4.546000 34.632000

H1 10.782000 5.319000 34.396000

H2 9.447000 4.954000 34.995998

O 17.879999 47.108002 23.084000

H1 18.301001 47.966000 22.864000

H2 17.051001 47.182999 22.572001

O 20.299999 15.027000 22.628000

H1 19.509001 15.580000 22.527000

H2 20.648001 15.347000 23.485001

O 61.469002 27.809999 23.761000

H1 60.960999 27.118000 24.209999

H2 61.474998 27.507000 22.843000

O 57.066002 37.804001 36.789001

H1 56.971001 38.457001 36.057999

H2 56.122002 37.597000 36.955002

O 41.119999 17.198000 45.271000

H1 40.823002 18.094000 45.504002

H2 40.693001 16.663000 45.966999

O 58.124001 13.672000 29.791000

H1 57.237000 13.417000 29.486000

H2 57.939999 13.932000 30.715000

O 12.991000 30.346001 29.127001

H1 12.391000 30.412001 29.885000

H2 13.547000 31.142000 29.247999

O 48.958000 46.595001 15.119000

H1 48.419998 45.964001 14.591000

H2 48.490002 47.424999 14.952000

O 25.181999 56.827999 31.752001

H1 24.745001 57.657001 32.018002

H2 25.872999 57.146000 31.150999

O 30.277000 17.256001 60.806000

H1 30.618999 16.879000 61.645000

H2 29.316999 17.180000 60.945000

O 64.800003 35.674000 17.295000

H1 64.150002 35.743000 18.014999

H2 64.334999 35.075001 16.677000

O 44.279999 38.844002 15.857000

H1 44.571999 39.750999 16.058001

H2 44.341000 38.845001 14.874000

O 63.215000 30.690001 37.411999

H1 64.098999 30.632999 37.019001

H2 62.901001 31.548000 37.084000

O 48.098999 12.491000 51.027000

H1 47.301998 11.983000 51.250000

H2 47.846001 13.390000 51.272999

O 43.240002 62.557999 26.959000

H1 42.955002 61.653999 26.739000

H2 43.889000 62.384998 27.669001

O 27.910000 3.412000 31.395000

H1 27.865999 3.888000 32.236000

H2 27.304001 2.673000 31.533001

O 58.812000 23.195999 18.664000

H1 59.335999 22.421000 18.947001

H2 58.486000 23.534000 19.520000

O 49.518002 57.514000 31.566000

H1 49.431000 57.029999 30.716000

H2 48.883999 57.028999 32.119999

O 18.176001 49.509998 47.268002

H1 17.457001 49.943001 46.777000

H2 18.138000 48.605000 46.918999

O 36.373001 15.096000 54.097000

H1 36.306999 14.817000 55.027000

H2 37.314999 14.951000 53.915001

O 31.193001 49.216000 55.658001

H1 31.139000 48.298000 55.987000

H2 30.499001 49.652000 56.188999

O 40.592999 56.252998 36.271000

H1 41.263000 56.900002 36.556000

H2 40.966999 55.421001 36.615002

O 26.736000 31.150999 60.407001

H1 27.455000 31.264999 59.757000

H2 27.177000 31.296000 61.245998

O 34.145000 26.594000 56.000999

H1 33.834999 27.510000 56.105000

H2 34.816002 26.691000 55.297001

O 18.298000 39.301998 65.186996

H1 18.789000 38.986000 64.405998

H2 17.754000 40.006001 64.803001

O 39.110001 9.206000 24.427000

H1 39.150002 9.446000 25.372999

H2 39.439999 10.029000 24.010000

O 21.864000 6.579000 56.911999

H1 21.384001 6.199000 57.674000

H2 21.282000 7.305000 56.652000

O 38.860001 17.796000 27.266001

H1 38.666000 18.605000 27.766001

H2 37.994999 17.573999 26.893999

O 5.425000 43.685001 37.530998

H1 6.202000 43.743000 38.122002

H2 4.695000 43.734001 38.189999

O 44.041000 45.834000 37.833000

H1 43.257000 46.356998 37.555000

H2 43.768002 45.625000 38.761002

O 24.114000 37.563999 8.312000

H1 25.073999 37.411999 8.166000

H2 23.753000 37.287998 7.451000

O 54.726002 13.389000 34.474998

H1 54.917999 12.801000 33.729000

H2 54.909000 14.265000 34.113998

O 43.431999 8.919000 32.425999

H1 42.904999 9.335000 31.716000

H2 42.919998 8.100000 32.583000

O 20.785999 18.597000 15.369000

H1 20.296000 17.826000 14.997000

H2 20.072001 19.010000 15.899000

O 6.045000 37.430000 52.863998

H1 5.943000 37.340000 53.832001

H2 6.937000 37.071999 52.726002

O 31.138000 17.212000 15.327000

H1 30.412001 16.733999 14.893000

H2 30.674999 17.691000 16.028999

O 52.290001 11.156000 21.659000

H1 52.457001 10.290000 22.077999

H2 51.696999 11.572000 22.313000

O 35.581001 25.221001 59.519001

H1 35.139000 26.013000 59.841000

H2 36.476002 25.313000 59.911999

O 60.862000 30.891001 25.724001

H1 61.261002 30.368999 25.010000

H2 59.912998 30.805000 25.528999

O 61.208000 50.383999 22.079000

H1 61.555000 50.977001 21.391001

H2 62.030998 50.116001 22.537001

O 56.414001 18.274000 23.964001

H1 55.660000 18.478001 23.378000

H2 57.091000 18.884001 23.624001

O 42.094002 54.924000 52.430000

H1 41.330002 55.491001 52.275002

H2 41.748001 54.034000 52.285999

O 21.886999 62.041000 40.094002

H1 22.509001 61.330002 39.851002

H2 21.073999 61.749001 39.644001

O 40.439999 40.160999 55.511002

H1 40.014000 39.681000 56.241001

H2 40.645000 41.016998 55.928001

O 27.902000 4.154000 28.014000

H1 28.534000 3.571000 28.457001

H2 28.200001 5.035000 28.287001

O 23.438999 21.766001 57.098000

H1 23.224001 22.485001 57.721001

H2 24.271000 21.427999 57.494999

O 53.251999 12.554000 25.183001

H1 53.706001 11.734000 25.452999

H2 53.980000 13.086000 24.841999

O 39.956001 23.283001 24.170000

H1 40.057999 23.907000 23.424000

H2 39.285999 22.673000 23.811001

O 23.886999 40.806999 64.390999

H1 24.448999 40.025002 64.223999

H2 23.379999 40.506001 65.172997

O 39.473999 61.932999 45.688999

H1 39.252998 61.125999 46.192001

H2 39.825001 61.566002 44.865002

O 62.284000 51.452000 35.188999

H1 62.623001 50.851002 34.500000

H2 61.570999 51.910999 34.717999

O 13.315000 9.542000 47.534000

H1 12.568000 10.124000 47.759998

H2 13.492000 9.808000 46.612000

O 20.027000 49.563000 55.449001

H1 20.267000 50.446999 55.784000

H2 19.082001 49.695999 55.240002

O 43.330002 11.368000 25.396999

H1 43.560001 10.456000 25.686001

H2 43.132999 11.211000 24.457001

O 18.902000 35.173000 62.070999

H1 18.792000 35.387001 63.014000

H2 18.524000 34.283001 62.026001

O 20.944000 29.559999 63.596001

H1 20.601999 29.539000 62.674999

H2 21.482000 30.365999 63.584000

O 51.863998 52.785999 26.271000

H1 52.292000 52.506001 27.110001

H2 50.943001 52.535000 26.438000

O 51.367001 59.775002 21.784000

H1 51.770000 60.035999 22.618000

H2 51.652000 60.476002 21.176001

O 15.938000 25.502001 59.950001

H1 15.820000 26.471001 59.942001

H2 15.255000 25.233000 60.590000

O 39.189999 16.014999 52.459000

H1 38.425999 16.528000 52.134998

H2 39.632000 16.658001 53.035000

O 14.057000 11.651000 38.249001

H1 15.020000 11.492000 38.276001

H2 13.946000 12.039000 37.368000

O 51.585999 24.504000 38.705002

H1 51.532001 23.587000 38.351002

H2 50.669998 24.629000 39.026001

O 27.193001 30.478001 63.525002

H1 27.656000 29.899000 62.916000

H2 27.910999 30.837999 64.077003

O 36.431000 50.548000 58.224998

H1 36.986000 51.310001 58.000000

H2 36.884998 49.823002 57.759998

O 64.804001 21.971001 29.801001

H1 63.855999 22.044001 30.007999

H2 64.853996 22.364000 28.921000

O 45.639999 59.098000 38.180000

H1 44.764000 59.505001 38.325001

H2 46.186001 59.907001 38.028999

O 18.333000 60.542000 25.556999

H1 17.562000 60.410999 26.143000

H2 18.313000 61.500999 25.426001

O 9.185000 33.068001 40.203999

H1 8.862000 32.185001 39.980000

H2 8.985000 33.563000 39.377998

O 41.883999 13.712000 39.679001

H1 41.597000 12.831000 39.979000

H2 42.396999 13.485000 38.877998

O 35.459000 10.158000 17.454000

H1 35.000000 10.900000 17.898001

H2 35.594002 9.550000 18.198000

O 40.561001 61.523998 42.724998

H1 41.118999 60.969002 43.318001

H2 41.012001 62.384998 42.806999

O 24.207001 58.951000 36.136002

H1 23.447001 58.362000 35.967999

H2 23.756001 59.709000 36.558998

O 8.870000 32.901001 43.827999

H1 8.054000 32.998001 44.352001

H2 8.918000 31.936001 43.730000

O 51.612999 7.317000 35.192001

H1 50.957001 7.697000 34.591000

H2 51.118000 6.621000 35.640999

O 43.493999 62.375000 45.845001

H1 43.643002 63.221001 45.361000

H2 42.716000 62.598999 46.375999

O 37.094002 19.886999 25.440001

H1 36.824001 19.517000 24.579000

H2 37.646000 20.634001 25.171000

O 41.175999 40.243000 67.021004

H1 40.749001 39.964001 66.194000

H2 42.099998 40.347000 66.740997

O 18.816000 48.226002 58.428001

H1 17.927999 48.035000 58.063000

H2 19.250000 47.365002 58.328999

O 7.510000 25.377001 42.208000

H1 8.172000 25.155001 41.519001

H2 6.806000 25.775000 41.675999

O 25.530001 63.887001 32.326000

H1 24.971001 64.672997 32.213001

H2 25.613001 63.833000 33.296001

O 16.999001 10.655000 48.578999

H1 16.974001 9.705000 48.797001

H2 17.111000 10.612000 47.601002

O 53.387001 32.667000 38.627998

H1 54.226002 32.966000 39.008999

H2 52.738998 33.125999 39.205002

O 45.910000 36.533001 15.569000

H1 45.547001 37.436001 15.512000

H2 45.932999 36.405998 16.542000

O 16.947001 22.834000 48.275002

H1 17.447001 23.224001 47.541000

H2 16.077999 22.669001 47.862000

O 50.834999 23.112000 49.867001

H1 51.284000 23.957001 49.660999

H2 51.606998 22.545000 50.085999

O 15.855000 41.487999 50.764999

H1 16.777000 41.383999 51.041000

H2 15.580000 42.289001 51.231998

O 36.577999 35.577999 18.115000

H1 35.827000 36.201000 18.083000

H2 36.369999 34.972000 17.388000

O 52.146000 50.338001 24.042999

H1 52.020000 49.884998 24.891001

H2 52.848999 49.817001 23.629000

O 41.757000 22.955999 44.890999

H1 41.874001 23.747999 45.443001

H2 40.890999 23.122000 44.484001

O 40.577000 46.595001 42.834000

H1 41.136002 47.161999 42.247002

H2 40.530998 45.786999 42.266998

O 11.767000 39.178001 35.125000

H1 12.328000 39.137001 34.335999

H2 12.296000 38.632000 35.757999

O 40.574001 40.575001 64.238998

H1 39.639999 40.782001 64.362000

H2 40.606998 40.273998 63.318001

O 11.902000 45.775002 31.506001

H1 12.433000 45.092999 31.065001

H2 12.551000 46.483002 31.639999

O 36.903000 7.768000 31.671000

H1 37.773998 8.018000 32.019001

H2 36.438000 8.622000 31.666000

O 47.125999 45.411999 35.098000

H1 47.155998 46.250999 34.580002

H2 47.865002 44.928001 34.698002

O 43.400002 48.230999 10.588000

H1 42.474998 48.091999 10.848000

H2 43.441002 47.719002 9.757000

O 13.616000 16.652000 46.008999

H1 13.535000 17.080999 46.873001

H2 14.557000 16.743000 45.812000

O 47.493999 28.832001 22.202000

H1 48.069000 29.518999 21.802000

H2 47.043999 28.480000 21.423000

O 33.700001 10.160000 39.207001

H1 32.938000 10.479000 39.716000

H2 34.429001 10.264000 39.839001

O 32.391998 9.950000 45.264000

H1 31.841999 9.160000 45.134998

H2 32.756001 10.094000 44.375999

O 6.944000 19.749001 40.560001

H1 5.978000 19.645000 40.430000

H2 7.090000 20.615999 40.118999

O 26.018999 49.687000 10.961000

H1 25.936001 49.172001 10.140000

H2 25.097000 49.936001 11.134000

O 17.391001 25.100000 17.757000

H1 17.013000 25.072001 16.865000

H2 17.233000 24.181000 18.063000

O 18.521999 26.354000 62.035999

H1 18.742001 26.249001 62.987000

H2 18.402000 25.421000 61.776001

O 12.590000 17.271999 50.710999

H1 13.470000 17.690001 50.831001

H2 12.423000 16.934000 51.608002

O 24.308001 17.938999 56.908001

H1 23.938999 18.414000 56.131001

H2 25.030001 17.443001 56.477001

O 47.868999 33.438000 57.926998

H1 48.119999 32.943001 58.729000

H2 48.153999 34.338001 58.161999

O 39.681999 46.257000 49.487000

H1 38.973999 46.914001 49.683998

H2 40.090000 46.701000 48.701000

O 13.140000 33.549000 33.332001

H1 12.973000 32.632999 33.078999

H2 12.378000 34.015999 32.942001

O 20.783001 2.932000 37.282001

H1 20.625999 3.340000 38.148998

H2 20.009001 2.358000 37.187000

O 30.837000 12.389000 56.599998

H1 31.108999 12.751000 55.742001

H2 31.343000 12.934000 57.223000

O 21.877001 20.065001 55.756001

H1 20.993000 20.398001 55.972000

H2 22.451000 20.697001 56.235001

O 14.969000 19.077000 21.683001

H1 15.320000 18.801001 22.554001

H2 14.530000 18.267000 21.384001

O 62.688999 48.189999 25.973000

H1 62.291000 48.560001 26.778000

H2 62.341999 47.282001 25.981001

O 48.125000 63.233002 33.433998

H1 47.301998 63.488998 33.001999

H2 47.810001 62.626999 34.133999

O 46.106998 28.224001 57.931000

H1 46.459000 27.556999 57.321999

H2 46.778000 28.222000 58.639000

O 46.458000 57.255001 40.681000

H1 47.292000 57.644001 40.991001

H2 46.193001 57.847000 39.970001

O 51.053001 47.801998 37.667000

H1 51.549000 47.820000 36.827000

H2 50.632999 46.912998 37.611000

O 27.525999 17.770000 57.606998

H1 28.115999 18.278000 57.018002

H2 26.978001 17.288000 56.965000

O 38.105999 11.125000 53.418999

H1 37.306000 11.434000 53.867001

H2 37.730999 10.728000 52.604000

O 61.933998 26.775000 31.025999

H1 62.824001 26.495001 30.735001

H2 62.048000 26.844999 31.983999

O 18.028999 29.139999 49.268002

H1 17.548000 29.771999 49.820999

H2 18.955000 29.410999 49.398998

O 33.090000 4.644000 41.750999

H1 33.215000 5.526000 41.348000

H2 32.532001 4.870000 42.519001

O 26.534000 44.768002 17.148001

H1 26.547001 45.715000 17.357000

H2 25.673000 44.498001 17.518000

O 15.321000 53.138000 56.220001

H1 15.086000 53.450001 55.321999

H2 14.438000 53.078999 56.631001

O 44.313999 47.025002 34.341999

H1 43.834000 47.208000 33.520000

H2 44.491001 46.063999 34.241001

O 44.164001 9.368000 23.572001

H1 44.063000 10.051000 22.886999

H2 43.958000 8.557000 23.080000

O 56.611000 15.218000 31.746000

H1 56.644001 16.188000 31.867001

H2 55.737000 15.016000 32.125000

O 29.733000 45.078999 71.268997

H1 29.482000 44.432999 70.573997

H2 28.872000 45.314999 71.633003

O 53.994999 35.063999 57.660999

H1 54.018002 34.150002 57.999001

H2 54.717999 35.479000 58.146999

O 8.414000 41.102001 55.785999

H1 9.159000 40.602001 55.415001

H2 7.659000 40.721001 55.289001

O 55.171001 57.806999 36.550999

H1 55.233002 58.202999 35.666000

H2 54.813999 58.544998 37.073002

O 55.036999 54.875999 26.912001

H1 54.956001 53.979000 26.556000

H2 55.394001 54.716000 27.799999

O 51.028999 27.101999 46.907001

H1 51.014999 27.089001 45.930000

H2 50.077999 27.098000 47.112000

O 33.873001 49.595001 56.915001

H1 32.993000 49.952000 57.118999

H2 34.456001 50.301998 57.227001

O 58.068001 33.396000 49.136002

H1 57.834999 34.278999 49.486000

H2 57.812000 32.820000 49.868000

O 41.645000 57.676998 49.935001

H1 41.952000 58.292999 49.231998

H2 41.916000 56.825001 49.558998

O 19.274000 57.818001 37.325001

H1 18.697001 57.117001 36.972000

H2 19.080000 58.557999 36.720001

O 38.766998 29.452000 12.700000

H1 37.865002 29.111000 12.596000

H2 38.803001 29.667999 13.641000

O 15.171000 14.502000 43.185001

H1 14.337000 14.068000 43.444000

H2 15.750000 14.258000 43.938999

O 9.938000 31.476999 32.756001

H1 10.351000 30.712999 33.215000

H2 9.005000 31.370001 33.047001

O 43.717999 38.057999 59.722000

H1 43.863998 38.639999 58.950001

H2 42.845001 37.681000 59.509998

O 46.582001 66.463997 32.590000

H1 47.541000 66.565002 32.445000

H2 46.252998 67.349998 32.355000

O 58.048000 26.059000 34.841999

H1 57.412998 26.705000 35.192001

H2 58.205002 26.389999 33.942001

O 31.042000 12.199000 21.990999

H1 30.355000 12.892000 21.982000

H2 31.356001 12.253000 22.910000

O 25.434000 14.788000 17.594000

H1 25.667000 15.689000 17.844999

H2 26.294001 14.407000 17.349001

O 52.368000 38.681999 53.987999

H1 53.110001 38.054001 54.019001

H2 51.743000 38.229000 53.403999

O 45.700001 40.237999 20.952000

H1 45.136002 40.014000 21.724001

H2 45.174000 40.990002 20.584000

O 37.410000 10.561000 48.250000

H1 36.583000 10.161000 47.945000

H2 37.081001 11.344000 48.737999

O 39.230999 0.314000 23.492001

H1 40.048000 0.502000 24.000999

H2 38.555000 0.663000 24.091999

O 17.490000 55.662998 53.481998

H1 17.938000 55.759998 54.337002

H2 16.565001 55.584000 53.723999

O 23.518000 56.331001 39.425999

H1 23.169001 55.491001 39.771000

H2 23.049999 56.979000 39.972000

O 34.284000 3.146000 44.612000

H1 34.203999 3.865000 45.266998

H2 35.242001 3.060000 44.527000

O 26.143000 20.997999 17.457001

H1 26.339001 20.552000 18.295000

H2 25.976000 20.257000 16.860001

O 40.485001 35.595001 13.680000

H1 40.518002 36.569000 13.777000

H2 39.764999 35.386002 14.315000

O 23.605000 64.459999 50.368999

H1 23.257000 63.573002 50.558998

H2 22.848000 65.028999 50.591999

O 33.497002 59.967999 22.459999

H1 32.794998 60.230000 23.084999

H2 33.277000 60.493999 21.674999

O 43.431999 54.366001 18.886999

H1 44.168999 54.046001 18.337999

H2 43.848999 54.417000 19.764999

O 50.119999 51.285000 18.724001

H1 50.866001 51.874001 18.521999

H2 50.441002 50.828999 19.520000

O 20.079000 62.935001 28.177000

H1 20.502001 62.054001 28.191999

H2 19.941999 63.071999 27.232000

O 49.944000 21.100000 58.993000

H1 49.439999 20.568001 58.355999

H2 50.514000 20.433001 59.404999

O 27.447001 35.414001 11.431000

H1 26.701000 35.914001 11.034000

H2 27.086000 35.214001 12.314000

O 34.636002 8.788000 37.132000

H1 34.575001 7.864000 37.452000

H2 34.464001 9.275000 37.967999

O 41.522999 62.146000 16.517000

H1 42.193001 62.838001 16.632000

H2 41.167000 62.057999 17.421000

O 12.646000 3.361000 40.978001

H1 12.273000 2.715000 41.605999

H2 11.908000 3.501000 40.369999

O 40.903000 50.346001 22.332001

H1 41.606998 49.743000 22.636999

H2 41.412998 50.990002 21.813999

O 22.327000 24.688000 26.400000

H1 23.132000 24.756001 25.857000

H2 22.252001 25.593000 26.754999

O 64.862999 28.183001 30.195999

H1 64.546997 27.260000 30.222000

H2 65.512001 28.136000 29.462000

O 36.314999 36.714001 11.609000

H1 37.029999 37.340000 11.388000

H2 35.827999 36.694000 10.751000

O 22.023001 32.144001 63.709999

H1 22.892000 31.761999 63.492001

H2 22.181999 32.492001 64.607002

O 61.224998 36.716999 33.810001

H1 61.667000 37.053001 34.613998

H2 60.319000 36.576000 34.140999

O 23.974001 25.282000 65.019997

H1 24.607000 25.523001 65.713997

H2 23.414000 26.080000 64.981003

O 13.794000 54.536999 29.615999

H1 14.489000 53.993999 30.030001

H2 14.293000 55.362000 29.434999

O 20.228001 22.579000 58.960999

H1 19.274000 22.629000 59.181999

H2 20.312000 21.624001 58.764999

O 51.729000 22.457001 56.174000

H1 51.599998 21.503000 56.250999

H2 51.841000 22.719000 57.112999

O 37.453999 64.010002 40.314999

H1 38.216000 64.182999 39.747002

H2 37.002998 64.876999 40.320999

O 5.305000 37.551998 40.424999

H1 5.379000 36.639000 40.726002

H2 4.781000 37.970001 41.118999

O 67.153000 40.980999 35.407001

H1 67.116997 41.875000 34.999001

H2 66.218002 40.858002 35.653000

O 12.733000 31.919001 44.285999

H1 12.100000 31.985001 43.547001

H2 13.576000 31.820999 43.813000

O 19.476000 32.689999 66.321999

H1 19.447001 33.609001 66.004997

H2 18.596001 32.588001 66.723000

O 44.557999 55.513000 41.131001

H1 43.743000 55.813000 40.702999

H2 45.198002 56.202999 40.837002

O 38.980999 4.475000 23.516001

H1 38.924999 5.376000 23.148001

H2 38.596001 4.601000 24.400000

O 41.941002 40.785000 13.946000

H1 42.140999 41.347000 13.188000

H2 42.636002 41.023998 14.576000

O 47.099998 51.092999 35.120998

H1 46.532001 51.700001 34.605999

H2 47.587002 51.731998 35.681000

O 29.143999 44.316002 15.929000

H1 29.327999 43.473999 15.458000

H2 28.233000 44.158001 16.240000

O 48.064999 11.093000 38.981998

H1 47.890999 10.145000 38.963001

H2 47.375000 11.448000 38.401001

O 46.096001 50.025002 45.574001

H1 46.687000 50.488998 44.962002

H2 46.713001 49.498001 46.102001

O 28.504000 23.549999 53.355000

H1 29.347000 23.731001 52.924000

H2 28.643000 22.684999 53.758999

O 14.413000 49.256001 43.597000

H1 14.972000 49.109001 42.819000

H2 13.739000 48.562000 43.481998

O 37.320999 5.595000 18.740999

H1 36.488998 6.002000 19.056000

H2 37.028999 4.669000 18.611000

O 29.826000 40.696999 7.984000

H1 28.888000 40.641998 7.718000

H2 29.951000 41.652000 8.072000

O 26.584000 23.377001 23.264000

H1 27.483999 23.712000 23.451000

H2 26.683001 22.431999 23.462000

O 16.528000 42.833000 57.701000

H1 15.621000 43.187000 57.584999

H2 16.613001 42.862000 58.669998

O 22.232000 35.987999 64.657997

H1 22.958000 35.998001 64.014000

H2 22.566000 36.585999 65.347000

O 26.802000 51.497002 59.198002

H1 27.614000 51.013000 59.417999

H2 26.127001 50.810001 59.245998

O 40.484001 38.202999 43.130001

H1 41.438000 38.199001 42.935001

H2 40.390999 39.063999 43.595001

O 10.257000 29.643999 47.912998

H1 10.708000 30.319000 47.363998

H2 9.408000 29.552999 47.460999

O 8.406000 46.435001 23.655001

H1 8.420000 45.453999 23.673000

H2 8.300000 46.637001 24.605000

O 16.257999 32.951000 15.278000

H1 17.121000 32.515999 15.128000

H2 16.532000 33.751999 15.759000

O 24.122000 3.522000 22.540001

H1 23.796000 4.243000 23.121000

H2 24.990000 3.340000 22.930000

O 23.533001 54.391998 57.997002

H1 23.306000 55.312000 58.227001

H2 24.487000 54.375999 58.240002

O 11.077000 51.817001 31.802000

H1 10.670000 51.214001 32.438000

H2 11.990000 51.866001 32.099998

O 63.422001 38.535999 40.766998

H1 63.416000 38.450001 41.736000

H2 62.856998 37.783001 40.505001

O 7.791000 34.631001 46.096001

H1 7.741000 35.277000 46.820999

H2 8.722000 34.340000 46.164001

O 44.681000 16.865000 23.263000

H1 45.604000 16.556999 23.186001

H2 44.792000 17.712000 23.726000

O 29.510000 62.595001 29.316999

H1 29.987000 62.272999 30.094000

H2 28.829000 63.165001 29.718000

O 13.588000 22.170000 28.099001

H1 13.863000 22.844000 28.735001

H2 13.160000 21.506001 28.660000

O 25.256001 11.347000 55.626999

H1 25.584000 11.653000 56.501999

H2 25.934000 10.682000 55.410000

O 40.771000 34.521999 66.653999

H1 41.653999 34.210999 66.941002

H2 40.841999 35.476002 66.831001

O 34.750999 12.276000 19.009001

H1 34.493000 11.906000 19.882000

H2 35.528000 12.810000 19.271000

O 21.077999 49.401001 19.025999

H1 21.302999 48.518002 19.388000

H2 21.282000 49.268002 18.084000

O 12.772000 28.483000 27.197001

H1 13.635000 28.538000 26.742001

H2 12.830000 29.239000 27.811001

O 6.812000 41.012001 35.681000

H1 6.729000 40.827999 36.639000

H2 5.972000 41.445999 35.487000

O 31.736000 60.396000 55.036999

H1 31.080999 61.068001 55.327999

H2 32.569000 60.895000 55.174000

O 10.127000 51.189999 26.695999

H1 9.335000 51.087002 26.134001

H2 10.094000 52.147999 26.889999

O 45.264000 35.209000 68.831001

H1 45.988998 35.237999 69.514000

H2 45.104000 34.247002 68.809998

O 60.297001 40.794998 42.073002

H1 60.687000 40.580002 41.208000

H2 59.861000 39.955002 42.306999

O 15.563000 47.299999 48.638000

H1 15.783000 47.918999 49.355000

H2 14.780000 46.847000 48.980999

O 23.143000 44.952000 60.400002

H1 23.195999 44.900002 61.362000

H2 22.363001 45.535000 60.278000

O 35.521000 59.459000 18.318001

H1 35.917000 60.318001 18.056000

H2 34.808998 59.761002 18.912001

O 18.360001 7.852000 28.497000

H1 18.190001 7.749000 27.551001

H2 17.677999 8.495000 28.757999

O 46.007999 42.587002 46.916000

H1 46.021999 43.158001 46.102001

H2 45.699001 43.268002 47.560001

O 44.099998 14.123000 51.504002

H1 43.473000 14.881000 51.577999

H2 43.995998 13.901000 50.562000

O 40.569000 45.109001 53.515999

H1 39.647999 45.379002 53.370998

H2 40.959999 45.928001 53.876999

O 19.386999 15.480000 42.143002

H1 20.149000 16.083000 42.205002

H2 18.652000 16.042999 42.422001

O 9.351000 42.528000 41.007999

H1 9.310000 42.740002 41.952999

H2 10.143000 43.007000 40.719002

O 52.520000 34.209000 19.754999

H1 52.167999 33.630001 20.445999

H2 52.004002 35.030998 19.906000

O 39.480999 50.905998 44.272999

H1 39.320999 50.789001 43.310001

H2 39.159000 51.827000 44.388000

O 13.114000 26.041000 42.777000

H1 13.933000 26.454000 43.112999

H2 12.438000 26.537001 43.268002

O 10.796000 49.631001 43.271000

H1 10.634000 49.110001 44.091999

H2 10.988000 48.917000 42.641998

O 21.093000 8.848000 28.671000

H1 21.150000 9.063000 27.721001

H2 20.247999 8.363000 28.707001

O 50.813999 54.526001 21.392000

H1 50.382999 55.341000 21.683001

H2 50.907001 54.028999 22.224001

O 40.209000 60.279999 49.144001

H1 39.905998 60.891998 49.837002

H2 40.751999 59.660000 49.664001

O 24.627001 25.549000 60.361000

H1 23.914000 25.083000 59.891998

H2 24.367001 26.476000 60.270000

O 41.615002 12.391000 55.834999

H1 41.630001 12.845000 56.692001

H2 41.868999 11.489000 56.077999

O 51.278999 44.150002 51.092999

H1 50.922001 44.355999 51.985001

H2 51.525002 45.040001 50.786999

O 13.193000 46.424000 24.712000

H1 13.675000 47.266998 24.705999

H2 12.457000 46.638000 25.327000

O 31.524000 1.619000 23.325001

H1 31.424999 2.309000 24.010000

H2 31.093000 0.857000 23.775000

O 19.240999 21.170000 19.552000

H1 19.841000 21.573999 18.903999

H2 19.006001 20.334000 19.136999

O 57.125000 53.145000 38.445000

H1 57.081001 54.070000 38.729000

H2 58.002998 53.103001 38.033001

O 40.792999 19.924000 45.841999

H1 40.104000 20.500999 45.483002

H2 41.118999 20.455000 46.598000

O 24.551001 35.605000 61.550999

H1 24.111000 34.797001 61.222000

H2 24.677000 36.096001 60.715000

O 42.030998 15.643000 29.816999

H1 42.387001 14.766000 29.599001

H2 42.699001 15.989000 30.431000

O 42.514999 46.921001 8.271000

H1 41.619999 46.609001 8.060000

H2 42.966000 46.791000 7.419000

O 37.326000 56.750999 51.723999

H1 36.398998 57.037998 51.686001

H2 37.525002 56.563000 50.797001

O 38.522999 14.228000 31.406000

H1 38.800999 14.326000 30.478001

H2 39.202000 14.737000 31.875000

O 54.368999 11.179000 39.230999

H1 54.319000 11.761000 38.439999

H2 55.264000 11.393000 39.556000

O 22.613001 7.197000 30.846001

H1 22.735001 8.156000 30.910000

H2 21.641001 7.123000 30.798000

O 14.016000 48.183998 32.077000

H1 14.109000 48.108002 33.048000

H2 13.880000 49.145000 31.981001

O 12.949000 10.501000 40.394001

H1 13.465000 10.955000 39.688000

H2 12.879000 9.609000 40.016998

O 27.926001 63.412998 25.813999

H1 27.629000 64.346001 25.878000

H2 27.083000 62.944000 25.766001

O 9.225000 19.004000 45.287998

H1 8.752000 18.403000 44.688999

H2 9.582000 19.667999 44.674999

O 50.053001 31.113001 66.276001

H1 50.152000 30.503000 65.540001

H2 50.973000 31.270000 66.554001

O 32.459999 63.220001 46.820999

H1 32.941002 62.375000 46.800999

H2 31.664000 62.992001 47.321999

O 40.812000 64.517998 38.464001

H1 41.566002 64.648003 37.853001

H2 40.126999 64.200996 37.853001

O 20.815001 54.758999 25.677999

H1 21.082001 53.821999 25.837000

H2 20.599001 54.716000 24.725000

O 24.047001 57.417000 44.555000

H1 24.187000 58.179001 43.966000

H2 24.737000 56.803001 44.252998

O 17.815001 44.287998 23.594999

H1 17.521999 44.264000 24.521999

H2 17.954000 45.249001 23.466000

O 26.489000 58.221001 29.129999

H1 26.952000 58.181999 29.992001

H2 26.848000 59.037998 28.749001

O 58.187000 12.151000 33.661999

H1 57.915001 11.336000 34.118999

H2 58.341000 12.749000 34.415001

O 23.289000 43.897999 63.640999

H1 22.830000 43.705002 64.476997

H2 22.533001 44.019001 63.035999

O 39.643002 19.808001 50.404999

H1 39.345001 19.768000 49.466999

H2 40.374001 19.177999 50.397999

O 52.692001 37.778000 16.000000

H1 52.730000 38.703999 16.319000

H2 53.124001 37.875000 15.124000

O 20.283001 16.898001 56.827000

H1 19.802000 16.112000 57.113998

H2 21.098000 16.513000 56.453999

O 59.575001 37.058998 25.412001

H1 60.146000 36.277000 25.538000

H2 59.992001 37.706001 25.996000

O 57.085999 49.995998 30.615999

H1 57.084999 49.085999 30.266001

H2 57.713001 49.914001 31.358999

O 62.054001 40.638000 30.246000

H1 61.721001 39.737000 30.230000

H2 61.269001 41.166000 30.049999

O 20.563000 11.863000 46.018002

H1 20.712000 11.451000 46.886002

H2 21.125999 11.297000 45.442001

O 59.646000 34.549999 22.098000

H1 59.397999 33.925999 21.396000

H2 60.555000 34.282001 22.299999

O 15.672000 13.016000 28.754000

H1 14.996000 13.713000 28.615999

H2 15.882000 13.151000 29.700001

O 11.930000 31.502001 46.792999

H1 12.390000 32.118000 47.376999

H2 12.371000 31.674999 45.932999

O 17.455000 18.900000 36.067001

H1 17.292999 18.238001 36.755001

H2 18.087000 19.503000 36.516998

O 49.361000 30.480000 21.108999

H1 49.355999 31.351000 21.561001

H2 50.083000 30.590000 20.476000

O 28.106001 45.974998 56.493999

H1 28.959000 46.391998 56.280998

H2 28.375999 45.049999 56.653999

O 18.752001 23.583000 61.730999

H1 18.701000 23.458000 62.702999

H2 17.917999 23.162001 61.452999

O 53.040001 12.435000 43.646999

H1 52.261002 12.510000 43.077999

H2 52.664001 12.093000 44.473000

O 56.723999 19.561001 45.313000

H1 57.354000 20.294001 45.449001

H2 55.876999 20.004000 45.495998

O 33.228001 36.254002 7.188000

H1 32.516998 35.909000 6.610000

H2 32.827999 37.076000 7.515000

O 12.033000 20.122999 37.771000

H1 11.409000 19.464001 38.109001

H2 12.072000 20.763000 38.509998

O 19.563000 25.197001 50.962002

H1 19.441000 25.638000 50.098000

H2 18.687000 24.816000 51.118999

O 54.590000 59.959000 25.854000

H1 54.837002 59.021000 25.812000

H2 54.335999 60.155998 24.945999

O 43.027000 22.756001 63.655998

H1 42.374001 23.120001 64.271004

H2 42.676998 23.010000 62.792000

O 23.871000 51.668999 20.513000

H1 23.525999 52.007000 21.372999

H2 23.153999 51.952999 19.914000

O 46.919998 62.557999 30.754000

H1 46.312000 61.998001 30.249001

H2 47.411999 61.902000 31.278000

O 49.648998 48.319000 39.984001

H1 49.827000 49.286999 40.008999

H2 50.213001 48.070999 39.217999

O 39.226002 56.037998 15.138000

H1 39.368999 56.313999 14.214000

H2 38.256001 55.959000 15.170000

O 20.353001 4.810000 28.101000

H1 20.153999 5.230000 28.969000

H2 19.917000 3.953000 28.197001

O 53.903000 20.101000 24.847000

H1 53.941002 19.589001 24.021999

H2 54.265999 19.475000 25.493000

O 27.556999 64.346001 49.827999

H1 28.322001 63.991001 49.334000

H2 27.819000 64.175003 50.744999

O 49.898998 41.948002 11.759000

H1 50.134998 42.069000 12.701000

H2 49.505001 41.041000 11.797000

O 25.771999 54.780998 61.275002

H1 26.636999 54.509998 61.634998

H2 25.818001 54.375999 60.381001

O 9.687000 40.898998 27.246000

H1 9.866000 40.264000 26.525999

H2 9.414000 41.685001 26.725000

O 42.821999 42.445999 16.285000

H1 42.411999 43.162998 16.805000

H2 42.439999 41.660999 16.738001

O 21.775000 41.544998 57.216000

H1 22.177999 41.073002 56.476002

H2 20.823000 41.387001 57.068001

O 18.957001 52.202999 61.589001

H1 19.545000 51.433998 61.660999

H2 18.079000 51.796001 61.680000

O 9.647000 42.272999 61.178001

H1 8.732000 42.000000 60.998001

H2 10.151000 41.727001 60.561001

O 42.283001 55.424999 39.252998

H1 41.581001 56.102001 39.366001

H2 41.896000 54.869999 38.550999

O 54.674999 26.753000 58.897999

H1 55.513000 27.174999 59.154999

H2 54.126999 27.525000 58.672001

O 33.695000 25.427999 10.786000

H1 33.783001 25.802000 9.892000

H2 34.568001 25.000999 10.897000

O 13.298000 46.375999 53.365002

H1 13.256000 47.316002 53.108002

H2 12.380000 46.210999 53.634998

O 19.667000 45.115002 63.936001

H1 20.284000 45.069000 64.695000

H2 18.851000 45.417999 64.380997

O 51.666000 57.625000 38.776001

H1 52.042000 58.048000 39.573002

H2 51.245998 58.401001 38.347000

O 48.716999 53.557999 41.183998

H1 48.504002 54.035999 40.368000

H2 47.813000 53.373001 41.528000

O 40.560001 47.327000 47.159000

H1 41.430000 46.942001 46.962002

H2 40.264999 47.610001 46.272999

O 13.712000 49.231998 47.222000

H1 13.898000 50.180000 47.097000

H2 14.543000 48.820000 46.952000

O 49.771999 9.173000 25.426001

H1 50.028999 9.226000 26.351999

H2 50.362000 9.803000 24.996000

O 7.119000 40.562000 38.304001

H1 6.803000 40.952999 39.143002

H2 8.036000 40.877998 38.272999

O 7.190000 25.450001 35.855999

H1 7.602000 25.180000 36.688999

H2 6.705000 24.636999 35.605000

O 39.590000 21.291000 10.813000

H1 39.264000 21.318001 11.726000

H2 39.039001 20.608000 10.411000

O 15.027000 25.115000 55.438999

H1 15.363000 25.572001 54.660999

H2 15.835000 24.938999 55.950001

O 13.453000 53.594002 37.612000

H1 12.751000 53.875999 37.008999

H2 13.756000 54.428001 37.987000

O 50.816002 44.362999 10.610000

H1 50.481998 43.647999 11.182000

H2 51.770000 44.342999 10.817000

O 33.731998 61.672001 30.277000

H1 33.258999 60.868000 30.007000

H2 33.193001 61.960999 31.042999

O 55.486000 22.532000 27.495001

H1 55.407001 23.063999 28.297001

H2 55.025002 21.712000 27.746000

O 16.962000 33.355000 24.802999

H1 16.761999 34.120998 25.372999

H2 16.181999 33.323002 24.231001

O 39.471001 58.909000 26.700001

H1 38.888000 58.333000 26.184000

H2 39.625000 58.369999 27.497000

O 51.736000 38.446999 41.973000

H1 52.398998 38.747002 42.627998

H2 52.217999 37.687000 41.571999

O 35.548000 53.372002 55.806999

H1 34.823002 53.150002 55.176998

H2 35.796001 54.258999 55.500000

O 25.643000 53.080002 31.568001

H1 25.514999 52.785999 32.480999

H2 26.516001 52.683998 31.354000

O 48.158001 51.146999 24.561001

H1 47.256001 51.535000 24.523001

H2 48.615002 51.792000 25.118999

O 29.204000 6.050000 19.181000

H1 28.667999 6.065000 19.988001

H2 30.084000 5.829000 19.528999

O 23.090000 4.726000 48.729000

H1 22.139999 4.705000 48.938000

H2 23.459999 4.223000 49.476002

O 29.704000 22.646000 57.132000

H1 30.608999 22.312000 57.216999

H2 29.292999 21.997000 56.541000

O 15.853000 49.367001 37.986000

H1 15.010000 49.251999 37.522999

H2 16.055000 50.304001 37.793999

O 34.501999 13.061000 51.641998

H1 33.931999 12.935000 50.875000

H2 34.488998 14.032000 51.743999

O 37.627998 18.514999 11.166000

H1 38.020000 17.896000 10.505000

H2 37.105999 19.091999 10.583000

O 44.629002 48.294998 41.553001

H1 44.806000 47.862999 42.417999

H2 45.528999 48.588001 41.318001

O 40.407001 62.527000 28.368000

H1 39.827000 61.941002 27.863001

H2 41.198002 62.573002 27.820000

O 10.397000 37.265999 48.617001

H1 11.335000 37.044998 48.791000

H2 10.285000 36.932999 47.713001

O 54.639000 32.327999 18.837000

H1 53.872002 32.778999 19.240000

H2 54.283001 32.132000 17.945000

O 20.016001 57.570999 19.663000

H1 20.365999 56.785999 20.114000

H2 20.422001 58.292000 20.181999

O 34.594002 23.250999 60.994999

H1 33.660000 23.042999 60.801998

H2 34.743999 24.014000 60.396999

O 31.386000 4.901000 20.923000

H1 30.893000 4.089000 21.090000

H2 31.462000 5.293000 21.812000

O 51.486000 34.355000 52.963001

H1 52.068001 34.265999 53.738998

H2 52.036999 34.881001 52.356998

O 14.251000 23.082001 17.597000

H1 13.607000 22.636999 18.184000

H2 15.081000 22.961000 18.097000

O 23.160000 9.930000 47.341000

H1 22.952000 9.569000 48.224998

H2 22.770000 9.246000 46.765999

O 54.750999 54.560001 42.932999

H1 55.152000 55.449001 42.925999

H2 54.730000 54.354000 41.978001

O 47.508999 51.205002 18.704000

H1 47.259998 51.227001 19.656000

H2 48.487999 51.278000 18.784000

O 13.106000 49.687000 21.323000

H1 13.168000 49.844002 20.367001

H2 12.868000 50.574001 21.650999

O 18.813999 27.172001 59.277000
[truncated: 8,450,399 more chars]
