# Supplementary material for: Conformational Change of H64 and Substrate Transportation: Insight Into a Full Picture of Enzymatic Hydration of CO2 by Carbonic Anhydrase
Source: Front Chem. 2021 Jul 9;9:706959. doi: 10.3389/fchem.2021.706959 (PMC8299336; doi:10.3389/fchem.2021.706959)
Supplement: Supplementary file 2 [file DataSheet1.docx]

Supplementary Material

**Conformational change of His64 and substrate transportation: Insight into a full picture of** **enzymatic hydration of CO_2_ by carbonic anhydrase**

Yuzhuang Fu^1^, Fangfang Fan^2^, Yuwei Zhang^1^, Binju Wang^1*^, Zexing Cao^1*^

^1^State Key Laboratory of Physical Chemistry of Solid Surfaces and Fujian Provincial Key Laboratory of Theoretical and Computational Chemistry, College of Chemistry and Chemical Engineering, Xiamen University, Xiamen, China

^2^School of Biological and Chemical Engineering, Zhejiang University of Science and Technology, Hangzhou, China

*** Correspondence:**Corresponding Author
wangbinju2018@xmu.edu.cn; zxcao@xmu.edu.cn

**Contents**

Figure S1 ……………………………………………………………………………………………3

Figure S2 ……………………………………………………………………………………………3

Table S1 ……………………………………………………………………………………………3

Figure S3 ……………………………………………………………………………………………4

Figure S4 ……………………………………………………………………………………………4

Figure S5 ……………………………………………………………………………………………5

Figure S6 ……………………………………………………………………………………………5

Figure S7 ……………………………………………………………………………………………6

xyz files ……………………………………………………………………………………………6


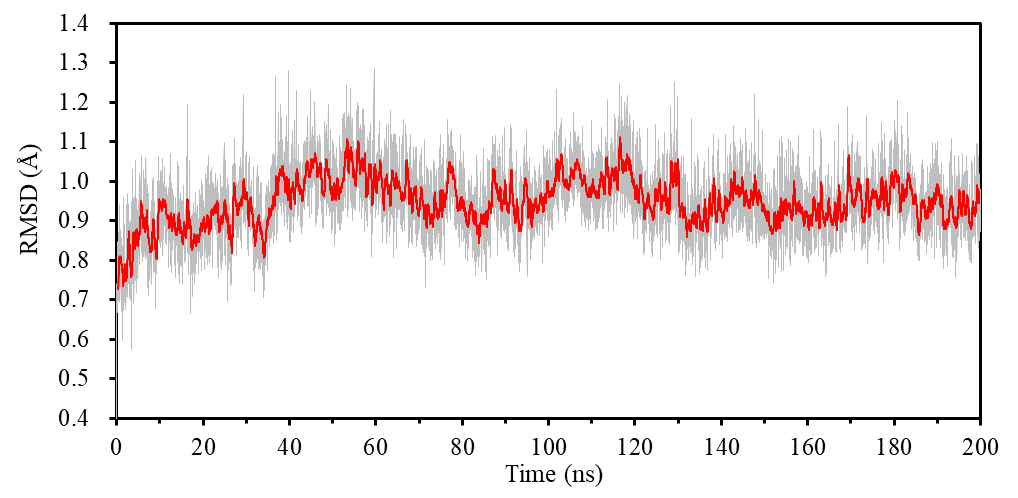


**Figure S1.** RMSD values of CA enzyme without CO_2_ during MM MD simulations.

**Table S1.** Probability of water molecules in the active domain.

|  | WT2_T199@OG1 | WT3_H64@ND1 | DW_T200@H |
| --- | --- | --- | --- |
| 1.8 Å | 24.20% | 47.92% | 28.64% |
| 2.2 Å | 92.40% | 76.96% | 64.44% |


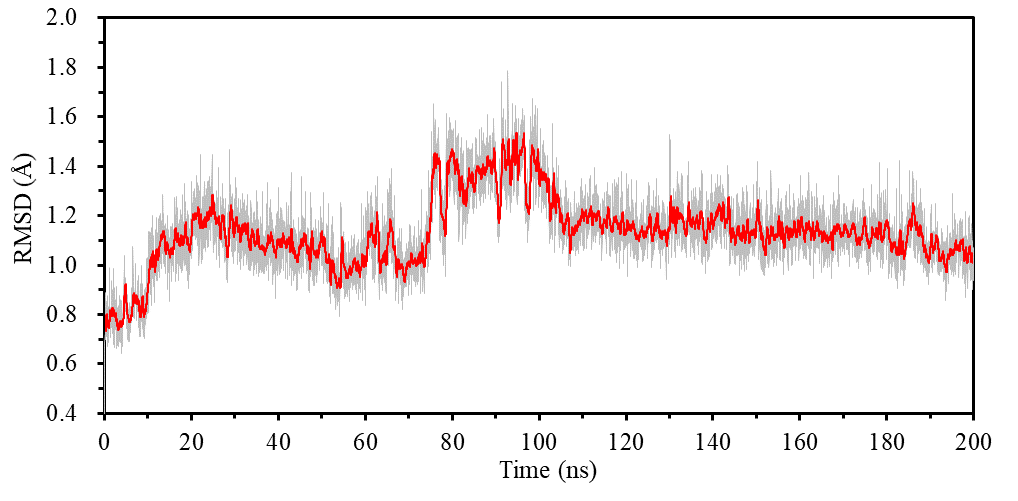


**Figure S2.** RMSD values of the CA-CO_2_ complex system during MM MD simulations.


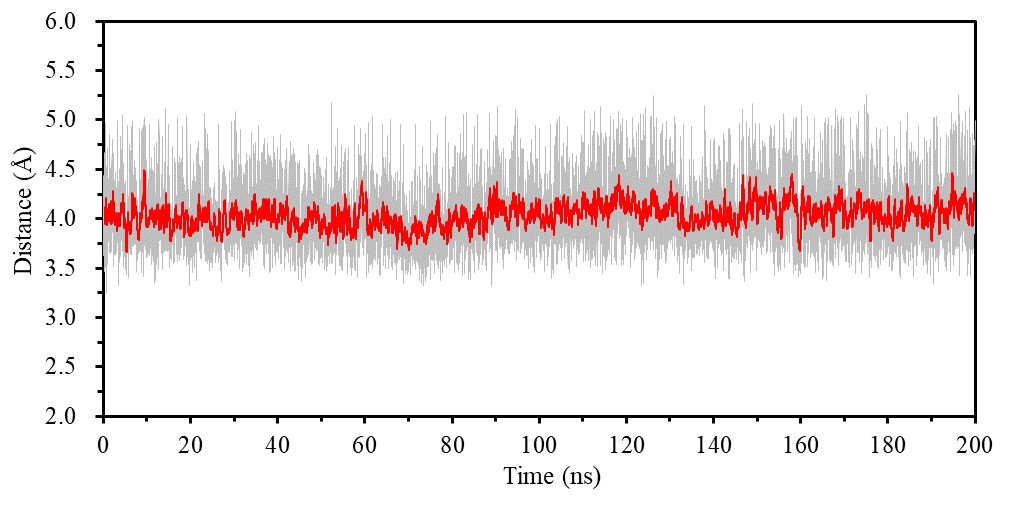


**Figure S3.** Statistics of distance between the zinc ion and CO_2_@C during MM MD simulations.


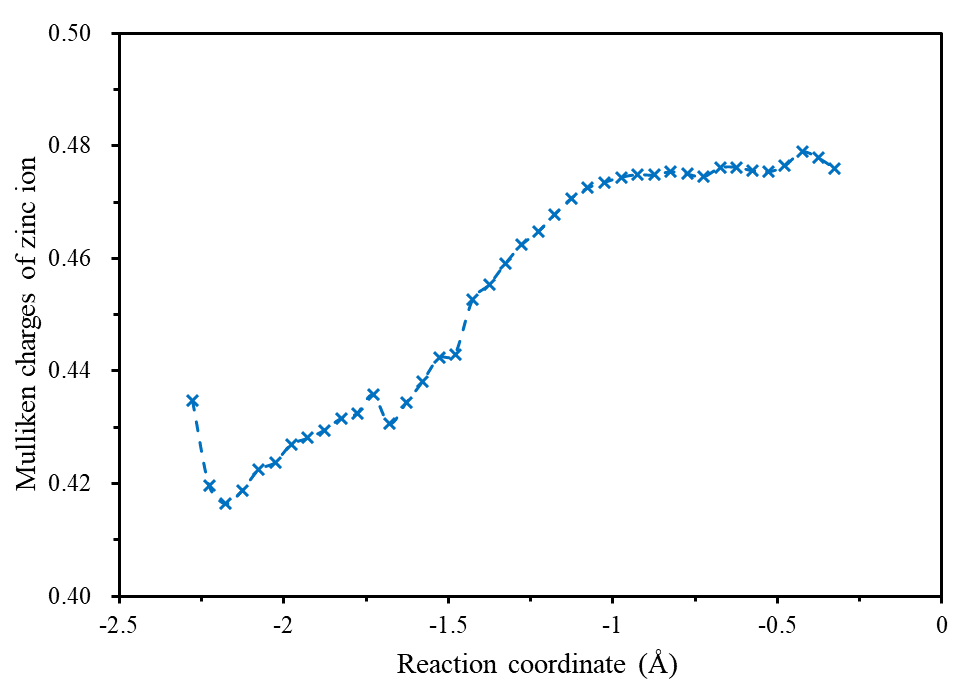


**Figure S4.** The Mulliken charges of the zinc ion during the proton transfer from Zn-bound water to His64 through a water chain.


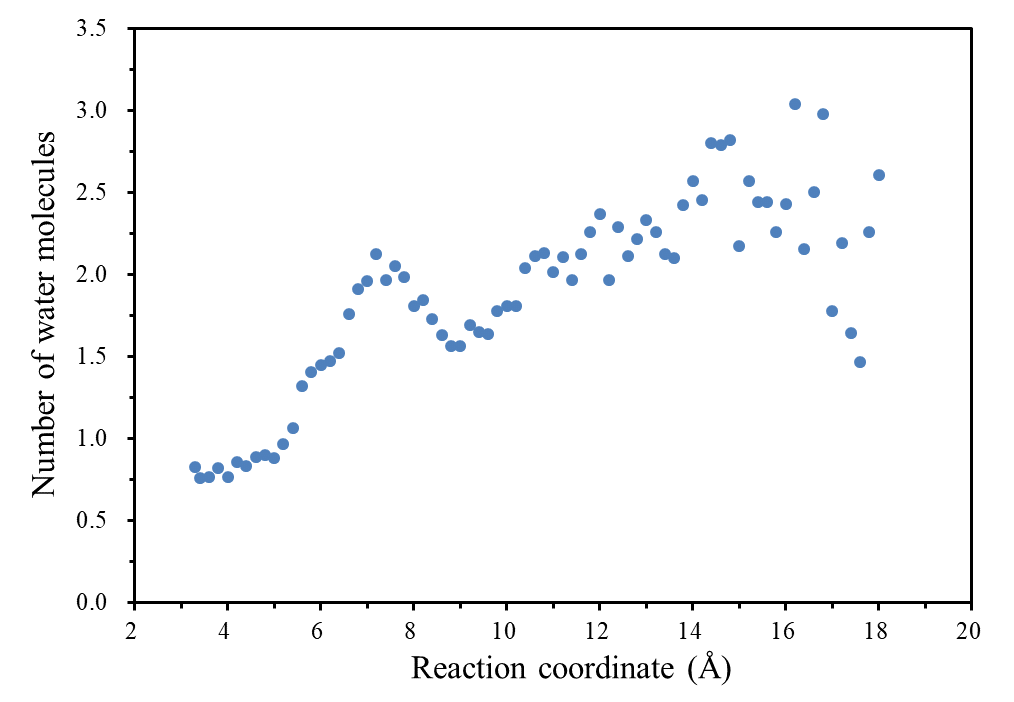


**Figure S5.** Statistics analysis of water molecules around CO_2_ within 3 Å during the delivery of CO_2_ from the bulk water to the active site of CA.


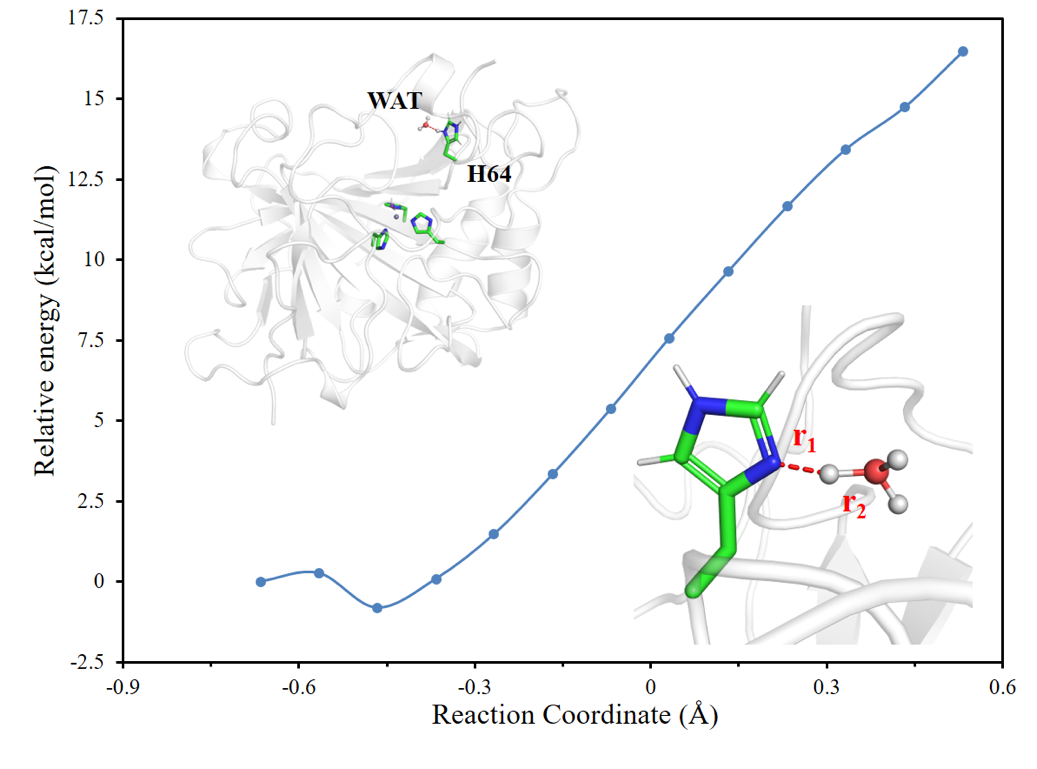


**Figure S6.** QM(B3LYP/TZVP–D3)/MM-predicted relative energy profile for the proton transfer from H64 to the water molecule which form hydrogen bond with H64@HD1.


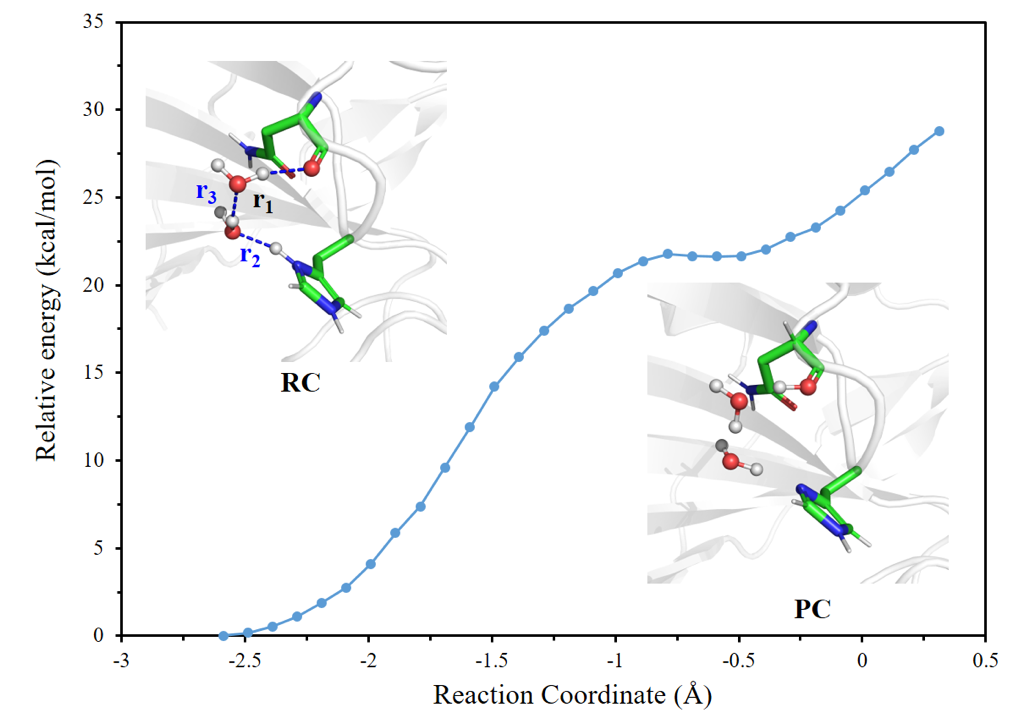


**Figure S7.** QM(B3LYP/TZVP–D3)/MM-predicted relative energy profile for the proton transfer from H64 to N62@O, where the reaction coordinate is defined as r_1_ – r_2_ – r_3_.

The xyz files of the key states are provided as the supplementary materials (shown in another word file):

Reactant complex (RC), transition state (TS) and product (PC) for the proton transfer from the zinc-bound water molecule and H64:

RC_PT.xyz; TS_PT.xyz; PC_PT.xyz

Reactant complex (RC), transition state (TS) and product (PC) for the hydration of CO_2_:

RC_hy.pdb; TS_hy.xyz; PC_hy.xyz
